# Dual Nickel/Photoredox‐Catalyzed Site‐Selective Cross‐Coupling of 1,2‐Bis‐Boronic Esters Enabled by 1,2‐Boron Shifts

**DOI:** 10.1002/anie.202207988

**Published:** 2022-07-14

**Authors:** Hui Wang, Wangyujing Han, Adam Noble, Varinder K. Aggarwal

**Affiliations:** ^1^ School of Chemistry University of Bristol Cantock's Close Bristol BS8 1TS UK; ^2^ Key Laboratory of Functional Molecular Solids (Ministry of Education) Anhui Key Laboratory of Molecular Based Materials College of Chemistry and Materials Science Anhui Normal University Wuhu 241002 China

**Keywords:** Boronic Esters, Cross-Coupling, Dual Catalysis, Nickel, Photoredox Catalysis

## Abstract

Site‐selective transition‐metal‐catalyzed mono‐deboronative cross‐couplings of 1,2‐bis‐boronic esters are valuable methods for the synthesis of functionalized organoboron compounds. However, such cross‐couplings are limited to reaction of the sterically less hindered primary boronic ester. Herein, we report a nickel/photoredox‐catalyzed mono‐deboronative arylation of 1,2‐bis‐boronic esters that is selective for coupling of the more sterically hindered secondary/tertiary position. This is achieved by taking advantage of a 1,2‐boron shift of primary β‐boryl radicals to the thermodynamically favored secondary/tertiary radicals, which are subsequently intercepted by the nickel catalyst to enable arylation. The mild conditions are amenable to a broad range of aryl halides to give β‐aryl boronic ester products in good yields and with high regioselectivity. This method also allows stereodivergent coupling of cyclic *cis*‐1,2‐bis‐boronic esters to give *trans*‐substituted products.

## Graphical abstract

A visible‐light‐induced nickel/photoredox‐catalyzed mono‐Suzuki–Miyaura cross‐coupling of 1,2‐bis‐boronic esters has been accomplished, which is selective for the most sterically hindered boronic ester. High regioselectivities were achieved through a facile radical 1,2‐boron shift process, wherein the initially generated primary β‐boryl radical is rapidly converted to the thermodynamically more stable secondary radical before nickel‐catalyzed cross‐coupling.

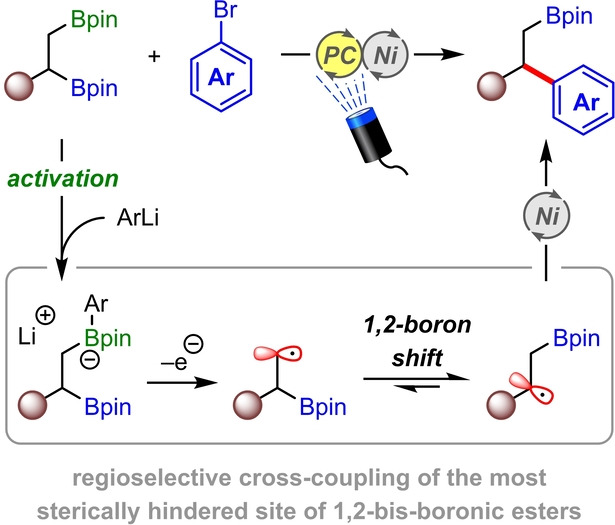

## Introduction

**Figure 1:**
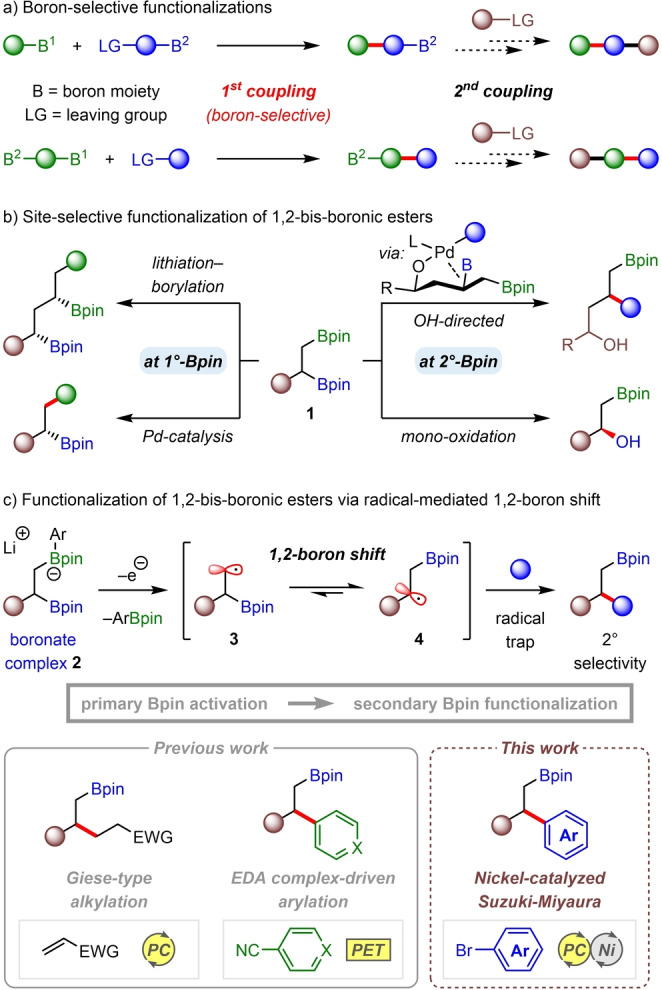
Site‐selective reactions of organoboron compounds. EDA= electron donor–acceptor complex; PC=photocatalyst; PET=photo‐ induced electron transfer.

Organoboron compounds are some of the most valuable building blocks in organic synthesis because of the unique reactivity of C−B bonds.^[1]^ A particularly powerful strategy in organoboron chemistry is the use of boron‐selective reactions, wherein the reactants possess two or more boron moieties that exhibit different reactivities (Figure 1a), thus enabling the concise synthesis of complex molecules via multiple C−C bond‐forming steps.^[2]^ For example, boron‐selective Suzuki–Miyaura cross‐couplings have been developed that employ a boronic acid protecting group, such as 1,8‐diaminonaphthalene (DAN) or *N*‐methyliminodiacetic acid (MIDA) ligands, which makes one of the boron moieties inactive towards transmetalation under palladium catalysis.^[3]^ Recently, chemists have turned their attention to boron‐selective reactions of 1,2‐bis‐boronic esters **1**, which are useful synthetic intermediates that are easily prepared by diboration of alkenes.^[4]^ For these substrates, boron protecting groups are not necessary because the two boronic esters are distinguishable by their different steric environments, thus enabling selective mono‐functionalization reactions of the primary boronic ester over the sterically more hindered secondary/tertiary position, including in Suzuki–Miyaura cross‐couplings and homologations with chiral carbenoids (Figure 1b, left).[^5^, ^6^]

In contrast to primary‐selective mono‐functionalizations of 1,2‐bis‐boronic esters, the development of reactions that display high selectivity for the more sterically hindered secondary/tertiary boronic ester remains a significant challenge. Strategies that have been employed to overcome the inherent steric bias include Morken's β‐hydroxy‐directed palladium‐catalyzed Suzuki–Miyaura cross‐coupling, which displays high selectivity for secondary over primary boronic esters;^[7]^ and the same group took advantage of the greater migratory aptitude of secondary over primary alkyl groups to develop a secondary‐selective mono‐oxidation using trimethylamine *N*‐oxide as the oxidant (Figure 1b, right).^[8]^ While these strategies are effective, they are either limited to substrates with pre‐installed directing groups or are only effective for simple C−B bond oxidation, which limits their broader applications. In this regard, developing a general approach towards mono‐selective functionalizations of the more hindered positions of 1,2‐bis‐boronic esters remains a highly desirable but unmet goal.

Recently, we developed a deboronative alkyl radical formation that involves the activation of pinacol boronic esters with aryllithium reagents to generate arylboronate complexes.^[9]^ These electron‐rich boron species undergo facile single‐electron oxidation and C(sp^3^)−B bond cleavage to form alkyl radicals under photoredox catalysis. Interestingly, when applied to Giese‐type reactions of 1,2‐bis‐boronic esters, despite selective activation of the primary boronic ester to form boronate complex **2**, the products were formed with complete selectivity for functionalization of the secondary boronic ester (Figure 1c).^[10]^ This selectivity arises from a facile 1,2‐boron shift of the primary β‐boryl radical intermediate **3** to give the more thermodynamically stable secondary β‐boryl radical **4**,^[11]^ which is then trapped by an electron‐deficient alkene. We also demonstrated that 1,2‐boron shifts could be used to achieve secondary/tertiary selective mono‐arylations of 1,2‐bis‐boronic esters by intercepting β‐boryl radical **4** with radical‐anions of arylnitriles,^[12]^ thus providing the opposite regioselectivity to Suzuki–Miyaura reactions.^[5]^ However, the success of this reaction is contingent on the use of highly electron‐deficient arylnitriles to facilitate the formation of the key aryl radical‐anion intermediates, which limits the scope of aromatic coupling partners that can be used in this transformation. To overcome this limitation and provide a more complementary methodology to palladium‐catalyzed cross‐couplings, we sought to intercept β‐boryl radical **4** in a transition metal‐catalyzed cross coupling with aryl halides (Figure 1c).

In recent years, dual nickel‐ and photoredox‐catalysis has been demonstrated to be a valuable synthetic tool in organic synthesis for cross‐coupling of alkyl radicals with halide electrophiles.^[13]^ In these reactions, the alkyl radicals can either be generated directly, by homolytic C(sp^3^)−X bond cleavage of a radical precursor,^[14]^ or indirectly, in processes such as conjunctive reactions with alkenes,^[15]^ cyclizations,^[16]^ β‐scissions,^[17]^ or hydrogen atom transfers (HAT).^[18]^ However, dual nickel and photoredox catalysis has not yet been applied to cross‐couplings of alkyl radicals resulting from 1,2‐boron shifts. Achieving this would significantly expand the scope of our boron‐selective reactions of 1,2‐bis‐boronic esters from electron‐deficient (hetero)arylnitriles to widely available (hetero)aryl halides. Furthermore, it would provide a complementary methodology to palladium‐catalyzed Suzuki–Miyaura cross‐couplings that uses the same reactants but in a mechanistically distinct process that results in a reversal of regioselectivity. Given that transition metal‐catalyzed cross‐couplings are often sensitive to sterics, we hypothesized that the key to developing a highly regioselective reaction would be to identify a nickel catalyst capable of intercepting the thermodynamically favored but sterically more hindered β‐boryl radical **4**, despite the accessibility of the sterically more accessible primary β‐boryl radical **3** via facile 1,2‐boron shift. Herein, we describe the successful realization of a dual nickel‐ and photoredox‐catalyzed cross‐coupling reaction of 1,2‐bis‐boronic esters with (hetero)aryl halides that proceeds with high regioselectivity for arylation of the more sterically hindered boronic ester group (Figure 1c).

## Results and Discussion

We began our studies by investigating the cross‐coupling of boronate complex **2 a**, derived from 1,2‐bis‐boronic ester **1 a** and phenyllithium,^[9]^ with 4‐bromobenzonitrile (**5 a**) (Table 1). We selected Ni(TMHD)_2_ as a catalyst because Molander has previously shown the THMD (2,2,6,6‐tetramethyl‐3,5‐heptanedionate) ligand is effective for nickel‐catalyzed cross‐couplings of sterically hindered alkyl radicals.^[19]^ Thus, a mixture of **2 a** and **5 a** in CH_3_CN was treated with 10 mol % Ni(TMHD)_2_ and 5 mol % of the organic photoredox catalyst 4CzIPN and then irradiated with blue‐light, which delivered the cross‐coupled product **6 aa** in 45 % yield and with 8 : 1 regioselectivity favoring reaction at the secondary boronic ester (entry 1). Switching to Lewis basic solvents, such as DMF and DMA, led to dramatic increases in both the yield and regioselectivities (entries 2 and 3). Other solvents were also investigated, but did not provide any further improvements (entries 4–6). The use of different ligands on the nickel catalyst had a significant effect on the regioselectivity of the reaction, with anionic diketonate‐based ligands, TMHD and acetylacetonate (acac), providing significantly higher secondary selectivity than bipyridine ligands (entries 2 and 7–9). This dramatic effect of the ligand type on regioselectivity is likely a result of the switch in mechanism for reductive elimination from outer‐sphere to inner‐sphere upon changing from anionic diketonate‐based ligands to neutral bipyridine ligands, which was previously described by Molander and Gutierrez.^[20]^ Interestingly, decreasing the equivalents of **5 a** from 3 to 1.5 resulted in an increase in regioselectivity, while maintaining an excellent yield of 92 % (entries 2, 10, and 11). The yield of **6 aa** was significantly reduced when the boronate complex was generated by activation of **1 a** with methyllithium instead of phenyllithium (entry 12). Finally, control experiments demonstrated that the nickel catalyst, photocatalyst, and light are all essential for the reaction (entries 13–15).

**Table 1:** Optimization studies.^[a]^

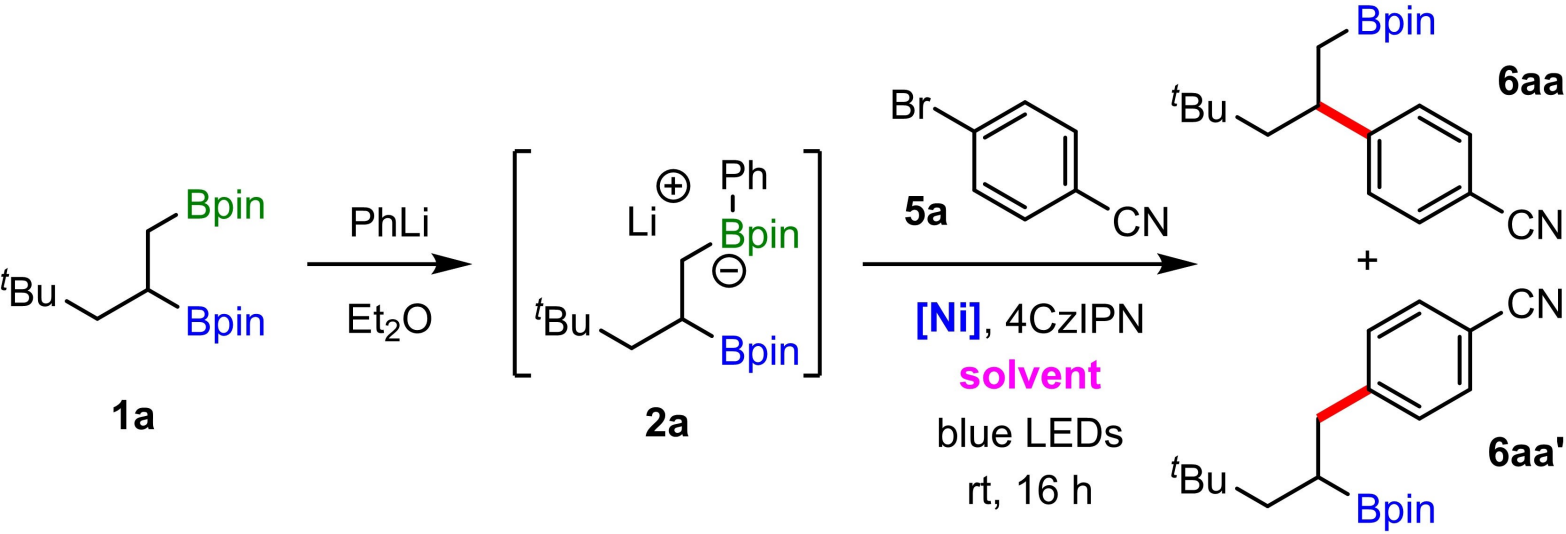				
Entry	[Ni]	Solvent	Yield of **6 aa**	*r.r*. (**6 aa : 6 aa′**)
1	Ni(TMHD)_2_	CH_3_CN	45 %	8 : 1
2	Ni(TMHD)_2_	DMF	92 %	16 : 1
3	Ni(TMHD)_2_	DMA	84 %	16 : 1
4	Ni(TMHD)_2_	THF	34 %	>20 : 1
5	Ni(TMHD)_2_	NMP	56 %	20 : 1
6	Ni(TMHD)_2_	Toluene	11 %	13 : 1
7	Ni(acac)_2_	DMF	81 %	>20 : 1
8	Ni(bpy)Br_2_	DMF	72 %	3 : 1
9	NiCl_2_⋅glyme/dtbbpy	DMF	40 %	3 : 1
10^[b]^	Ni(TMHD)_2_	DMF	92 %	18 : 1
**11** ^[c]^	**Ni(TMHD)_2_**	**DMF**	**92 % (81 %)** ^[d]^	**20 : 1**
12^[c,e]^	Ni(TMHD)_2_	DMF	40 %	15 : 1
13^[c]^	–	DMF	0 %	–
14^[c,f]^	Ni(TMHD)_2_	DMF	0 %	–
15^[c,g]^	Ni(TMHD)_2_	DMF	0 %	–

[a] **1 a** (0.2 mmol), PhLi (1.1 equiv), **5 a** (3.0 equiv), 4CzIPN (5.0 mol %), [Ni] (10 mol %), solvent (2.0 mL); yields and regiomeric ratios (r.r.) were determined by GC‐FID analysis using 1,3,5‐trimethoxybenzene as an internal standard. [b] **5 a** (2.0 equiv). [c] **5 a** (1.5 equiv). [d] Yield of isolated product. [e] MeLi instead of PhLi. [f] Without photocatalyst. [g] Without light.

With optimized conditions in hand, we examined the scope of this site‐selective coupling with respect to the aryl bromides (Table 2). Aryl bromides containing electron‐withdrawing groups reacted efficiently to provide the corresponding primary β‐aryl boronic esters **6 aa**–**6 ag** in moderate to good yields and with excellent regioselectivities. Furthermore, the synthesis of **6 aa** was performed on a 2 mmol scale with similar efficiency, thus demonstrating the scalability of the coupling reaction. Products derived from some electron‐neutral aryl bromides (**6 ah**–**6 ak**) were also formed in synthetically useful yields. However, reactions of electron‐rich aryl bromides were low‐yielding due to the formation of inactive nickel‐black.^[21]^ Pleasingly, replacing the aryl bromide with the corresponding iodide enabled formation of the desired products in moderate yields and with complete regioselectivity (**6 al**–**6 ao**). The reaction showed excellent functional group tolerance, allowing the site‐selective installation of aromatic groups substituted with cyano, trifluoromethyl, ketone, ester, amide, fluoride, and chloride groups. *meta*‐Substituted aryl bromides/iodides, bearing electron‐withdrawing (**6 ap**–**6 at**) and electron‐donating (**6 au**) groups, were also successfully engaged in the reaction with excellent regioselectivity. Coupling with sterically encumbered *ortho*‐substituted aryl bromides was also possible, providing **6 av** and **6 aw** in 31 % and 56 %, respectively. Notably, the use of heteroaryl halides enabled the highly regioselective installation of various heterocycles, such as pyridine (**6 ax** and **6 ay**), pyrimidine (**6 az** and **6 aaa**), dihydrobenzofuran (**6 aab**), and benzothiazole (**6 aac**). Lastly, we demonstrated that alkenyl bromides were also effective coupling partners, with homoallylic boronic ester **6 aad** formed in moderate yield and high regioselectivity.

**Table 2:**
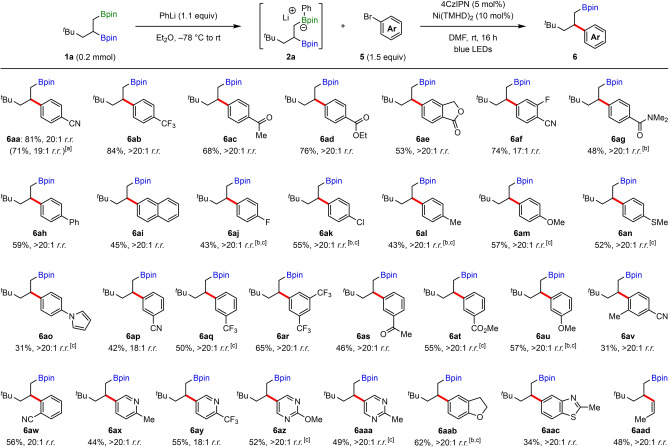
Scope of aryl halides.

[a] Using 2.0 mmol of **1 a**. [b] Isolated as the corresponding alcohol after oxidation. [c] Reaction performed with the corresponding aryl iodide.

We next turned our attention to the scope of 1,2‐bis‐boronic esters (Table 3). Unfortunately, upon replacing the *tert*‐butyl group of **1 a** with a trimethyl silyl (TMS) group, arylated product **6 ba** was formed in only 32 % yield, albeit with excellent regioselectivity. To our delight, we found that substituting phenyllithium for [4‐(dimethylamino)phenyl]lithium in the boronic ester activation step (forming **2**) resulted in a significant increase in yield of **6 ba** to 70 %.^[12]^ Interestingly, when investigating a range of other primary alkene‐derived 1,2‐bis‐boronic esters with groups of varying size at the β‐position to the secondary boronic ester, a clear downward trend in the regioselectivity was observed with sterically less demanding groups (**6 ca**–**6 ea**). For example, when the β‐substituent was changed from *tert*‐butyl (**6 aa**) to *iso*‐propyl (**6 da**) to ethyl (**6 ea**), the selectivity for arylation of the secondary boronic ester decreased from 20 : 1 to 6 : 1 to 4 : 1. This seemingly counterintuitive regioselectivity trend could result from a change in the mechanism of reductive elimination between the equilibrating primary and secondary β‐boryl radicals and the nickel catalyst.^[20]^ Whilst sterically hindered substrates (e.g., **6 aa** and **6 ba**) proceed through the expected outer‐sphere pathway, for less hindered substrates (e.g., **6 da** and **6 ea**) a more facile inner‐sphere pathway via the primary β‐boryl radical becomes competitive (vida infra).

**Table 3:**
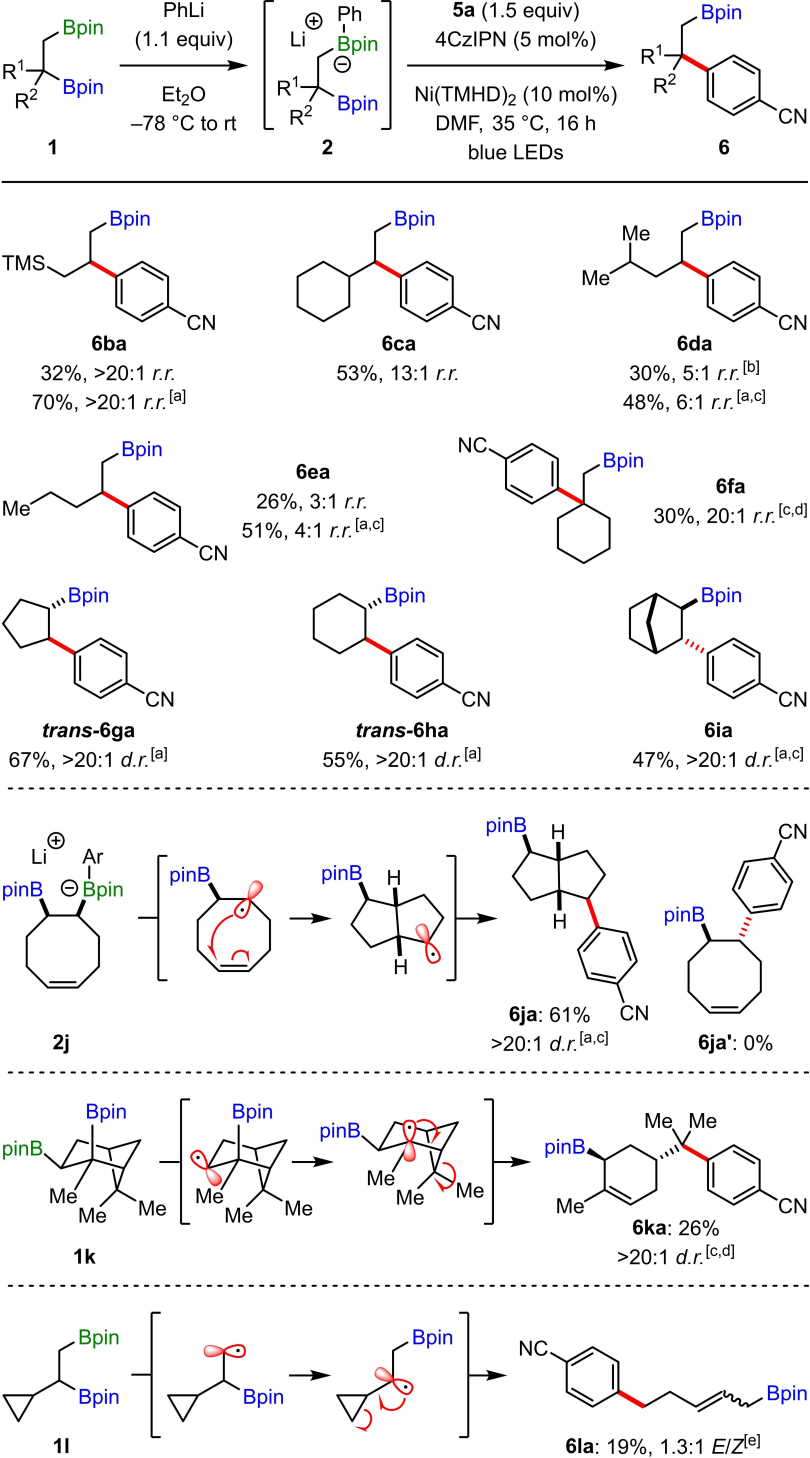
Scope of 1,2‐bis‐boronic esters.

[a] Boronic ester activation performed with [4‐(dimethylamino)phenyl]lithium instead of PhLi. [b] Yield was determined by ^1^H NMR analysis using 1,3,5‐trimethxoylbenzene as an internal standard. [c] Yield of the major isomer, isolated after oxidation. [d] Boronic ester activation performed with MeLi instead of PhLi, and coupling performed with **2 a** (3.0 equiv) and CH_3_CN as solvent. [e] Yield of the corresponding alcohol, isolated after oxidation.

We subsequently investigated the use of 1,2‐bis‐boronic esters derived from a 1,1‐disubstituted alkene and were pleased to observe that β‐aryl boronic ester **6 fa** was formed with excellent regioselectivity for arylation of the tertiary boronic ester (Table 3). For this substrate, we found that optimum yield was obtained when the boronic ester activation was performed with methyllithium, and when CH_3_CN was used as solvent in the cross‐coupling. Our cross‐coupling conditions could also be applied to cyclic *cis*‐1,2‐bis‐boronic esters, providing the *trans*‐products in moderate to good yields and with excellent diastereoselectivity (**6 ga**–**6 ia**), thus providing the opposite selectivity to Pd‐catalyzed cross‐couplings, which yield *cis*‐products due to stereoretentive transmetalation (see Supporting Information).^[12]^ Further extension of the scope of cyclic *cis*‐1,2‐bis‐boronic esters was illustrated in three radical cascade reactions. Boronate complex **2 j** formed from diborated cyclooctadiene and [4‐(dimethylamino)phenyl]lithium afforded arylated *cis*‐bicyclo[3.3.0]octane **6 ja** in excellent diastereoselectivity and as the only observed product, thus demonstrating that transannular cyclization (rate=3.3×10^4^ s^−1^)^[22]^ outcompetes interception of the initial β‐boryl radical by the nickel catalyst. 1,2‐Bis‐boronic ester **1 k**, derived from (−)‐α‐pinene, underwent a 1,2‐boron shift and cyclobutane ring opening to give cyclohexenyl boronic ester **6 ka** as a single diastereomer, reflecting a stereospecific 1,2‐boron shift process. Finally, the reaction of cyclopropane‐containing substrate **1 l** yielded allyl boronic ester **6 la** via a similar 1,2‐boron shift/ring opening cascade.

**Scheme 1:**
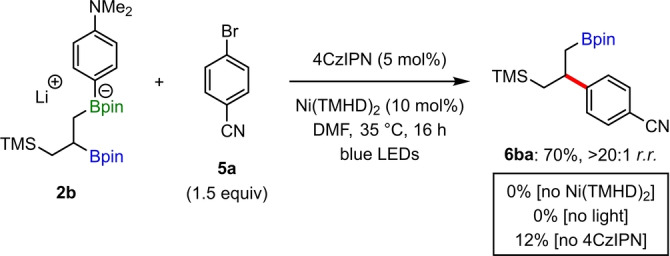
Investigation into EDA‐mediated pathway.

We next proceeded to further probe the mechanism of this nickel‐catalyzed site‐selective cross‐coupling. As shown in Table 3, the yields of the reactions were found to be dependent on the identity of the aryllithium used for boronate complex formation, with higher yields obtained with [4‐(dimethylamino)phenyl]lithium compared to phenyllithium. We hypothesize that this effect is due to the electron‐donating dimethylamino group providing a more electron‐rich arylboronate complex, which facilitates deboronative alkyl radical generation by lowering the oxidation potential of **2**, and through advantageous electron donor‐acceptor (EDA) complex formation with electron‐deficient aryl bromide **5 a**.[^12^, ^23^] To investigate this, a series of control reactions were carried out for the synthesis of **6 ba** from boronate complex **2 b** (Scheme 1). Whilst no product was obtained in reactions performed without the nickel catalyst or light, in contrast to reactions that utilized phenyllithium for boronate complex formation (see entry 14, Table 1), product **6 ba** could be obtained in 12 % yield in the absence of photocatalyst when using [4‐(dimethylamino)phenyl]lithium. This is consistent with a pathway that involves photoinduced electron transfer of an EDA complex between boronate complex **2 b** and aryl bromide **5 a**.

To provide a greater understanding of the factors that influence regioselectivity, we studied the reaction of 1‐pentene‐derived bis‐boronic ester **1 e** with aryl bromide **5 a** under various conditions (Table 4). The choice of alkyllithium used to generate boronate complex **2** from **1 a** had a significant impact on the yield of **6 ea**, but the regioselectivity was largely unaffected (entries 1–3). On the other hand, changing solvent from DMF to acetonitrile resulted in a dramatic decrease in selectivity, with only a slight preference for the formation of secondary coupled product **6 ea** over the primary coupled product **6 ea′** (entries 4 and 5). Finally, changing the nickel catalyst from Ni(THMD)_2_ to Ni(bpy)Br_2_ led to a low‐yielding and completely unselective reaction (entry 6). These results highlight the importance of both solvent and the ligand on nickel on the regioselectivity of this 1,2‐boron shift‐mediated cross‐coupling reaction.^[24]^

**Table 4:** Regioselectivity investigation.

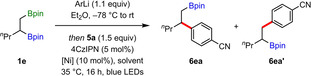					
Entry	ArLi	[Ni]	Solvent	Yield of **6 ea**	**6 ea : 6 ea′**
1	4‐(Me_2_N)C_6_H_4_Li	Ni(TMHD)_2_	DMF	51 %	4.0 : 1
2	PhLi	Ni(TMHD)_2_	DMF	26 %	3.0 : 1
3	MeLi	Ni(TMHD)_2_	DMF	35 %	3.7 : 1
4	PhLi	Ni(TMHD)_2_	CH_3_CN	22 %	1.3 : 1
5	MeLi	Ni(TMHD)_2_	CH_3_CN	39 %	1.3 : 1
6	PhLi	Ni(bpy)Br_2_	DMF	17 %	1.0 : 1

**Scheme 2:**
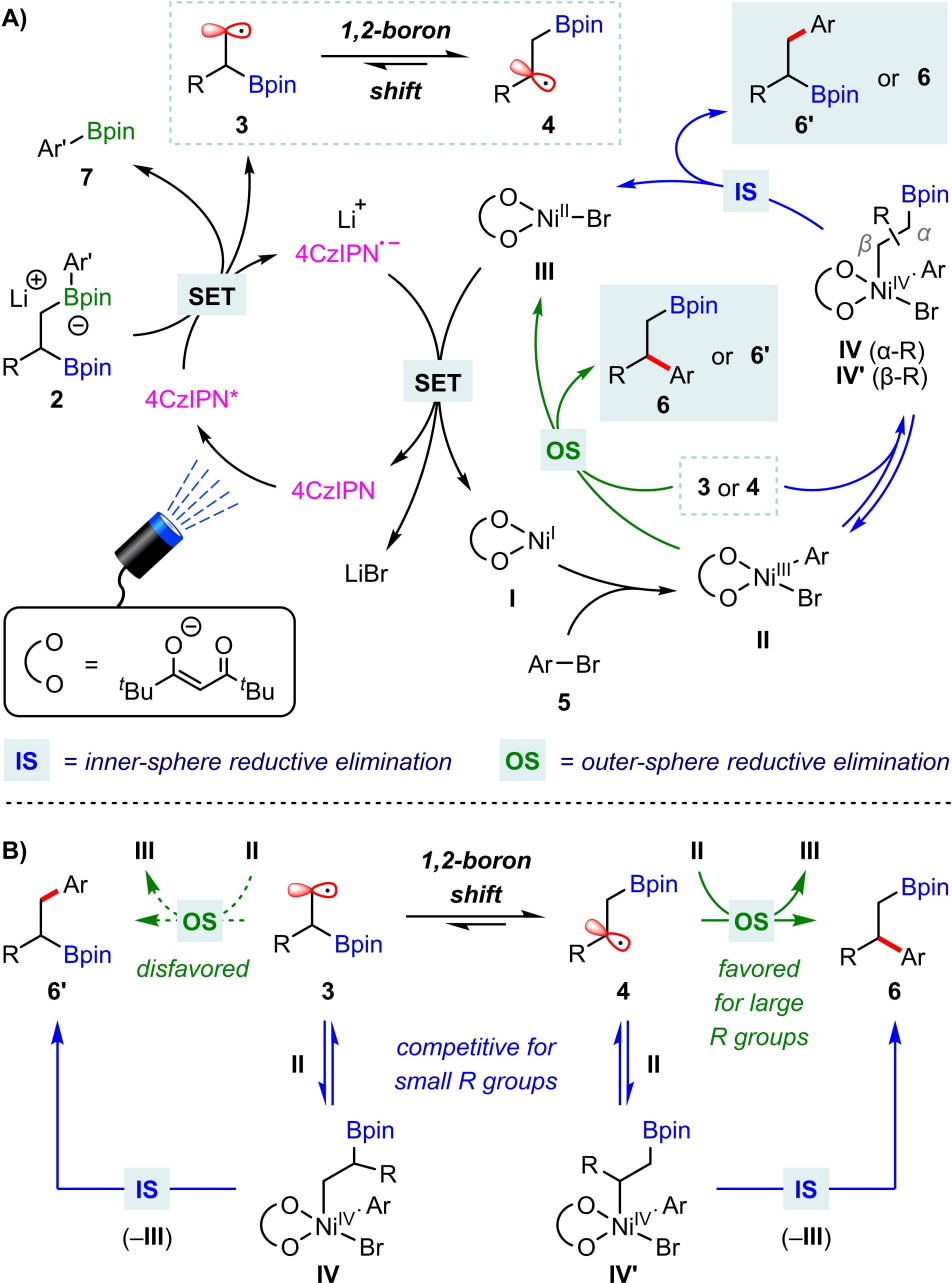
Proposed mechanism.

Based on the above observations and previous reports,[^19^, ^20^] we propose the following mechanism for the regioselective cross‐coupling of 1,2‐bis‐boronic esters (Scheme 2A). Photoexcitation of 4CzIPN (giving 4CzIPN*), followed by single electron oxidation of boronate complex **2** promotes C(sp^3^)−B bond cleavage to generate primary β‐boryl radical **3** and aryl boronic ester **7**. Radical **3** undergoes rapid 1,2‐boron shift to form the thermodynamically favored secondary β‐boryl radical **4**. Concurrently, oxidative addition of aryl bromide **5** to the diketonate‐ligated nickel(I) catalyst **I** yields the nickel(III) aryl bromide **II**. The reaction of **II** with β‐boryl radicals **3** or **4** to give arylated products **6′** or **6**, respectively, can proceed via two pathways: 1) outer‐sphere reductive elimination, wherein C(sp^3^)−C(sp^2^) bond formation occurs without addition of the radical to the nickel center; or 2) inner‐sphere reductive elimination, proceeding via nickel(IV) complex **IV** or **IV′**. Single electron transfer between the resulting nickel(II) bromide **III** and the reduced state of the photocatalyst (4CzIPN^.−^) regenerates **I** and ground state 4CzIPN. Considering that the 1,2‐boron shift is reversible,^[10]^ the regioselectivity of the reaction is likely determined during the reductive elimination step. A recent computational study of nickel/photoredox‐catalyzed Suzuki–Miyaura cross‐couplings of alkyl trifluoroborates by Molander and Gutierrez concluded that the steric hindrance of the alkyl radical intermediates strongly influences the reaction pathway.^[20]^ For the outer‐sphere reductive elimination pathway, a significantly lower energy barrier was calculated for tertiary alkyl radicals than for secondary alkyl radicals. In addition, for sterically hindered radicals, the formation of nickel(IV) alkyl‐aryl complexes by radical addition to **II** was endergonic and there was a higher energy barrier for the subsequent C(sp^3^)−C(sp^2^) formation, thus disfavoring the inner‐sphere reductive elimination pathway. By analogy, in the arylation of equilibrating β‐boryl radicals **3** and **4**, an outer‐sphere reductive elimination should favor arylation of secondary radical **4** over primary radical **3**, whereas an inner‐sphere pathway should favour arylation of the less sterically hindered primary radical **3** (Scheme 2B). Therefore, the high regioselectivity observed for sterically hindered substrates (e.g., R=CH_2_^*t*^Bu) could result from a sterically disfavoured inner‐sphere pathway, which means that the outer‐sphere reductive elimination of **4** dominates. For less hindered substrates (e.g., R=^*n*^Pr), the inner‐sphere pathway via radical **3** becomes competitive, resulting in lower regioselectivity. The reduction in regioselectivity observed when the nickel(I)‐diketonate catalyst is replaced with a nickel(0)‐bipyridine catalyst is a result of inner‐sphere reductive elimination (from a nickel(III) alkyl‐aryl complex) becoming the energetically favored pathway with neutral bipyridine ligands.[^20^, ^25^] In contrast to the outer‐sphere pathway, there appears to be only a small difference between the energy barriers for inner‐sphere reductive elimination of primary and secondary β‐boryl radicals **3** and **4**.

**Scheme 3:**
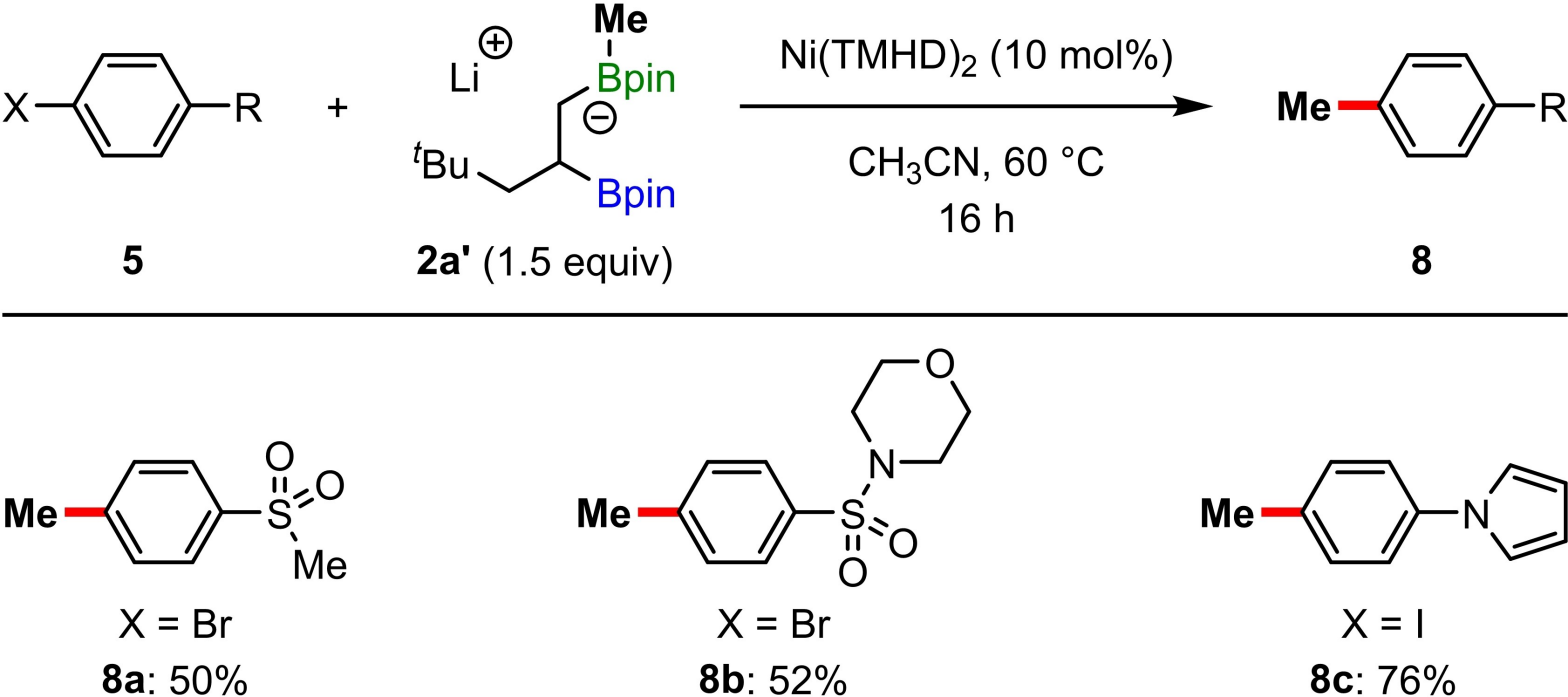
Ni‐catalyzed methylations with methyl boronate complexes.

During the initial development of this regioselective mono‐deboronative cross‐coupling, our plan was to utilize methyllithium for boronate complex formation because the volatile MeBpin by‐product could be easily removed during purification. While these reactions were successful (see entry 12, Table 1), significantly lower yields were obtained compared to those performed with aryllithium activators. Interestingly, through careful analysis of the crude reaction mixtures, we identified significant amounts methylbenzene products **8**, formed by direct methylation of the aryl halide. Further control experiments indicated that photocatalyst and light were not needed for this process, and that the yield could be improved by increasing the temperature of the reaction to 60 °C. Under these conditions, various aryl bromides (**8 a** and **8 b**) and iodides (**8 c**) were methylated in moderate to good yields using boronate complex **2 a′** as the methylating agent, and no β‐aryl boronic ester products **6** were observed (Scheme 3). Methylations are some of the most important reactions in synthetic chemistry, particularly in medicinal chemistry, where the incorporation of methyl groups into drug candidates can lead to enhanced pharmacological properties.^[26]^ Although, methylation of aryl halides can be achieved with a wide variety of methylating agents,^[27]^ the use methyl boronate complexes derived from readily available methyllithium and pinacol boronic esters under mild nickel‐catalyzed conditions provides a potentially useful alternative to more established protocols.^[28]^

**Scheme 4:**
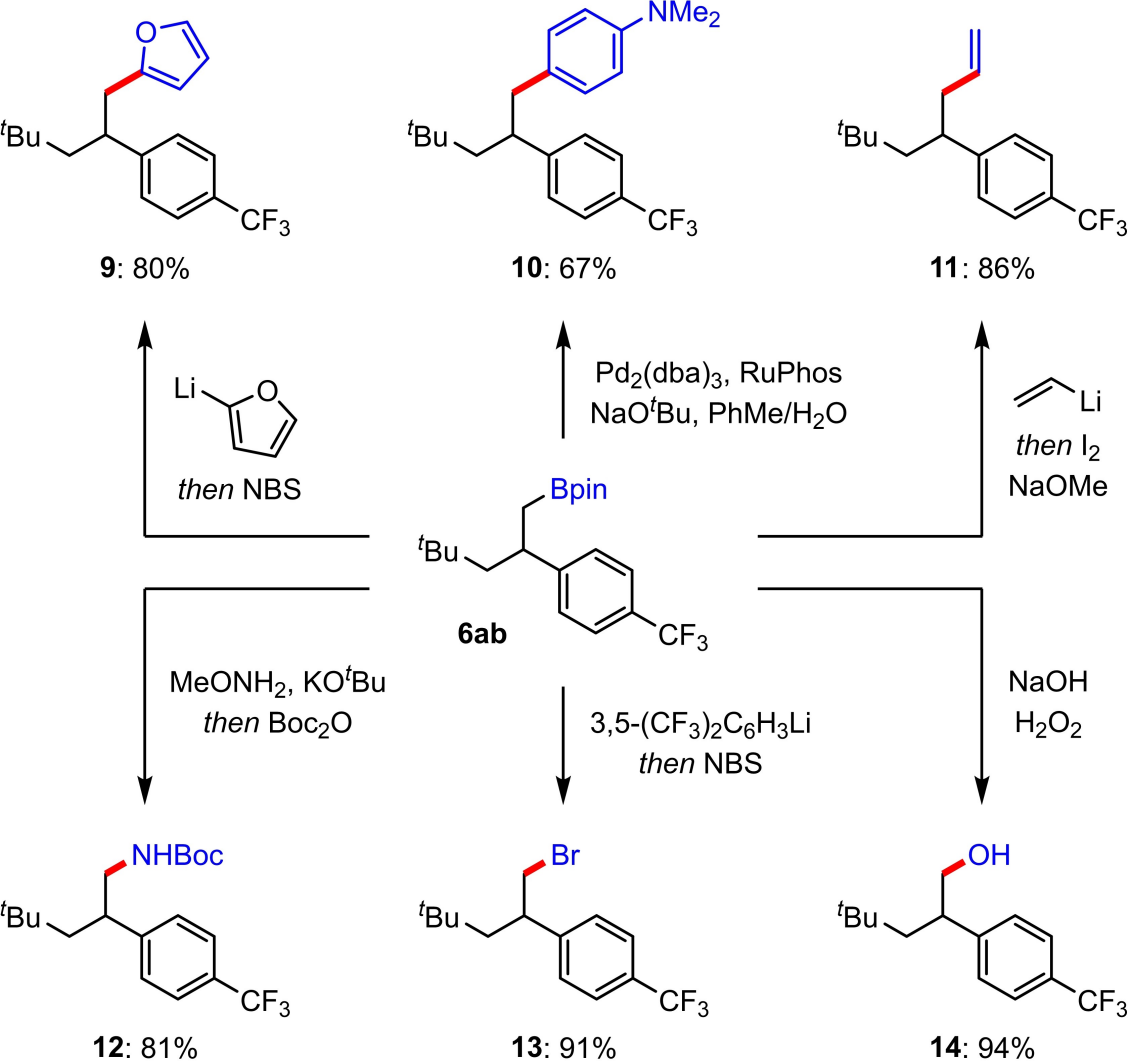
Boronic ester derivatization reactions.

Finally, we sought to demonstrate the synthetic utility of the primary β‐aryl boronic ester products through a series of derivatizations of the boronic ester group of **6 ab** (Scheme 4). Arylation was successfully achieved using both transition metal‐free and palladium‐catalyzed coupling reactions, affording good yields of furan **9** and 1,2‐diaryl ethane **10**, respectively.[^29^, ^30^] A further C(sp^3^)−C(sp^2^) bond formation was achieved through Zweifel olefination, which provided terminal alkene **11** in excellent yield.^[31]^ Using a recently developed amination procedure by Morken,^[32]^ homobenzylic amine **12** was obtained in 81 % yield. Conversion to bromide **13** was possible by electrophilic substitution of the boronate complex formed from **6 ab** and 3,5‐bis(trifluoromethyl)phenyllithium with *N*‐bromosuccinimide.^[33]^ Lastly, alcohol **14** was formed in 94 % yield by oxidation with H_2_O_2_/NaOH.

## Conclusion

In conclusion, we have developed a dual nickel/photoredox‐catalyzed site‐selective cross‐coupling reaction of 1,2‐bis‐boronic esters with aryl halides. This reaction provides access to secondary‐coupled products with high regioselectivity via 1,2‐boron shifts, highlighting its complementary selectivity to Pd‐catalyzed cross‐coupling reactions. Moreover, application of this method to cross‐couplings of readily prepared cyclic *cis*‐1,2‐bis‐boronic esters provides *trans*‐arylated products through a stereodivergent process. In addition, we have shown that the regioselectivity of the products was highly dependent on solvent, the type of ligand on the nickel catalyst, and the steric influence of the substituents on the 1,2‐bis‐boronic ester, suggesting that the mechanism of reductive elimination during C(sp^3^)−C(sp^2^) bond formation (inner‐sphere vs. outer‐sphere) is an important contributing factor to the high regioselectivity.

## Conflict of interest

The authors declare no conflict of interest.

1

## Supporting information

As a service to our authors and readers, this journal provides supporting information supplied by the authors. Such materials are peer reviewed and may be re‐organized for online delivery, but are not copy‐edited or typeset. Technical support issues arising from supporting information (other than missing files) should be addressed to the authors.

Supporting Information

## Data Availability

The data that support the findings of this study are available in the supplementary material of this article.
