# Supplementary material for: Dual Nickel/Photoredox‐Catalyzed Site‐Selective Cross‐Coupling of 1,2‐Bis‐Boronic Esters Enabled by 1,2‐Boron Shifts
Source: Angew Chem Int Ed Engl. 2022 Jul 14;61(34):e202207988. doi: 10.1002/anie.202207988 (PMC9543306; doi:10.1002/anie.202207988)
Supplement: Supplementary file 1 — Supporting Information [file ANIE-61-0-s001.pdf]

Angewandte Chemie
Eine Zeitschrift der Gesellschaft Deutscher Chemiker

Supporting Information

# Dual Nickel/Photoredox-Catalyzed Site-Selective Cross-Coupling of 1,2-Bis-Boronic Esters Enabled by 1,2-Boron Shifts

*H. Wang, W. Han, A. Noble, V. K. Aggarwal**

# TABLE OF CONTENTS

LIST OF SUPPLEMENTARY SCHEMES, FIGURES AND TABLES ........ S2

LIST OF CHARACTERISED PRODUCTS ........ S2

1. MATERIALS AND GENERAL METHODS ........ S4

1.1. Glassware, Solvents, and Reagents ........ S4

1.2. Chromatography and Instrumentation ........ S4

1.3. Naming of Compounds ........ S4

2. EXPERIMENTAL DATA ........ S5

2.1. Photochemical Equipment and Setup ........ S5

2.2. Reaction Optimization ........ S6

2.2.1. Optimization Studies and Control Reactions ........ S6

2.3. General Procedures ........ S7

2.3.1. General Procedure A: Diboration of Terminal Alkenes ........ S7

2.3.2. General Procedure B: For Reactions of Aryl Halides **5a-5ac** with **1a**, **1c**, **1f** and **1k** ........ S7

2.3.3. General Procedure C: For Reactions of Aryl Halide **5a** with **1b**, **1d-1e** and **1g-1j** ........ S8

2.4. Synthesis and Purchasing of Starting Materials ........ S10

2.5. Characterization Data ........ S11

3. MECHANISTIC STUDIES ........ S46

3.1. Radical Clock Experiment ........ S46

3.2. 2.0 mmol-Scale Reaction ........ S47

3.3. Pd-catalyzed Cross-Coupling Reaction of Cyclic Bis-boronic Ester ........ S48

3.4. Regioselectivity Studies for the Reaction of **1e** ........ S49

3.4.1. Regioselectivity of the Reaction of Pinacol Bis-Boronic Ester **1e** ........ S49

3.4.2. Regioselectivity of the Reaction of Neopentyl Glycol Bis-Boronic Ester **1e′** ........ S50

3.5. Ni-Catalyzed Methylation of Aryl Halides ........ S51

3.6. Transformations of **6ab** ........ S53

3.6.1. Synthesis of Compound **9** ........ S53

3.6.2. Synthesis of Compound **10** ........ S54

3.6.3. Synthesis of Compound **11** ........ S55

3.6.4. Synthesis of Compound **12** ........ S56

3.6.5. Synthesis of Compound **13** ........ S57

3.6.6. Synthesis of Compound **14** ........ S58

4. SPECTROSCOPIC DATA ........ S60

5. REFERENCES ........ S180

# LIST OF SUPPLEMENTARY SCHEMES, FIGURES AND TABLES

Figure S1. Photochemical Reaction Setup ........ S5
Table S1: Optimization Studies for the Selective Cross-Coupling Reaction ........ S6
Figure S2. Initial Reaction Setup 1 ........ S8
Figure S3. Initial Reaction Setup 2 ........ S9
Scheme S1. Previously Reported Boronic Ester Substrates ........ S10
Figure S4. The GC-Fid Result for the Regioselectivity of **6ca** ........ S37
Figure S5. Crude $^{1}$H NMR Result of **6da** with PhLi as Lithium Reagent ........ S38
Figure S6. Crude $^{1}$H NMR Result of **6da** with 4-$Me_2NC_6H_4Li$ as Lithium Reagent ........ S39
Figure S7. 2.0 mmol-Scale Reaction Setup ........ S47
Table S2: Studies for the Regioselectivity of the Reaction with **1e** ........ S49
Figure S8. GC-Fid Results for the Regioselectivity of Reaction with **1e** ........ S49
Figure S9. GC-Fid Results for the Regioselectivity of Reaction with **1e′** ........ S50

# LIST OF CHARACTERISED PRODUCTS

[2,3-Bis(4,4,5,5-tetramethyl-1,3,2-dioxaborolan-2-yl)propyl]trimethylsilane (**1b**) ........ S11
2,2'-(4-Methylpentane-1,2-diyl)bis(4,4,5,5-tetramethyl-1,3,2-dioxaborolane) (**1d**) ........ S11
2,2'-(Pentane-1,2-diyl)bis(4,4,5,5-tetramethyl-1,3,2-dioxaborolane) (**1e**) ........ S12
2,2'-(Pentane-1,2-diyl)bis(4,4,5,5-tetramethyl-1,3,2-dioxaborolane) (**1e′**) ........ S12
(*cis*)-1,2-Bis(4,4,5,5-tetramethyl-1,3,2-dioxaborolan-2-yl)cyclopentane (**1g**) ........ S13
(*cis*)-1,2-Bis(4,4,5,5-tetramethyl-1,3,2-dioxaborolan-2-yl)cyclohexane (**1h**) ........ S14
(1*R*,2*S*,3*R*,4*S*)-2,3-Bis(4,4,5,5-tetramethyl-1,3,2-dioxaborolan-2-yl)bicyclo[2.2.1]heptane (**1i**) ........ S14
2,2'-(1-Cyclopropylethane-1,2-diyl)bis(4,4,5,5-tetramethyl-1,3,2-dioxaborolane) (**1l**) ........ S15
4-[4,4-Dimethyl-1-(4,4,5,5-tetramethyl-1,3,2-dioxaborolan-2-yl)pentan-2-yl]benzonitrile (**6aa**) ........ S15
2-{4,4-Dimethyl-2-[4-(trifluoromethyl)phenyl]pentyl}-4,4,5,5-tetramethyl-1,3,2-dioxaborolane (**6ab**) ........ S16
1-{4-[4,4-Dimethyl-1-(4,4,5,5-tetramethyl-1,3,2-dioxaborolan-2-yl)pentan-2-yl]phenyl}ethan-1-one (**6ac**) ........ S17
Ethyl 4-[4,4-dimethyl-1-(4,4,5,5-tetramethyl-1,3,2-dioxaborolan-2-yl)pentan-2-yl]benzoate (**6ad**) ........ S17
5-[4,4-Dimethyl-1-(4,4,5,5-tetramethyl-1,3,2-dioxaborolan-2-yl)pentan-2-yl]isobenzofuran-1(3*H*)-one (**6ae**) S18
4-[4,4-Dimethyl-1-(4,4,5,5-tetramethyl-1,3,2-dioxaborolan-2-yl)pentan-2-yl]-2-fluorobenzonitrile (**6af**) ........ S19
4-(1-Hydroxy-4,4-dimethylpentan-2-yl)-*N*,*N*-dimethylbenzamide (**6ag**) ........ S20
2-{2-[(1,1'-Biphenyl)-4-yl]-4,4-dimethylpentyl}-4,4,5,5-tetramethyl-1,3,2-dioxaborolane (**6ah**) ........ S20
2-[4,4-Dimethyl-2-(naphthalen-2-yl)pentyl]-4,4,5,5-tetramethyl-1,3,2-dioxaborolane (**6ai**) ........ S21
2-(4-Fluorophenyl)-4,4-dimethylpentan-1-ol (**6aj**) ........ S22
2-(4-Chlorophenyl)-4,4-dimethylpentan-1-ol (**6ak**) ........ S22
4,4-Dimethyl-2-(*p*-tolyl)pentan-1-ol (**6al**) ........ S23
2-[2-(4-Methoxyphenyl)-4,4-dimethylpentyl]-4,4,5,5-tetramethyl-1,3,2-dioxaborolane (**6am**) ........ S24
2-{4,4-Dimethyl-2-[4-(methylthio)phenyl]pentyl}-4,4,5,5-tetramethyl-1,3,2-dioxaborolane (**6an**) ........ S24
1-{4-[4,4-Dimethyl-1-(4,4,5,5-tetramethyl-1,3,2-dioxaborolan-2-yl)pentan-2-yl]phenyl}-1*H*-pyrrole (**6ao**) ........ S25
3-[4,4-Dimethyl-1-(4,4,5,5-tetramethyl-1,3,2-dioxaborolan-2-yl)pentan-2-yl]benzonitrile (**6ap**) ........ S26
4,4-Dimethyl-2-[3-(trifluoromethyl)phenyl]pentan-1-ol (**6aq**) ........ S26
2-{2-[3,5-Bis(trifluoromethyl)phenyl]-4,4-dimethylpentyl}-4,4,5,5-tetramethyl-1,3,2-dioxaborolane (**6ar**) ........ S27
1-{3-[4,4-Dimethyl-1-(4,4,5,5-tetramethyl-1,3,2-dioxaborolan-2-yl)pentan-2-yl]phenyl}ethan-1-one (**6as**) ........ S28
Methyl 3-[4,4-dimethyl-1-(4,4,5,5-tetramethyl-1,3,2-dioxaborolan-2-yl)pentan-2-yl]benzoate (**6at**) ........ S28

2-(3-Methoxyphenyl)-4,4-dimethylpentan-1-ol (**6au**) ........ S29
4-[4,4-Dimethyl-1-(4,4,5,5-tetramethyl-1,3,2-dioxaborolan-2-yl)pentan-2-yl]-3-methylbenzonitrile (**6av**) ........ S30
2-[4,4-Dimethyl-1-(4,4,5,5-tetramethyl-1,3,2-dioxaborolan-2-yl)pentan-2-yl]benzonitrile (**6aw**) ........ S30
5-[4,4-Dimethyl-1-(4,4,5,5-tetramethyl-1,3,2-dioxaborolan-2-yl)pentan-2-yl]-2-methylpyridine (**6ax**) ........ S31
5-[4,4-Dimethyl-1-(4,4,5,5-tetramethyl-1,3,2-dioxaborolan-2-yl)pentan-2-yl]-2-(trifluoromethyl)pyridine (**6ay**) ........ S32
5-[4,4-Dimethyl-1-(4,4,5,5-tetramethyl-1,3,2-dioxaborolan-2-yl)pentan-2-yl]-2-methoxypyrimidine (**6az**) ........ S32
5-[4,4-Dimethyl-1-(4,4,5,5-tetramethyl-1,3,2-dioxaborolan-2-yl)pentan-2-yl]-2-methylpyrimidine (**6aaa**) ........ S33
2-(2,3-Dihydrobenzofuran-5-yl)-4,4-dimethylpentan-1-ol (**6aab**) ........ S34
5-[4,4-Dimethyl-1-(4,4,5,5-tetramethyl-1,3,2-dioxaborolan-2-yl)pentan-2-yl]-2-methylbenzo[*d*]thiazole (**6aac**) ........ S34
(*Z*)-4,4,5,5-Tetramethyl-2-(2-neopentylpent-3-en-1-yl)-1,3,2-dioxaborolane (**6aad**) ........ S35
4-[1-(4,4,5,5-Tetramethyl-1,3,2-dioxaborolan-2-yl)-3-(trimethylsilyl)propan-2-yl]benzonitrile (**6ba**) ........ S36
4-[1-Cyclohexyl-2-(4,4,5,5-tetramethyl-1,3,2-dioxaborolan-2-yl)ethyl]benzonitrile (**6ca**) ........ S37
4-(1-Hydroxy-4-methylpentan-2-yl)benzonitrile (**6da**) ........ S38
4-(1-Hydroxypentan-2-yl)benzonitrile (**6ea**) ........ S40
4-(2-Hydroxypentyl)benzonitrile (**6ea′**) ........ S40
4-[1-(Hydroxymethyl)cyclohexyl]benzonitrile (**6fa**) ........ S41
(*trans*)-4-[2-(4,4,5,5-Tetramethyl-1,3,2-dioxaborolan-2-yl)cyclopentyl]benzonitrile (**(*trans*)-6ga**) ........ S41
(*trans*)-4,4,5,5-Tetramethyl-2-(2-phenylcyclohexyl)-1,3,2-dioxaborolane (**(*trans*)-6ha**) ........ S42
4-(3-Hydroxybicyclo[2.2.1]heptan-2-yl)benzonitrile (**6ia**) ........ S43
4-[(1*R*,6ar,4*R*,6a*S*)-4-Hydroxyoctahydropentalen-1-yl)benzonitrile (**6ja**) ........ S44
4-{2-[(1*R*,5*S*)-5-Hydroxy-4-methylcyclohex-3-en-1-yl]propan-2-yl}benzonitrile (**6ka**) ........ S44
(*E/Z*)-4-(5-Hydroxypent-3-en-1-yl)benzonitrile (**6la**) ........ S46
4-[(*cis*)-2-(4,4,5,5-Tetramethyl-1,3,2-dioxaborolan-2-yl)cyclopentyl]benzonitrile (**(*cis*)-6ga**) ........ S48
1-Methyl-4-(methylsulfonyl)benzene (**8a**) ........ S51
4-Tosylmorpholine (**8b**) ........ S52
1-(*p*-Tolyl)-1*H*-pyrrole (**8c**) ........ S52
2-{4,4-Dimethyl-2-[4-(trifluoromethyl)phenyl]pentyl}furan (**9**) ........ S53
4-{4,4-Dimethyl-2-[4-(trifluoromethyl)phenyl]pentyl}-*N,N*-dimethylaniline (**10**) ........ S54
1-(6,6-Dimethylhept-1-en-4-yl)-4-(trifluoromethyl)benzene (**11**) ........ S55
*tert*-Butyl {4,4-dimethyl-2-[4-(trifluoromethyl)phenyl]pentyl}carbamate (**12**) ........ S56
1-(1-Bromo-4,4-dimethylpentan-2-yl)-4-(trifluoromethyl)benzene (**13**) ........ S57
4,4-Dimethyl-2-[4-(trifluoromethyl)phenyl]pentan-1-ol (**14**) ........ S58

# 1. MATERIALS AND GENERAL METHODS

## 1.1. Glassware, Solvents, and Reagents

All manipulations were performed with oven-dried (130 °C for a minimum of 12 h) or flame-dried glassware using standard Schlenk techniques under an atmosphere of nitrogen, unless otherwise stated.

All anhydrous solvents were commercially supplied or dried using an Anhydrous Engineering alumina column drying system ($CH_3CN$, DMF, DMA, toluene, NMP, and THF). Reagents were purchased from commercial sources and used as received. All organolithium reagents were titrated against *N*-benzylbenzamide.[1]

## 1.2. Chromatography and Instrumentation

**Thin layer chromatography** (TLC) was performed using Merck Kieselgel 60 F254 fluorescent treated silica, which was visualised under UV light, or by staining with aqueous basic potassium permanganate followed by heating, or Hanessian's stain (CAM stain) followed by heating, or *p*-anisaldehyde solution followed by heating, as stated.

**Flash column chromatography** (FCC) was carried out using Sigma-Aldrich silica gel (60 Å, 230-400 mesh, 40-63 μm) or boric acid impregnated silica gel.[2] In cases where automated column chromatography was employed the solvent gradient and flow rate are indicated.

**NMR spectra** were recorded at various field strengths, as indicated, using Bruker 400 MHz, Varian VNMR 400 MHz for $^1$H, $^{11}$B, $^{13}$C and $^{19}$F acquisitions. All NMR spectra were recorder at 25 °C unless otherwise stated. Chemical shifts (*δ*) are reported in parts per million (ppm) and referenced to $CDCl_3$ ($^1$H: 7.26 ppm; $^{13}$C: 77.16 ppm). Coupling constants (*J*) are given in Hertz (Hz) and refer to apparent multiplicities (s = singlet, d = doublet, t = triplet, q = quartet, quin = quintet, hex = hextet, h = heptet, m = multiplet, brs = broad signal, dd = doublet of doublets, etc.). The $^1$H NMR spectra are reported as follows: chemical shift (multiplicity, coupling constants, number of protons).

**High resolution mass spectra (HRMS)** were recorded on a Bruker Daltonics MicrOTOF II by Electrospray Ionisation (ESI); a Thermo Scientific QExactive by Electron Ionisation (EI); a Thermo Scientific Orbitrap Elite by ESI or Atmospheric Pressure Chemical Ionisation (APCI); or a Bruker UltrafleXtreme by Matrix-assisted Laser Desorption/Ionisation (MALDI).

**IR spectra** were recorded neat as a thin film on a Perkin Elmer Spectrum One FT-IR. Selected absorption maxima ($\nu_{max}$) are reported in wavenumbers (cm$^{-1}$).

**Gas chromatography–mass spectrometry** (**GC-MS**) was recorded on an Agilent 6890 Series GC and 5973 detector using a HP-5MS UI column (15 m x 0.25 mm x 0.25 μm).

## 1.3. Naming of Compounds

Compound names are those generated by ChemDraw Professional 20.0 software (PerkinElmer), following the IUPAC nomenclature.

# 2. EXPERIMENTAL DATA

## 2.1. Photochemical Equipment and Setup

The photochemical reactions were carried out in the photochemical reactor (PhotoCube™, manufactured by ThalesNano) which the LED panels (blue light, $\lambda_{max}$ = 457 nm) are on the 4 sides (the intensity knob is set to 70% and the current selector is set to Hi mode) (Figure S1).

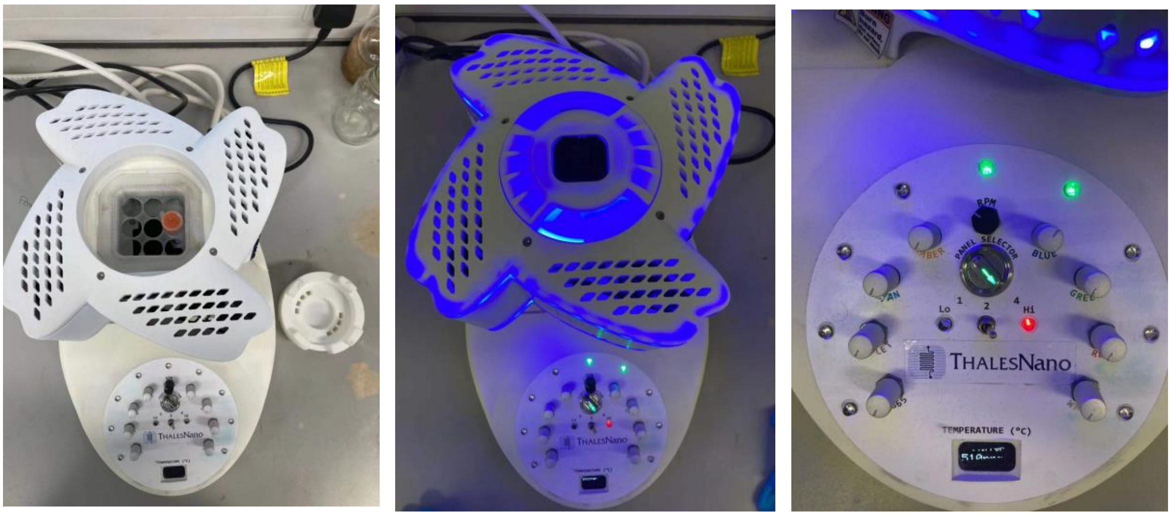


**Figure S1. Photochemical Reaction Setup**

## 2.2. Reaction Optimization

### 2.2.1. Optimization Studies and Control Reactions

**Table S1: Optimization Studies for the Selective Cross-Coupling Reaction**

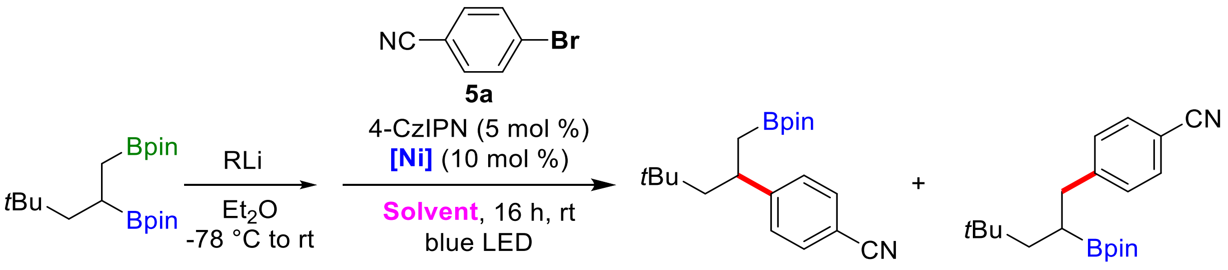


| Entry[a] | RLi | [Ni] | Solvent | Yield (6aa)[b] | *r.r.* (6aa/6aa')[c] |
|---|---|---|---|---|---|
| 1 | PhLi | $Ni(TMHD)_2$ | $CH_3CN$ | 45% | 7.8/1.0 |
| 2 | PhLi | $Ni(TMHD)_2$ | DMF | 92% (95%)[d] | 16.4/1.0 |
| 3 | PhLi | $Ni(TMHD)_2$ | DMA | 84% | 15.9/1.0 |
| 4 | PhLi | $Ni(TMHD)_2$ | Toluene | 11% | 12.7/1.0 |
| 5 | PhLi | $Ni(TMHD)_2$ | THF | 34% | >20/1.0 |
| 6 | PhLi | $Ni(TMHD)_2$ | NMP | 56% | 20/1.0 |
| 7 | PhLi | $Ni(acac)_2$ | DMF | 81% | >20/1.0 |
| 8 | PhLi | $Ni(bpy)Br_2$ | DMF | 72% | 3.2/1.0 |
| 9 | PhLi | $NiCl_2$·glyme/dtbpy | DMF | 40% | 3.1/1.0 |
| 10 | PhLi | $Ni(COD)_2$/dtbpy | DMF | 14% | 1.1/1.0 |
| 11 | PhLi | $Ni(dppp)Cl_2$ | DMF | 0% | -- |
| 12[e] | PhLi | $Ni(TMHD)_2$ | DMF | 92% | 18/1.0 |
| **13[f]** | PhLi | **$Ni(TMHD)_2$** | **DMF** | **92% (81%)[g]** | **20/1.0** |
| 14[f] | MeLi | $Ni(TMHD)_2$ | DMF | 40% | 15/1.0 |
| 15[f] | PhLi | -- | DMF | 0% | -- |
| 16[f],[h] | PhLi | $Ni(TMHD)_2$ | DMF | 0% | -- |
| 17[f],[i] | PhLi | $Ni(TMHD)_2$ | DMF | 0% | -- |

[a] **1a** (0.2 mmol), PhLi (1.1 equiv.), **5a** (3.0 equiv.), 4-CzIPN (5.0 mol %), [Ni] (10.0 mol %), Solvent (2.0 mL) under $N_2$ in photochemical reactor (PhotoCube™) for 16 hours. [b] Yield was determined by GC-Fid analysis using 1,3,5-trimethoxybenzene as internal standard; [c] The regioselectivity was determined by GC-Fid analysis; [d] $^1$H NMR yield by using 1,3,5-trimethoxybenzene as internal standard; [e] **5a** (2.0 equiv.); [f] **5a** (1.5 equiv.); [g] Isolated yield; [h] Without photocatalyst; [i] Without light.

## 2.3. General Procedures

### 2.3.1. General Procedure A: Diboration of Terminal Alkenes

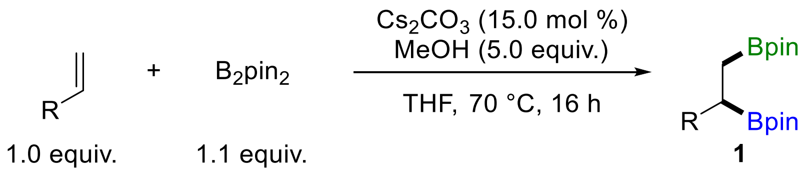

Following a modified literature procedure:[3]

To a flame-dried Schlenk flask were added $B_2pin_2$ (1.1 equiv.) and $Cs_2CO_3$ (15.0 mol %), after which the flask was evacuated and back-filled with nitrogen three times. Subsequently, anhydrous THF (0.25 M with respect to the alkene substrate), anhydrous methanol (5.0 equiv.) and the corresponding alkene (1.0 equiv.) were added sequentially *via* syringe. The resulting suspension was heated to 70 °C for 16 h, after which the reaction mixture was filtered through a plug of silica (eluting with diethyl ether). The filtrate was concentrated under reduced pressure and the resulting crude material was purified by flash column chromatography (pentane/diethyl ether) to give the bis-boronic ester product **1**.

### 2.3.2. General Procedure B: For Reactions of Aryl Halides 5a-5ac with 1a, 1c, 1f and 1k

In glovebox, to a 7.0 mL vial equipped with a magnetic stir bar was added the boronic ester (**1**) (0.20 mmol, 1.0 equiv.). The vial was sealed with a septum and removed from the glovebox, and then anhydrous diethyl ether (2.0 mL) was added under $N_2$. The solution was cooled to −78 °C (dry ice/acetone) and PhLi (1.9 M in dibutyl ether, 0.12 mL, 0.22 mmol, 1.1 equiv.) was added dropwise (**Figure S2**). The mixture was allowed to stir for 30 min at −78 °C before removing the cooling bath and warming to ambient temperature. After 30 min, the solvent was removed under high vacuum, and the vial with boronate complex was quickly put into the glovebox, and then the aryl halides **5** (0.30 mmol, 1.5 equiv.), 4-CzIPN (7.9 mg, 0.01 mmol, 5.0 mol %), $Ni(TMHD)_2$ (8.5 mg, 0.02 mmol, 10.0 mol %) and anhydrous DMF (2.0 mL) were added. The vial was tightly sealed, then removed from the glovebox and stirred under blue LED irradiation for 16 h (see **Figure S1** for experimental setup). After irradiation, the reaction mixture was transferred into a 25 mL vial, diluted with $Et_2O$ (5.0 mL) and washed with water (5.0 mL). The organic phase was separated, and the aqueous phase was extracted with diethyl ether (4*5.0 mL). The organic phases were combined and concentrated under reduced pressure. The crude material was purified by flash column chromatography (pentane/$Et_2O$ or *n*-hexane/EtOAc) to afford the coupled product (or oxidized to the corresponding alcohol according to the procedure reported below).

*Oxidation of Boronic Esters*: The crude material was dissolved in THF (3.0 mL) and cooled to 0 °C. An aqueous solution of sodium hydroxide (2.0 M, 1.0 mL) and aqueous hydrogen peroxide (30%, 1.0 mL) were added dropwise. After 10 min, the vigorously stirred biphasic reaction mixture was allowed to reach ambient temperature and stirred for another 30 min, at which point TLC analysis showed the disappearance of the boronic ester. The mixture was diluted with water (5.0 mL) and ethyl acetate (10 mL). The organic phase was separated, and the aqueous phase was extracted with ethyl acetate (3*10 mL). The combined organic extracts were dried over anhydrous sodium sulfate, filtered and concentrated under reduced pressure. The resulting

crude material was purified by flash column chromatography to afford the alcohol products.

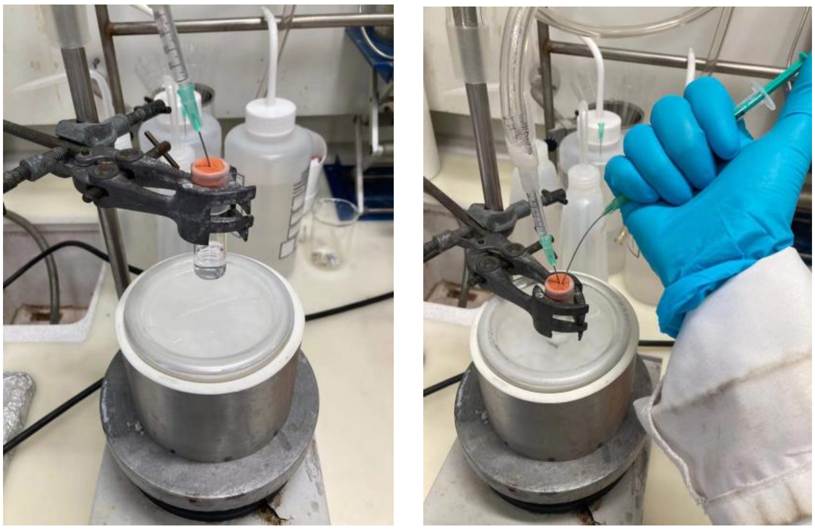

**Figure S2. Initial Reaction Setup 1**

### 2.3.3. General Procedure C: For Reactions of Aryl Halide 5a with 1b, 1d-1e and 1g-1j

A flame-dried nitrogen-flushed Schlenk tube (10 mL) was charged with 4-bromo-*N,N*-dimethylaniline (44.0 mg, 0.22 mmol, 1.1 equiv.) and THF (0.6 mL). The solution was cooled to –78 °C (dry ice/acetone) and then *t*-BuLi (1.7 M in pentane, 260 µL, 0.44 mmol, 3.2 equiv.) was added slowly dropwise. The mixture was stirred for 5 min before removing the cooling bath, then the solution was allowed to stir at 0 °C for 30 min (**Figure S3**).

In glovebox, to a 7 mL vial equipped with a magnetic stir bar was added the boronic ester (**1**) (0.20 mmol, 1.0 equiv.). The vial was sealed with a septum and removed from the glovebox, and then anhydrous diethyl ether (2.0 mL) was added under $N_2$. The solution was cooled to −78 °C (dry ice/acetone) and the pre-prepared [4-(dimethylamino)phenyl]lithium solution (from above) was added dropwise. The mixture was allowed to stir for 30 min at −78 °C before removing the cooling bath and warming to ambient temperature. After 30 min, the solvent was removed under high vacuum, and the vial with boronate complex was quickly put into the glovebox, and 4-bromobenzonitrile (**1a**) (54.6 mg, 0.30 mmol, 1.5 equiv.), 4-CzIPN (7.9 mg, 0.01 mmol, 5.0 mol %), $Ni(TMHD)_2$ (8.5 mg, 0.02 mmol, 10.0 mol %) and anhydrous DMF (2.0 mL) were added. The vial was tightly sealed, then removed from the glovebox and stirred under blue LED irradiation for 16 h. After irradiation, the reaction mixture was transferred to a 25 mL vial, diluted with $Et_2O$ (5.0 mL) and washed with 2.0 M aqueous HCl solution (5.0 mL). The organic phase was separated, and the aqueous phase was extracted with diethyl ether (4*5.0 mL). The organic phases were combined and concentrated under reduced pressure. The crude material was purified by flash column chromatography (pentane/diethyl ether or *n*-hexane/EtOAc) to afford the coupled product (or oxidized to the corresponding alcohol according to the procedure reported above).

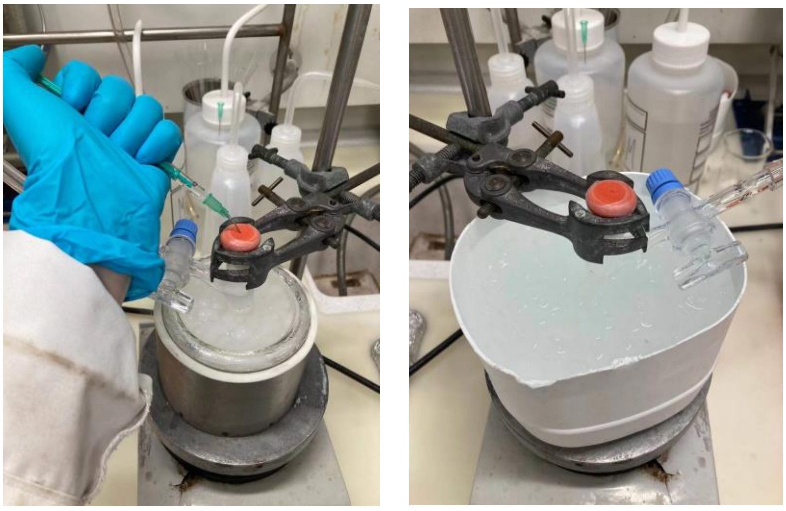

**Figure S3. Initial Reaction Setup 2**

## 2.4. Synthesis and Purchasing of Starting Materials

All aryl halides **5** were purchased from commercial sources and used as received.

The following boronic esters were synthesised according to the reports by our group:[4]

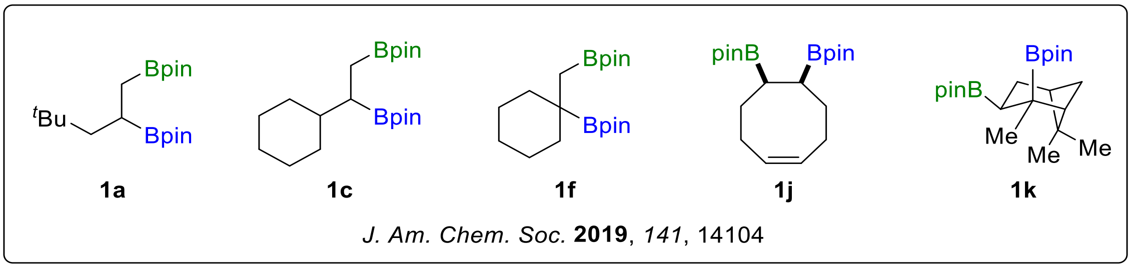


**Scheme S1. Previously Reported Boronic Ester Substrates**

## 2.5. Characterization Data

**[2,3-Bis(4,4,5,5-tetramethyl-1,3,2-dioxaborolan-2-yl)propyl]trimethylsilane (1b)**

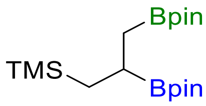


**1b**

Prepared following **General Procedure A** using allyltrimethylsilane (1.59 mL, 10.0 mmol, 1.0 equiv.), $B_2pin_2$ (2.79 g, 11.0 mmol, 1.1 equiv.), $Cs_2CO_3$ (0.53 g, 1.5 mmol, 15 mol %), anhydrous methanol (2.02 mL, 50.0 mmol, 5.0 equiv.) and anhydrous THF (50 mL). Purification by flash column chromatography (95:5 pentane/$Et_2O$) gave the title compound (2.84 g, 77%) as a colorless oil.

**TLC**: $R_f$ = 0.28 (95:5 *n*-hexane/EtOAc, CAM stain).

**NMR Spectroscopy (*see spectra*):**

$^1$**H NMR** (400 MHz, $CDCl_3$): $\delta_H$ 1.23 (s, 12H), 1.22 (s, 12H), 1.21 – 1.14 (m, 1H), 0.96 – 0.76 (m, 3H), 0.49 (dd, *J* = 14.6, 6.6 Hz, 1H), -0.02 (s, 9H) ppm;

$^{13}$**C NMR** (101 MHz, $CDCl_3$): $\delta_C$ 82.9, 82.9, 25.1, 25.0, 24.9, 24.9, 20.5, -0.6 ppm. The carbon attached to boron was not observed due to quadrupolar relaxation;

$^{11}$**B NMR** (128 MHz, $CDCl_3$): $\delta_B$ 33.00 ppm.

**IR** (film): $\nu_{max}$ 2978, 1740, 1369, 1311, 1245, 1215, 968, 835, 690 cm$^{-1}$.

All recorded spectroscopic data matched those previously reported in the literature.[5]

**2,2'-(4-Methylpentane-1,2-diyl)bis(4,4,5,5-tetramethyl-1,3,2-dioxaborolane) (1d)**

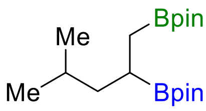


**1d**

Prepared following **General Procedure A** using 4-methylpent-1-ene (1.27 mL, 10.0 mmol, 1.0 equiv.), $B_2pin_2$ (2.79 g, 11.0 mmol, 1.1 equiv.), $Cs_2CO_3$ (0.53 g, 1.5 mmol, 15 mol %), anhydrous methanol (2.02 mL, 50.0 mmol, 5.0 equiv.) and anhydrous THF (50 mL). Purification by flash column chromatography (95:5 pentane/$Et_2O$) gave the title compound (2.5 g, 74%) as a colorless oil.

**TLC**: $R_f$ = 0.24 (95:5 *n*-hexane/EtOAc, CAM stain).

**NMR Spectroscopy (*see spectra*):**

$^1$**H NMR** (400 MHz, $CDCl_3$): $\delta_H$ 1.65 – 1.53 (m, 1H), 1.41 – 1.30 (m, 1H), 1.23 (s, 12H), 1.22 (s, 12H), 1.21 – 1.11 (m, 2H), 0.86 (d, *J* = 6.6 Hz, 3H), 0.84 (d, *J* = 6.6 Hz, 3H), 0.82 – 0.79 (m, 2H) ppm;

$^{13}$**C NMR** (101 MHz, $CDCl_3$): $\delta_C$ 83.0, 82.9, 43.1, 26.9, 25.1, 25.0, 24.9, 24.9, 23.0, 22.8 ppm. The carbon

attached to boron was not observed due to quadrupolar relaxation;

**$^{11}$B NMR** (128 MHz, $CDCl_3$): $\delta_B$ 34.00 ppm.

**IR** (film): $\nu_{max}$ 2977, 2953, 1467, 1369, 1311, 1247, 1212, 1141, 968, 848, 672 $cm^{-1}$.

**HRMS** ($APCI^+$): m/z calculated for $C_{18}H_{37}B_2O_4$ $[M+H]^+$, 339.2872; found, 339.2879.

### 2,2'-(Pentane-1,2-diyl)bis(4,4,5,5-tetramethyl-1,3,2-dioxaborolane) (1e)

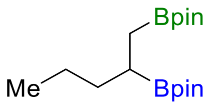


**1e**

Prepared following **General Procedure A** using pent-1-ene (1.09 mL, 10.0 mmol, 1.0 equiv.), $B_2pin_2$ (2.79 g, 11.0 mmol, 1.10 equiv.), $Cs_2CO_3$ (0.53 g, 1.5 mmol, 15 mol %), anhydrous methanol (2.02 mL, 50.0 mmol, 5.0 equiv.) and anhydrous THF (50 mL). Purification by flash column chromatography (95:5 pentane/$Et_2O$) gave the title compound (2.3 g, 71%) as a colorless oil.

**TLC**: $R_f$ = 0.24 (95:5 *n*-hexane/EtOAc, CAM stain).

**NMR Spectroscopy (*see spectra*):**

**$^1$H NMR** (400 MHz, $CDCl_3$): $\delta_H$ 1.47 – 1.39 (m, 1H), 1.35 – 1.26 (m, 3H), 1.22 (s, 12H), 1.22 (s, 12H), 1.16 – 1.07 (m, 1H), 0.91 – 0.75 (m, 5H) ppm;

**$^{13}$C NMR** (101 MHz, $CDCl_3$): $\delta_C$ 82.9, 82.9, 36.3, 25.0, 25.0, 24.9, 24.9, 22.1, 14.5 ppm. The carbon attached to boron was not observed due to quadrupolar relaxation;

**$^{11}$B NMR** (128 MHz, $CDCl_3$): $\delta_B$ 33.34 ppm.

**IR** (film): $\nu_{max}$ 2977, 2927, 1369, 1350, 1310, 1212, 1140, 968, 846, 671, 578 $cm^{-1}$.

All recorded spectroscopic data matched those previously reported in the literature.[4]

### 2,2'-(Pentane-1,2-diyl)bis(4,4,5,5-tetramethyl-1,3,2-dioxaborolane) (1e′)

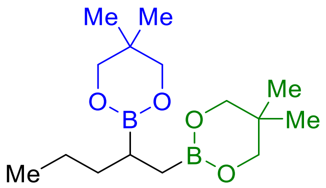


**1e'**

Prepared following **General Procedure A** using pent-1-ene (1.1 mL, 10 mmol, 1.0 equiv.), 5,5,5',5'-tetramethyl-2,2'-bi(1,3,2-dioxaborinane) (2.5 g, 11 mmol, 1.1 equiv.), $Cs_2CO_3$ (0.53 g, 1.5 mmol, 15 mol %), anhydrous methanol (2.0 mL, 50 mmol, 5.0 equiv.) and anhydrous THF (50 mL). Purification by flash column

chromatography (90:10 hexane/EA) gave the title compound (0.49 g, 16%) as a colorless oil.

**TLC**: $R_f$ = 0.40 (90:10 hexane/EtOAc, CAM stain).

**NMR Spectroscopy (*see spectra*):**

$^{1}$**H NMR** (400 MHz, $CDCl_3$): $\delta_H$ 3.55 (s, 8H), 1.45 – 1.35 (m, 1H), 1.35 – 1.25 (m, 2H), 1.25 – 1.17 (m, 1H), 1.00 – 0.96 (m, 1H), 0.94 (s, 12H), 0.86 (t, *J* = 7.0 Hz, 3H), 0.72 (d, *J* = 9.2 Hz, 2H) ppm ;

$^{13}$**C NMR** (101 MHz, $CDCl_3$) $\delta_C$ 72.1, 72.1, 36.6, 31.8, 31.7, 22.2, 22.0, 22.0, 14.6 ppm. The carbon attached to boron was not observed due to quadrupolar relaxation.

$^{11}$**B NMR** (128 MHz, $CDCl_3$) $\delta_B$ 30.94 ppm.

**IR** (film): $v_{max}$ 2957, 2927, 2873, 1476, 1412, 1377, 1333, 1289, 1248, 1220, 1175, 1145, 1083, 1010, 930, 835, 814, 735cm$^{-1}$.

**HRMS** (ESI$^+$): m/z calculated for $C_{15}H_{30}O_2B_2$ $[M+H]^+$, 297.2403; found, 297.2402.

**(*cis*)-1,2-Bis(4,4,5,5-tetramethyl-1,3,2-dioxaborolan-2-yl)cyclopentane (1g)**

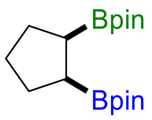


**1g**

Prepared following **General Procedure A** using cyclopentene (0.88 mL, 10 mmol, 1.0 equiv.), $B_2pin_2$ (2.79 g, 11.0 mmol, 1.10 equiv.), $Cs_2CO_3$ (0.53 g, 1.5 mmol, 15 mol %), anhydrous methanol (2.02 mL, 50.0 mmol, 5.0 equiv.) and anhydrous THF (50 mL). Purification by flash column chromatography (95:5 pentane/$Et_2O$) gave the title compound (2.0 g, 62%) as a colorless oil.

**TLC**: $R_f$ = 0.28 (95:5 *n*-hexane/EtOAc, CAM stain).

**NMR Spectroscopy (*see spectra*):**

$^{1}$**H NMR** (400 MHz, $CDCl_3$): $\delta_H$ 1.77 – 1.65 (m, 2H), 1.66 – 1.56 (m, 3H), 1.55 – 1.47 (m, 1H), 1.39 (td, *J* = 6.1, 2.5 Hz, 2H), 1.23 (s, 12H), 1.23 (s, 12H) ppm;

$^{13}$**C NMR** (101 MHz, $CDCl_3$): $\delta_C$ 82.9, 28.8, 25.8, 25.0, 24.9 ppm. The carbon attached to boron was not observed due to quadrupolar relaxation;

$^{11}$**B NMR** (128 MHz, $CDCl_3$): $\delta_B$ 34.64 ppm.

**IR** (film): $v_{max}$ 2976, 2941, 1740, 1377, 1308, 1219, 1141, 970, 858, 727, 670 cm$^{-1}$.

All recorded spectroscopic data matched those previously reported in the literature.[6]

**(*cis*)-1,2-Bis(4,4,5,5-tetramethyl-1,3,2-dioxaborolan-2-yl)cyclohexane (1h)**

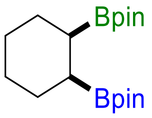


**1h**

Prepared following **General Procedure A** using cyclohexene (1.01 mL, 10.0 mmol, 1.0 equiv.), $B_2pin_2$ (2.79 g, 11.0 mmol, 1.1 equiv.), $Cs_2CO_3$ (0.53 g, 1.5 mmol, 15 mol %), anhydrous methanol (2.02 mL, 50.0 mmol, 5.0 equiv.) and anhydrous THF (50 mL). Purification by flash column chromatography (95:5 pentane/$Et_2O$) gave the title compound (2.16 g, 67%) as a colorless oil.

**TLC**: $R_f$ = 0.28 (95:5 *n*-hexane/EtOAc, CAM stain).

**NMR Spectroscopy (*see spectra*):**

$^1$**H NMR** (400 MHz, $CDCl_3$): $\delta_H$ 1.66 – 1.48 (m, 4H), 1.48 – 1.31 (m, 4H), 1.29 – 1.14 (m, 26H) ppm;

$^{13}$**C NMR** (101 MHz, $CDCl_3$): $\delta_C$ 82.9, 28.2, 27.0, 25.0, 25.0 ppm. The carbon attached to boron was not observed due to quadrupolar relaxation;

$^{11}$**B NMR** (128 MHz, $CDCl_3$): $\delta_B$ 34.44 ppm.

**IR** (film): $\nu_{max}$ 2976, 2924, 2850, 1740, 1412, 1378, 1306, 1237, 1216, 1144, 970, 859 cm$^{-1}$.

All recorded spectroscopic data matched those previously reported in the literature.[7]

**(1*R*,2*S*,3*R*,4*S*)-2,3-Bis(4,4,5,5-tetramethyl-1,3,2-dioxaborolan-2-yl)bicyclo[2.2.1]heptane (1i)**

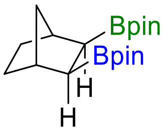


**1i**

Prepared following **General Procedure A** using bicyclo[2.2.1]hept-2-ene (0.47 g, 5.0 mmol, 1.0 equiv.), $B_2pin_2$ (1.4 g, 5.5 mmol, 1.1 equiv.), $Cs_2CO_3$ (244 mg, 0.75 mmol, 15 mol %), anhydrous methanol (1.0 mL, 25.0 mmol, 5.0 equiv.) and anhydrous THF (25 mL). Purification by flash column chromatography (91:9 pentane/$Et_2O$) gave the title compound (1.1 g, 64%) as a white solid.

**TLC**: $R_f$ = 0.32 (91:9 *n*-hexane/EtOAc, CAM stain)

**NMR Spectroscopy (*see spectra*):**

$^1$**H NMR** (400 MHz, $CDCl_3$): $\delta_H$ 2.26 (dd, *J* = 2.5, 1.7 Hz, 2H), 1.56 – 1.48 (m, 2H), 1.45 (dt, *J* = 9.3, 1.9 Hz, 1H), 1.30 – 1.24 (m, 2H), 1.23 (s, 24H), 1.15 – 1.07 (m, 3H) ppm;

$^{13}$**C NMR** (101 MHz, $CDCl_3$): $\delta_C$ 83.0, 39.4, 38.1, 32.2, 25.0, 24.9 ppm. The carbon attached to boron was not observed due to quadrupolar relaxation;

**$^{11}$B NMR** (128 MHz, $CDCl_3$): $\delta_B$ 32.72 ppm.

**IR** (film): $\nu_{max}$ 2957, 2947, 2865, 1714, 1406, 1362, 1307, 1220, 1143, 1105, 1010, 857, 669, 529 cm$^{-1}$.

**HRMS** (EI$^+$): m/z calculated for $C_{19}H_{34}O_4B_2$ $[M-CH_3]^+$, 333.2403; found, 333.2399.

### 2,2'-(1-Cyclopropylethane-1,2-diyl)bis(4,4,5,5-tetramethyl-1,3,2-dioxaborolane) (1l)

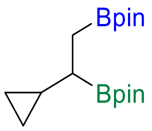


**1l**

Prepared following **General Procedure A** using vinylcyclopropane (0.34 g, 5.0 mmol, 1.0 equiv.), $B_2pin_2$ (1.4 g, 5.5 mmol, 1.1 equiv.), $Cs_2CO_3$ (244 mg, 0.75 mmol, 15 mol %), anhydrous methanol (1.0 mL, 25.0 mmol, 5.0 equiv.) and anhydrous THF (25 mL). Purification by flash column chromatography (91:9 pentane/$Et_2O$) gave the title compound (0.87 g, 54%) as a colorless oil.

**TLC**: $R_f$ = 0.40 (91:9 *n*-hexane/EtOAc, CAM stain)

**NMR Spectroscopy (*see spectra*):**

**$^1$H NMR** (400 MHz, $CDCl_3$): $\delta_H$ 1.24 (s, 12H), 1.22 (s, 12H), 0.97 – 0.88 (m, 2H), 0.70 (dtt, *J* = 9.7, 7.9, 4.9 Hz, 1H), 0.52 – 0.42 (m, 1H), 0.41 – 0.32 (m, 2H), 0.18 – 0.08 (m, 1H), 0.08 – -0.01 (m, 1H) ppm;

**$^{13}$C NMR** (101 MHz, $CDCl_3$): $\delta_C$ 83.0, 82.9, 25.1, 24.9, 24.9, 24.9, 15.0, 4.9, 4.3 ppm. The carbon attached to boron was not observed due to quadrupolar relaxation;

**$^{11}$B NMR** (128 MHz, $CDCl_3$): $\delta_B$ 34.57 ppm.

**IR** (film): $\nu_{max}$ 2978, 1370,1311, 1215, 1140,969, 883, 844, 733, 670 cm$^{-1}$.

**HRMS** (EI$^+$): m/z calculated for $C_{17}H_{32}O_4B_2$ $[M-CH_3]^+$, 307.2246; found, 307.2245.

All recorded spectroscopic data matched those previously reported in the literature.[8]

### 4-[4,4-Dimethyl-1-(4,4,5,5-tetramethyl-1,3,2-dioxaborolan-2-yl)pentan-2-yl]benzonitrile (6aa)

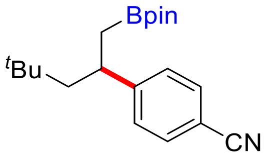


**6aa**

Prepared following **General Procedure B**, using 2,2'-(4,4-dimethylpentane-1,2-diyl)bis(4,4,5,5-tetramethyl-1,3,2-dioxaborolane) (**1a**) (70.5 mg, 0.2 mmol, 1.0 equiv.), PhLi (1.9 M in dibutyl ether, 0.12 mL, 0.22 mmol, 1.1 equiv.), 4-bromobenzonitrile (**5a**) (54.6 mg, 0.30 mmol, 1.5 equiv.), 4-CzIPN (7.9 mg, 0.01 mmol, 5.0 mol %),

and Ni(TMHD)$_2$ (8.5 mg, 0.02 mmol, 10.0 mol %). Purification by flash column chromatography (90:10 pentane/$Et_2O$) gave the title compound **6aa** (53 mg, 81%) as a white solid.

**TLC**: $R_f$ = 0.28 (91:9 *n*-hexane/EtOAc, CAM stain)

**NMR Spectroscopy (*see spectra*):**

$^{1}$**H NMR** (400 MHz, $CDCl_3$): $\delta_H$ 7.52 (d, *J* = 8.4 Hz, 2H), 7.34 (d, *J* = 8.4 Hz, 2H), 3.03 (tdd, *J* = 9.2, 7.0, 3.4 Hz, 1H), 1.72 (dd, *J* = 14.2, 9.5 Hz, 1H), 1.55 (dd, *J* = 14.2, 3.4 Hz, 1H), 1.15 (dd, *J* = 15.4, 7.0 Hz, 1H), 1.09 (s, 6H), 1.08 (s, 6H), 1.06 (dd, *J* = 15.4, 6.6 Hz, 1H), 0.74 (s, 9H) ppm;

$^{13}$**C NMR** (101 MHz, $CDCl_3$): $\delta_C$ 155.2, 132.1, 128.7, 119.4, 109.5, 83.3, 52.9, 38.8, 31.6, 30.3, 24.9, 24.8 ppm. The carbon attached to boron was not observed due to quadrupolar relaxation;

$^{11}$**B NMR** (128 MHz, $CDCl_3$): $\delta_B$ 32.54 ppm.

**IR** (film): $\nu_{max}$ 2954, 2227, 1606, 1468, 1371, 1324, 1144, 967, 850, 834, 673 cm$^{-1}$.

**HRMS** (ESI$^+$): m/z calculated for $C_{20}H_{30}BNNaO_2$ [M+Na]$^+$, 350.2265; found, 350.2282.

### 2-{4,4-Dimethyl-2-[4-(trifluoromethyl)phenyl]pentyl}-4,4,5,5-tetramethyl-1,3,2-dioxaborolane (6ab)

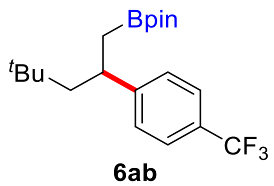


**6ab**

Prepared following **General Procedure B**, using 2,2'-(4,4-dimethylpentane-1,2-diyl)bis(4,4,5,5-tetramethyl-1,3,2-dioxaborolane) (**1a**) (70.5 mg, 0.2 mmol, 1.0 equiv.), PhLi (1.9 M in dibutyl ether, 0.12 mL, 0.22 mmol, 1.1 equiv.), 1-bromo-4-(trifluoromethyl)benzene (**5b**) (67.5 mg, 0.30 mmol, 1.5 equiv.), 4-CzIPN (7.9 mg, 0.01 mmol, 5.0 mol %), and Ni(TMHD)$_2$ (8.5 mg, 0.02 mmol, 10.0 mol %). Purification by flash column chromatography (98:2 pentane/ $Et_2O$) gave the title compound **6ab** (62.2 mg, 84%) as a white solid.

**TLC**: $R_f$ = 0.31 (97:3 pentane/$Et_2O$, CAM stain)

**NMR Spectroscopy (*see spectra*):**

$^{1}$**H NMR** (400 MHz, $CDCl_3$): $\delta_H$ 7.41 (d, *J* = 7.6 Hz, 2H), 7.27 (d, *J* = 7.6 Hz, 2H), 2.97 (tdd, *J* = 9.1, 7.1, 3.4 Hz, 1H), 1.69 (dd, *J* = 14.1, 9.5 Hz, 1H), 1.49 (dd, *J* = 14.1, 3.4 Hz, 1H), 1.14 – 0.96 (m, 14H), 0.68 (s, 9H) ppm;

$^{13}$**C NMR** (101 MHz, $CDCl_3$): $\delta_C$ 153.5, 128.2, 128.2 (q, $^{2}J_{C\text{-}F}$ = 32.1 Hz), 125.2 (q, $^{3}J_{C\text{-}F}$ = 3.9 Hz), 124.6 (q, $^{1}J_{C\text{-}F}$ = 271.5 Hz), 83.2, 53.0, 38.5, 31.6, 30.4, 24.9, 24.8 ppm. The carbon attached to boron was not observed due to quadrupolar relaxation;

$^{19}$**F NMR** (377 MHz, $CDCl_3$): $\delta_F$ -62.10 ppm;

$^{11}$**B NMR** (128 MHz, $CDCl_3$): $\delta_B$ 31.53 ppm.

**IR** (film): $v_{max}$ 2978, 2953, 1617, 1468, 1365, 1322, 1162, 1122, 1068, 1018, 967, 836, 609 cm$^{-1}$.

**HRMS** (ESI$^+$): m/z calcd for $C_{20}H_{30}BF_3O_2$ [M+Na]$^+$, 393.2187; found, 393.2187.

### 1-{4-[4,4-Dimethyl-1-(4,4,5,5-tetramethyl-1,3,2-dioxaborolan-2-yl)pentan-2-yl]phenyl}ethan-1-one (6ac)

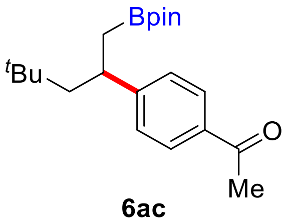


**6ac**

Prepared following **General Procedure B**, using 2,2'-(4,4-dimethylpentane-1,2-diyl)bis(4,4,5,5-tetramethyl-1,3,2-dioxaborolane) (**1a**) (70.5 mg, 0.2 mmol, 1.0 equiv.), PhLi (1.9 M in dibutyl ether, 0.12 mL, 0.22 mmol, 1.1 equiv.), 1-(4-bromophenyl)ethan-1-one (**5c**) (59.7 mg, 0.30 mmol, 1.5 equiv.), 4-CzIPN (7.9 mg, 0.01 mmol, 5.0 mol %), and Ni(TMHD)$_2$ (8.5 mg, 0.02 mmol, 10.0 mol %). Purification by flash column chromatography (89:11 pentane/ $Et_2O$) gave the title compound **6ac** (47 mg, 68%) as a colorless oil.

**TLC**: $R_f$ = 0.23 (91:9 *n*-hexane/EtOAc, CAM stain)

**NMR Spectroscopy (*see spectra*):**

$^1$**H NMR** (400 MHz, $CDCl_3$): $\delta_H$ 7.83 (d, *J* = 8.3 Hz, 2H), 7.32 (d, *J* = 8.4 Hz, 2H), 3.04 (tdd, *J* = 10.3, 7.4, 3.3 Hz, 1H), 2.55 (s, 3H), 1.76 (dd, *J* = 14.1, 9.6 Hz, 1H), 1.54 (dd, *J* = 14.1, 3.3 Hz, 1H), 1.15 (dd, *J* = 15.3, 7.3 Hz, 1H), 1.11 – 1.03 (m, 13H), 0.74 (s, 9H) ppm;

$^{13}$**C NMR** (101 MHz, $CDCl_3$): $\delta_C$ 198.0, 155.4, 135.0, 128.5, 128.0, 83.2, 52.9, 38.6, 31.6, 30.3, 26.7, 24.9, 24.8 ppm. The carbon attached to boron was not observed due to quadrupolar relaxation;

$^{11}$**B NMR** (128 MHz, $CDCl_3$): $\delta_B$ 33.01 ppm.

**IR** (film): $v_{max}$ 2951, 1683, 1606, 1468, 1415, 1363, 1323, 1267, 1144, 967, 850, 601 cm$^{-1}$.

**HRMS** (EI$^+$): m/z calculated for $C_{21}H_{33}BO_3$ [M]$^+$, 344.2517; found, 344.2513.

### Ethyl 4-[4,4-dimethyl-1-(4,4,5,5-tetramethyl-1,3,2-dioxaborolan-2-yl)pentan-2-yl]benzoate (6ad)

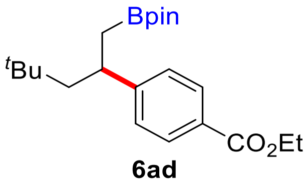


**6ad**

Prepared following **General Procedure B**, using 2,2'-(4,4-dimethylpentane-1,2-diyl)bis(4,4,5,5-tetramethyl-1,3,2-dioxaborolane) (**1a**) (70.5 mg, 0.2 mmol, 1.0 equiv.), PhLi (1.9 M in dibutyl ether, 0.12 mL, 0.22 mmol, 1.1

equiv.), ethyl 4-bromobenzoate (**5d**) (68.7 mg, 0.30 mmol, 1.5 equiv.), 4-CzIPN (7.9 mg, 0.01 mmol, 5.0 mol %), and $Ni(TMHD)_2$ (8.5 mg, 0.02 mmol, 10.0 mol %). Purification by flash column chromatography (91:9 pentane/ $Et_2O$) gave the title compound **6ad** (57 mg, 76%) as a colorless oil.

**TLC**: $R_f$ = 0.43 (91:9 *n*-hexane/EtOAc, CAM stain)

**NMR Spectroscopy (*see spectra*):**

$^1$**H NMR** (400 MHz, $CDCl_3$): $\delta_H$ 7.92 (d, *J* = 8.3 Hz, 2H), 7.30 (d, *J* = 8.3 Hz, 2H), 4.35 (q, *J* = 7.1 Hz, 2H), 3.20 – 2.85 (m, 1H), 1.76 (dd, *J* = 14.1, 9.7 Hz, 1H), 1.38 (t, *J* = 7.1 Hz, 3H), 1.15 (dd, *J* = 15.4, 7.3 Hz, 1H), 1.10 (s, 6H), 1.08 (s, 6H), 1.07 – 1.03 (m, 1H), 0.74 (s, 9H) ppm;

$^{13}$**C NMR** (101 MHz, $CDCl_3$): $\delta_C$ 167.0, 154.9, 129.6, 128.0, 127.8, 83.2, 60.8, 53.0, 38.7, 31.6, 30.4, 24.9, 24.8, 14.5 ppm. The carbon attached to boron was not observed due to quadrupolar relaxation;

$^{11}$**B NMR** (128 MHz, $CDCl_3$): $\delta_B$ 32.52 ppm.

**IR** (film): $\nu_{max}$ 2978, 2952, 1717, 1609, 1365, 1323, 1275, 1145, 1101, 968, 846, 772, 710 $cm^{-1}$.

**HRMS** ($EI^+$): m/z calculated for $C_{22}H_{35}BO_4$ $[M]^+$, 374.2623; found, 374.2619.

**5-[4,4-Dimethyl-1-(4,4,5,5-tetramethyl-1,3,2-dioxaborolan-2-yl)pentan-2-yl]isobenzofuran-1(3*H*)-one (6ae)**

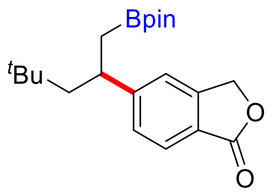


**6ae**

Prepared following **General Procedure B**, using 2,2'-(4,4-dimethylpentane-1,2-diyl)bis(4,4,5,5-tetramethyl-1,3,2-dioxaborolane) (**1a**) (70.5 mg, 0.2 mmol, 1.0 equiv.), PhLi (1.9 M in dibutyl ether, 0.12 mL, 0.22 mmol, 1.1 equiv.), 5-bromoisobenzofuran-1(3*H*)-one (**5e**) (63.9 mg, 0.30 mmol, 1.5 equiv.), 4-CzIPN (7.9 mg, 0.01 mmol, 5.0 mol %), and $Ni(TMHD)_2$ (8.5 mg, 0.02 mmol, 10.0 mol %). Purification by flash column chromatography (80:20 *n*-hexane/EtOAc) gave the title compound **6ae** (38 mg, 53%) as a colorless oil.

**TLC**: $R_f$ = 0.30 (80:40 *n*-hexane/EtOAc, CAM stain)

**NMR Spectroscopy (*see spectra*):**

$^1$**H NMR** (400 MHz, $CDCl_3$): $\delta_H$ 7.77 (d, *J* = 7.9 Hz, 1H), 7.41 (d, *J* = 7.9 Hz, 1H), 7.34 (s, 1H), 5.25 (d, *J* = 4.9 Hz, 2H), 3.11 (tdd, *J* = 9.0, 7.0, 3.5 Hz, 1H), 1.77 (dd, *J* = 14.2, 9.4 Hz, 1H), 1.58 (dd, *J* = 14.2, 3.4 Hz, 1H), 1.18 (dd, *J* = 15.5, 7.1 Hz, 1H), 1.14 – 1.08 (m, 1H), 1.07 (s, 6H), 1.06 (s, 6H), 0.74 (s, 9H) ppm;

$^{13}$**C NMR** (101 MHz, $CDCl_3$): $\delta_C$ 171.3, 157.0, 147.0, 129.2, 125.6, 123.4, 121.0, 83.2, 69.6, 53.1, 39.0, 31.6, 30.3, 24.8, 24.8 ppm. The carbon attached to boron was not observed due to quadrupolar relaxation;

$^{11}$**B NMR** (128 MHz, $CDCl_3$): $\delta_B$ 33.07 ppm.

**IR** (film): $v_{max}$ 2954, 1761, 1617, 1467, 1363, 1319, 1274, 1142, 1039, 1006, 847, 733, 699 cm$^{-1}$.

**HRMS** (EI$^+$): m/z calculated for $C_{21}H_{31}BO_4$ [M]$^+$, 358.2310; found, 358.2309.

### 4-[4,4-Dimethyl-1-(4,4,5,5-tetramethyl-1,3,2-dioxaborolan-2-yl)pentan-2-yl]-2-fluorobenzonitrile (6af)

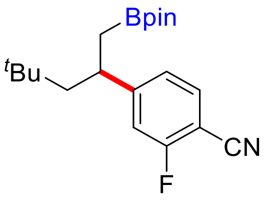


**6af**

Prepared following **General Procedure B**, using 2,2'-(4,4-dimethylpentane-1,2-diyl)bis(4,4,5,5-tetramethyl-1,3,2-dioxaborolane) (**1a**) (70.5 mg, 0.2 mmol, 1.0 equiv.), PhLi (1.9 M in dibutyl ether, 0.12 mL, 0.22 mmol, 1.1 equiv.), 4-bromo-2-fluorobenzonitrile (**5f**) (60 mg, 0.30 mmol, 1.5 equiv.), 4-CzIPN (7.9 mg, 0.01 mmol, 5.0 mol %), and Ni(TMHD)$_2$ (8.5 mg, 0.02 mmol, 10.0 mol %). Purification by flash column chromatography (91:9 pentane/$Et_2O$) gave the title compound **6af** (51 mg, 74%) as a colorless oil.

**TLC**: $R_f$ = 0.29 (91:9 *n*-hexane/EtOAc, CAM stain)

**NMR Spectroscopy (*see spectra*):**

$^1$**H NMR** (400 MHz, $CDCl_3$): $\delta_H$ 7.48 (dd, $J$ = 8.0, 6.7 Hz, 1H), 7.13 (dd, $J$ = 8.0, 1.5 Hz, 1H), 7.09 (dd, $J$ = 6.7, 1.5 Hz, 1H), 3.08 – 2.98 (m, 1H), 1.70 (dd, $J$ = 14.2, 9.5 Hz, 1H), 1.54 (dd, $J$ = 14.2, 3.3 Hz, 1H), 1.17 – 1.14 (m, 1H), 1.11 (s, 12H), 1.03 (dd, $J$ = 15.6, 8.7 Hz, 1H), 0.75 (s, 9H) ppm;

$^{13}$**C NMR** (101 MHz, $CDCl_3$): $\delta_C$ 163.2 (d, $^1J_{C\text{-}F}$ = 258.2 Hz), 158.8 (d, $^3J_{C\text{-}F}$ = 7.2 Hz), 133.2, 124.4 (d, $^3J_{C\text{-}F}$ = 3.1 Hz), 115.6 (d, $^2J_{C\text{-}F}$ = 18.9 Hz), 114.5, 98.4 (d, $^2J_{C\text{-}F}$ = 15.8 Hz), 83.4, 52.6, 38.8, 31.6, 30.3, 24.8, 24.8 ppm. The carbon attached to boron was not observed due to quadrupolar relaxation;

$^{19}$**F NMR** (376 MHz, $CDCl_3$): $\delta_F$ -107.13 (dd, $J$ = 10.4, 6.6 Hz) ppm;

$^{11}$**B NMR** (128 MHz, $CDCl_3$): $\delta_B$ 32.11 ppm.

**IR** (film): $v_{max}$ 2954, 2234, 1739, 1619, 1568, 1499, 1432, 1365, 1323, 1142, 1108, 968, 847, 657 cm$^{-1}$.

**HRMS** (EI$^+$): m/z calculated for $C_{20}H_{29}BFNO_2$ [M]$^+$, 345.2270; found, 345.2269.

**4-(1-Hydroxy-4,4-dimethylpentan-2-yl)-*N,N*-dimethylbenzamide (6ag)**

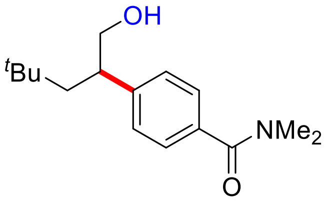

**6ag**

Prepared following **General Procedure B**, using 2,2'-(4,4-dimethylpentane-1,2-diyl)bis(4,4,5,5-tetramethyl-1,3,2-dioxaborolane) (**1a**) (70.5 mg, 0.2 mmol, 1.0 equiv.), PhLi (1.9 M in dibutyl ether, 0.12 mL, 0.22 mmol, 1.1 equiv.), 4-bromo-*N*,*N*-dimethylbenzamide (**5g**) (68.5 mg, 0.30 mmol, 1.5 equiv.), 4-CzIPN (7.9 mg, 0.01 mmol, 5.0 mol %), and $Ni(TMHD)_2$ (8.5 mg, 0.02 mmol, 10.0 mol %). The crude material was oxidized following the procedure mentioned above. Purification by flash column chromatography (97:3 $Et_2O$/MeOH) gave the title compound **6ag** (25.4 mg, 48%) as a colorless oil.

**TLC**: $R_f$ = 0.20 (67:33 *n*-hexane/EtOAc, $KMnO_4$ stain)

**NMR Spectroscopy (*see spectra*):**

$^1$**H NMR** (400 MHz, $CDCl_3$): $\delta_H$ 7.37 (d, *J* = 8.3 Hz, 2H), 7.27 (d, *J* = 8.1 Hz, 2H), 3.71 – 3.55 (m, 2H), 3.09 (brs, 3H), 2.99 (brs, 3H), 2.90 (tdd, *J* = 8.5, 5.9, 3.4 Hz, 1H), 1.65 (dd, *J* = 14.1, 8.4 Hz, 1H), 1.54 (dd, *J* = 14.1, 3.5 Hz, 1H), 1.38 (brs, 1H), 0.81 (s, 9H) ppm;

$^{13}$**C NMR** (101 MHz, $CDCl_3$): $\delta_C$ 171.7, 146.3, 134.6, 128.4, 127.7, 69.0, 45.8, 45.5, 39.8, 35.5, 31.3, 30.2 ppm.

**IR** (film): $\nu_{max}$ 3410, 2950, 1738, 1617, 1491, 1394, 1267, 1218, 1081, 842 $cm^{-1}$.

**HRMS** ($EI^+$): m/z calculated for $C_{16}H_{24}O_2N$ $[M-H]^+$, 262.1802; found, 262.1801.

**2-{2-[(1,1'-Biphenyl)-4-yl]-4,4-dimethylpentyl}-4,4,5,5-tetramethyl-1,3,2-dioxaborolane (6ah)**

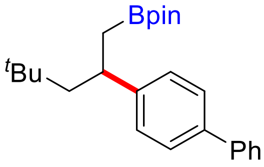

**6ah**

Prepared following **General Procedure B**, using 2,2'-(4,4-dimethylpentane-1,2-diyl)bis(4,4,5,5-tetramethyl-1,3,2-dioxaborolane) (**1a**) (70.5 mg, 0.2 mmol, 1.0 equiv.), PhLi (1.9 M in dibutyl ether, 0.12 mL, 0.22 mmol, 1.1 equiv.), 4-bromo-1,1'-biphenyl (**5h**) (70.0 mg, 0.30 mmol, 1.5 equiv.), 4-CzIPN (7.9 mg, 0.01 mmol, 5.0 mol %), and $Ni(TMHD)_2$ (8.5 mg, 0.02 mmol, 10.0 mol %). Purification by flash column chromatography (98:2 pentane/$Et_2O$) gave the title compound **6ah** (45 mg, 59%) as a colorless oil.

**TLC**: $R_f$ = 0.41 (97:3 *n*-hexane/EtOAc, CAM stain)

**NMR Spectroscopy (*see spectra*):**

**$^{1}$H NMR** (400 MHz, $CDCl_3$): $\delta_H$ 7.63 – 7.56 (m, 2H), 7.48 (d, *J* = 8.2 Hz, 2H), 7.42 (t, *J* = 8.3 Hz, 2H), 7.34 – 7.29 (m, 3H), 3.04 (tdd, *J* = 8.7, 6.9, 3.5 Hz, 1H), 1.80 (dd, *J* = 13.4, 8.8 Hz, 1H), 1.56 (dd, *J* = 14.5, 3.0 Hz, 1H), 1.28 – 1.13 (m, 2H), 1.11 (s, 6H), 1.08 (s, 6H), 0.79 (s, 9H) ppm;

**$^{13}$C NMR** (101 MHz, $CDCl_3$): $\delta_C$ 148.4, 141.4, 138.5, 128.8, 128.2, 127.0, 127.0, 126.9, 83.1, 53.2, 38.2, 31.6, 30.4, 24.9, 24.8 ppm. The carbon attached to boron was not observed due to quadrupolar relaxation;

**$^{11}$B NMR** (128 MHz, $CDCl_3$): $\delta_B$ 33.49 ppm.

**IR** (film): $\nu_{max}$ 2950, 1739, 1487, 1365, 1322, 1145, 967, 849, 765, 734, 697 $cm^{-1}$.

**HRMS** ($EI^+$): m/z calculated for $C_{25}H_{35}BO_2$ $[M]^+$, 378.2725; found, 378.2719.

**2-[4,4-Dimethyl-2-(naphthalen-2-yl)pentyl]-4,4,5,5-tetramethyl-1,3,2-dioxaborolane (6ai)**

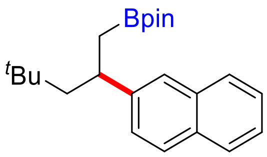


**6ai**

Prepared following **General Procedure B**, using 2,2'-(4,4-dimethylpentane-1,2-diyl)bis(4,4,5,5-tetramethyl-1,3,2-dioxaborolane) (**1a**) (70.5 mg, 0.2 mmol, 1.0 equiv.), PhLi (1.9 M in dibutyl ether, 0.12 mL, 0.22 mmol, 1.1 equiv.), 2-bromonaphthalene (**5i**) (62.2 mg, 0.30 mmol, 1.5 equiv.), 4-CzIPN (7.9 mg, 0.01 mmol, 5.0 mol %), and $Ni(TMHD)_2$ (8.5 mg, 0.02 mmol, 10.0 mol %). Purification by flash column chromatography (98:2 pentane/$Et_2O$) gave the title compound **6ai** (32 mg, 45%) as a colorless oil.

**TLC**: $R_f$ = 0.25 (95:5 *n*-hexane/EtOAc, CAM stain)

**NMR Spectroscopy (*see spectra*):**

**$^{1}$H NMR** (400 MHz, $CDCl_3$): $\delta_H$ 7.79 – 7.71 (m, 3H), 7.66 (d, *J* = 1.8 Hz, 1H), 7.47 – 7.34 (m, 3H), 3.18 (tdd, *J* = 9.0, 7.3, 3.4 Hz, 1H), 1.89 (dd, *J* = 14.1, 9.6 Hz, 1H), 1.60 (dd, *J* = 14.1, 3.4 Hz, 1H), 1.24 (dd, *J* = 15.2, 7.3 Hz, 1H), 1.17 (dd, *J* = 15.2, 8.6 Hz, 1H), 1.06 (s, 6H), 1.03 (s, 6H), 0.78 (s, 9H) ppm;

**$^{13}$C NMR** (101 MHz, $CDCl_3$): $\delta_C$ 146.7, 133.7, 132.2, 127.8, 127.7, 127.6, 126.3, 126.1, 125.7, 124.9, 83.0, 52.9, 38.7, 31.6, 30.4, 24.9, 24.8 ppm. The carbon attached to boron was not observed due to quadrupolar relaxation;

**$^{11}$B NMR** (128 MHz, $CDCl_3$): $\delta_B$ 33.56 ppm.

**IR** (film): $\nu_{max}$ 2950, 1739, 1467, 1361, 1319, 1144, 968, 855, 846, 816, 745, 477 $cm^{-1}$.

**HRMS** ($EI^+$): m/z calculated for $C_{23}H_{33}BO_2$ $[M]^+$, 352.2568; found, 352.2565.

**2-(4-Fluorophenyl)-4,4-dimethylpentan-1-ol (6aj)**

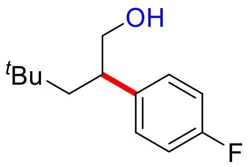

**6aj**

Prepared following **General Procedure B**, using 2,2'-(4,4-dimethylpentane-1,2-diyl)bis(4,4,5,5-tetramethyl-1,3,2-dioxaborolane) (**1a**) (70.5 mg, 0.2 mmol, 1.0 equiv.), PhLi (1.9 M in dibutyl ether, 0.12 mL, 0.22 mmol, 1.1 equiv.), 1-fluoro-4-iodobenzene (**5j**) (66.6 mg, 0.30 mmol, 1.5 equiv.), 4-CzIPN (7.9 mg, 0.01 mmol, 5.0 mol %), and Ni(TMHD)$_2$ (8.5 mg, 0.02 mmol, 10.0 mol %). The crude material was oxidized following the procedure mentioned above. Purification by flash column chromatography (80:20 pentane/EtOAc) gave the title compound **6aj** (18 mg, 43%) as a colorless oil.

**TLC**: $R_f$ = 0.22 (80:20 *n*-hexane/EtOAc, $KMnO_4$ stain)

**NMR Spectroscopy (*see spectra*):**

$^1$**H NMR** (400 MHz, $CDCl_3$): $\delta_H$ 7.19 (dd, *J* = 8.7, 5.4 Hz, 2H), 7.00 (dd, *J* = 8.7, 5.7 Hz, 2H), 3.69-3.60 (m, 1H), 3.55 (dd, *J* = 10.6, 5.7 Hz, 1H), 2.86 (tdd, *J* = 8.5, 5.7, 3.5 Hz, 1H), 1.60 (dd, *J* = 14.0, 8.5 Hz, 1H), 1.52 (dd, *J* = 14.0, 3.5 Hz, 1H), 1.37 (brs, 1H), 0.80 (s, 9H) ppm;

$^{13}$**C NMR** (100 MHz, $CDCl_3$): $\delta_C$ 161.6 (d, $^1J_{C\text{-}F}$ = 244.3 Hz), 139.9 (d, $^4J_{C\text{-}F}$ = 3.0 Hz), 129.6 (d, $^3J_{C\text{-}F}$ = 7.8 Hz), 115.4 (d, $^2J_{C\text{-}F}$ = 21.1 Hz), 69.0, 45.9, 44.6, 31.1, 30.0 ppm;

$^{19}$**F NMR** (377 MHz, $CDCl_3$): $\delta_F$ -116.66 (tt, *J* = 8.7, 5.3 Hz) ppm.

**IR** (film): $\nu_{max}$ 3354, 2952, 2867, 1738, 1605, 1508, 1475, 1365, 1222, 1157, 1032, 830, 733, 547 cm$^{-1}$.

**HRMS** (ESI$^+$) m/z calcd for $C_{13}H_{19}FNaO$ $[M+Na]^+$, 233.1312; found, 233.1312.

**2-(4-Chlorophenyl)-4,4-dimethylpentan-1-ol (6ak)**

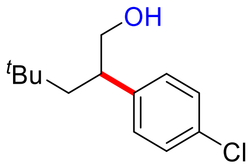

**6ak**

Prepared following **General Procedure B**, using 2,2'-(4,4-dimethylpentane-1,2-diyl)bis(4,4,5,5-tetramethyl-1,3,2-dioxaborolane) (**1a**) (70.5 mg, 0.2 mmol, 1.0 equiv.), PhLi (1.9 M in dibutyl ether, 0.12 mL, 0.22 mmol, 1.1 equiv.), 1-chloro-4-iodobenzene (**5k**) (71.5 mg, 0.30 mmol, 1.5 equiv.), 4-CzIPN (7.9 mg, 0.01 mmol, 5.0 mol %), and Ni(TMHD)$_2$ (8.5 mg, 0.02 mmol, 10.0 mol %). The crude material was oxidized following the procedure mentioned above. Purification by flash column chromatography (89:11 *n*-hexane/EtOAc) gave the title compound **6ak** (25 mg, 55%) as a colorless oil.

**TLC**: $R_f$ = 0.42 (86:14 *n*-hexane/EtOAc, $KMnO_4$ stain)

**NMR Spectroscopy (*see spectra*):**

$^1$**H NMR** (400 MHz, $CDCl_3$): $\delta_H$ 7.29 (d, *J* = 8.5 Hz, 2H), 7.18 (d, *J* = 8.5 Hz, 2H), 3.66 (dd, *J* = 10.7, 5.9 Hz, 1H), 3.57 (dd, *J* = 10.7, 8.4 Hz, 1H), 2.86 (tdd, *J* = 8.7, 5.9, 3.5 Hz, 1H), 1.61 (dd, *J* = 14.0, 8.4 Hz, 1H), 1.52 (dd, *J* = 14.0, 3.5 Hz, 1H), 1.33 (brs, 1H), 0.81 (s, 9H) ppm;

$^{13}$**C NMR** (100 MHz, $CDCl_3$): $\delta_C$ 143.0, 132.4, 129.7, 128.9, 69.1, 45.9, 45.0, 31.3, 30.2 ppm.

**IR** (film): $\nu_{max}$ 3346, 2953, 2867, 1738, 1491, 1475, 1365, 1217, 1092, 1062, 1014, 824 cm$^{-1}$.

**HRMS** (ESI$^+$) m/z calcd for $C_{13}H_{19}ClNaO$ [M+Na]$^+$, 249.1017; found, 249.1022.

**4,4-Dimethyl-2-(*p*-tolyl)pentan-1-ol (6al)**

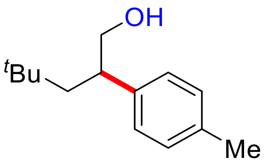


**6al**

Prepared following **General Procedure B**, using 2,2'-(4,4-dimethylpentane-1,2-diyl)bis(4,4,5,5-tetramethyl-1,3,2-dioxaborolane) (**1a**) (70.5 mg, 0.2 mmol, 1.0 equiv.), PhLi (1.9 M in dibutyl ether, 0.12 mL, 0.22 mmol, 1.1 equiv.), 1-iodo-4-methylbenzene (**5l**) (65.4 mg, 0.30 mmol, 1.5 equiv.), 4-CzIPN (7.9 mg, 0.01 mmol, 5.0 mol %), and $Ni(TMHD)_2$ (8.5 mg, 0.02 mmol, 10.0 mol %). The crude material was oxidized following the procedure mentioned above. Purification by flash column chromatography (83:17 pentane/EtOAc) gave the title compound **6al** (18 mg, 43%) as a colorless oil.

**TLC**: $R_f$ = 0.38 (80:20 *n*-hexane/EtOAc, $KMnO_4$ stain)

**NMR Spectroscopy (*see spectra*):**

$^1$**H NMR** (400 MHz, $CDCl_3$): $\delta_H$ 7.12 (s, 4H), 3.66 (ddd, *J* = 10.6, 8.4, 5.8 Hz, 1H), 3.57 (ddd, *J* = 10.4, 8.6, 3.7 Hz, 1H), 2.84 (tdd, *J* = 8.6, 5.8, 3.5 Hz, 1H), 2.33 (s, 3H), 1.64 (dd, *J* = 14.0, 8.3 Hz, 1H), 1.49 (dd, *J* = 14.0, 3.5 Hz, 1H), 1.27 – 1.19 (m, 1H), 0.82 (s, 9H) ppm;

$^{13}$**C NMR** (101 MHz, $CDCl_3$): $\delta_C$ 141.1, 136.2, 129.5, 128.3, 69.2, 46.0, 45.1, 31.3, 30.2, 21.2 ppm.

**IR** (film): $\nu_{max}$ 3347, 2951, 2866, 1738, 1513, 1466, 1365, 1217, 1063, 1033, 814, 547 cm$^{-1}$.

**HRMS** (ESI$^+$): m/z calculated for $C_{14}H_{22}NaO$ [M+Na]$^+$, 229.1563; found, 229.1563.

**2-[2-(4-Methoxyphenyl)-4,4-dimethylpentyl]-4,4,5,5-tetramethyl-1,3,2-dioxaborolane (6am)**

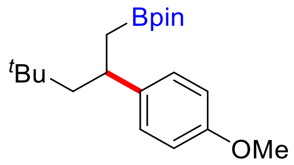


**6am**

Prepared following **General Procedure B**, using 2,2'-(4,4-dimethylpentane-1,2-diyl)bis(4,4,5,5-tetramethyl-1,3,2-dioxaborolane) (**1a**) (70.5 mg, 0.2 mmol, 1.0 equiv.), PhLi (1.9 M in dibutyl ether, 0.12 mL, 0.22 mmol, 1.1 equiv.), 1-iodo-4-methoxybenzene (**5m**) (70.2 mg, 0.30 mmol, 1.5 equiv.), 4-CzIPN (7.9 mg, 0.01 mmol, 5.0 mol %), and Ni(TMHD)$_2$ (8.5 mg, 0.02 mmol, 10.0 mol %). Purification by flash column chromatography (95:5 pentane/E$_2$tO) gave the title compound **6am** (38 mg, 57%) as a colorless oil.

**TLC**: $R_f$ = 0.23 (95:5 pentane/E$_2$tO, CAM stain)

**NMR Spectroscopy (*see spectra*):**

$^{1}$**H NMR** (400 MHz, CDCl$_3$): $\delta_H$ 7.13 (d, $J$ = 8.7 Hz, 2H), 6.77 (d, $J$ = 8.6 Hz, 2H), 3.76 (s, 3H), 2.99 – 2.89 (m, 1H), 1.69 (dd, $J$ = 14.0, 9.6 Hz, 1H), 1.50 (dd, $J$ = 14.0, 3.5 Hz, 1H), 1.16 – 1.01 (m, 14H), 0.74 (s, 9H) ppm;

$^{13}$**C NMR** (101 MHz, CDCl$_3$): $\delta_C$ 157.7, 141.4, 128.6, 113.5, 83.0, 55.4, 53.3, 37.7, 31.5, 30.4, 24.9, 24.8. ppm. The carbon attached to boron was not observed due to quadrupolar relaxation;

$^{11}$**B NMR** (128 MHz, CDCl$_3$): $\delta_B$ 30.93 ppm.

**IR** (film): $\nu_{max}$ 2905, 1737, 1611, 1511, 1466, 1365, 1322, 1245, 1145, 1038, 968, 830 cm$^{-1}$.

**HRMS** (EI$^+$): m/z calculated for $C_{20}H_{33}BO_3$ [M]$^+$, 332.2517; found, 332.2513.

**2-{4,4-Dimethyl-2-[4-(methylthio)phenyl]pentyl}-4,4,5,5-tetramethyl-1,3,2-dioxaborolane (6an)**

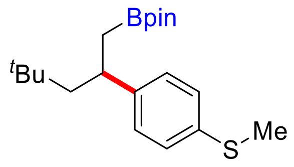


**6an**

Prepared following **General Procedure B**, using 2,2'-(4,4-dimethylpentane-1,2-diyl)bis(4,4,5,5-tetramethyl-1,3,2-dioxaborolane) (**1a**) (70.5 mg, 0.2 mmol, 1.0 equiv.), PhLi (1.9 M in dibutyl ether, 0.12 mL, 0.22 mmol, 1.1 equiv.), (4-iodophenyl)(methyl)sulfane (**5n**) (75.1 mg, 0.30 mmol, 1.5 equiv.), 4-CzIPN (7.9 mg, 0.01 mmol, 5.0 mol %), and Ni(TMHD)$_2$ (8.5 mg, 0.02 mmol, 10.0 mol %). Purification by flash column chromatography (95:5 pentane/E$_2$tO) gave the title compound **6an** (36 mg, 52%) as a colorless oil.

**TLC**: $R_f$ = 0.47 (95:5 pentane/E$_2$tO, CAM stain)

**NMR Spectroscopy (*see spectra*):**

**$^{1}$H NMR** (400 MHz, $CDCl_3$): $\delta_H$ 7.15 (s, 4H), 3.00 – 2.88 (m, 1H), 2.44 (s, 3H), 1.71 (dd, $J$ = 14.0, 9.5 Hz, 1H), 1.51 (dd, $J$ = 14.0, 3.4 Hz, 1H), 1.16 – 1.13 (m, 1H), 1.11 (s, 6H), 1.08 (s, 6H), 1.05 (dd, $J$ = 15.2, 8.9 Hz, 1H), 0.75 (s, 9H) ppm;

**$^{13}$C NMR** (101 MHz, $CDCl_3$): $\delta_C$ 146.6, 134.8, 128.3, 127.3, 83.1, 53.1, 38.1, 31.5, 30.4, 24.9, 24.8, 16.7 ppm. The carbon attached to boron was not observed due to quadrupolar relaxation;

**$^{11}$B NMR** (128 MHz, $CDCl_3$): $\delta_B$ 32.93 ppm.

**IR** (film): $\nu_{max}$ 2951, 1738, 1467, 1365, 1321, 1217, 1145, 1094, 968, 848, 818 cm$^{-1}$.

**HRMS** (EI$^+$): m/z calculated for $C_{20}H_{33}BSO_2$ [M]$^+$, 348.2289; found, 348.2284.

**1-{4-[4,4-Dimethyl-1-(4,4,5,5-tetramethyl-1,3,2-dioxaborolan-2-yl)pentan-2-yl]phenyl}-1*H*-pyrrole (6ao)**

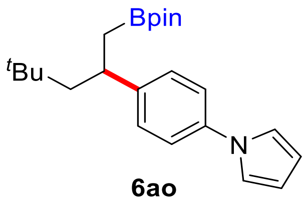


**6ao**

Prepared following **General Procedure B**, using 2,2'-(4,4-dimethylpentane-1,2-diyl)bis(4,4,5,5-tetramethyl-1,3,2-dioxaborolane) (**1a**) (70.5 mg, 0.2 mmol, 1.0 equiv.), PhLi (1.9 M in dibutyl ether, 0.12 mL, 0.22 mmol, 1.1 equiv.), 1-(4-iodophenyl)-1*H*-pyrrole (**5o**) (80.7 mg, 0.30 mmol, 1.5 equiv.), 4-CzIPN (7.9 mg, 0.01 mmol, 5.0 mol %), and Ni(TMHD)$_2$ (8.5 mg, 0.02 mmol, 10.0 mol %). Purification by flash column chromatography (91:9 pentane/$Et_2O$) gave the title compound **6ao** (23 mg, 31%) as a white solid.

**TLC**: $R_f$ = 0.37 (91/9 *n*-hexane/EtOAc, CAM stain)

**NMR Spectroscopy (*see spectra*):**

**$^{1}$H NMR** (400 MHz, $CDCl_3$): $\delta_H$ 7.30 – 7.25 (m, 4H), 7.07 – 7.04 (m, 2H), 6.34 – 6.31 (m, 2H), 3.06 – 2.97 (tdd, $J$ = 9.2, 7.1, 3.5, 1H), 1.75 (dd, $J$ = 14.1, 9.5 Hz, 1H), 1.59 – 1.52 (dd, $J$ = 14.1, 3.5 Hz,1H), 1.21 (dd, $J$ = 15.0, 6.9 Hz, 1H), 1.17 – 1.13 (m, 1H), 1.11 (s, 6H), 1.08 (s, 6H), 0.78 (s, 9H) ppm;

**$^{13}$C NMR** (101 MHz, $CDCl_3$): $\delta_C$ 146.8, 138.6, 128.8, 120.3, 119.4, 110.1, 83.1, 53.3, 38.0, 31.6, 30.4, 24.9, 24.8 ppm. The carbon attached to boron was not observed due to quadrupolar relaxation;

**$^{11}$B NMR** (128 MHz, $CDCl_3$): $\delta_B$ 33.35 ppm.

**IR** (film): $\nu_{max}$ 2950, 1738, 1612, 1520, 1481, 1365, 1328, 1144, 1071, 1021, 967, 924, 849, 723 cm$^{-1}$.

**HRMS** (EI$^+$): m/z calcd for $C_{23}H_{34}O_2NB$ [M]$^+$: 367.2677, found: 367.2672.

**3-[4,4-Dimethyl-1-(4,4,5,5-tetramethyl-1,3,2-dioxaborolan-2-yl)pentan-2-yl]benzonitrile (6ap)**

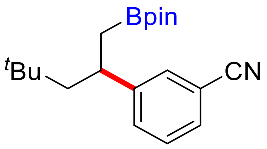


**6ap**

Prepared following **General Procedure B**, using 2,2'-(4,4-dimethylpentane-1,2-diyl)bis(4,4,5,5-tetramethyl-1,3,2-dioxaborolane) (**1a**) (70.5 mg, 0.2 mmol, 1.0 equiv.), PhLi (1.9 M in dibutyl ether, 0.12 mL, 0.22 mmol, 1.1 equiv.), 3-bromobenzonitrile (**5p**) (54.6 mg, 0.30 mmol, 1.5 equiv.), 4-CzIPN (7.9 mg, 0.01 mmol, 5.0 mol %), and $Ni(TMHD)_2$ (8.5 mg, 0.02 mmol, 10.0 mol %). Purification by flash column chromatography (91:9 pentane/$Et_2O$) gave the title compound **6ap** (27.5 mg, 42%) as a colorless oil.

**TLC**: $R_f$ = 0.27 (91/9 *n*-hexane/EtOAc, CAM stain)

**NMR Spectroscopy (*see spectra*):**

$^{1}$**H NMR** (400 MHz, $CDCl_3$): $\delta_H$ 7.53 (t, *J* = 1.7 Hz, 1H), δ 7.47 (dt, *J* = 7.8, 1.6 Hz, 1H), 7.42 (dt, *J* = 7.7, 1.4 Hz, 1H), 7.33 (t, *J* = 7.7 Hz, 1H), 3.01 (tdd, *J* = 9.6, 6.8, 3.4 Hz, 1H), 1.72 (dd, *J* = 14.2, 9.5 Hz, 1H), 1.55 (dd, *J* = 14.2, 3.5 Hz, 1H), 1.15 (dd, *J* = 15.5, 6.8 Hz, 1H), 1.09 (s, 6H), 1.08 (s, 6H), 1.07 – 1.01 (m, 1H), 0.74 (s, 9H) ppm;

$^{13}$**C NMR** (101 MHz, $CDCl_3$): $\delta_C$ 150.8, 132.4, 131.7, 129.5, 129.0, 119.4, 112.1, 83.2, 52.8, 38.3, 31.5, 30.3, 24.8, 24.8 ppm. The carbon attached to boron was not observed due to quadrupolar relaxation;

$^{11}$**B NMR** (128 MHz, $CDCl_3$): $\delta_B$ 33.05 ppm.

**IR** (film): $\nu_{max}$ 2930, 2229, 1738, 1468, 1365, 1322, 1214, 1143, 967, 846, 796, 696 $cm^{-1}$.

**HRMS** ($EI^+$) m/z calcd for $C_{20}H_{30}NBO_2$ $[M]^+$, 327.2364; found, 327.2364.

**4,4-Dimethyl-2-[3-(trifluoromethyl)phenyl]pentan-1-ol (6aq)**

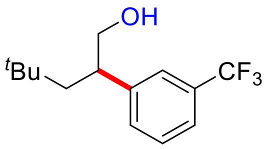


**6aq**

Prepared following **General Procedure B**, using 2,2'-(4,4-dimethylpentane-1,2-diyl)bis(4,4,5,5-tetramethyl-1,3,2-dioxaborolane) (**1a**) (70.5 mg, 0.2 mmol, 1.0 equiv.), PhLi (1.9 M in dibutyl ether, 0.12 mL, 0.22 mmol, 1.1 equiv.), 1-iodo-3-(trifluoromethyl)benzene (**5q**) (81.6 mg, 0.30 mmol, 1.5 equiv.), 4-CzIPN (7.9 mg, 0.01 mmol, 5.0 mol %), and $Ni(TMHD)_2$ (8.5 mg, 0.02 mmol, 10.0 mol %). The crude material was oxidized following the procedure mentioned above. Purification by flash column chromatography (80:20 *n*-hexane/EtOAc) gave the title compound **6aq** (26 mg, 50%) as a colorless oil.

**TLC**: $R_f$ = 0.22 (80:20 *n*-hexane/EtOAc, $KMnO_4$ stain)

**NMR Spectroscopy (*see spectra*):**

**$^1$H NMR** (400 MHz, $CDCl_3$): $\delta_H$ 7.52 – 7.46 (m, 2H), 7.46 – 7.41 (m, 2H), 3.70 (dd, $J$ = 10.7, 5.9 Hz, 1H), 3.63 (dd, $J$ = 10.7, 8.2 Hz, 1H), 2.95 (tdd, $J$ = 8.2, 5.9, 3.6 Hz, 1H), 1.66 (dd, $J$ = 14.1, 8.3 Hz, 1H), 1.58 (dd, $J$ = 14.1, 3.6 Hz, 1H), 1.38 (brs, 1H), 0.82 (s, 9H) ppm;

**$^{13}$C NMR** (101 MHz, $CDCl_3$): $\delta_C$ 145.7, 131.9 (q, $^4J_{C\text{-}F}$ = 1.0 Hz), 131.1 (q, $^2J_{C\text{-}F}$ = 32.0 Hz), 129.1, 125.0 (q, $^3J_{C\text{-}F}$ = 3.8 Hz), 124.3 (d, $^1J_{C\text{-}F}$ = 272.4 Hz), 123.6 (q, $^3J_{C\text{-}F}$ = 3.9 Hz), 69.0, 45.8, 45.4, 31.3, 30.2 ppm;

**$^{19}$F NMR** (377 MHz, $CDCl_3$) $\delta_F$ = -62.53 ppm.

**IR** (film): $\nu_{max}$ 3329, 2955, 1738, 1476, 1449, 1366, 1324, 1161, 1123, 1073, 1033, 801, 704 cm$^{-1}$.

**HRMS** (ESI$^+$) m/z calcd for $C_{14}H_{19}F_3NaO$ [M+Na]$^+$, 283.1280; found, 283.1280.

**2-{2-[3,5-Bis(trifluoromethyl)phenyl]-4,4-dimethylpentyl}-4,4,5,5-tetramethyl-1,3,2-dioxaborolane (6ar)**

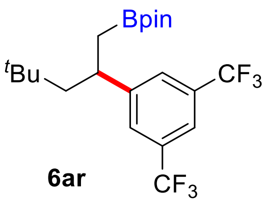


**6ar**

Prepared following **General Procedure B**, using 2,2'-(4,4-dimethylpentane-1,2-diyl)bis(4,4,5,5-tetramethyl-1,3,2-dioxaborolane) (**1a**) (70.5 mg, 0.2 mmol, 1.0 equiv.), PhLi (1.9 M in dibutyl ether, 0.12 mL, 0.22 mmol, 1.1 equiv.), 1-bromo-3,5-bis(trifluoromethyl)benzene (**5r**) (87.9 mg, 0.30 mmol, 1.5 equiv.), 4-CzIPN (7.9 mg, 0.01 mmol, 5.0 mol %), and $Ni(TMHD)_2$ (8.5 mg, 0.02 mmol, 10.0 mol %). Purification by flash column chromatography (67/33 pentane/toluene) gave the title compound **6ar** (57 mg, 65%) as a colorless oil.

**TLC**: $R_f$ = 0.25 (95:5 *n*-hexane/EtOAc, CAM stain)

**NMR Spectroscopy (*see spectra*):**

**$^1$H NMR** (400 MHz, $CDCl_3$): $\delta_H$ 7.69 (s, 2H), 7.65 (s, 1H), 3.13 (tdd, $J$ = 9.5, 6.4, 3.7 Hz, 1H), 1.77 (dd, $J$ = 14.2, 9.2 Hz, 1H), 1.61 (dd, $J$ = 14.2, 3.7 Hz, 1H), 1.20 (dd, $J$ = 15.7, 6.4 Hz, 1H), 1.10 (dd, $J$ = 9.2, 6.4 Hz, 1H), 1.08 (s, 6H), 1.07 (s, 6H), 0.77 (s, 9H) ppm;

**$^{13}$C NMR** (101 MHz, $CDCl_3$): $\delta_C$ 151.9, 131.3 (q, $^2J_{C\text{-}F}$ = 32.9 Hz), 128.2 (q, $^3J_{C\text{-}F}$ = 2.5 Hz), 123.69 (d, $^1J_{C\text{-}F}$ = 272.5 Hz), 119.76 (q, $^3J_{C\text{-}F}$ = 3.9 Hz), 119.7, 83.3, 52.8, 38.5, 31.6, 30.4, 24.8, 24.7 ppm. The carbon attached to boron was not observed due to quadrupolar relaxation;

**$^{19}$F NMR** (377 MHz, $CDCl_3$): $\delta_F$ -62.82 ppm;

**$^{11}$B NMR** (128 MHz, $CDCl_3$): $\delta_B$ 33.36 ppm.

**IR** (film): $\nu_{max}$ 2970, 1739, 1365, 1277, 1217, 1169, 1133, 845, 707, 683 cm$^{-1}$.

**HRMS** (EI$^+$): m/z calculated for $C_{21}H_{29}F_6BO_2$ [M]$^+$, 438.2159; found, 438.2154.

**1-{3-[4,4-Dimethyl-1-(4,4,5,5-tetramethyl-1,3,2-dioxaborolan-2-yl)pentan-2-yl]phenyl}ethan-1-one (6as)**

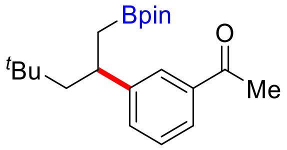


**6as**

Prepared following **General Procedure B**, using 2,2'-(4,4-dimethylpentane-1,2-diyl)bis(4,4,5,5-tetramethyl-1,3,2-dioxaborolane) (**1a**) (70.5 mg, 0.2 mmol, 1.0 equiv.), PhLi (1.9 M in dibutyl ether, 0.12 mL, 0.22 mmol, 1.1 equiv.), 1-(3-bromophenyl)ethan-1-one (**5s**) (59.7 mg, 0.30 mmol, 1.5 equiv.), 4-CzIPN (7.9 mg, 0.01 mmol, 5.0 mol %), and Ni(TMHD)$_2$ (8.5 mg, 0.02 mmol, 10.0 mol %). Purification by flash column chromatography (88:12 pentane/$Et_2O$) gave the title compound **6as** (32 mg, 46%) as a colorless oil.

**TLC**: $R_f$ = 0.18 (91:9 *n*-hexane/EtOAc, CAM stain)

**NMR Spectroscopy (*see spectra*):**

$^1$**H NMR** (400 MHz, $CDCl_3$): $\delta_H$ 7.83 (t, *J* = 1.8 Hz, 1H), 7.72 (dt, *J* = 7.7, 1.5 Hz, 1H), 7.44 (dt, *J* = 7.6, 1.5 Hz, 1H), 7.32 (t, *J* = 7.6 Hz, 1H), 3.10 – 3.01 (tdd, *J* = 9.5, 7.0, 3.4 Hz, 1H), 2.58 (s, 3H), 1.78 (dd, *J* = 14.1, 9.5 Hz, 1H), 1.55 (dd, *J* = 14.1, 3.5 Hz, 1H), 1.16 (dd, *J* = 15.3, 7.2 Hz, 1H), 1.10 (d, *J* = 8.7 Hz, 1H), 1.08 (s, 6H), 1.07 (s, 6H), 0.74 (s, 9H) ppm;

$^{13}$**C NMR** (101 MHz, $CDCl_3$): $\delta_C$ 198.4, 149.8, 137.0, 132.5, 128.3, 127.8, 125.8, 83.0, 52.8, 38.4, 31.4, 30.3, 26.7, 24.8, 24.7 ppm. The carbon attached to boron was not observed due to quadrupolar relaxation;

$^{11}$**B NMR** (128 MHz, $CDCl_3$): $\delta_B$ 33.17 ppm.

**IR** (film): $\nu_{max}$ 2951, 1738, 1686, 1599,1583, 1438, 1362, 1321, 1271, 1215, 1143, 967, 907, 698 $cm^{-1}$.

**HRMS** ($EI^+$): m/z calculated for $C_{21}H_{32}BO_3$ $[M]^+$, 344.2517; found, 344.2514.

**Methyl 3-[4,4-dimethyl-1-(4,4,5,5-tetramethyl-1,3,2-dioxaborolan-2-yl)pentan-2-yl]benzoate (6at)**

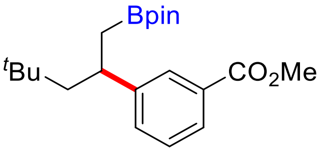


**6at**

Prepared following **General Procedure B**, using 2,2'-(4,4-dimethylpentane-1,2-diyl)bis(4,4,5,5-tetramethyl-1,3,2-dioxaborolane) (**1a**) (70.5 mg, 0.2 mmol, 1.0 equiv.), PhLi (1.9 M in dibutyl ether, 0.12 mL, 0.22 mmol, 1.1 equiv.), ethyl 3-iodobenzoate (**5t**) (82.8 mg, 0.30 mmol, 1.5 equiv.), 4-CzIPN (7.9 mg, 0.01 mmol, 5.0 mol %), and Ni(TMHD)$_2$ (8.5 mg, 0.02 mmol, 10.0 mol %). Purification by flash column chromatography (91:9 pentane/$Et_2O$) gave the title compound **6at** (40 mg, 55%) as a colorless oil.

**TLC**: $R_f$ = 0.27 (91:9 *n*-hexane/EtOAc, CAM stain)

**NMR Spectroscopy (*see spectra*):**

**$^{1}$H NMR** (400 MHz, $CDCl_3$): $\delta_H$ 7.92 (t, $J$ = 1.8 Hz, 1H), 7.80 (dt, $J$ = 7.7, 1.4 Hz, 1H), 7.43 (dt, $J$ = 7.7, 1.5 Hz, 1H), 7.30 (t, $J$ = 7.7 Hz, 1H), 3.90 (s, 3H), 3.09 – 3.00 (tdd, $J$ = 9.6, 7.1, 3.4 Hz, 1H), 1.79 (dd, $J$ = 14.1, 9.6 Hz, 1H), 1.55 (dd, $J$ = 14.1, 3.4 Hz, 1H), 1.16 (dd, $J$ = 15.3, 7.1 Hz, 1H), 1.11 – 1.05 (m, 13H), 0.74 (s, 9H) ppm;

**$^{13}$C NMR** (101 MHz, $CDCl_3$): $\delta_C$ 167.5, 149.7, 132.5, 123.0, 129.1, 128.2, 127.1, 83.1, 52.9, 52.1, 38.5, 31.6, 30.4, 24.9, 24.8 ppm. The carbon attached to boron was not observed due to quadrupolar relaxation;

**$^{11}$B NMR** (128 MHz, $CDCl_3$): $\delta_B$ 33.36 ppm.

**IR** (film): $\nu_{max}$ 2951, 1724, 1434, 1365, 1321, 1281, 1215, 1144, 1108, 968, 847, 757 cm$^{-1}$.

**HRMS** (EI$^+$): m/z calculated for $C_{21}H_{33}BO_4$ [M]$^+$, 360.2466; found, 360.2463.

**2-(3-Methoxyphenyl)-4,4-dimethylpentan-1-ol (6au)**

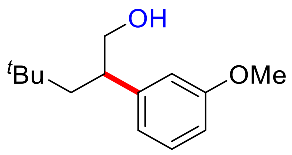


**6au**

Prepared following **General Procedure B**, using 2,2'-(4,4-dimethylpentane-1,2-diyl)bis(4,4,5,5-tetramethyl-1,3,2-dioxaborolane) (**1a**) (70.5 mg, 0.2 mmol, 1.0 equiv.), PhLi (1.9 M in dibutyl ether, 0.12 mL, 0.22 mmol, 1.1 equiv.), 1-iodo-3-methoxybenzene (**5u**) (46.8 mg, 0.30 mmol, 1.5 equiv.), 4-CzIPN (7.9 mg, 0.01 mmol, 5.0 mol %), and Ni(TMHD)$_2$ (8.5 mg, 0.02 mmol, 10.0 mol %). The crude material was oxidized following the procedure mentioned above. Purification by flash column chromatography (83/17 DCM /$Et_2O$) gave the title compound **6au** (25 mg, 57%) as a colorless oil.

**TLC**: $R_f$ = 0.70 (83/17 DCM /$Et_2O$, CAM stain)

**NMR Spectroscopy (*see spectra*):**

**$^{1}$H NMR** (400 MHz, $CDCl_3$): $\delta_H$ 7.23 (t, $J$ = 7.8 Hz, 1H), 6.83 (d, $J$ = 7.6 Hz, 1H), 6.80 – 6.73 (m, 2H), 3.80 (s, 3H), 3.69 – 3.62 (m, 1H), 3.62 – 3.54 (m, 1H), 2.90 – 2.81 (tdd, $J$ = 8.3, 5.9, 3.5 Hz, 1H), 1.69 – 1.61 (dd, $J$ =14.1, 8.3 Hz 1H), 1.49 (dd, $J$ = 14.1, 3.5 Hz, 1H), 1.32 (brs, 1H), 0.82 (s, 9H) ppm;

**$^{13}$C NMR** (101 MHz, $CDCl_3$): $\delta_C$ 156.0, 146.1, 129.7, 120.8, 114.4, 111.7, 69.1, 55.3, 45.9, 45.6, 31.3, 30.1 ppm.

**IR** (film): $\nu_{max}$ 3379, 2951, 1734, 1600, 1584, 1487, 1466, 1487, 1262, 1151, 1034, 864, 779, 702 cm$^{-1}$.

**HRMS** (EI$^+$): m/z calculated for $C_{14}H_{22}O_2$ [M]$^+$, 222.1614; found, 222.1613.

**4-[4,4-Dimethyl-1-(4,4,5,5-tetramethyl-1,3,2-dioxaborolan-2-yl)pentan-2-yl]-3-methylbenzonitrile (6av)**

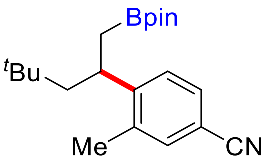


**6av**

Prepared following **General Procedure B**, using 2,2'-(4,4-dimethylpentane-1,2-diyl)bis(4,4,5,5-tetramethyl-1,3,2-dioxaborolane) (**1a**) (70.5 mg, 0.2 mmol, 1.0 equiv.), PhLi (1.9 M in dibutyl ether, 0.12 mL, 0.22 mmol, 1.1 equiv.), 4-bromo-3-methylbenzonitrile (**5v**) (58.8 mg, 0.30 mmol, 1.5 equiv.), 4-CzIPN (7.9 mg, 0.01 mmol, 5.0 mol %), and Ni(TMHD)$_2$ (8.5 mg, 0.02 mmol, 10.0 mol %). Purification by flash column chromatography (91:9 pentane/Et$_2$O) gave the title compound **6av** (21.3 mg, 31%) as a colorless oil.

**TLC**: $R_f$ = 0.37 (91:9 *n*-hexane/EtOAc, CAM stain)

**NMR Spectroscopy (*see spectra*):**

$^1$**H NMR** (400 MHz, CDCl$_3$): $\delta_H$ 7.41 (dd, *J* = 8.1, 1.8 Hz, 1H), 7.38 – 7.35 (m, 1H), 7.32 (d, *J* = 8.1 Hz, 1H), 3.26 (tdd, *J* = 8.8, 6.7, 4.1 Hz, 1H), 2.45 (s, 3H), 1.73 (dd, *J* = 14.2, 8.6 Hz, 1H), 1.55 (dd, *J* = 14.2, 4.1 Hz, 1H), 1.16 (dd, *J* = 15.3, 6.9 Hz, 1H), 1.09 – 0.99 (m, 13H), 0.77 (s, 9H) ppm;

$^{13}$**C NMR** (101 MHz, CDCl$_3$): $\delta_C$ 153.8, 136.6, 133.7, 129.9, 127.4, 119.6, 108.9, 83.2, 53.2, 32.8, 31.6, 30.4, 24.9, 24.7, 20.1 ppm. The carbon attached to boron was not observed due to quadrupolar relaxation;

$^{11}$**B NMR** (128 MHz, CDCl$_3$): $\delta_B$ 32.78 ppm.

**IR** (film): $\nu_{max}$ 2957, 2228, 1739, 1605, 1468, 1365, 1323, 1216, 1144, 967, 848, 604 cm$^{-1}$.

**HRMS** (EI$^+$): m/z calculated for $C_{21}H_{32}BNO_2$ [M]$^+$, 341.2521; found, 341.2518.

**2-[4,4-Dimethyl-1-(4,4,5,5-tetramethyl-1,3,2-dioxaborolan-2-yl)pentan-2-yl]benzonitrile (6aw)**

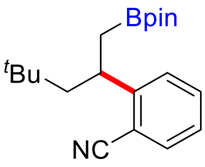


**6aw**

Prepared following **General Procedure B**, using 2,2'-(4,4-dimethylpentane-1,2-diyl)bis(4,4,5,5-tetramethyl-1,3,2-dioxaborolane) (**1a**) (70.5 mg, 0.2 mmol, 1.0 equiv.), PhLi (1.9 M in dibutyl ether, 0.12 mL, 0.22 mmol, 1.1 equiv.), 2-bromobenzonitrile (**5w**) (54.6 mg, 0.30 mmol, 1.5 equiv.), 4-CzIPN (7.9 mg, 0.01 mmol, 5.0 mol %), and Ni(TMHD)$_2$ (8.5 mg, 0.02 mmol, 10.0 mol %). Purification by flash column chromatography (91:9 pentane/Et$_2$O) gave the title compound **6aw** (37 mg, 56%) as a colorless oil.

**TLC**: $R_f$ = 0.35 (91:9 *n*-hexane/EtOAc, CAM stain)

**NMR Spectroscopy (*see spectra*):**

**$^{1}$H NMR** (400 MHz, $CDCl_3$): $\delta_H$ 7.54 (dd, *J* = 7.7, 1.4 Hz, 1H), 7.48 (td, *J* = 7.7, 1.4 Hz, 1H), 7.39 (dd, *J* = 8.0, 1.3 Hz, 1H), 7.21 (td, *J* = 7.5, 1.3 Hz, 1H), 3.47 (tdd, *J* = 9.5, 6.7, 3.5 Hz, 1H), 1.80 (dd, *J* = 14.2, 9.3 Hz, 1H), 1.63 (dd, *J* = 14.2, 3.5 Hz, 1H), 1.26 – 1.19 (m, 1H), 1.14 (dd, *J* = 15.6, 8.9 Hz, 1H), 1.09 (s, 6H), 1.08 (s, 6H), 0.79 (s, 9H) ppm;

**$^{13}$C NMR** (101 MHz, $CDCl_3$): $\delta_C$ 153.3, 132.8, 132.7, 127.9, 126.2, 118.8, 112.7, 83.2, 53.1, 36.4, 31.5, 30.3, 24.8, 24.8 ppm. The carbon attached to boron was not observed due to quadrupolar relaxation;

**$^{11}$B NMR** (128 MHz, $CDCl_3$): $\delta_B$ 32.96 ppm.

**IR** (film): $\nu_{max}$ 2951, 2224, 1738, 1477, 1365, 1326, 1144, 968, 847, 763, 551 cm$^{-1}$.

**HRMS** (ESI$^+$): m/z calculated for $C_{20}H_{30}BNNaO_2$ [M+Na]$^+$, 350.2265; found, 350.2282.

### 5-[4,4-Dimethyl-1-(4,4,5,5-tetramethyl-1,3,2-dioxaborolan-2-yl)pentan-2-yl]-2-methylpyridine (6ax)

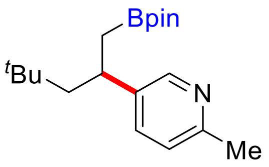


**6ax**

Prepared following **General Procedure B**, using 2,2'-(4,4-dimethylpentane-1,2-diyl)bis(4,4,5,5-tetramethyl-1,3,2-dioxaborolane) (**1a**) (70.5 mg, 0.2 mmol, 1.0 equiv.), PhLi (1.9 M in dibutyl ether, 0.12 mL, 0.22 mmol, 1.1 equiv.), 5-bromo-2-methylpyridine (**5x**) (51.6 mg, 0.30 mmol, 1.5 equiv.), 4-CzIPN (7.9 mg, 0.01 mmol, 5.0 mol %), and Ni(TMHD)$_2$ (8.5 mg, 0.02 mmol, 10.0 mol %). Purification by flash column chromatography (67:33 *n*-hexane/EtOAc) gave the title compound **6ax** (28 mg, 44%) as a colorless oil.

**TLC**: $R_f$ = 0.50 (50:50 *n*-hexane/EtOAc, CAM stain)

**NMR Spectroscopy (*see spectra*):**

**$^{1}$H NMR** (400 MHz, $CDCl_3$): $\delta_H$ 8.35 (dd, *J* = 2.2 Hz, 1H), 7.42 (dd, *J* = 8.0, 2.4 Hz, 1H), 7.03 (d, *J* = 8.0 Hz, 1H), 2.97 (tdd, *J* = 9.7, 7.1, 3.3 Hz, 1H), 2.48 (s, 3H), 1.71 (dd, *J* = 14.1, 9.7 Hz, 1H), 1.54 (dd, *J* = 14.1, 3.4 Hz, 1H), 1.14 (dd, *J* = 13.8, 3.4 Hz, 1H), 1.10 (s, 6H), 1.08 (s, 6H), 1.05 (dd, *J* =13.8, 9.7 Hz, 1H), 0.74 (s, 9H) ppm;

**$^{13}$C NMR** (101 MHz, $CDCl_3$): $\delta_C$ 155.6, 149.1, 141.1, 135.1, 122.9, 83.2, 52.8, 35.5, 31.5, 30.4, 24.9, 24.8, 24.1 ppm. The carbon attached to boron was not observed due to quadrupolar relaxation;

**$^{11}$B NMR** (128 MHz, $CDCl_3$): $\delta_B$ 33.21 ppm.

**IR** (film): $\nu_{max}$ 2952, 1738, 1600, 1488, 1363, 1321, 1217, 1144, 967, 848, 736 cm$^{-1}$.

**HRMS** (EI$^+$): m/z calculated for $C_{19}H_{32}BNO_2$ [M]$^+$, 317.2521; found, 317.2519.

**5-[4,4-Dimethyl-1-(4,4,5,5-tetramethyl-1,3,2-dioxaborolan-2-yl)pentan-2-yl]-2-(trifluoromethyl)pyridine (6ay)**

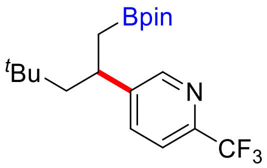


**6ay**

Prepared following **General Procedure B**, using 2,2'-(4,4-dimethylpentane-1,2-diyl)bis(4,4,5,5-tetramethyl-1,3,2-dioxaborolane) (**1a**) (70.5 mg, 0.2 mmol, 1.0 equiv.), PhLi (1.9 M in dibutyl ether, 0.12 mL, 0.22 mmol, 1.1 equiv.), 5-bromo-2-(trifluoromethyl)pyridine (**5y**) (67.8 mg, 0.30 mmol, 1.5 equiv.), 4-CzIPN (7.9 mg, 0.01 mmol, 5.0 mol %), and Ni(TMHD)$_2$ (8.5 mg, 0.02 mmol, 10.0 mol %). Purification by flash column chromatography (91:9 pentane/Et$_2$O) gave the title compound **6ay** (41 mg, 55%) as a colorless oil.

**TLC**: $R_f$ = 0.31 (91:9 *n*-hexane/EtOAc, CAM stain)

**NMR Spectroscopy (*see spectra*):**

$^1$**H NMR** (400 MHz, CDCl$_3$): $\delta_H$ 8.60 (d, $J$ = 2.2 Hz, 1H), 7.71 (dd, $J$ = 8.0, 2.2 Hz, 1H), 7.56 (d, $J$ = 8.0 Hz, 1H), 3.09 (tdd, $J$ = 9.4, 6.8, 3.4 Hz, 1H), 1.76 (dd, $J$ = 14.2, 9.4 Hz, 1H), 1.61 (dd, $J$ = 14.2, 3.4 Hz, 1H), 1.21 – 1.03 (m, 14H), 0.76 (s, 9H) ppm;

$^{13}$**C NMR** (101 MHz, CDCl$_3$): $\delta_C$ 150.2, 148.1, 145.8 (q, $^2J_{C\text{-}F}$ = 34.5 Hz), 135.9, 121.9 (q, $^1J_{C\text{-}F}$ = 273.8 Hz), 120.1 (q, $^3J_{C\text{-}F}$ = 2.8 Hz), 83.4, 52.7, 45.3, 35.8, 31.6, 30.4, 24.8 ppm. The carbon attached to boron was not observed due to quadrupolar relaxation;

$^{19}$**F NMR** (377 MHz, CDCl$_3$): $\delta_F$ -67.65 ppm;

$^{11}$**B NMR** (128 MHz, CDCl$_3$): $\delta_B$ 32.78 ppm.

**IR** (film): $\nu_{max}$ 2955, 1739, 1595, 1557, 1472, 1408, 1365, 1321, 1144, 1038, 967, 847, 803 cm$^{-1}$.

**HRMS** (EI$^+$): m/z calculated for $C_{19}H_{29}O_2NBF_3$ [M]$^+$, 371.2238; found, 371.2235.

**5-[4,4-Dimethyl-1-(4,4,5,5-tetramethyl-1,3,2-dioxaborolan-2-yl)pentan-2-yl]-2-methoxypyrimidine (6az)**

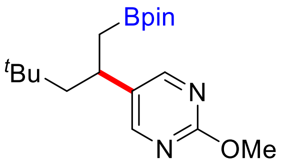


**6az**

Prepared following **General Procedure B**, using 2,2'-(4,4-dimethylpentane-1,2-diyl)bis(4,4,5,5-tetramethyl-1,3,2-dioxaborolane) (**1a**) (70.5 mg, 0.2 mmol, 1.0 equiv.), PhLi (1.9 M in dibutyl ether, 0.12 mL, 0.22 mmol, 1.1 equiv.), 5-iodo-2-methoxypyrimidine (**5z**) (70.8 mg, 0.30 mmol, 1.5 equiv.), 4-CzIPN (7.9 mg, 0.01 mmol, 5.0 mol %), and Ni(TMHD)$_2$ (8.5 mg, 0.02 mmol, 10.0 mol %). Purification by flash column chromatography (75:25

*n*-hexane/EtOAc) gave the title compound **6az** (35 mg, 52%) as a colorless oil.

**TLC**: $R_f$ = 0.45 (67:33 *n*-hexane/EtOAc, CAM stain)

**NMR Spectroscopy (*see spectra*):**

$^{1}$**H NMR** (400 MHz, $CDCl_3$): $\delta_H$ 8.37 (s, 2H), 3.96 (s, 3H), 2.93 (tdd, *J* = 9.7, 6.8, 3.4 Hz, 1H), 1.71 (dd, *J* = 14.1, 9.6 Hz, 1H), 1.57 (dd, *J* = 14.1, 3.4 Hz, 1H), 1.16 (dd, *J* = 14.8, 7.8 Hz, 1H), 1.12 (s, 12H), 1.06 (dd, *J* = 15.7, 9.0 Hz, 1H), 0.77 (s, 9H) ppm;

$^{13}$**C NMR** (101 MHz, $CDCl_3$): $\delta_C$ 164.3, 158.6, 134.9, 83.4, 54.8, 52.5, 33.0, 31.5, 30.5, 24.9, 24.8 ppm. The carbon attached to boron was not observed due to quadrupolar relaxation;

$^{11}$**B NMR** (128 MHz, $CDCl_3$): $\delta_B$ 33.13 ppm.

**IR** (film): $\nu_{max}$ 2952, 1739, 1588, 1550, 1445, 1364, 1321, 1272, 1144, 967, 847, 753 cm$^{-1}$.

**HRMS** ($EI^+$): m/z calculated for $C_{18}H_{31}BN_2O_3$ $[M]^+$, 334.2422; found, 334.2422.

**5-[4,4-Dimethyl-1-(4,4,5,5-tetramethyl-1,3,2-dioxaborolan-2-yl)pentan-2-yl]-2-methylpyrimidine (6aaa)**

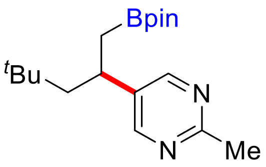


**6aaa**

Prepared following **General Procedure B**, using 2,2'-(4,4-dimethylpentane-1,2-diyl)bis(4,4,5,5-tetramethyl-1,3,2-dioxaborolane) (**1a**) (70.5 mg, 0.2 mmol, 1.0 equiv.), PhLi (1.9 M in dibutyl ether, 0.12 mL, 0.22 mmol, 1.1 equiv.), 5-iodo-2-methylpyrimidine (**5aa**) (66.0 mg, 0.30 mmol, 1.5 equiv.), 4-CzIPN (7.9 mg, 0.01 mmol, 5.0 mol %), and $Ni(TMHD)_2$ (8.5 mg, 0.02 mmol, 10.0 mol %). Purification by flash column chromatography (67:33 *n*-hexane/EtOAc) gave the title compound **6aaa** (31 mg, 49%) as a colorless oil.

**TLC**: $R_f$ = 0.28 (67:33 *n*-hexane/EtOAc, CAM stain)

**NMR Spectroscopy (*see spectra*):**

$^{1}$**H NMR** (400 MHz, $CDCl_3$): $\delta_H$ 8.50 (s, 2H), 2.94 (tdd, *J* = 9.2, 6.9, 3.4 Hz, 1H), 2.67 (s, 3H), 1.74 (dd, *J* = 14.2, 9.5 Hz, 1H), 1.59 (dd, *J* = 14.2, 3.4 Hz, 1H), 1.18 (dd, *J* = 15.7, 6.9 Hz, 1H), 1.11 (s, 12H), 1.08 (dd, *J* = 15.7, 8.8 Hz, 1H), 0.77 (s, 9H) ppm;

$^{13}$**C NMR** (101 MHz, $CDCl_3$): $\delta_C$ 165.5, 156.5, 138.4, 83.4, 52.4, 33.6, 31.6, 30.4, 25.7, 24.9, 24.8 ppm. The carbon attached to boron was not observed due to quadrupolar relaxation;

$^{11}$**B NMR** (128 MHz, $CDCl_3$): $\delta_B$ 32.96 ppm.

**IR** (film): $\nu_{max}$ 2952, 1739, 1469, 1365, 1337, 1176, 1135, 1086, 1026, 967, 846, 641 cm$^{-1}$.

**HRMS** ($EI^+$): m/z calculated for $C_{18}H_{31}O_2N_2B$ $[M]^+$, 318.2473; found, 318.2471.

**2-(2,3-Dihydrobenzofuran-5-yl)-4,4-dimethylpentan-1-ol (6aab)**

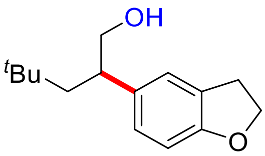

**6aab**

Prepared following **General Procedure B**, using 2,2'-(4,4-dimethylpentane-1,2-diyl)bis(4,4,5,5-tetramethyl-1,3,2-dioxaborolane) (**1a**) (70.5 mg, 0.2 mmol, 1.0 equiv.), PhLi (1.9 M in dibutyl ether, 0.12 mL, 0.22 mmol, 1.1 equiv.), 5-iodo-2,3-dihydrobenzofuran (**5ab**) (73.8 mg, 0.30 mmol, 1.5 equiv.), 4-CzIPN (7.9 mg, 0.01 mmol, 5.0 mol %), and $Ni(TMHD)_2$ (8.5 mg, 0.02 mmol, 10.0 mol %). The crude material was oxidized following the procedure mentioned above. Purification by flash column chromatography (67:33 pentane/$Et_2O$) gave the title compound **6aab** (29 mg, 62%) as a colorless oil.

**TLC**: $R_f$ = 0.34 (67:33 *n*-hexane/EtOAc, $KMnO_4$ stain)

**NMR Spectroscopy (*see spectra*):**

$^{1}$**H NMR** (400 MHz, $CDCl_3$): $\delta_H$ 7.06 (d, *J* = 1.7 Hz, 1H), 6.95 (dd, *J* = 8.2, 2.1 Hz, 1H), 6.72 (d, *J* = 8.1 Hz, 1H), 4.55 (t, *J* = 8.7 Hz, 2H), 3.64 (ddd, *J* = 10.5, 8.7, 5.7 Hz, 1H), 3.53 (ddd, *J* = 10.5, 8.8, 3.9 Hz, 1H), 3.20 (t, *J* = 8.7 Hz, 2H), 2.81 (tdd, *J* = 8.9, 5.8, 3.5 Hz, 1H), 1.59 (dd, *J* = 14.0, 8.3 Hz, 1H), 1.47 (dd, *J* = 14.0, 3.6 Hz, 1H), 1.26 (brs, 1H), 0.82 (s, 9H) ppm;

$^{13}$**C NMR** (101 MHz, $CDCl_3$): $\delta_C$ 159.0, 136.1, 128.0, 127.5, 124.7, 109.4, 71.3, 69.3, 46.2, 44.9, 31.3, 30.2, 30.0.

**IR** (film): $\nu_{max}$ 3391, 2951, 2863, 1738, 1614, 1492, 1365, 1242, 1062, 1033, 984, 902, 816, 725 $cm^{-1}$.

**HRMS** ($EI^+$): m/z calculated for $C_{15}H_{22}O_2$ $[M]^+$, 234.1614; found, 234.1612.

**5-[4,4-Dimethyl-1-(4,4,5,5-tetramethyl-1,3,2-dioxaborolan-2-yl)pentan-2-yl]-2-methylbenzo[*d*]thiazole (6aac)**

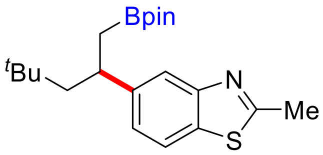

**6aac**

Prepared following **General Procedure B**, using 2,2'-(4,4-dimethylpentane-1,2-diyl)bis(4,4,5,5-tetramethyl-1,3,2-dioxaborolane) (**1a**) (70.5 mg, 0.2 mmol, 1.0 equiv.), PhLi (1.9 M in dibutyl ether, 0.12 mL, 0.22 mmol, 1.1 equiv.), 5-bromo-2-methylbenzo[*d*]thiazole (**5ac**) (68.5 mg, 0.30 mmol, 1.5 equiv.), 4-CzIPN (7.9 mg, 0.01 mmol, 5.0 mol %), and $Ni(TMHD)_2$ (8.5 mg, 0.02 mmol, 10.0 mol %). Purification by flash column chromatography (83:17 pentane/$Et_2O$) gave the title compound **6aac** (25 mg, 34%) as a colorless oil.

**TLC**: $R_f$ = 0.37 (75:25 *n*-hexane/EtOAc, CAM stain)

**NMR Spectroscopy (*see spectra*):**

**$^{1}$H NMR** (400 MHz, $CDCl_3$): $\delta_H$ 7.81 (d, $J$ = 1.7 Hz, 1H), 7.67 (d, $J$ = 8.2 Hz, 1H), 7.27 – 7.23 (m, 1H), 3.12 (tdd, $J$ = 10.9, 8.0, 3.2 Hz, 1H), 2.80 (s, 3H), 1.82 (dd, $J$ = 14.1, 9.8 Hz, 1H), 1.58 (dd, $J$ = 14.1, 3.2 Hz, 1H), 1.24 – 1.12 (m, 2H), 1.09 (s, 6H), 1.07 (s, 6H), 0.74 (d, $J$ = 0.8 Hz, 9H) ppm;

**$^{13}$C NMR** (101 MHz, $CDCl_3$): $\delta_C$ 166.8, 153.8, 147.7, 132.7, 125.0, 121.4, 120.9, 83.1, 53.1, 38.6, 31.6, 30.4, 24.9, 24.8, 20.3 ppm. The carbon attached to boron was not observed due to quadrupolar relaxation;

**$^{11}$B NMR** (128 MHz, $CDCl_3$): $\delta_B$ 33.13 ppm.

**IR** (film): $\nu_{max}$ 2951, 1739, 1457, 1365, 1321, 1217, 1166, 1145, 968, 877, 847, 644 cm$^{-1}$.

**HRMS** (EI$^+$): m/z calculated for $C_{21}H_{32}BNS$ [M]$^+$, 373.2241; found, 373.2239.

**(*Z*)-4,4,5,5-Tetramethyl-2-(2-neopentylpent-3-en-1-yl)-1,3,2-dioxaborolane (6aad)**

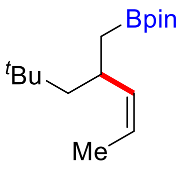


**6aad**

Prepared following **General Procedure B**, using 2,2'-(4,4-dimethylpentane-1,2-diyl)bis(4,4,5,5-tetramethyl-1,3,2-dioxaborolane) (**1a**) (70.5 mg, 0.2 mmol, 1.0 equiv.), PhLi (1.8 M in dibutyl ether, 0.12 mL, 0.22 mmol, 1.1 equiv.), (*Z*)-1-bromoprop-1-ene (36.3 mg, 0.30 mmol, 1.5 equiv.), 4-CzIPN (7.9 mg, 0.01 mmol, 5.0 mol %), and Ni(TMHD)$_2$ (8.5 mg, 0.02 mmol, 10.0 mol %). Purification by flash column chromatography (75:25 pentane/DCM) gave the title compound **6aad** (26 mg, 48%, >20:1 r.r) as a colorless oil.

**NOTE:** The product was formed as an 89:11 mixture of *Z* and *E* alkene isomers, which reflects the *Z*:*E* ratio of the 1-bromoprop-1-ene starting material.

$R_f$ = 0.50 (75:25 pentane/DCM, CAM stain)

**NMR Spectroscopy (*see spectra*)**

**$^{1}$H NMR** (400 MHz, $CDCl_3$) $\delta_H$ 5.26 – 5.20 (m, 2H), 2.82 – 2.69 (m, 1H), 1.65 (d, $J$ = 5.0 Hz, 3H), 1.29 – 1.24 (m, 2H), 1.23 (s, 6H), 1.22 (s, 6H), 0.87 (s, 9H), 0.84 (dd, $J$ = 14.9, 8.6 Hz, 1H), 0.75 (dd, $J$ = 14.9, 8.1 Hz, 1H) ppm;

**$^{13}$C NMR** (101 MHz, $CDCl_3$) $\delta_C$ 140.3, 119.7, 83.7, 53.3, 31.3, 30.4, 29.8, 27.6, 25.2, 24.9, 13.5 ppm. The carbon attached to boron was not observed due to quadrupolar relaxation.

$^{11}$B NMR (128 MHz, $CDCl_3$) $\delta_B$ 32.48 ppm.

**IR** (film): $\nu_{max}$ 2955, 2925, 2856, 1739, 1476, 1468, 1412, 1363, 1320, 1288, 1248, 1216, 1146, 969, 845, 729, 413 cm$^{-1}$.

**HRMS** (ESI$^+$): m/z calculated for $C_{16}H_{31}O_2B$ [M]$^+$, 266.2412; found, 266.2412.

**4-[1-(4,4,5,5-Tetramethyl-1,3,2-dioxaborolan-2-yl)-3-(trimethylsilyl)propan-2-yl]benzonitrile (6ba)**

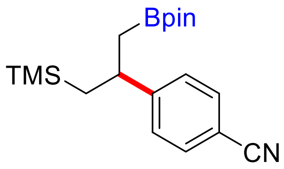


**6ba**

Prepared following **General Procedure B**, using [2,3-bis(4,4,5,5-tetramethyl-1,3,2-dioxaborolan-2-yl)propyl]trimethylsilane (**1b**) (73.7 mg, 0.2 mmol, 1.0 equiv.), PhLi (1.9 M in dibutyl ether, 0.12 mL, 0.22 mmol, 1.1 equiv.), 4-bromobenzonitrile (**5a**) (54.6 mg, 0.30 mmol, 1.5 equiv.), 4-CzIPN (7.9 mg, 0.01 mmol, 5.0 mol %), and Ni(TMHD)$_2$ (8.5 mg, 0.02 mmol, 10.0 mol %). Purification by flash column chromatography (91:9 pentane/Et$_2$O) gave the title compound **6ba** (22 mg, 32%) as a colorless oil.

Prepared following **General Procedure C**, using [2,3-bis(4,4,5,5-tetramethyl-1,3,2-dioxaborolan-2-yl)propyl]trimethylsilane (**1b**) (73.7 mg, 0.2 mmol, 1.0 equiv.), 4-bromo-*N,N*-dimethylaniline (44 mg, 0.22 mmol, 1.1 equiv.), *tert*-butyllithium (1.7 M in pentane, 260 µL, 0.44 mmol, 2.2 equiv.), 4-bromobenzonitrile (**5a**) (54.6 mg, 0.30 mmol, 1.5 equiv.), 4-CzIPN (7.9 mg, 0.01 mmol, 5.0 mol %), and Ni(TMHD)$_2$ (8.5 mg, 0.02 mmol, 10.0 mol %). Purification by flash column chromatography (91:9 pentane/Et$_2$O) gave the title compound **6ba** (48 mg, 70%) as a colorless oil.

**TLC**: $R_f$ = 0.30 (91:9 pentane/Et$_2$O, CAM stain)

**NMR Spectroscopy (*see spectra*):**

> $^{1}$**H NMR** (400 MHz, CDCl$_3$): $\delta_H$ 7.53 (d, $J$ = 8.4 Hz, 2H), 7.33 (d, $J$ = 8.3 Hz, 2H), 3.05 (tt, $J$ = 8.7, 7.0 Hz, 1H), 1.22 (dd, $J$ = 15.5, 6.8 Hz, 1H), 1.15 (dd, $J$ = 15.4, 9.0 Hz, 1H), 1.08 (s, 6H), 1.08 (s, 6H), 1.01 – 0.95 (m, 2H), -0.19 (s, 9H) ppm;
>
> $^{13}$**C NMR** (101 MHz, CDCl$_3$): $\delta_C$ 155.2, 132.2, 128.3, 119.4, 109.6, 83.2, 38.4, 28.2, 24.8, 24.8, -0.9 ppm. The carbon attached to boron was not observed due to quadrupolar relaxation;
>
> $^{11}$**B NMR** (128 MHz, CDCl$_3$): $\delta_B$ 31.91 ppm.

**IR** (film): $\nu_{max}$ 2978, 2227, 1738, 1607, 1370, 1326, 1248, 1144, 968, 864, 836 cm$^{-1}$.

**HRMS** (ESI$^+$): m/z calculated for $C_{19}H_{30}BNNaO_2Si$ [M+Na]$^+$, 366.2035; found, 366.2053.

**4-[1-Cyclohexyl-2-(4,4,5,5-tetramethyl-1,3,2-dioxaborolan-2-yl)ethyl]benzonitrile (6ca)**

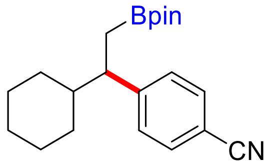


**6ca**

Prepared following **General Procedure B**, using 2,2'-(1-cyclohexylethane-1,2-diyl)bis(4,4,5,5-tetramethyl-1,3,2-dioxaborolane) (**1c**) (72.8 mg, 0.2 mmol, 1.0 equiv.), PhLi (1.9 M in dibutyl ether, 0.12 mL, 0.22 mmol, 1.1 equiv.), 4-bromobenzonitrile (**5a**) (54.6 mg, 0.30 mmol, 1.5 equiv.), 4-CzIPN (7.9 mg, 0.01 mmol, 5.0 mol %), and Ni(TMHD)$_2$ (8.5 mg, 0.02 mmol, 10.0 mol %). Purification by flash column chromatography (91:9 pentane/$Et_2O$) gave the title compound **6ca** (36 mg, 53%) as a colorless oil. The regioselectivity was determined by GC-Fid analysis from the crude reaction mixture to be 13/1.0 (*r.r.*) (**Figure S4**).

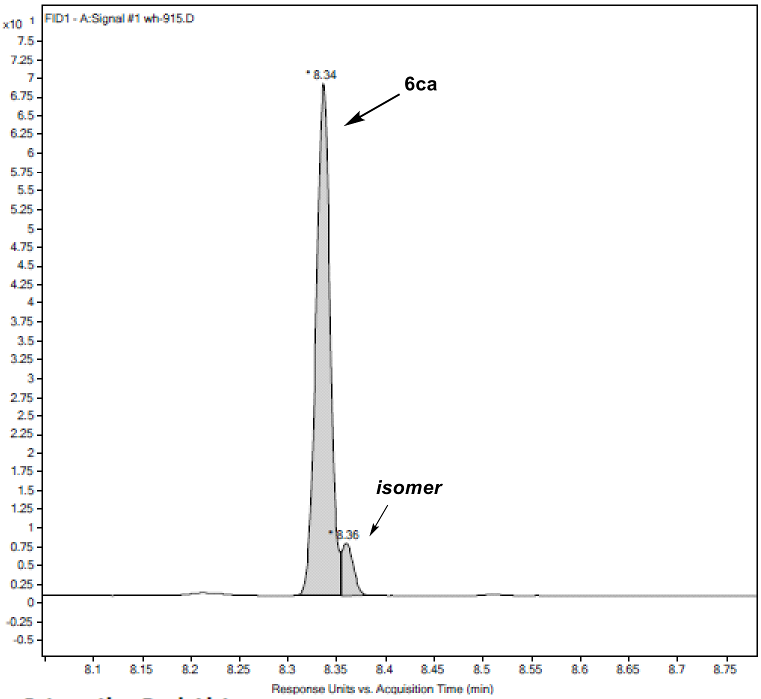


**Integration Peak List**

| Peak | Start | RT | End | Height | Area | Area % |
|---|---|---|---|---|---|---|
| 1 | 8.31 | 8.34 | 8.35 | 1165120.77 | 1190235.6 | 100 |
| 2 | 8.35 | 8.36 | 8.38 | 120031.11 | 97673.29 | 8.21 |

**Figure S4. The GC-Fid Result for the Regioselectivity of 6ca**

**TLC**: $R_f$ = 0.32 (91:9 *n*-hexane/EtOAc, CAM stain)

**NMR Spectroscopy (*see spectra*):**

**$^1$H NMR** (400 MHz, $CDCl_3$): $\delta_H$ 7.53 (d, *J* = 8.3 Hz, 2H), 7.26 (d, *J* = 8.3 Hz, 2H), 2.66 (ddd, *J* = 10.7, 7.7, 5.6 Hz, 1H), 1.85 (d, *J* = 12.4 Hz, 1H), 1.78 – 1.69 (m, 1H), 1.64 – 1.57 (m, 2H), 1.39 – 1.15 (m, 4H), 1.17 – 1.03 (m, 3H), 1.03 (s, 6H), 1.00 (s, 6H), 0.90 (tdd, *J* = 12.5, 11.2, 3.6 Hz, 1H), 0.82 – 0.68 (m, 1H) ppm;

**$^{13}$C NMR** (101 MHz, $CDCl_3$): $\delta_C$ 152.6, 131.8, 129.3, 119.4, 109.6, 83.2, 47.9, 45.0, 31.1, 30.9, 26.6, 26.5, 24.8, 24.6 ppm. The carbon attached to boron was not observed due to quadrupolar relaxation;

**$^{11}$B NMR** (128 MHz, $CDCl_3$): $\delta_B$ 33.49 ppm.

**IR** (film): $\nu_{max}$ 2977, 2926, 2852, 2227, 1738, 1607, 1449, 1365, 1325, 1217, 1144, 968, 847, 579 cm$^{-1}$.

**HRMS** (ESI$^+$): m/z calculated for $C_{21}H_{30}BNNaO_2$ [M+Na]$^+$, 362.2266; found, 362.2281.

### 4-(1-Hydroxy-4-methylpentan-2-yl)benzonitrile (6da)

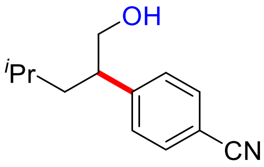


**6da**

Prepared following **General Procedure B**, using [2,2'-(4-methylpentane-1,2-diyl)bis(4,4,5,5-tetramethyl-1,3,2-dioxaborolane) (**1d**) (67.6 mg, 0.2 mmol, 1.0 equiv.), PhLi (1.9 M in dibutyl ether, 0.12 mL, 0.22 mmol, 1.1 equiv.), 4-bromobenzonitrile (**5a**) (54.6 mg, 0.30 mmol, 1.5 equiv.), 4-CzIPN (7.9 mg, 0.01 mmol, 5.0 mol %), and $Ni(TMHD)_2$ (8.5 mg, 0.02 mmol, 10.0 mol %). The yield and regioselectivity of **6da** were determined by crude $^1$H NMR of the reaction using 1,3,5-trimethoxybenzene as internal standard to be 30% (*r.r.*: 5.4/1.0) (**Figure S5**).

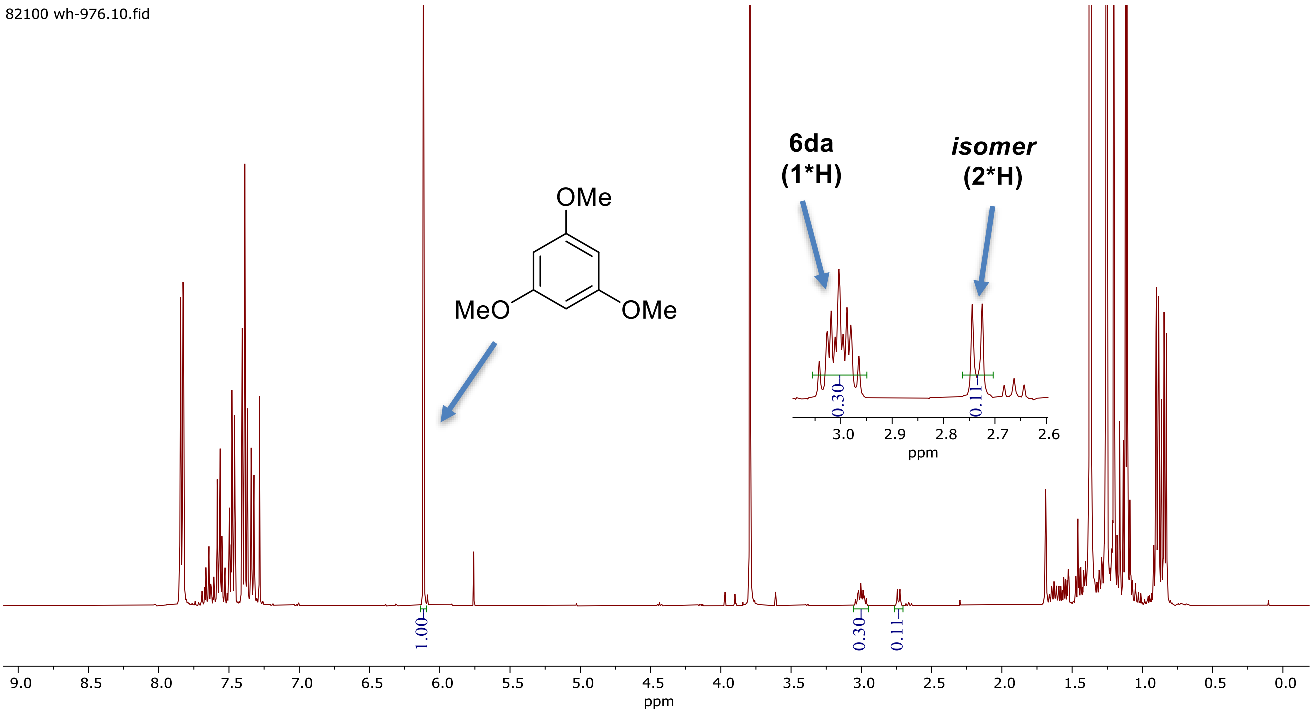


**Figure S5. Crude $^1$H NMR Result of 6da with PhLi as Lithium Reagent**

Prepared following **General Procedure C**, using 2,2'-(4-methylpentane-1,2-diyl)bis(4,4,5,5-tetramethyl-1,3,2-dioxaborolane) (**1d**) (67.6 mg, 0.20 mmol, 1.0 equiv.), 4-bromo-*N,N*-dimethylaniline (44 mg, 0.22 mmol, 1.1 equiv.), *tert*-butyllithium (1.7 M in pentane, 260 µL, 0.44 mmol, 2.2 equiv.), 4-bromobenzonitrile (**5a**) (54.6 mg, 0.30 mmol, 1.5 equiv.), 4-CzIPN (7.9 mg, 0.01 mmol, 5.0 mol %), and Ni(TMHD)$_2$ (8.5 mg, 0.02 mmol, 10.0 mol %). The $^1$H NMR yield and regioselectivity of **6da** were determined by crude $^1$H NMR of the reaction using 1,3,5-trimethoxybenzene as internal standard to be 56% (*r.r.*: 6.3/1.0) (**Figure S6**). The crude material was oxidized following the procedure mentioned above. Purification by flash column chromatography (67:33 *n*-hexane/EtOAc) gave the title compound **6da** (17 mg, 42%) as a colorless oil.

**TLC**: $R_f$ = 0.19 (80:20 *n*-hexane/EtOAc, $KMnO_4$ stain)

**NMR Spectroscopy (*see spectra*):**

$^1$**H NMR** (400 MHz, $CDCl_3$): $\delta_H$ 7.63 – 7.57 (d, $J$ = 8.2 Hz, 2H), 7.34 (d, $J$ = 8.2 Hz, 2H), 3.76 (dd, $J$ = 10.8, 5.5 Hz, 1H), 3.70 (dd, $J$ = 10.8, 7.7 Hz, 1H), 2.95 (ddt, $J$ = 9.6, 7.6, 5.5 Hz, 1H), 1.61 – 1.46 (m, 2H), 1.42 (brs, 1H), 1.40 – 1.30 (m, 1H), 0.87 (d, $J$ = 4.2 Hz, 3H), 0.85 (d, $J$ = 4.2 Hz, 3H) ppm;

$^{13}$**C NMR** (101 MHz, $CDCl_3$): $\delta_C$ 149.0, 132.5, 129.1, 119.0, 110.6, 67.6, 46.7, 41.0, 25.5, 23.5, 21.9 ppm.

**IR** (film): $\nu_{max}$ 3436, 2955, 2928, 2869, 2228, 1739, 1608, 1505, 1467, 1415, 1367, 1177, 1053, 835, 569 cm$^{-1}$.

**HRMS** (EI$^+$): m/z calculated for $C_{13}H_{18}ON$ [M+H]$^+$, 204.1382; found, 204.1383.

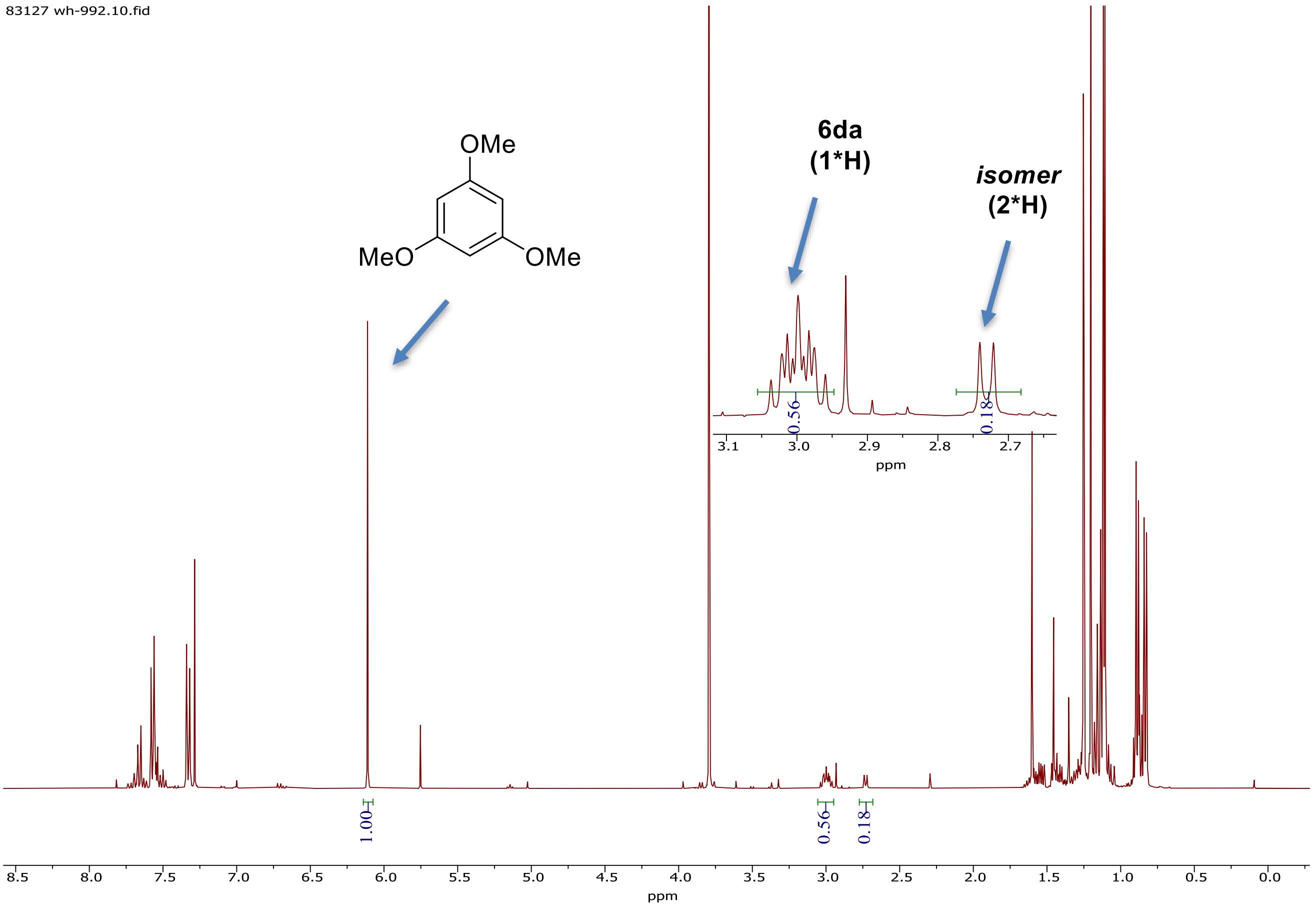


**Figure S6. Crude $^1$H NMR Result of 6da with 4-$Me_2NC_6H_4$Li as Lithium Reagent**

**4-(1-Hydroxypentan-2-yl)benzonitrile (6ea)**

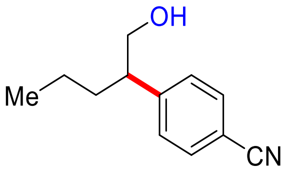

**6ea**

Prepared following **General Procedure C**, using 2,2'-(pentane-1,2-diyl)bis(4,4,5,5-tetramethyl-1,3,2-dioxaborolane) (**1e**) (64.8 mg, 0.20 mmol, 1.0 equiv.), 4-bromo-*N,N*-dimethylaniline (44.0 mg, 0.22 mmol, 1.1 equiv.), *tert*-butyllithium (1.7 M in pentane, 260 µL, 0.44 mmol, 2.2 equiv.), 4-bromobenzonitrile (**5a**) (54.6 mg, 0.30 mmol, 1.5 equiv.), 4-CzIPN (7.9 mg, 0.01 mmol, 5.0 mol %), and Ni(TMHD)$_2$ (8.5 mg, 0.02 mmol, 10.0 mol %). The crude material was oxidized following the procedure mentioned above. Purification by flash column chromatography (75:25 *n*-hexane/EtOAc) gave the title compound **6ea** (15.0 mg, 40%) as a colorless oil and compound **6ea'** (4.0 mg, 11%). The regioselectivity was determined by GC-Fid analysis from the crude reaction mixture (results see **Figure S8**).

**TLC**: $R_f$ = 0.38 (67:33 *n*-hexane/EtOAc, $KMnO_4$ stain)

**NMR Spectroscopy (*see spectra*):**

$^1$**H NMR** (400 MHz, $CDCl_3$): $\delta_H$ 7.61 (d, *J* = 8.4 Hz, 2H), 7.33 (d, *J* = 8.1 Hz, 2H), 3.87 – 3.67 (m, 2H), 2.86 (ddt, *J* = 9.5, 7.6, 5.5 Hz, 1H), 1.71 (dddd, *J* = 13.6, 9.6, 6.8, 5.4 Hz, 1H), 1.62 – 1.48 (m, 1H), 1.37 (t, *J* = 5.5 Hz, 1H), 1.29 – 1.12 (m, 2H), 0.88 (t, *J* = 7.3 Hz, 3H) ppm;

$^{13}$**C NMR** (101 MHz, $CDCl_3$): $\delta_C$ 148.9, 132.5, 129.0, 119.1, 110.6, 67.1, 48.7, 34.1, 20.5, 14.1 ppm.

**IR** (film): $\nu_{max}$ 3441, 2957, 2872, 2228, 1738, 1608, 1505, 1466, 1415, 1378, 1217, 1051, 1019, 834 cm$^{-1}$.

**HRMS** (EI$^+$): m/z calculated for $C_{12}H_{15}ON$ [M]$^+$, 189.1148; found, 189.1148.

**4-(2-Hydroxypentyl)benzonitrile (6ea')**

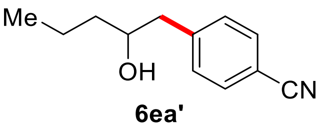

**6ea'**

**TLC**: $R_f$ = 0.48 (67:33 *n*-hexane/EtOAc, $KMnO_4$ stain)

**NMR Spectroscopy (*see spectra*):**

$^1$**H NMR** (400 MHz, $CDCl_3$): $\delta_H$ 7.60 (d, *J* = 8.0 Hz, 2H), 7.34 (d, *J* = 8.0 Hz, 2H), 3.97 – 3.80 (m, 1H), 2.86 (dd, *J* = 13.7, 4.2 Hz, 1H), 2.73 (dd, *J* = 13.7, 8.3 Hz, 1H), 1.56 – 1.43 (m, 2H), 1.44 – 1.35 (m, 2H), 0.94 (t, *J* = 7.0 Hz, 3H) ppm;

$^{13}$**C NMR** (101 MHz, $CDCl_3$): $\delta_C$ 144.8, 132.3, 130.4, 119.1, 110.5, 72.2, 44.2, 39.4, 19.0, 14.1 ppm.

**IR** (film): $\nu_{max}$ 3457, 2958, 2931, 2872, 2228, 1738, 1608, 1506, 1365, 1217, 1121, 1019, 818 cm$^{-1}$.

**HRMS** (EI$^+$): m/z calculated for $C_{12}H_{16}ON$ [M+H]$^+$, 190.1226; found, 190.1226.

### 4-[1-(Hydroxymethyl)cyclohexyl]benzonitrile (6fa)

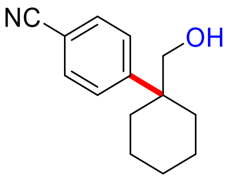


**6fa**

Prepared following a modified **General Procedure B**, using 4,4,5,5-tetramethyl-2-{[1-(4,4,5,5-tetramethyl-1,3,2-dioxaborolan-2-yl)cyclohexyl]methyl}-1,3,2-dioxaborolane (**1f**) (70.0 mg, 0.2 mmol, 1.0 equiv.), MeLi (1.6 M in diethyl ether, 0.14 mL, 0.22 mmol, 1.1 equiv.), 4-bromobenzonitrile (**5a**) (109.2 mg, 0.60 mmol, 3.0 equiv.), 4-CzIPN (7.9 mg, 0.01 mmol, 5.0 mol %), and Ni(TMHD)$_2$ (8.5 mg, 0.02 mmol, 10.0 mol %) in $CH_3CN$. After reaction, the mixture was transferred to a round flask (50 mL) and concentrated under reduced pressure. The crude material was oxidized following the procedure mentioned above. Purification by flash column chromatography (67:33 *n*-hexane/EtOAc) gave the title compound **6fa** (13 mg, 30%) as a colorless oil.

**TLC**: $R_f$ = 0.37 (67:33 *n*-hexane/EtOAc, $KMnO_4$ stain)

**NMR Spectroscopy (*see spectra*):**

$^1$**H NMR** (400 MHz, $CDCl_3$): $\delta_H$ 7.65 (d, *J* = 8.7 Hz, 2H), 7.51 (d, *J* = 8.6 Hz, 2H), 3.53 (s, 2H), 2.19 – 2.08 (m, 2H), 1.69 – 1.50 (m, 5H), 1.45 – 1.24 (m, 3H), 1.21 (brs, 1H) ppm;

$^{13}$**C NMR** (101 MHz, $CDCl_3$): $\delta_C$ 150.4, 132.3, 128.4, 119.1, 110.1, 72.6, 44.7, 32.5, 26.5, 22.1 ppm.

**IR** (film): $\nu_{max}$ 3441, 2927, 2859, 2226, 1738, 1606, 1505, 1455, 1376, 1230, 1039, 833, 621 cm$^{-1}$.

**HRMS** (EI$^+$): m/z calculated for $C_{14}H_{18}ON$ [M+H]$^+$, 216.1383; found, 216.1383.

### (*trans*)-4-[2-(4,4,5,5-Tetramethyl-1,3,2-dioxaborolan-2-yl)cyclopentyl]benzonitrile ((*trans*)-6ga)

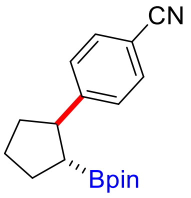


**(*trans*)-6ga**

Prepared following **General Procedure C**, using 1,2-bis(4,4,5,5-tetramethyl-1,3,2-dioxaborolan-2-yl)cyclopentane (**1g**) (64.5 mg, 0.2 mmol, 1.0 equiv.), 4-bromo-*N,N*-dimethylaniline (44.0 mg, 0.22 mmol, 1.1

equiv.), *tert*-butyllithium (1.7 M in pentane, 260 µL, 0.44 mmol, 2.2 equiv.), 4-bromobenzonitrile (**5a**) (54.6 mg, 0.30 mmol, 1.5 equiv.), 4-CzIPN (7.9 mg, 0.01 mmol, 5.0 mol %), and $Ni(TMHD)_2$ (8.5 mg, 0.02 mmol, 10.0 mol %). Purification by flash column chromatography (91:9 pentane/$Et_2O$) gave the title compound **(*trans*)-6ga** (40 mg, 67%) as a colorless oil.

**TLC**: $R_f$ = 0.30 (91:9 *n*-hexane/EtOAc, CAM stain)

**NMR Spectroscopy (*see spectra*):**

**$^1$H NMR** (400 MHz, $CDCl_3$): $\delta_H$ 7.54 (d, *J* = 8.3 Hz, 2H), 7.34 (d, *J* = 8.2 Hz, 2H), 3.15 – 3.03 (m, 1H), 2.16 – 2.04 (m, 1H), 2.06 – 1.93 (m, 1H), 1.89 – 1.72 (m, 2H), 1.72 – 1.56 (m, 2H), 1.38 – 1.26 (m, 1H), 1.16 (s, 6H), 1.15 (s, 6H) ppm;

**$^{13}$C NMR** (101 MHz, $CDCl_3$): $\delta_C$ 152.2, 132.1, 128.2, 119.4, 109.6, 83.3, 49.4, 35.9, 29.2, 26.6, 24.8, 24.7 ppm. The carbon attached to boron was not observed due to quadrupolar relaxation;

**$^{11}$B NMR** (128 MHz, $CDCl_3$): $\delta_B$ 35.96 ppm.

**IR** (film): $\nu_{max}$ 2974, 2870, 2227, 1740, 1607, 1415, 1370, 1319, 1216, 1143, 851, 830, 561 $cm^{-1}$.

**HRMS** ($ESI^+$): m/z calculated for $C_{18}H_{25}BNO_2$ $[M+H]^+$, 298.1973; found, 298.1974.

**(*trans*)-4,4,5,5-Tetramethyl-2-(2-phenylcyclohexyl)-1,3,2-dioxaborolane ((*trans*)-6ha)**

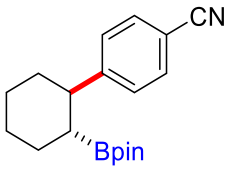


**(*trans*)-6ha**

Prepared following **General Procedure C**, using 1,2-bis(4,4,5,5-tetramethyl-1,3,2-dioxaborolan-2-yl)cyclohexane (**1h**) (67.3 mg, 0.2 mmol, 1.0 equiv.), 4-bromo-*N,N*-dimethylaniline (44.0 mg, 0.22 mmol, 1.1 equiv.), *tert*-butyllithium (1.7 M in pentane, 260 µL, 0.44 mmol, 2.2 equiv.), 4-bromobenzonitrile (**5a**) (54.6 mg, 0.30 mmol, 1.5 equiv.), 4-CzIPN (7.9 mg, 0.01 mmol, 5.0 mol %), and $Ni(TMHD)_2$ (8.5 mg, 0.02 mmol, 10.0 mol %). Purification by flash column chromatography (91:9 pentane/$Et_2O$) gave the title compound **(*trans*)-6ha** (31.5 mg, 55%) as a colorless oil.

**TLC**: $R_f$ = 0.31 (80:20 *n*-hexane/EtOAc, CAM stain)

**NMR Spectroscopy (*see spectra*):**

**$^1$H NMR** (400 MHz, $CDCl_3$): $\delta_H$ 7.53 (d, *J* = 8.3 Hz, 2H), 7.31 (d, *J* = 8.3 Hz, 2H), 2.63 (td, *J* = 11.7, 3.2 Hz, 1H), 1.90 – 1.74 (m, 4H), 1.50 – 1.17 (m, 5H), 0.96 (s, 6H), 0.95 (s, 6H) ppm;

**$^{13}$C NMR** (101 MHz, $CDCl_3$): $\delta_C$ 153.6, 132.1, 128.5, 119.4, 109.6, 83.0, 46.4, 35.8, 28.1, 26.8, 26.7, 24.6, 24.4 ppm. The carbon attached to boron was not observed due to quadrupolar relaxation;

**$^{11}$B NMR** (128 MHz, $CDCl_3$): $\delta_B$ 33.91 ppm.

**IR** (film): $\nu_{max}$ 2977, 2923, 2851, 2226, 1607, 1446, 1377, 1323, 1260, 1143, 848, 832 cm$^{-1}$.

**HRMS** (ESI$^+$): m/z calculated for $C_{19}H_{27}BNO_2$ [M+H]$^+$, 312.2129; found, 312.2131.

**4-(3-Hydroxybicyclo[2.2.1]heptan-2-yl)benzonitrile (6ia)**

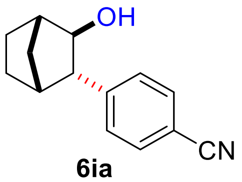


**6ia**

Prepared following **General Procedure C**, using (1*R*,2*S*,3*R*,4*S*)-2,3-bis(4,4,5,5-tetramethyl-1,3,2-dioxaborolan-2-yl)bicyclo[2.2.1]heptane (**1i**) (69.6 mg, 0.2 mmol, 1.0 equiv.), 4-bromo-*N,N*-dimethylaniline (44.0 mg, 0.22 mmol, 1.1 equiv.), *tert*-butyllithium (1.7 M in pentane, 260 µL, 0.44 mmol, 2.2 equiv.), 4-bromobenzonitrile (**5a**) (54.6 mg, 0.30 mmol, 1.5 equiv.), 4-CzIPN (7.9 mg, 0.01 mmol, 5.0 mol %), and Ni(TMHD)$_2$ (8.5 mg, 0.02 mmol, 10.0 mol %). The crude material was oxidized following the procedure mentioned above. Purification by flash column chromatography (67:33 *n*-hexane/EtOAc) gave the title compound **6ia** (20 mg, 47%) as a colorless oil.

**TLC**: $R_f$ = 0.30 (67:33 *n*-hexane/EtOAc, $KMnO_4$ stain)

**NMR Spectroscopy (*see spectra*):**

$^1$**H NMR** (400 MHz, $CDCl_3$): $\delta_H$ 7.58 (d, *J* = 8.3 Hz, 2H), 7.31 (d, *J* = 8.3 Hz, 2H), 3.94 (s, 1H), 2.97 (t, *J* = 4.3 Hz, 1H), 2.56 – 2.46 (m, 1H), 2.26 (d, *J* = 3.5 Hz, 1H), 1.95 – 1.84 (m, 2H), 1.69 – 1.52 (m, 1H), 1.45 – 1.38 (m, 1H), 1.35 – 1.23 (m, 1H), 1.15 – 1.02 (m, 2H) ppm;

$^{13}$**C NMR** (101 MHz, $CDCl_3$): $\delta_C$ 147.2, 132.1, 128.9, 119.2, 109.9, 79.3, 58.5, 45.6, 41.4, 36.8, 25.2, 21.8 ppm.

**IR** (film): $\nu_{max}$ 3436, 2956, 2875, 2227, 1739, 1607, 1504, 1365, 1217, 1065, 1007, 834, 818, 559 cm$^{-1}$.

**HRMS** (EI$^+$): m/z calculated for $C_{14}H_{15}ON$ [M]$^+$, 213.1148; found, 213.1147.

**4-[(1*R*,6ar,4*R*,6a*S*)-4-Hydroxyoctahydropentalen-1-yl)benzonitrile (6ja)**

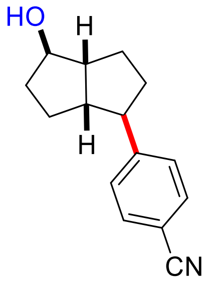


**6ja**

Prepared following **General Procedure C**, using (*Z*)-5,6-bis(4,4,5,5-tetramethyl-1,3,2-dioxaborolan-2-yl)cyclooct-1-ene (**1j**) (69.6 mg, 0.2 mmol, 1.0 equiv.), 4-bromo-*N,N*-dimethylaniline (44.0 mg, 0.22 mmol, 1.1 equiv.), *tert*-butyllithium (1.7 M in pentane, 260 µL, 0.44 mmol, 2.2 equiv.), 4-bromobenzonitrile (**5a**) (54.6 mg, 0.30 mmol, 1.5 equiv.), 4-CzIPN (7.9 mg, 0.01 mmol, 5.0 mol %), and $Ni(TMHD)_2$ (8.5 mg, 0.02 mmol, 10.0 mol %). The crude material was oxidized following the procedure mentioned above. Purification by flash column chromatography (67:33 *n*-hexane/EtOAc) gave the title compound **6ja** (28 mg, 61%) as a colorless oil.

**TLC**: $R_f$ = 0.20 (67:33 *n*-hexane/EtOAc, $KMnO_4$ stain)

**NMR Spectroscopy (*see spectra*):**

> **$^1$H NMR** (400 MHz, $CDCl_3$): $\delta_H$ 7.58 (d, *J* = 8.5 Hz, 2H), 7.33 (d, *J* = 8.2 Hz, 2H), 4.07 (dt, *J* = 4.0, 1.9 Hz, 1H), 2.66 – 2.48 (m, 3H), 2.14 (dddd, *J* = 12.8, 8.4, 6.5, 2.1 Hz, 1H), 2.07 – 1.93 (m, 2H), 1.93 – 1.80 (m, 1H), 1.74 – 1.59 (m, 2H), 1.53 – 1.36 (m, 2H), 1.28 – 1.16 (m, 1H) ppm;

> **$^{13}$C NMR** (101 MHz, $CDCl_3$): $\delta_C$ 151.2, 132.4, 128.2, 119.3, 110.0, 79.4, 53.7, 53.5, 50.7, 36.2, 33.5, 31.3, 28.9 ppm.

**IR** (film): $\nu_{max}$ 3404, 2944, 2865, 2226, 1736, 1606, 1504, 1448, 1347, 1032, 828, 560 $cm^{-1}$.

**HRMS** ($ESI^+$): m/z calculated for $C_{15}H_{17}NONa$ $[M+Na]^+$, 250.1202; found, 250.1198.

**4-{2-[(1*R*,5*S*)-5-Hydroxy-4-methylcyclohex-3-en-1-yl]propan-2-yl}benzonitrile (6ka)**

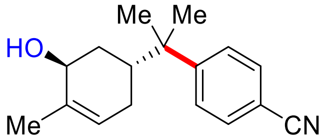


**6ka**

Prepared following a modified **General Procedure B**, using 2,2'-{(2*R*)-2,6,6-trimethylbicyclo[3.1.1]heptane-2,3-diyl}bis(4,4,5,5-tetramethyl-1,3,2-dioxaborolane) (**1k**) (78.0 mg, 0.2 mmol, 1.0 equiv.), MeLi (1.6 M in diethyl ether, 0.14 mL, 0.22 mmol, 1.1 equiv.), 4-bromobenzonitrile (**5a**) (109.2 mg, 0.60 mmol, 3.0 equiv.), 4-CzIPN (7.9 mg, 0.01 mmol, 5.0 mol %), and $Ni(TMHD)_2$ (8.5 mg, 0.02 mmol, 10.0 mol %) in $CH_3CN$. After reaction, the mixture was transferred to a round flask (50 mL) and concentrated under reduced pressure. The crude material

was oxidized following the procedure mentioned above. Purification by flash column chromatography (67:33 *n*-hexane/EtOAc) gave the title compound **6ka** (13 mg, 26%) as a colorless oil.

**TLC**: $R_f$ = 0.43 (67:33 *n*-hexane/EtOAc, $KMnO_4$ stain)

**NMR Spectroscopy (*see spectra*):**

> **$^1$H NMR** (400 MHz, $CDCl_3$): $\delta_H$ 7.58 (d, *J* = 8.7 Hz, 2H), 7.45 (d, *J* = 8.7 Hz, 2H), 5.53 – 5.44 (m, 1H), 3.97 (dd, *J* = 3.8, 2.0 Hz, 1H), 2.00 (dddd, *J* = 13.4, 11.3, 4.9, 2.5 Hz, 1H), 1.78 – 1.71 (m, 3H), 1.71 – 1.58 (m, 3H), 1.51 (brs, 1H), 1.31 (s, 3H), 1.27 (s, 3H) ppm;

> **$^{13}$C NMR** (101 MHz, $CDCl_3$): $\delta_C$ 155.3, 134.4, 132.0, 127.2, 125.6, 119.2, 109.6, 68.8, 40.3, 38.5, 33.4, 27.5, 25.3, 24.8, 20.9 ppm.

**IR** (film): $\nu_{max}$ 3411, 2969, 2916, 2227, 1739, 1606, 1505, 1366, 1217, 1051, 908, 838, 732, 547 $cm^{-1}$.

**HRMS** ($EI^+$): m/z calculated for $C_{14}H_{17}ON$ $[M]^+$, 255.1618; found, 255.1618.

# 3. MECHANISTIC STUDIES

## 3.1. Radical Clock Experiment

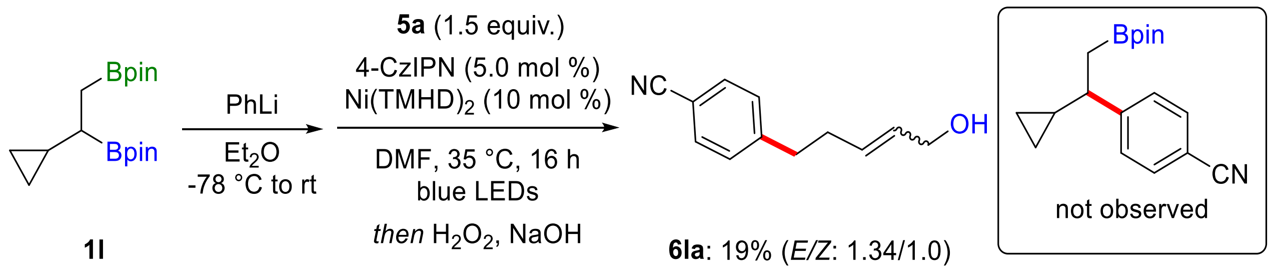


Following **General Procedure B**, using 2,2'-(1-cyclopropylethane-1,2-diyl)bis(4,4,5,5-tetramethyl-1,3,2-dioxaborolane) (**1l**) (64.4 mg, 0.20 mmol, 1.0 equiv.), PhLi (1.9 M in dibutyl ether, 0.12 mL, 0.22 mmol, 1.1 equiv.), 4-bromobenzonitrile (**5a**) (54.6 mg, 0.30 mmol, 1.5 equiv.), 4-CzIPN (7.9 mg, 0.01 mmol, 5.0 mol %), and Ni(TMHD)$_2$ (8.5 mg, 0.02 mmol, 10.0 mol %). The crude material was oxidized following the procedure mentioned above. Purification by flash column chromatography (67:33 *n*-hexane/EtOAc) gave the title compound **6la** (7.0 mg, 19%) as a colorless oil.

### (*E/Z*)-4-(5-Hydroxypent-3-en-1-yl)benzonitrile (6la)

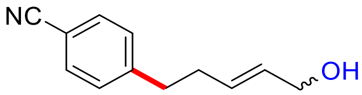


**6la**

**TLC**: $R_f$ = 0.37 (67:33 *n*-hexane/EtOAc, $KMnO_4$ stain)

**NMR Spectroscopy (*see spectra*):**

$^1$**H NMR** (400 MHz, $CDCl_3$): $\delta_H$ 7.57 (dd, *J* = 8.3, 1.8 Hz, 2H), 7.28 (d, *J* = 8.0 Hz, 2H), 5.76 – 5.46 (m, 2H), 4.12 – 4.05 (m, 2H), 2.81 – 2.71 (m, 2H), 2.49 – 2.34 (m, 2H), 1.37 (brs, 1H) ppm;

$^{13}$**C NMR** (101 MHz, $CDCl_3$): $\delta_C$ (*Major isomer*) 147.4, 132.3, 131.0, 130.5, 129.4, 119.2, 110.0, 63.6, 35.8, 33.5, ppm; $\delta_C$ (*Minor isomer*) 147.2, 132.3, 130.9, 130.0, 129.5, 119.1, 110.1, 58.6, 36.0, 28.9 ppm.

**IR** (film): $\nu_{max}$ 3402, 2929, 2858, 2227, 1738, 1607, 1505, 1415, 1365, 1217, 1091, 972, 824, 558 cm$^{-1}$.

**HRMS** (ESI$^+$): m/z calculated for $C_{12}H_{13}NONa$ [M+Na]$^+$, 210.0889; found, 210.0884.

### 3.2. 2.0 mmol-Scale Reaction

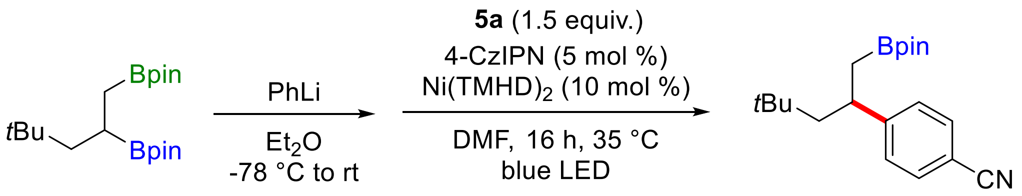


**1a** (2.0 mmol)

**6aa**: 71% (*r.r.*: 19:1)

Following **General Procedure B**, in a 30 mL glass vial using 2,2'-(4,4-dimethylpentane-1,2-diyl)bis(4,4,5,5-tetramethyl-1,3,2-dioxaborolane) (**1a**) (704.3 mg, 2.0 mmol, 1.0 equiv.), PhLi (1.9 M in dibutyl ether, 1.16 mL, 2.2 mmol, 1.1 equiv.), 4-bromobenzonitrile (**5a**) (546.0 mg, 3.0 mmol, 1.5 equiv.), 4-CzIPN (80.0 mg, 0.1 mmol, 5.0 mol %), $Ni(TMHD)_2$ (85 mg, 0.2 mmol, 10.0 mol %), and anhydrous DMF (20 mL). Purification by flash column chromatography (90:10 pentane/$Et_2O$) gave the title compound **6aa** (0.46 g, 71%) as a white solid. (See **Figure S7** for experimental setup)

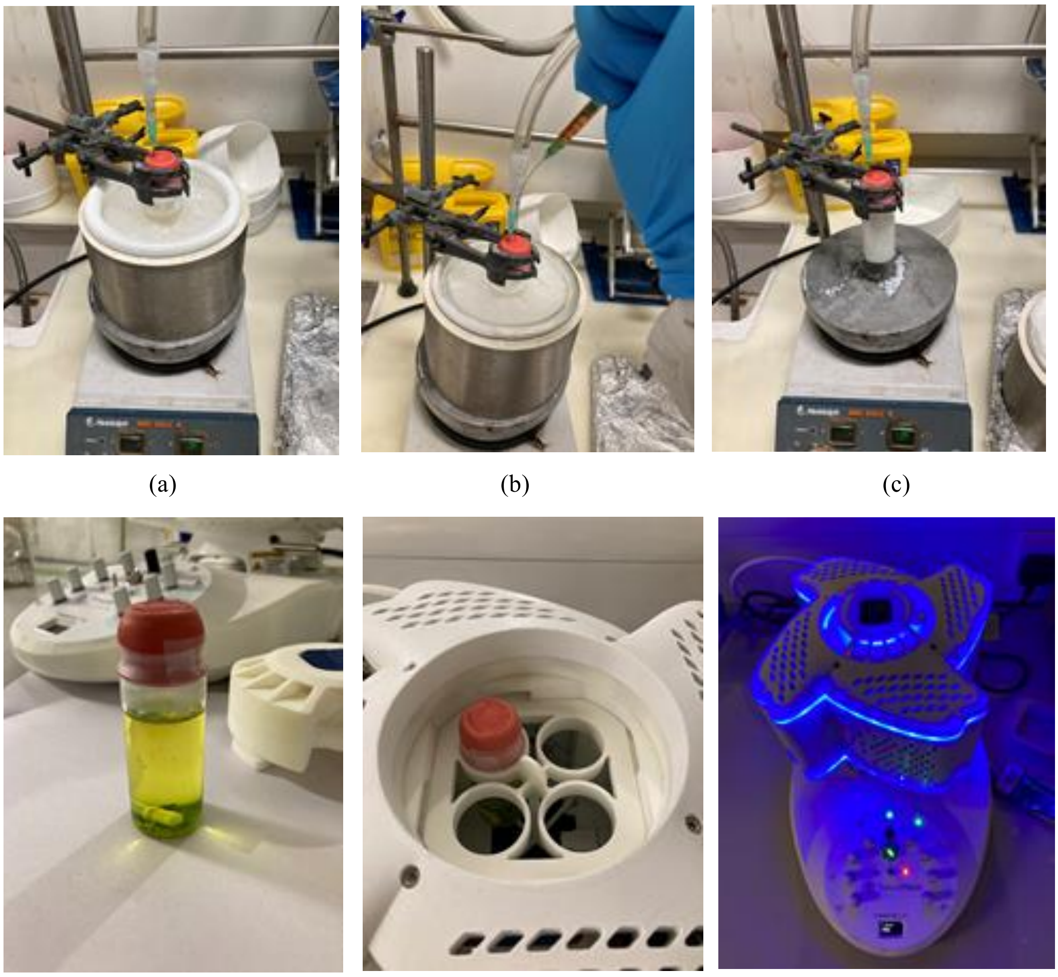

(a) (b) (c)

(d) (e) (f)

**Figure S7. 2.0 mmol-Scale Reaction Setup**

### 3.3. Pd-catalyzed Cross-Coupling Reaction of Cyclic Bis-boronic Ester

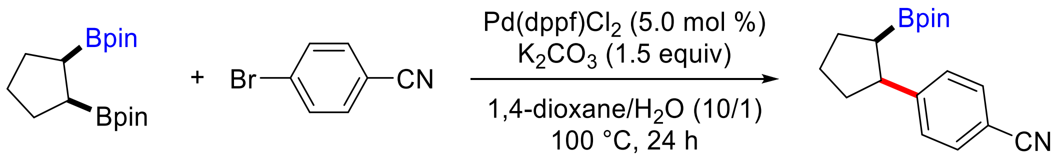


**1g** **5a** **(*cis*)-6ga** 43%

According to a modified literature procedure:[9] To a Schlenk flask were added 1,2-bis(4,4,5,5-tetramethyl-1,3,2-dioxaborolan-2-yl)cyclopentane (**1g**) (64.0 mg, 0.20 mmol, 1.0 equiv.), 4-bromobenzonitrile (55.0 mg, 0.30 mmol, 1.5 equiv.), Pd(dppf)$Cl_2$ (7.4 mg, 0.010 mmol, 5.0 mol%), and $K_2CO_3$ (41.0 mg, 0.30 mmol, 1.5 equiv.), after which the flask was evacuated and back-filled with nitrogen three times. Subsequently, 1,4-dioxane (1.0 mL) and $H_2O$ (0.1 mL) were added via syringe and the resulting suspension was heated to 100 °C for 24 h, after which the reaction mixture was filtered over Celite, eluting with diethyl ether. The filtrate was concentrated under reduced pressure and the resulting crude material was purified by flash column chromatography (91:9 pentane/$Et_2O$) to afford **(*cis*)-6ga** (25.6 mg, 43%) as a colorless oil.

**4-[(*cis*)-2-(4,4,5,5-Tetramethyl-1,3,2-dioxaborolan-2-yl)cyclopentyl]benzonitrile ((*cis*)-6ga)**

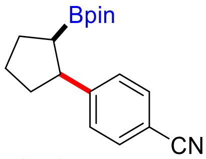


**(*cis*)-6ga**

**TLC**: $R_f$ = 0.26 (91:9 *n*-hexane/EtOAc, CAM stain)

**NMR Spectroscopy (*see spectra*):**

**$^1$H NMR** (400 MHz, $CDCl_3$): $\delta_H$ 7.54 (d, *J* = 8.4 Hz, 2H), 7.34 (d, *J* = 8.3 Hz, 2H), 3.11 – 2.99 (m, 1H), 2.20 (dt, *J* = 12.8, 6.9 Hz, 1H), 2.11 (dtdd, *J* = 12.7, 7.9, 4.8, 1.1 Hz, 1H), 1.92 (ddt, *J* = 12.7, 9.3, 7.6 Hz, 1H), 1.86 – 1.75 (m, 1H), 1.69 – 1.38 (m, 3H), 1.25 (s, 12H) ppm;

**$^{13}$C NMR** (101 MHz, $CDCl_3$): $\delta_C$ 152.0, 132.2, 128.2, 119.4, 109.5, 83.2, 47.7, 37.5, 34.7, 27.7, 24.9 ppm. The carbon attached to boron was not observed due to quadrupolar relaxation;

**$^{11}$B NMR** (128 MHz, $CDCl_3$): $\delta_B$ 34.45 ppm.

**IR** (film): $\nu_{max}$ 2974, 2870, 2227, 1740, 1607, 1415, 1370, 1319, 1216, 1143, 851, 830 cm$^{-1}$.

**HRMS** (ESI$^+$): m/z calculated for $C_{18}H_{24}BNNO_2$ [M+Na]$^+$, 320.1796; found, 320.1801.

## 3.4. Regioselectivity Studies for the Reaction of 1e

### 3.4.1. Regioselectivity of the Reaction of Pinacol Bis-Boronic Ester 1e

**Table S2: Studies for the Regioselectivity of the Reaction with 1e**

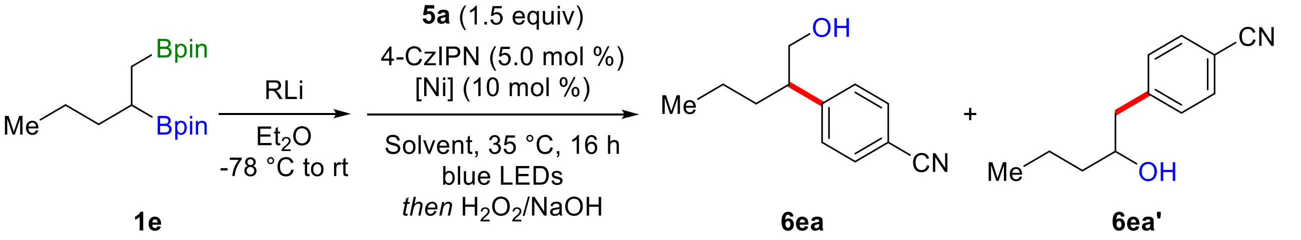


| Entry[a] | RLi | [Ni] | Solvent | Yield/6ea[b] | *r.r.* (6ea/6ea')[c] |
|---|---|---|---|---|---|
| 1 | PhLi | $Ni(TMHD)_2$ | DMF | 26% | 3.0/1.0 |
| 2 | $4\text{-}Me_2NC_6H_4Li$ | $Ni(TMHD)_2$ | DMF | 51%[d] | 4.0/1.0 |
| 3 | PhLi | $Ni(bpy)Br_2$ | DMF | 17% | 1.0/1.0 |
| 4 | PhLi | $Ni(TMHD)_2$ | $CH_3CN$ | 22% | 1.3/1.0 |
| 5 | MeLi | $Ni(TMHD)_2$ | $CH_3CN$ | 39% | 1.3/1.0 |
| 6 | MeLi | $Ni(TMHD)_2$ | DMF | 35% | 3.7/1.0 |

[a] **1b** (0.2 mmol), RLi (1.1 equiv), **5a** (1.5 equiv), 4-CzIPN (5.0 mol %), [Ni] (10.0 mol %), Solvent (2.0 mL) under $N_2$ in photochemical reactor (PhotoCube™) for 16 hours. [b] $^1$H NMR yield by using 1,3,5-trimethoxybenzene as internal standard; [c] Regioselectivity determined by GC-Fid analysis (**see Figure S8**); [d] Isolated yield.

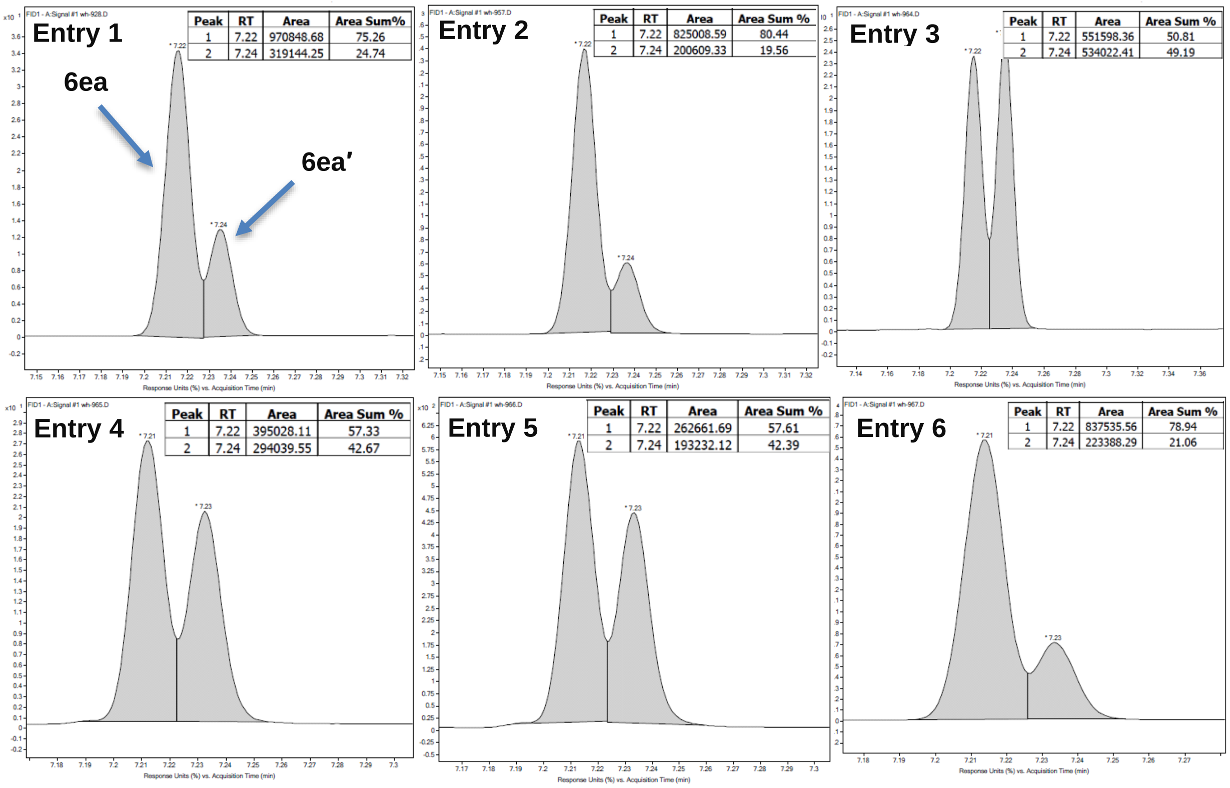


**Figure S8. GC-Fid Results for the Regioselectivity of Reaction with 1e**

### 3.4.2. Regioselectivity of the Reaction of Neopentyl Glycol Bis-Boronic Ester 1e′

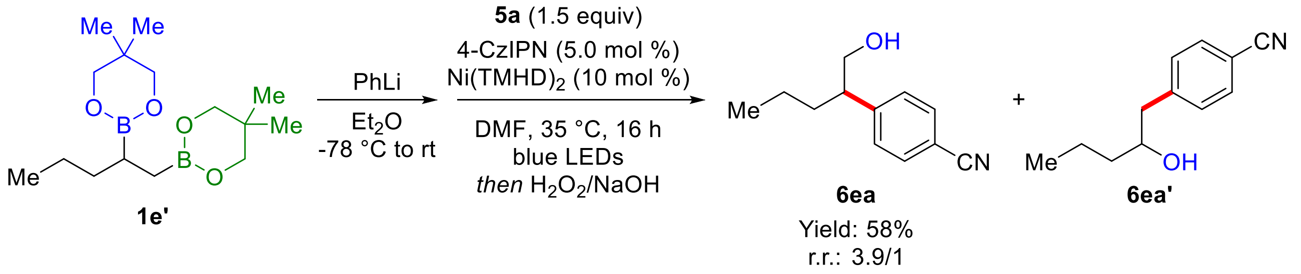


The above reaction was carried out according to **General Procedure C**, using 2,2'-(pentane-1,2-diyl)bis(5,5-dimethyl-1,3,2-dioxaborinane) (**1e′**) (59.2 mg, 0.20 mmol, 1.0 equiv.), PhLi (1.9 M in dibutyl ether, 0.12 mL, 0.22 mmol, 1.1 equiv.), 4-bromobenzonitrile (**5a**) (54.6 mg, 0.30 mmol, 1.5 equiv.), 4-CzIPN (7.9 mg, 0.01 mmol, 5.0 mol %), and Ni(TMHD)2 (8.5 mg, 0.02 mmol, 10.0 mol %). The crude material was oxidized before the yield and regioselectivity were determined by $^{1}$H NMR and GC-Fid analysis (**Figure S9**), respectively.

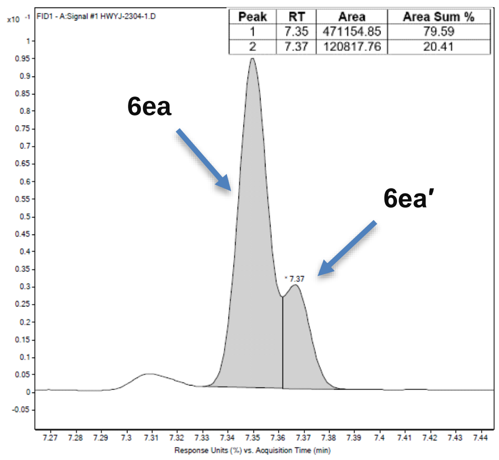


**Figure S9. GC-Fid Results for the Regioselectivity of Reaction with 1e′**

### 3.5. Ni-Catalyzed Methylation of Aryl Halides

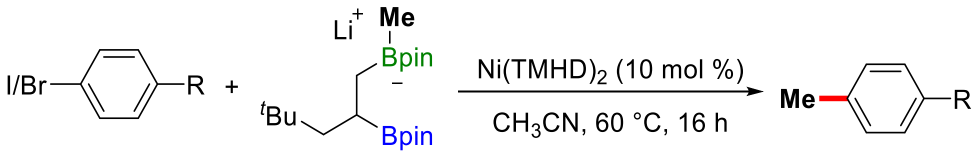


**5** **2a'** **8**

In glovebox, a flame-dried nitrogen-flushed Schlenk tube (10 mL) equipped with a magnetic stir bar was added 2,2'-(4,4-dimethylpentane-1,2-diyl)bis(4,4,5,5-tetramethyl-1,3,2-dioxaborolane) (**1a**) (105.6 mg, 0.30 mmol, 1.5 equiv.). The tube was sealed with a septum and removed from the glovebox, and then anhydrous diethyl ether was added under $N_2$. The solution was cooled to −78 °C (dry ice/acetone) and MeLi (1.6 M in diethyl ether, 200 µL, 0.32 mmol, 1.6 equiv.) was added dropwise. The mixture was allowed to stir for 30 min at −78 °C before removing the cooling bath and warming to ambient temperature. After 30 min, the solvent was removed under high vacuum, and the vial with boronate complex was quickly put into the glovebox, and then aryl halides **5** (0.20 mmol, 1.0 equiv.), $Ni(TMHD)_2$ (8.5 mg, 0.02 mmol, 10.0 mol %) and anhydrous $CH_3CN$ (2.0 mL) were added. The Schlenk tube was tightly sealed, then removed from the glovebox and stirred at 60 °C for 16 h. After reaction, the mixture was transferred to a round flask (50 mL) and concentrated under reduced pressure. The crude material was purified by flash column chromatography (pentane/$Et_2O$) to afford the methylation product **8a**-**8c**.

**1-Methyl-4-(methylsulfonyl)benzene (8a)**

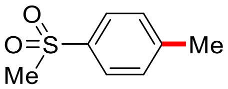


**8a**

Prepared following above procedure, using 1-bromo-4-(methylsulfonyl)benzene (47.0 mg, 0.20 mmol, 1.0 equiv.), $Ni(TMHD)_2$ (8.5 mg, 0.02 mmol, 10.0 mol %). Purification by flash column chromatography (67:33 *n*-hexane/EtOAc) gave the title compound **8a** (17 mg, 50%) as a colorless oil.

**TLC**: $R_f$ = 0.35 (67:33 *n*-hexane/EtOAc, $KMnO_4$ stain)

**NMR Spectroscopy (*see spectra*):**

**$^1$H NMR** (400 MHz, $CDCl_3$): $\delta_H$ 7.82 (d, $J$ = 8.4 Hz, 2H), 7.36 (d, $J$ = 8.6 Hz, 2H), 3.03 (s, 3H), 2.45 (s, 3H) ppm;

**$^{13}$C NMR** (101 MHz, $CDCl_3$): $\delta_C$ 144.8, 137.9, 130.1, 127.5, 44.7, 21.7 ppm.

All recorded spectroscopic data matched those previously reported in the literature.[10]

**4-Tosylmorpholine (8b)**

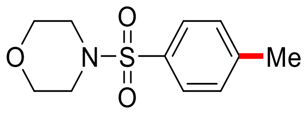

**8b**

Prepared following above procedure, using 4-[(4-bromophenyl)sulfonyl]morpholine (61.3 mg, 0.20 mmol, 1.0 equiv.), Ni(TMHD)$_2$ (8.5 mg, 0.02 mmol, 10.0 mol %). Purification by flash column chromatography (75:25 *n*-hexane/EtOAc) gave the title compound **8b** (25 mg, 52%) as a colorless oil.

**TLC**: $R_f$ = 0.42 (67:33 *n*-hexane/EtOAc, $KMnO_4$ stain)

**NMR Spectroscopy (*see spectra*):**

$^1$**H NMR** (400 MHz, $CDCl_3$): $\delta_H$ 7.63 (d, *J* = 8.3 Hz, 2H), 7.34 (d, *J* = 8.0 Hz, 2H), 3.75 – 3.68 (m, 4H), 3.03 – 2.90 (m, 4H), 2.44 (s, 3H) ppm;

$^{13}$**C NMR** (101 MHz, $CDCl_3$): $\delta_C$ 144.1, 132.3, 129.9, 128.0, 66.2, 46.1, 21.7 ppm.

All recorded spectroscopic data matched those previously reported in the literature.[11]

**1-(*p*-Tolyl)-1*H*-pyrrole (8c)**

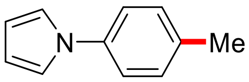

**8c**

Prepared following above procedure, using 1-(4-iodophenyl)-1*H*-pyrrole (53.8 mg, 0.20 mmol, 1.0 equiv.), Ni(TMHD)$_2$ (8.5 mg, 0.02 mmol, 10.0 mol %). Purification by flash column chromatography (95:5 pentane/$Et_2O$) gave the title compound **8c** (24 mg, 76%) as a colorless oil.

**TLC**: $R_f$ = 0.69 (95:5 *n*-hexane/EtOAc, $KMnO_4$ stain)

**NMR Spectroscopy (*see spectra*):**

$^1$**H NMR** (400 MHz, $CDCl_3$): $\delta_H$ 7.29 (d, *J* = 8.5 Hz, 2H), 7.23 (d, *J* = 8.3 Hz, 2H), 7.07 (t, *J* = 2.2 Hz, 2H), 6.34 (t, *J* = 2.2 Hz, 2H), 2.38 (s, 3H) ppm;

$^{13}$**C NMR** (101 MHz, $CDCl_3$): $\delta_C$ 138.7, 135.5, 130.2, 120.7, 119.5, 110.2, 21.0 ppm.

All recorded spectroscopic data matched those previously reported in the literature.[12]

## 3.6. Transformations of 6ab

### 3.6.1. Synthesis of Compound 9

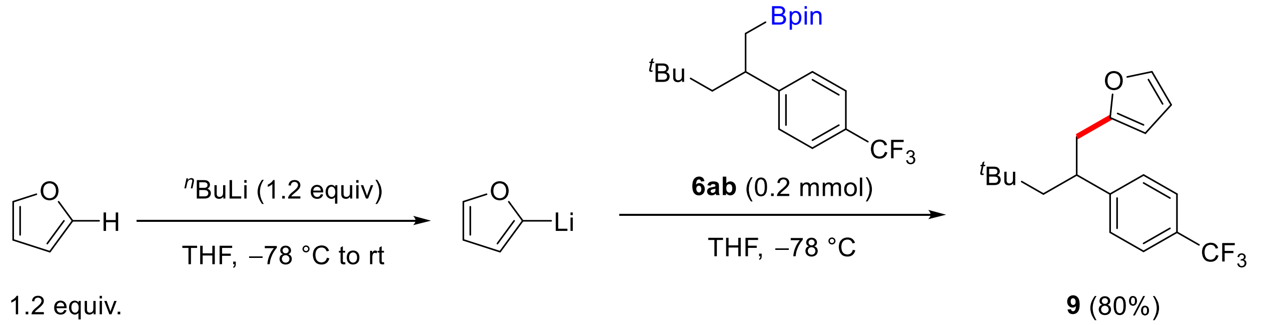


Following a known procedure,[13] To a flame-dried Schlenk flask with a magnetic stir bar was added furan (18 µL, 0.24 mmol, 1.2 equiv.) and anhydrous THF (0.8 mL) under $N_2$. The solution was cooled to −78 °C (dry ice/acetone) and $^n$BuLi (1.6 M in hexanes, 150 µL, 0.24 mmol, 1.2 equiv.) was added dropwise. The cooling bath then was removed, and the mixture was stirred at room temperature for 1 h. The mixture was cooled to –78 °C and 2-{4,4-dimethyl-2-[4-(trifluoromethyl)phenyl]pentyl}-4,4,5,5-tetramethyl-1,3,2-dioxaborolane (**6ab**) (74.1 mg, 0.20 mmol, 1.0 equiv.) was added dropwise as a solution in THF (0.4 M). The mixture was stirred at –78 °C for 1 h and then a solution of NBS (43 mg, 0.24 mmol, 1.2 equiv.) in THF (0.8 M) was added dropwise. After 1 h at –78 °C, $Na_2S_2O_3$ sat. (2.0 mL) was added and the reaction mixture was allowed to warm to room temperature. The reaction mixture was diluted with $Et_2O$ (5.0 mL) and water (5.0 mL). The layers were separated, and the aqueous layer was extracted with $Et_2O$ (3*5.0 mL). The combined organic layers were dried with sodium sulfate, filtered and concentrated under vacuum. The resulting crude material was purified by flash column chromatography (98:2 pentane/$Et_2O$) to afford the title compound **8** (49.5 mg, 80%) as a colorless oil.

**2-{4,4-Dimethyl-2-[4-(trifluoromethyl)phenyl]pentyl}furan (9)**

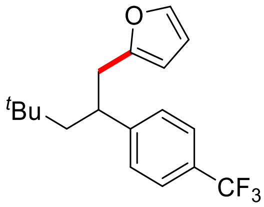


**9**

**TLC**: $R_f$ = 0.57 (98:2 pentane/$Et_2O$, $KMnO_4$ stain)

**NMR Spectroscopy (*see spectra*):**

**$^1$H NMR** (400 MHz, $CDCl_3$): $\delta_H$ 7.53 – 7.47 (m, 2H), 7.29 – 7.22 (m, 3H), 6.19 (dd, $J$ = 3.2, 1.9 Hz, 1H), 5.78 (dd, $J$ = 3.2, 0.8 Hz, 1H), 3.24 – 3.06 (m, 1H), 2.86 (dd, $J$ = 14.8, 7.1 Hz, 1H), 2.78 (dd, $J$ = 14.8, 7.9 Hz, 1H), 1.77 (dd, $J$ = 14.1, 9.0 Hz, 1H), 1.63 (dd, $J$ = 14.1, 3.1 Hz, 1H), 0.76 (s, 9H) ppm;

**$^{13}$C NMR** (101 MHz, $CDCl_3$): $\delta_C$ 153.9, 151.2, 141.1, 128.40 (q, $^2J_{C\text{-}F}$ = 32.3 Hz), 128.2, 125.3 (q, $^3J_{C\text{-}F}$ = 3.8 Hz), 124.5 (q, $^1J_{C\text{-}F}$ = 271.8 Hz), 110.2, 106.6, 49.0, 42.0, 38.3, 31.5, 30.2 ppm;

**$^{19}$F NMR** (377 MHz, $CDCl_3$): $\delta_F$ -62.24 ppm.

**IR** (film): $\nu_{max}$ 2955, 1721, 1618, 1476, 1366, 1323, 1162, 1122, 1069, 1018, 836, 730, 599 cm$^{-1}$.

**HRMS** ($EI^+$): m/z calculated for $C_{18}H_{21}F_3O$ $[M]^+$, 310.1539; found, 310.1539.

### 3.6.2. Synthesis of Compound 10

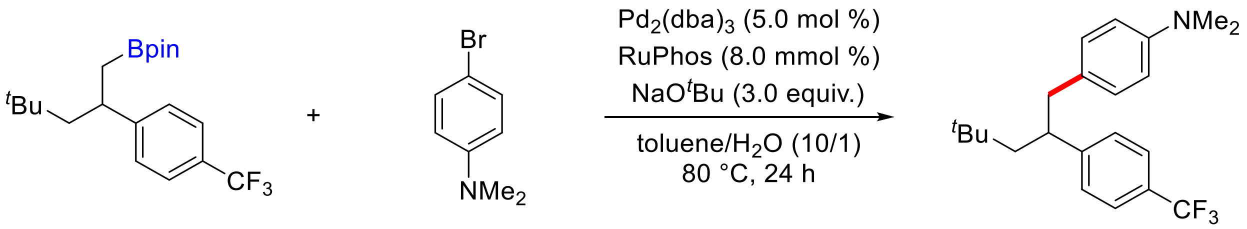


**6ab** (1.2 equiv.) 0.2 mmol **10** (67%)

Following a known procedure,[16] To a flame-dried Schlenk flask with a magnetic stir bar was added 4-bromo-*N,N*-dimethylaniline (40 mg, 0.20 mmol, 1.0 equiv.), 2-{4,4-dimethyl-2-[4-(trifluoromethyl)phenyl]pentyl}-4,4,5,5-tetramethyl-1,3,2-dioxaborolane (**6ab**) (89 mg, 0.24 mmol, 1.2 equiv.), $Pd_2(dba)_3$ (9.2 mg, 0.01 mmol, 5.0 mol %), RuPhos (7.5 mg, 0.016 mmol, 8.0 mol %), toluene (1.0 mL), and water (0.1 mL) under $N_2$. The reaction mixture was stirred for 24 hours at 80 °C. After that, the reaction mixture was cooled to room temperature, $Et_2O$ (5.0 mL) and water (4.0 mL) was added. The organic phase was separated, and the aqueous phase was extracted with $Et_2O$ (3*5.0 mL). The combined organic extracts were dried over anhydrous sodium sulfate, filtered and concentrated under reduced pressure. The resulting crude material was purified by flash column chromatography (91:9 pentane/$Et_2O$) to afford the title compound **10** (49 mg, 67%) as a white solid.

### 4-{4,4-Dimethyl-2-[4-(trifluoromethyl)phenyl]pentyl}-*N,N*-dimethylaniline (10)

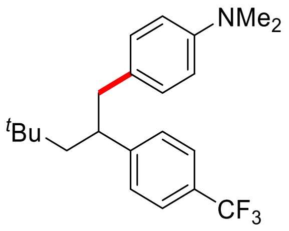


**10**

**TLC**: $R_f$ = 0.44 (91:9 pentane/$Et_2O$, $KMnO_4$ stain)

**NMR Spectroscopy (*see spectra*):**

**$^1$H NMR** (400 MHz, $CDCl_3$): $\delta_H$ 7.47 (d, $J$ = 8.0 Hz, 2H), 7.22 (d, $J$ = 8.0 Hz, 2H), 6.82 (d, $J$ = 8.6 Hz, 2H), 6.61 (d, $J$ = 8.2 Hz, 2H), 2.96 (tdd, $J$ = 8.6, 6.7, 3.3 Hz, 1H), 2.76 (dd, $J$ = 13.1, 7.1 Hz, 6H), 2.70 (dd, $J$ = 13.6, 8.1 Hz, 1H), 1.76 (dd, $J$ = 14.1, 8.9 Hz, 1H), 1.67 (dd, $J$ = 14.1, 3.3 Hz, 1H), 0.75 (s, 9H) ppm;

**$^{13}$C NMR** (101 MHz, $CDCl_3$): $\delta_C$ 151.9, 149.1, 129.9, 128.5, 128.5, 128.1 (q, $^2J_{C-F}$ = 32.2 Hz), 125.2 (q, $^3J_{C-}$

$_F$ = 3.8 Hz), 124.6 (q, $^1J_{C-F}$ = 271.7 Hz), 112.9, 49.0, 45.5, 45.2, 41.0, 31.5, 30.3 ppm;

**$^{19}$F NMR** (377 MHz, $CDCl_3$): $\delta_F$ -62.13 ppm.

**IR** (film): $\nu_{max}$ 2952, 1738, 1683, 1616, 1521, 1323, 1161, 1108, 1069, 1017, 833, 607 cm$^{-1}$.

**HRMS** (ESI$^+$): m/z calculated for $C_{22}H_{29}F_3N$ [M+H]$^+$, 364.2247; found, 364.2254.

### 3.6.3. Synthesis of Compound 11

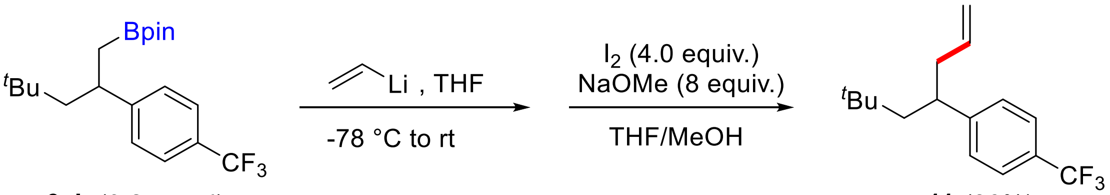


**6ab** (0.2 mmol) **11** (86%)

Following a modified procedure,[14] To a flame-dried Schlenk flask with a magnetic stir bar was added 2-{4,4-dimethyl-2-[4-(trifluoromethyl)phenyl]pentyl}-4,4,5,5-tetramethyl-1,3,2-dioxaborolane (**6ab**) (74.1 mg, 0.20 mmol, 1.0 equiv.) and anhydrous THF (1.0 mL) under $N_2$. The solution was cooled to −78 °C (dry ice/acetone) and vinyl lithium (0.43 M in THF, 200 µL, 0.22 mmol, 1.1 equiv.) was added dropwise. The mixture was allowed to stir for 30 min at −78 °C before removing the cooling bath and then warmed to ambient temperature for another 30 min. Then the reaction mixture was cooled to −78°C and a solution of $I_2$ (203 mg, 0.80 mmol, 4.0 equiv.) in anhydrous THF (1.6 mL) was added dropwise and the resulting mixture was stirred at −78 °C for 20 min. A suspension of NaOMe (86.4 mg, 1.60 mmol, 8.0 equiv.) in MeOH (1.0 mL) was added dropwise, and the resulting mixture was warmed to 0 °C and stirred for 30 min. After that, $Na_2S_2O_3$ sat. (3.0 mL) and $Et_2O$ (6.0 mL) were added. The organic phase was separated, and the aqueous phase was extracted with $Et_2O$ (3*6.0 mL). The combined organic extracts were dried over anhydrous sodium sulfate, filtered and concentrated under reduced pressure. The resulting crude material was purified by flash column chromatography (99:1 pentane/$Et_2O$) to afford the title compound **11** (46.5 mg, 86%) as a colorless oil.

**1-(6,6-Dimethylhept-1-en-4-yl)-4-(trifluoromethyl)benzene (11)**

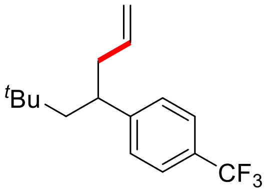


**11**

**TLC**: $R_f$ = 0.72 (98:2 pentane/$Et_2O$, $KMnO_4$ stain)

**NMR Spectroscopy (<u>*see spectra*</u>):**

**$^1$H NMR** (400 MHz, $CDCl_3$): $\delta_H$ 7.52 (d, *J* = 7.7 Hz, 2H), 7.28 (d, *J* = 8.0 Hz, 2H), 5.67 – 5.50 (m, 1H), 4.97

– 4.92 (m, 1H), 4.91 – 4.89 (m, 1H), 2.79 (tdd, $J$ = 8.4, 6.5, 3.5 Hz, 1H), 2.39 – 2.21 (m, 2H), 1.70 (dd, $J$ = 14.1, 8.8 Hz, 1H), 1.62 (dd, $J$ = 14.1, 3.5 Hz, 1H), 0.77 (s, 9H) ppm;

**$^{13}$C NMR** (101 MHz, $CDCl_3$): $\delta_C$ 151.6, 136.6, 128.3, 128.3 (q, $^2J_{C\text{-}F}$ = 32.2 Hz), 125.3 (q, $^3J_{C\text{-}F}$ = 3.7 Hz), 124.5 (q, $^1J_{C\text{-}F}$ = 271.7 Hz), 116.6, 49.4, 44.1, 42.8, 31.5, 30.3 ppm;

**$^{19}$F NMR** (377 MHz, $CDCl_3$): $\delta_F$ -62.21 ppm.

**IR** (film): $\nu_{max}$ 2955, 1738, 1618, 1476, 1420, 1366, 1323, 1162, 1122, 1069, 1018, 914, 835, 605 cm$^{-1}$.

**HRMS** (EI$^+$): m/z calculated for $C_{16}H_{22}F_2$ [M-F]$^+$, 251.1606; found, 251.1603.

### 3.6.4. Synthesis of Compound 12

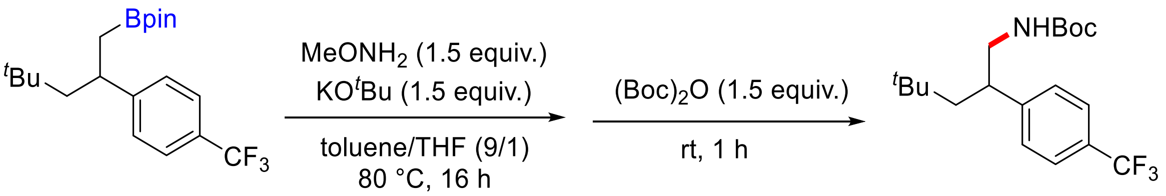


**6ba** (0.2 mmol) **12** (81%)

Following a known procedure,[15] To a flame-dried Schlenk flask with a magnetic stir bar was added potassium *tert*-butoxide (33.7 mg, 0.30 mmol, 1.5 equiv.), methoxyamine (0.16 mL, 1.94 M in THF, 0.30 mmol, 1.5 equiv.) (prepared according to the literature)[15], and anhydrous toluene under $N_2$. Then 2-{4,4-dimethyl-2-[4-(trifluoromethyl)phenyl]pentyl}-4,4,5,5-tetramethyl-1,3,2-dioxaborolane (**6ab**) (74.1 mg, 0.20 mmol, 1.0 equiv.) was added as a solution in toluene (1.4 mL). The solution was stirred for 16 hours at 80 °C. After that, the reaction mixture was cooled to room temperature and $Boc_2O$ (0.30 mL, 1.0 M in THF, 0.3 mmol, 1.5 equiv.) was added. The resulting mixture then was stirred for 1 h at room temperature. After the reaction, $Et_2O$ (5.0 mL) and water (5.0 mL) was added. The organic phase was separated, and the aqueous phase was extracted with $Et_2O$ (3*5.0 mL). The combined organic extracts were dried over anhydrous sodium sulfate, filtered and concentrated under reduced pressure. The resulting crude material was purified by flash column chromatography (89:11 pentane/$Et_2O$) to afford the title compound **12** (58 mg, 81%) as a white solid.

**_tert_-Butyl {4,4-dimethyl-2-[4-(trifluoromethyl)phenyl]pentyl}carbamate (12)**

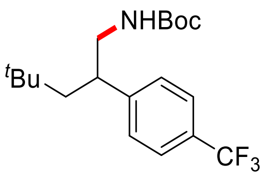


**12**

**TLC**: $R_f$ = 0.45 (86:14 *n*-hexane/EtOAc, $KMnO_4$ stain)

**NMR Spectroscopy (*see spectra*):**

**$^{1}$H NMR** (400 MHz, $CDCl_3$): $\delta_H$ 7.55 (d, $J$ = 7.8 Hz, 2H), 7.31 (d, $J$ = 7.9 Hz, 2H), 4.36 (brs, 1H), 3.46 – 3.29 (m, 1H), 3.06 (ddd, $J$ = 13.6, 9.1, 5.1 Hz, 1H), 2.95 (d, $J$ = 8.2 Hz, 1H), 1.68 (dd, $J$ = 14.1, 8.3 Hz, 1H), 1.54 (dd, $J$ = 14.1, 3.3 Hz, 1H), 1.38 (s, 9H), 0.80 (s, 9H) ppm;

**$^{13}$C NMR** (101 MHz, $CDCl_3$): $\delta_C$ 155.9, 149.3, 128.9 (q, $^2J_{C\text{-}F}$ = 32.5 Hz), 128.6, 125.6 (q, $^3J_{C\text{-}F}$ = 3.9 Hz), 124.4 (q, $^1J_{C\text{-}F}$ = 271.9 Hz), 79.4, 48.1, 47.1, 42.9, 31.4, 30.1, 28.5 ppm;

**$^{19}$F NMR** (377 MHz, $CDCl_3$): $\delta_F$ -62.35 ppm.

**IR** (film): $\nu_{max}$ 3358, 2957, 1699, 1506, 1366, 1324, 1250, 1161, 1123, 1111, 1067, 1018, 836, 733, 608 cm$^{-1}$.

**HRMS** (ESI$^+$): m/z calculated for $C_{19}H_{28}F_3NNaO_2$ [M+Na]$^+$, 382.1964; found, 382.1974.

### 3.6.5. Synthesis of Compound 13

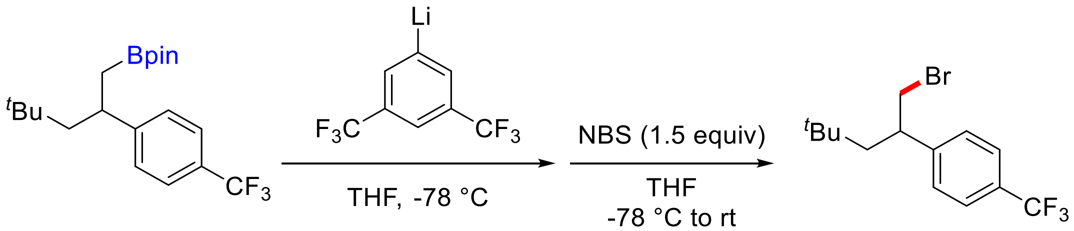


**6ba** (0.2 mmol.) **13** (91%)

Following a known procedure,[17] To a flame-dried Schlenk flask with a magnetic stir bar was added 1-bromo-3,5-bis(trifluoromethyl)benzene (64.5 mg, 0.22 mmol, 1.1 equiv.) and anhydrous THF (2 mL). The solution was cooled to −78 °C (dry ice/acetone) and $^n$BuLi (1.6 M in hexanes, 138 µL, 0.22 mmol, 1.1 equiv.) was added dropwise. The mixture was allowed to stir for 1 h at −78 °C before the solution of 2-{4,4-dimethyl-2-[4-(trifluoromethyl)phenyl]pentyl}-4,4,5,5-tetramethyl-1,3,2-dioxaborolane (**6ab**) (74.1 mg, 0.20 mmol, 1.0 equiv.) in THF (1.0 mL) was added dropwise. The mixture was allowed to stir for 30 min at −78 °C before removing the cooling bath and warmed to room temperature for another 30 min. Then, a solution of NBS (53.4 mg, 0.3 mmol, 1.5 equiv.) in THF (2 mL) was added dropwise, and the reaction mixture was stirred for 1 h. After that, $Na_2S_2O_3$ sat. (3.0 mL) and $Et_2O$ (6.0 mL) were added. The organic phase was separated, and the aqueous phase was extracted with $Et_2O$ (3*10.0 mL). The combined organic extracts were dried over anhydrous sodium sulfate, filtered and concentrated under reduced pressure. The resulting crude material was purified by flash column chromatography (99:1 pentane/$Et_2O$) to afford the title compound **13** (58.8 mg, 91%) as a white solid.

**1-(1-Bromo-4,4-dimethylpentan-2-yl)-4-(trifluoromethyl)benzene (13)**

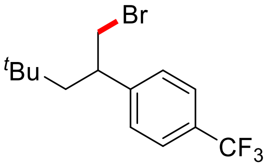


**13**

**TLC**: $R_f$ = 0.58 (98:2 pentane/$Et_2O$, $KMnO_4$ stain)

**NMR Spectroscopy (*see spectra*):**

**$^{1}$H NMR** (400 MHz, $CDCl_3$): 7.58 (d, $J$ = 8.5 Hz, 2H), 7.34 (d, $J$ = 8.0 Hz, 2H), 3.51 (dd, $J$ = 9.9, 6.6 Hz, 1H), 3.44 (dd, $J$ = 9.9, 7.6 Hz, 1H), 3.13 (tdd, $J$ = 7.6, 6.6, 3.5 Hz, 1H), 1.82 – 1.77 (m, 2H), 0.82 (s, 9H) ppm;

**$^{13}$C NMR** (101 MHz, $CDCl_3$): $\delta_C$ 148.5, 129.3 (q, $^{2}J_{C-F}$ = 32.4 Hz), 128.4, 125.6 (q, $^{3}J_{C-F}$ = 3.8 Hz), 124.3 (q, $^{1}J_{C-F}$ = 272.1 Hz), 48.1, 45.2, 40.0, 31.4, 30.1 ppm;

**$^{19}$F NMR** (377 MHz, $CDCl_3$): $\delta_F$ -62.38 ppm.

**IR** (film): $\nu_{max}$ 2958, 1738, 1619, 1419, 1367, 1323, 1163, 1122, 1069, 833, 734, 607 cm$^{-1}$.

**HRMS** (EI$^+$): m/z calculated for $C_{14}H_{18}F_3Br$ [M]$^+$, 322.0538; found, 322.0534.

### 3.6.6. Synthesis of Compound 14

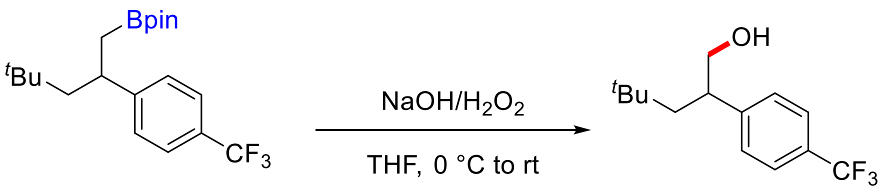


**6ab** (0.2 mmol) **14** (94%)

To a 50 mL round flask with a magnetic stir bar was added 2-{4,4-dimethyl-2-[4-(trifluoromethyl)phenyl]pentyl}-4,4,5,5-tetramethyl-1,3,2-dioxaborolane (**6ab**) (74.1 mg, 0.20 mmol, 1.0 equiv.) and THF (3.0 mL). The reaction mixture was cooled to 0 °C. An aqueous solution of sodium hydroxide (2.0 M, 1.0 mL) and aqueous hydrogen peroxide (30%, 1.0 mL) were added dropwise. After 10 min, the vigorously stirred biphasic reaction mixture was allowed to reach ambient temperature and stirred for another 30 min, at which point TLC analysis showed the disappearance of the boronic ester. The mixture was diluted with water (5.0 mL) and ethyl acetate (10 mL). The organic phase was separated, and the aqueous phase was extracted with ethyl acetate (3*10 mL). The combined organic extracts were dried over anhydrous sodium sulfate, filtered and concentrated under reduced pressure. The resulting crude material was purified by flash column chromatography (86:14 *n*-hexane/EtOAc) to afford the title compound **13** (49 mg, 94%) as a colorless oil.

### 4,4-Dimethyl-2-[4-(trifluoromethyl)phenyl]pentan-1-ol (14)

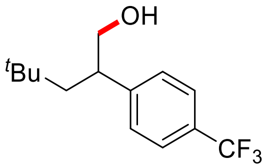


**14**

**TLC**: $R_f$ = 0.45 (80:20 *n*-hexane/EtOAc, $KMnO_4$ stain)

**NMR Spectroscopy (*see spectra*):**

**$^1$H NMR** (400 MHz, $CDCl_3$): $\delta_H$ 7.57 (d, $J$ = 7.6 Hz, 2H), 7.34 (d, $J$ = 7.9 Hz, 2H), 4.00 (ddt, $J$ = 8.2, 6.4, 4.6 Hz, 1H), 2.81 (dd, $J$ = 13.5, 4.7 Hz, 1H), 2.74 (dd, $J$ = 13.5, 8.2 Hz, 1H), 1.47 – 1.43 (m, 2H), 1.42 (brs, 1H), 0.96 (s, 9H) ppm;

**$^{13}$C NMR** (101 MHz, $CDCl_3$): $\delta_C$ 143.1, 130.0, 129.0 (q, $^2J_{C\text{-}F}$ = 32.3 Hz), 125.5 (q, $^3J_{C\text{-}F}$ = 3.8 Hz), 124.4 (q, $^1J_{C\text{-}F}$ =271.8 Hz), 70.4, 50.7, 45.9, 30.5, 30.2 ppm;

**$^{19}$F NMR** (377 MHz, $CDCl_3$): $\delta_F$ -62.41 ppm.

**IR** (film): $v_{max}$ 3414, 2953, 1739, 1618, 1418, 1366, 1322, 1162, 1122, 1108, 1066, 1019, 820, 636, 597 $cm^{-1}$.

**HRMS** (ESI$^+$): m/z calculated for $C_{14}H_{19}F_3NaO$ [M+Na]$^+$, 283.1280; found, 283.1277.

# 4. SPECTROSCOPIC DATA

$^{1}H$ NMR (400 MHz, $CDCl_3$) of **1b** (see procedure)

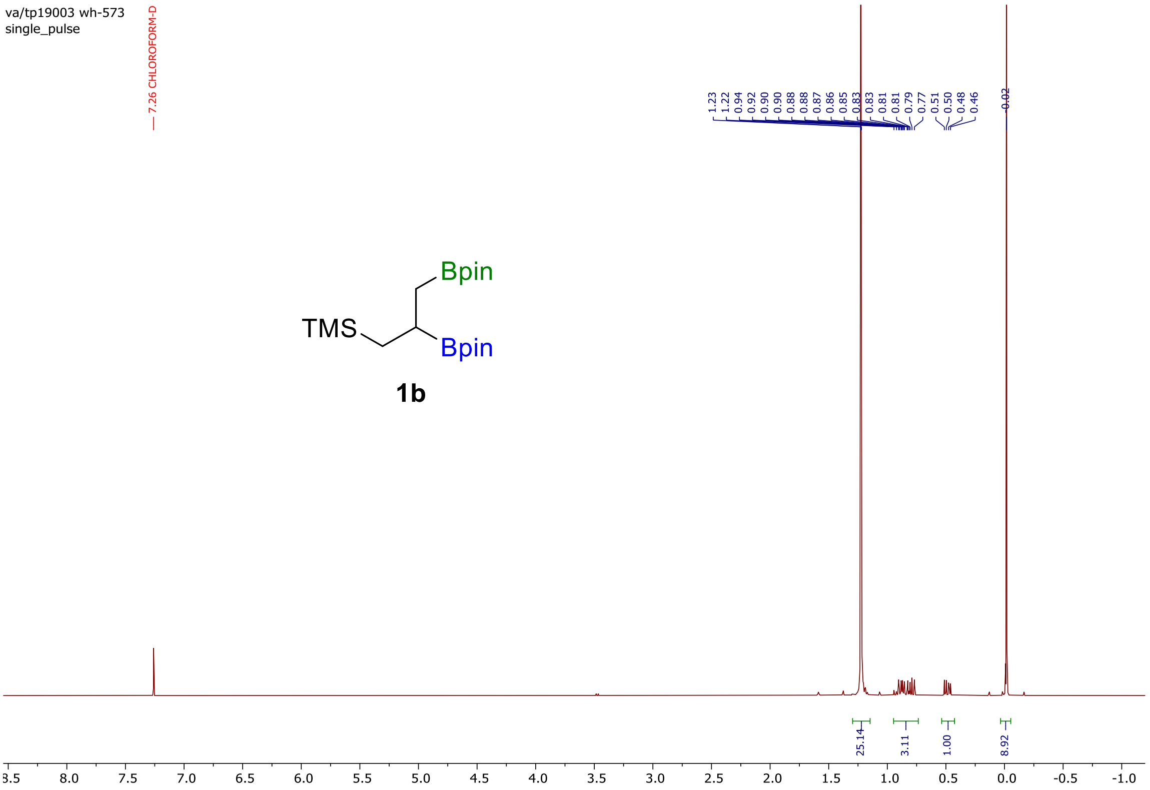


$^{13}C$ NMR (101 MHz, $CDCl_3$) of **1b**

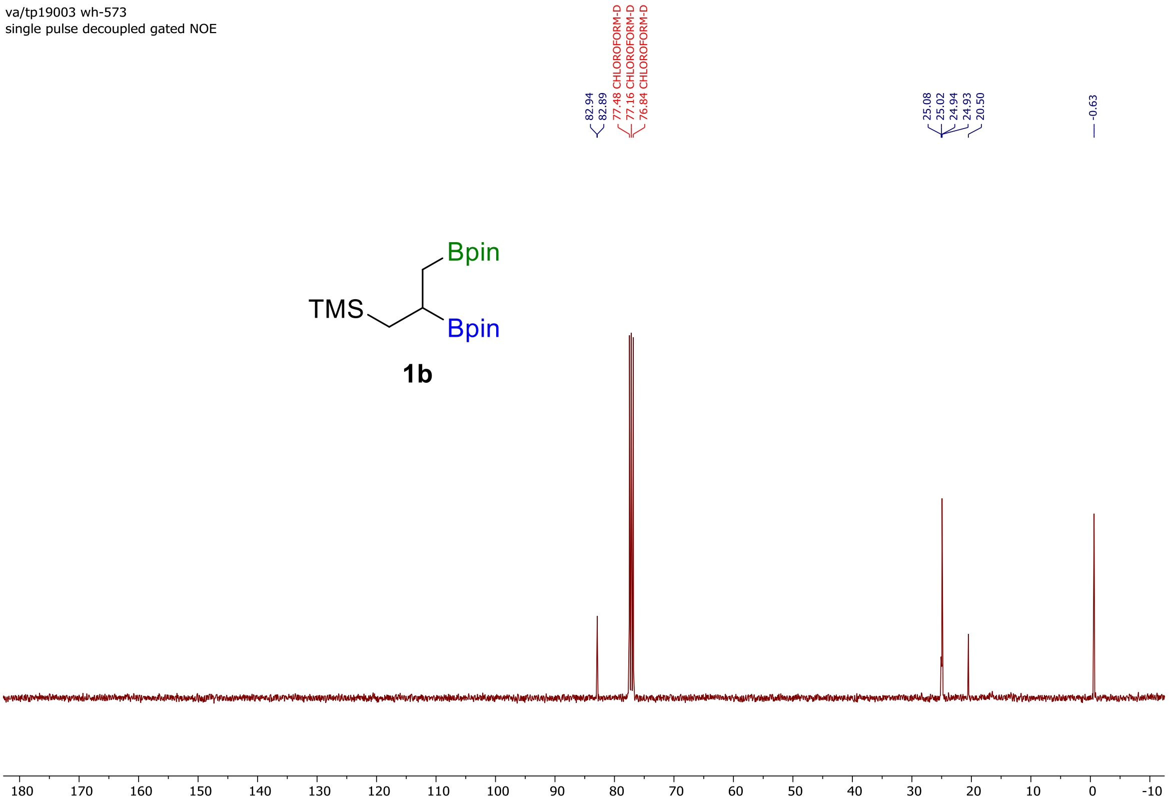

## $^{11}$B NMR (128 MHz, $CDCl_3$) of **1b**

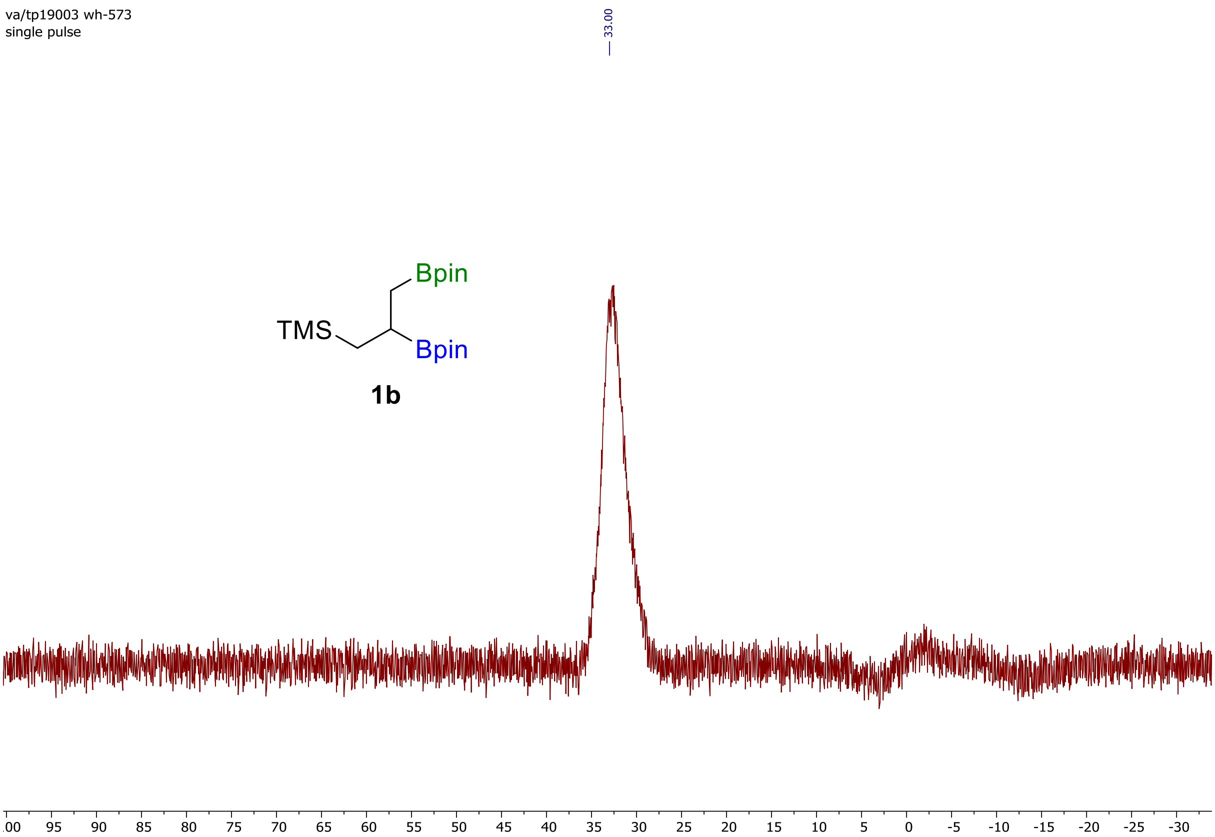


## $^{1}$H NMR (400 MHz, $CDCl_3$) of **1d** (*see procedure*)

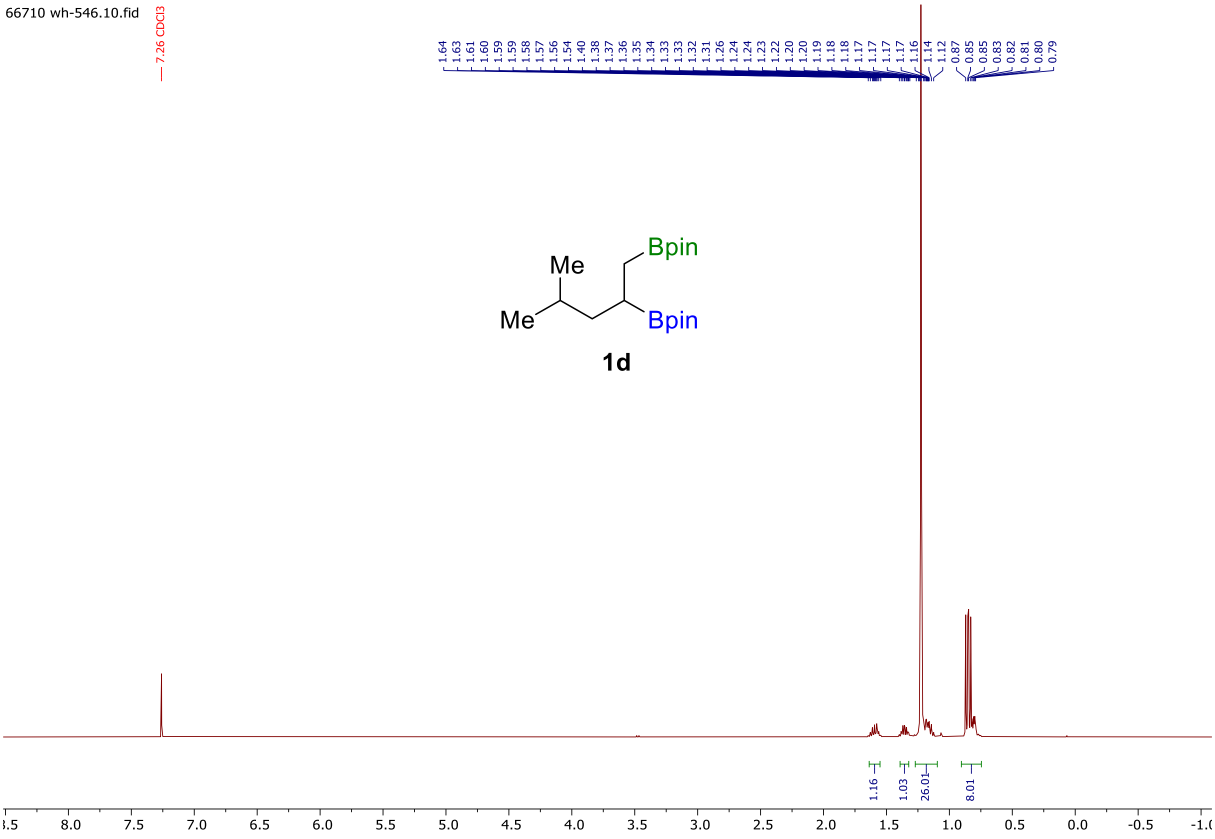

$^{13}$C NMR (101 MHz, $CDCl_3$) of **1d**

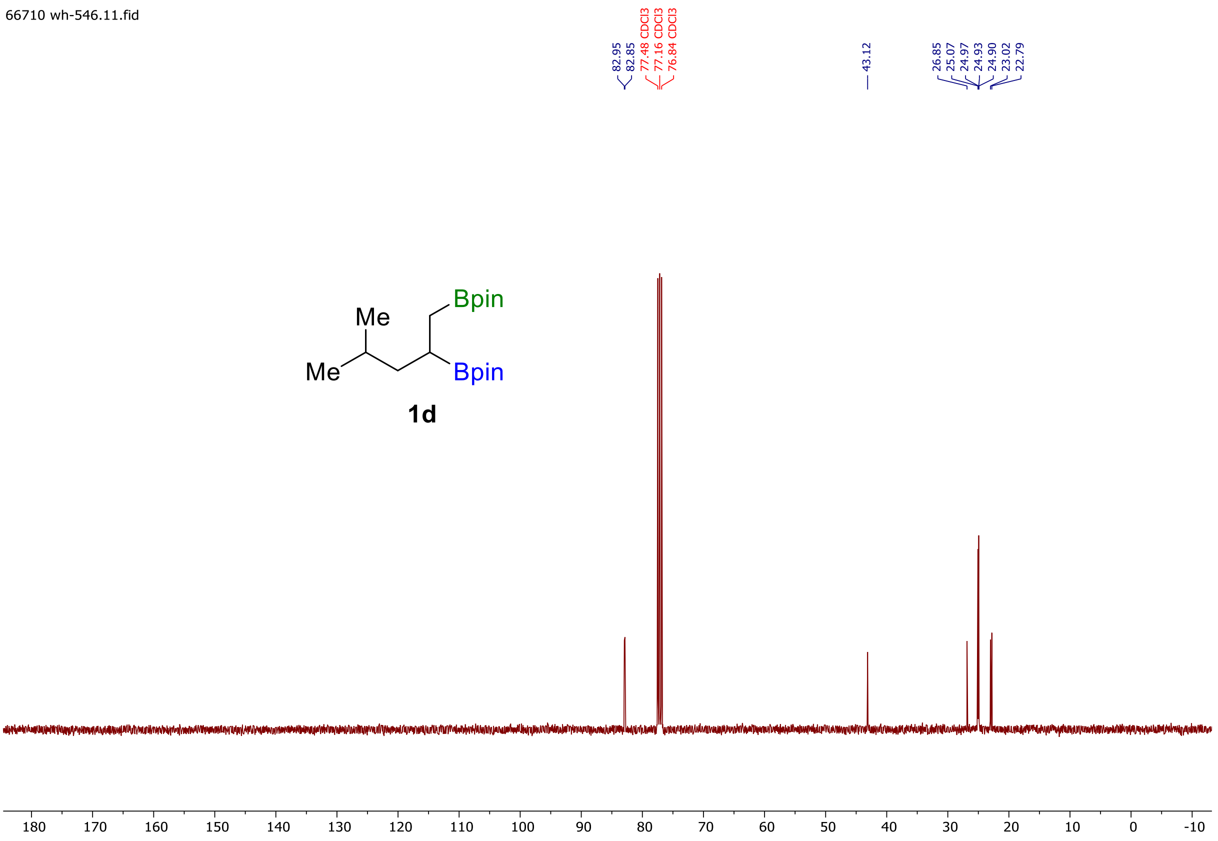


$^{11}$B NMR (128 MHz, $CDCl_3$) of **1d**

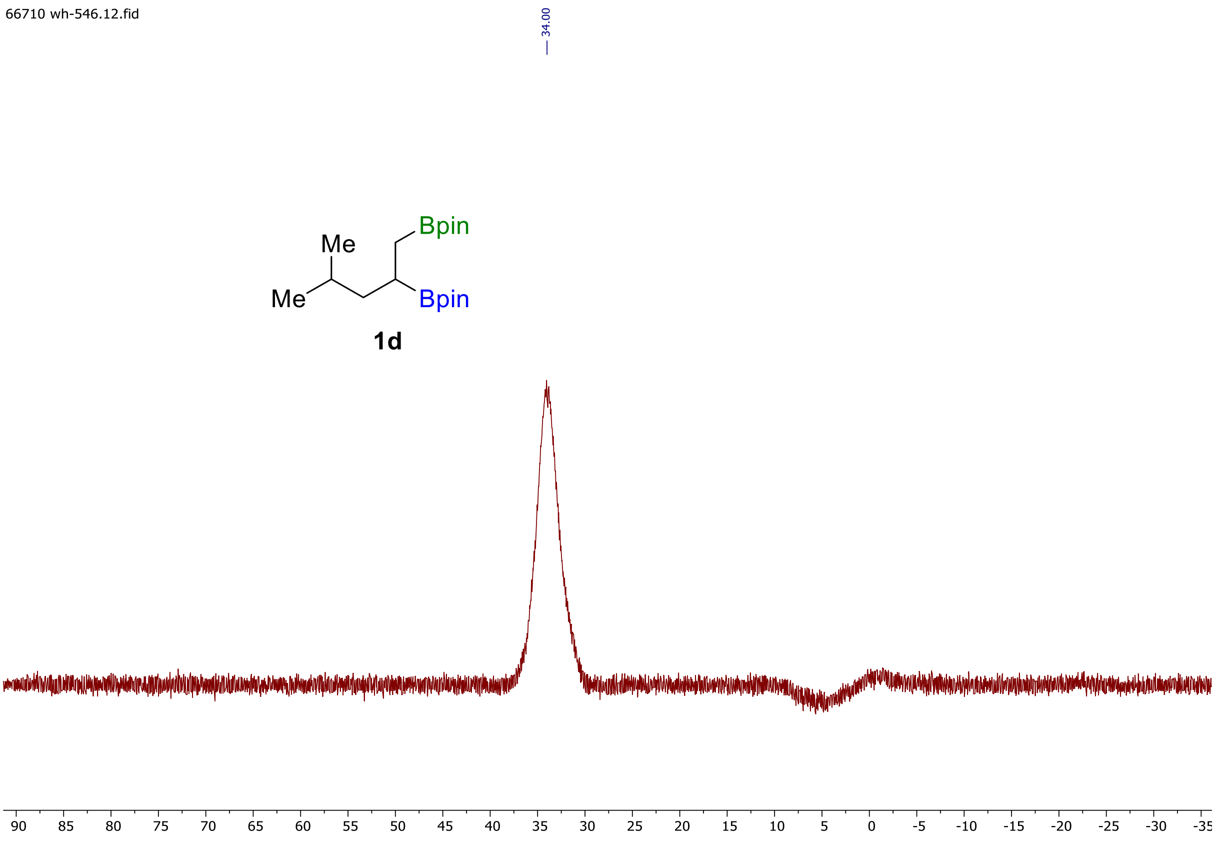

$^{1}H$ NMR (400 MHz, $CDCl_3$) of **1e** (see procedure)

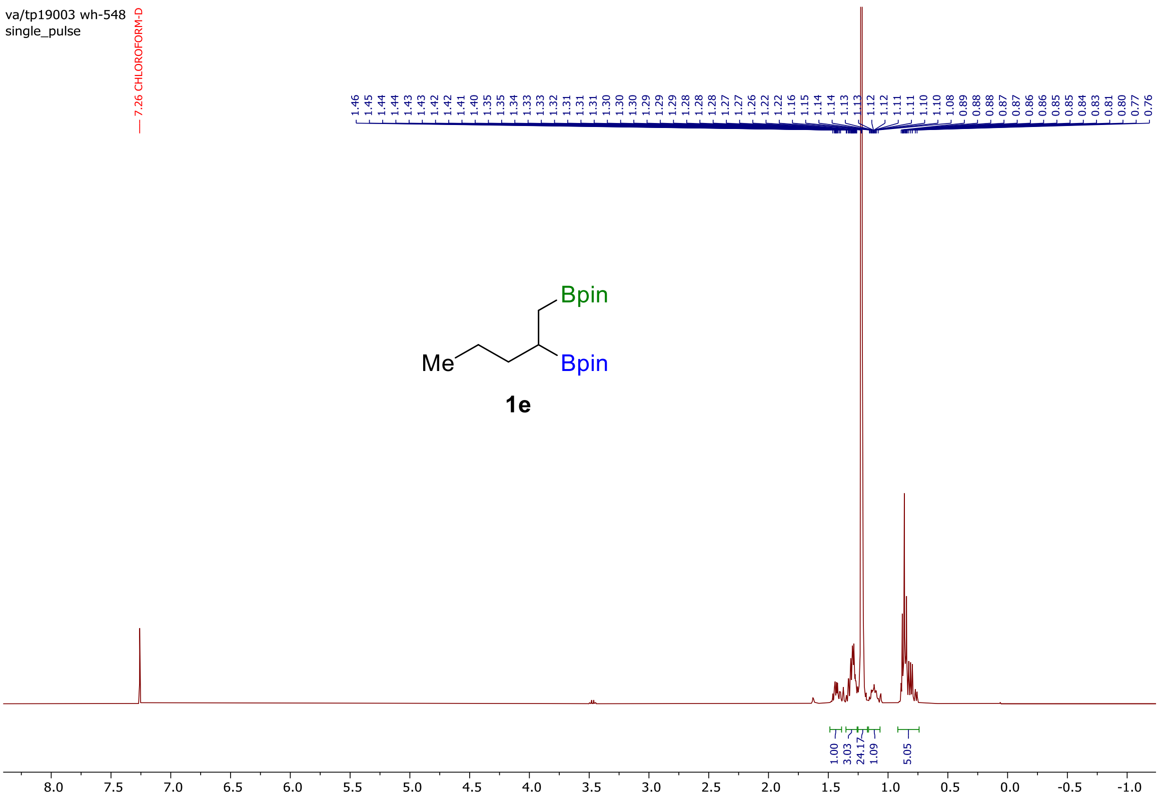

$^{13}C$ NMR (101 MHz, $CDCl_3$) of **1e**

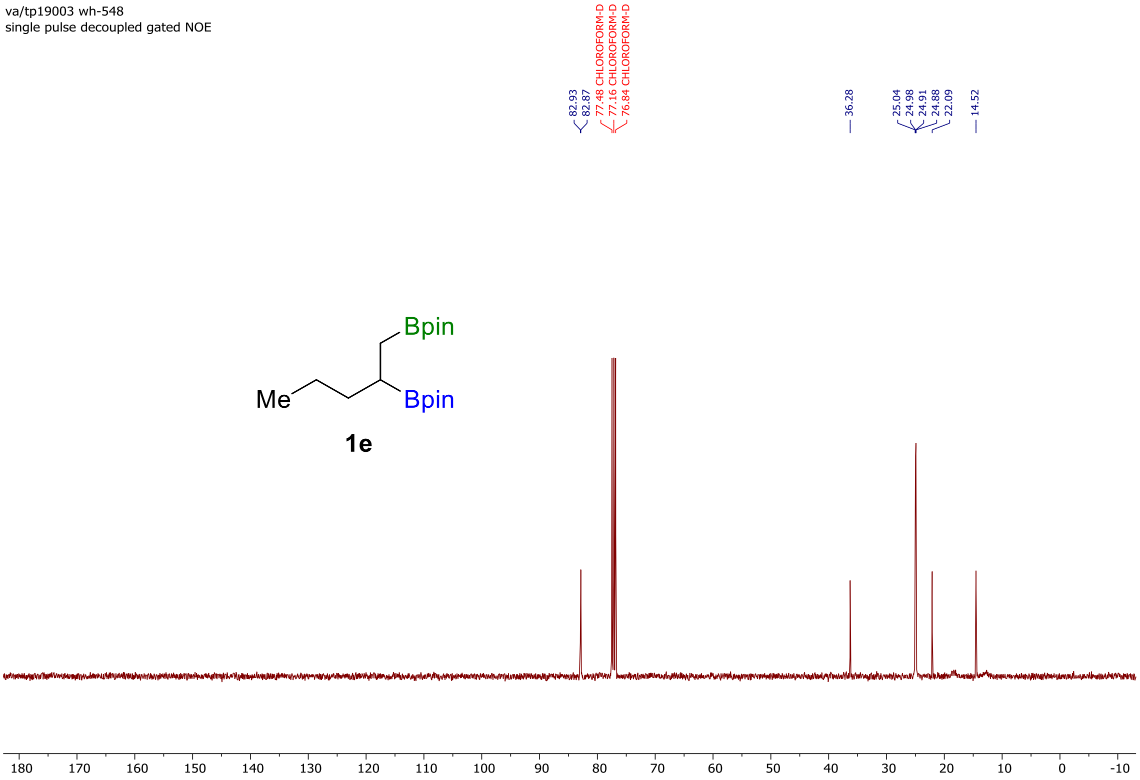

$^{11}B$ NMR (128 MHz, $CDCl_3$) of **1e**

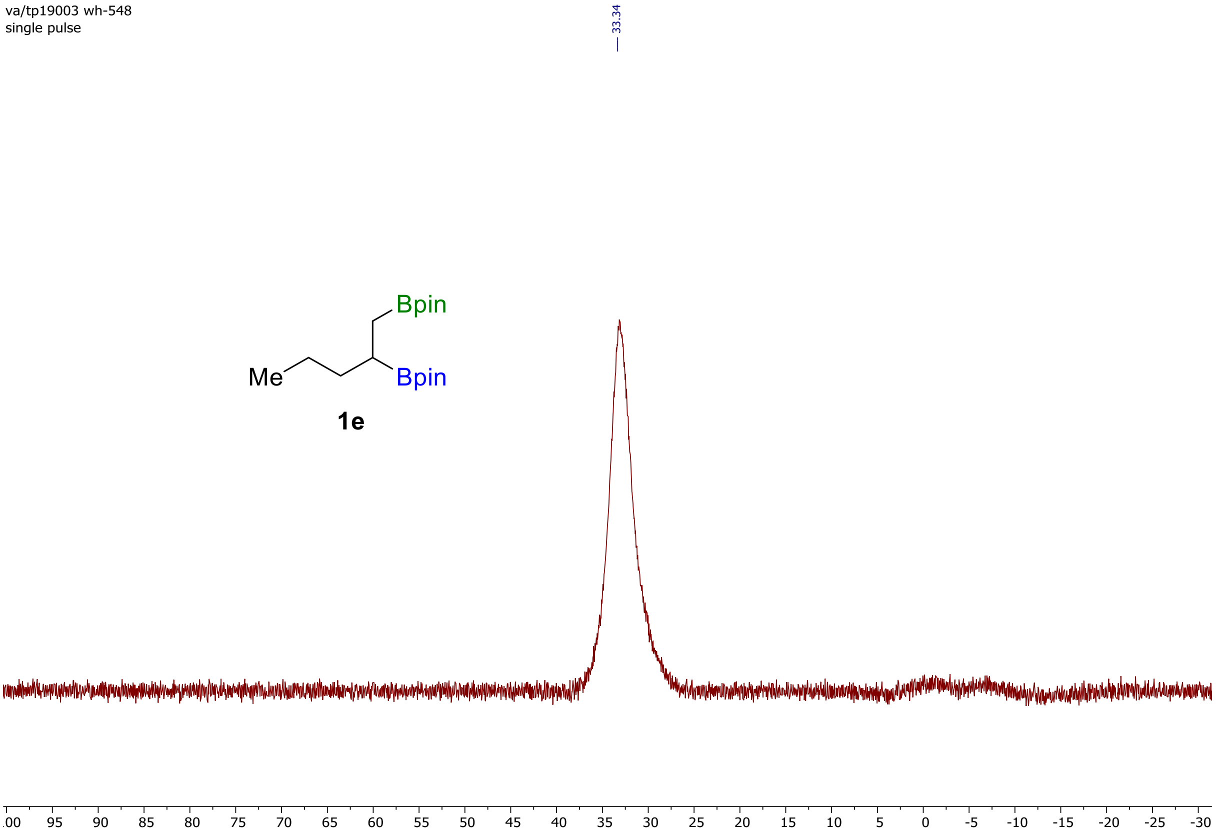


$^{1}H$ NMR (400 MHz, $CDCl_3$) of **1e'** (*see procedure*)

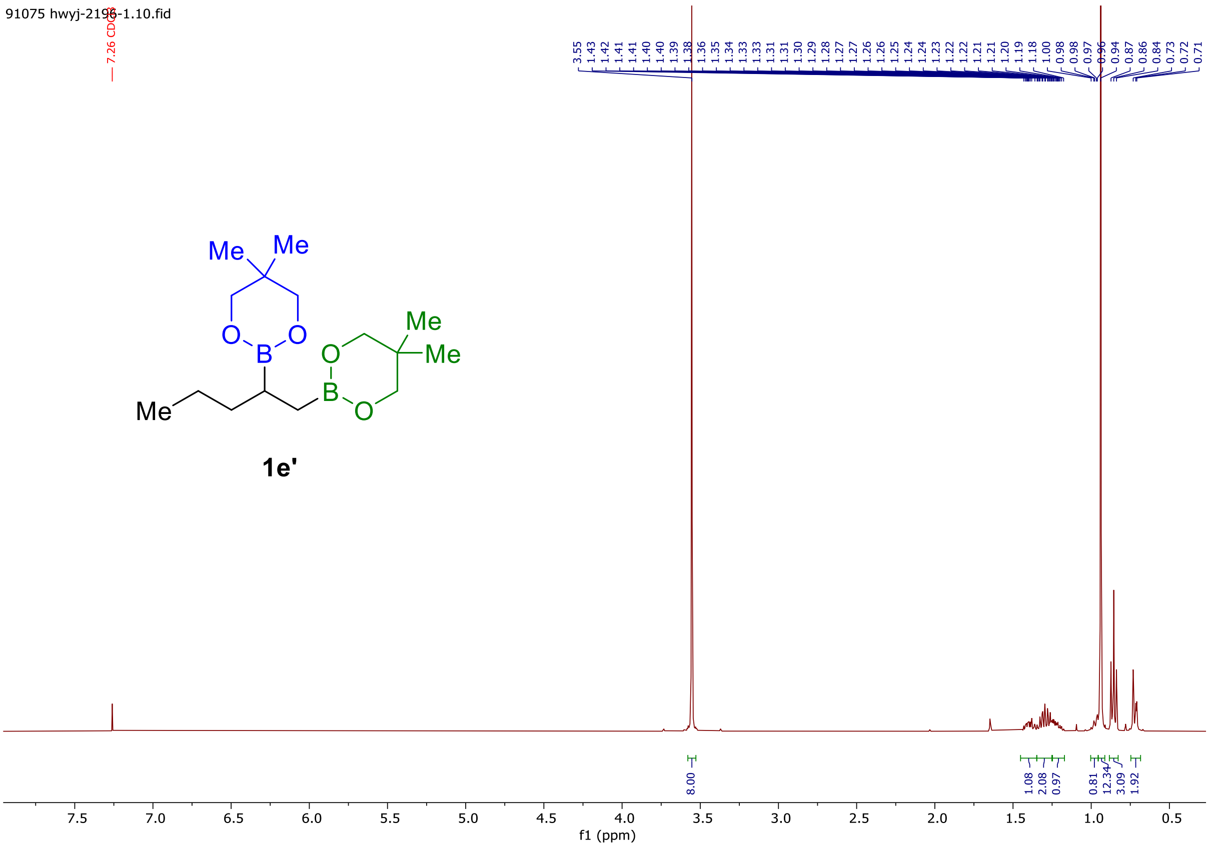

$^{13}$C NMR (101 MHz, $CDCl_3$) of **1e′**

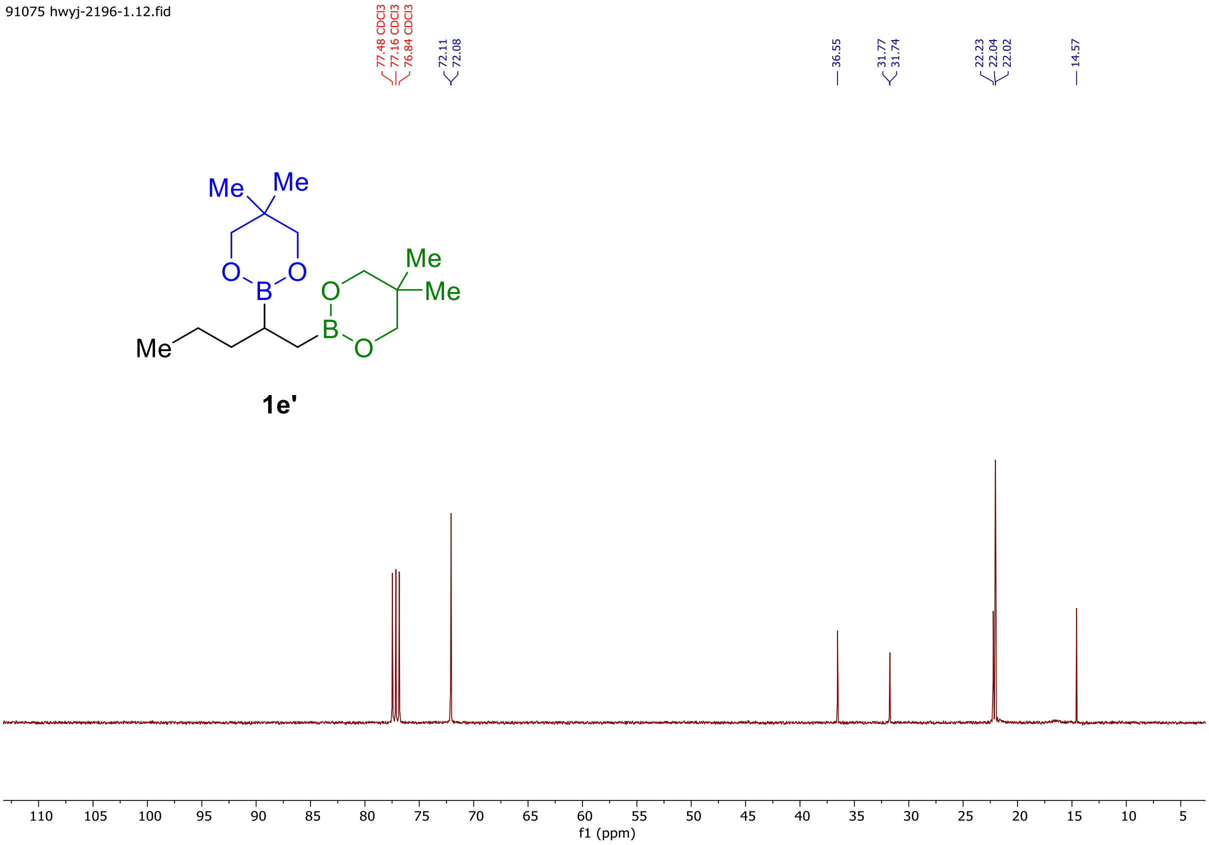


$^{11}$B NMR (128 MHz, $CDCl_3$) of **1e′**

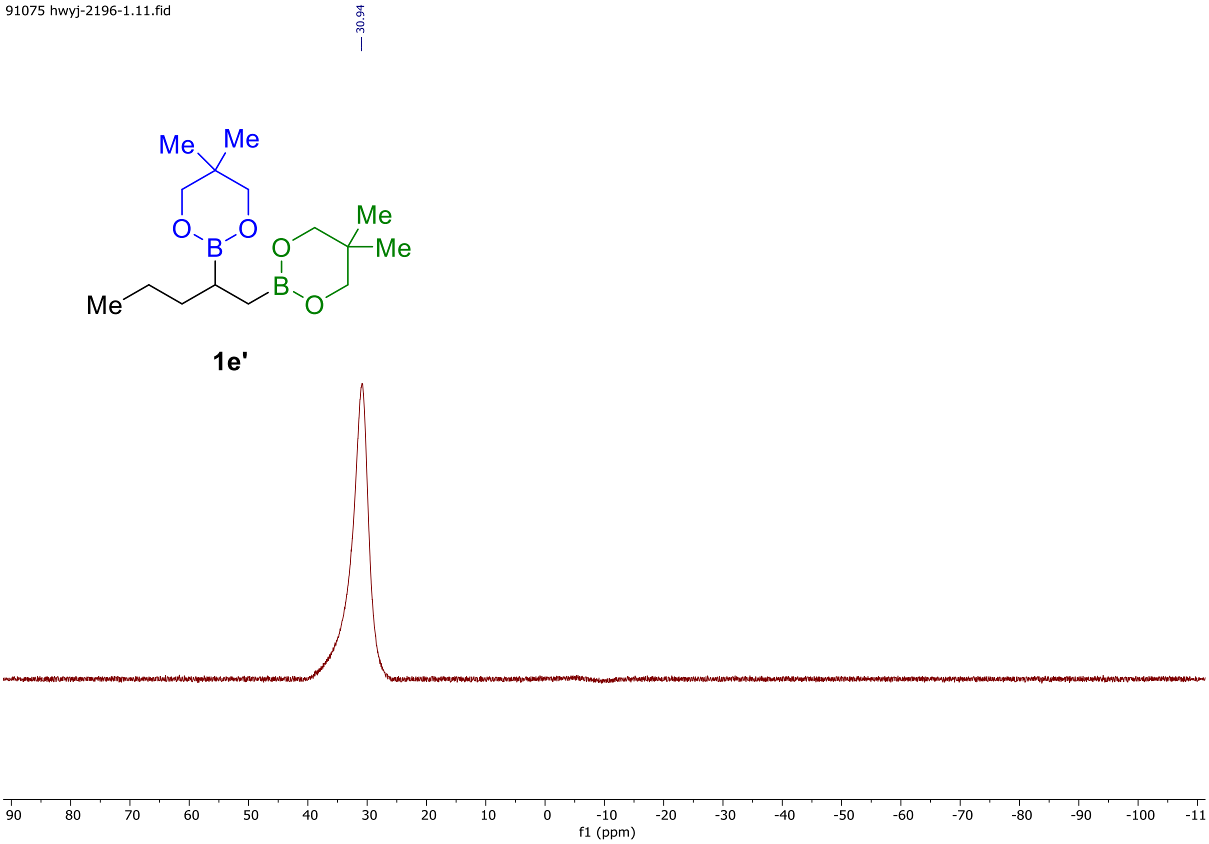

$^{1}H$ NMR (400 MHz, $CDCl_3$) of **1g** (*see procedure*)

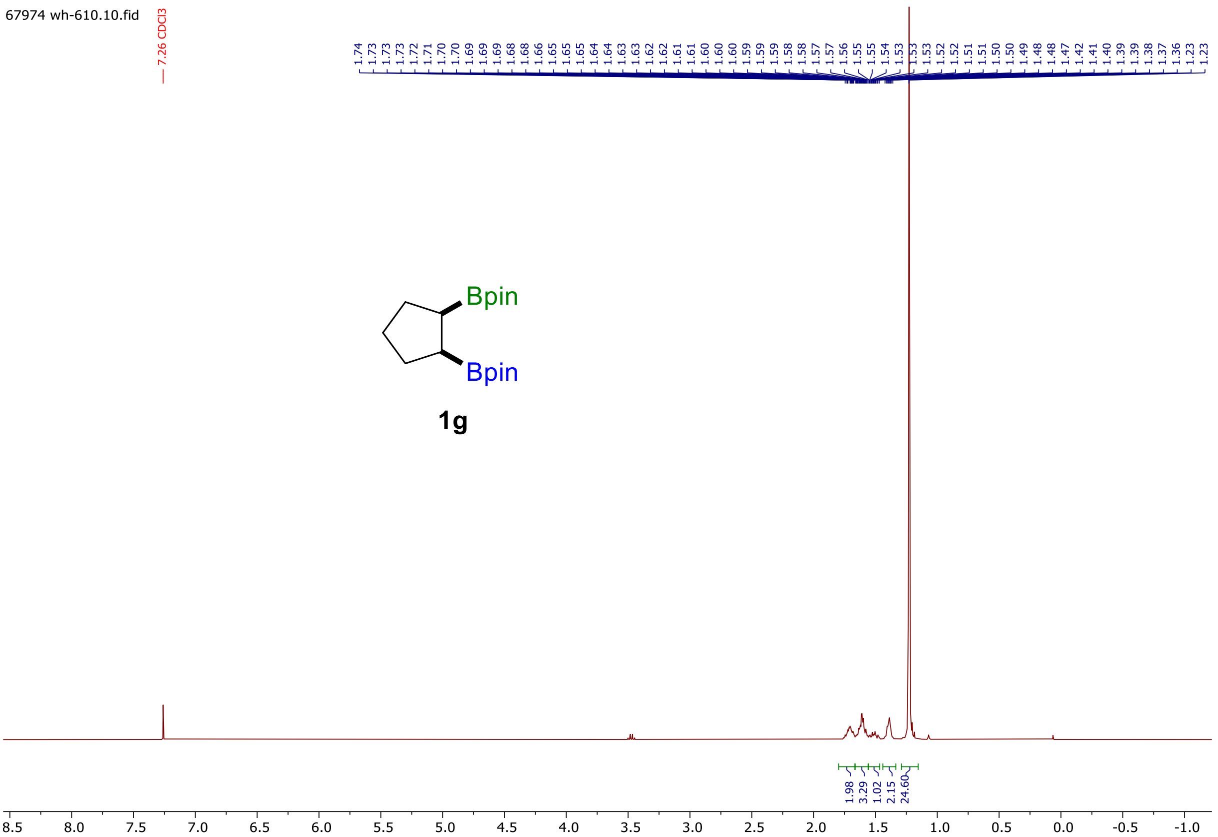


$^{13}C$ NMR (101 MHz, $CDCl_3$) of **1g**

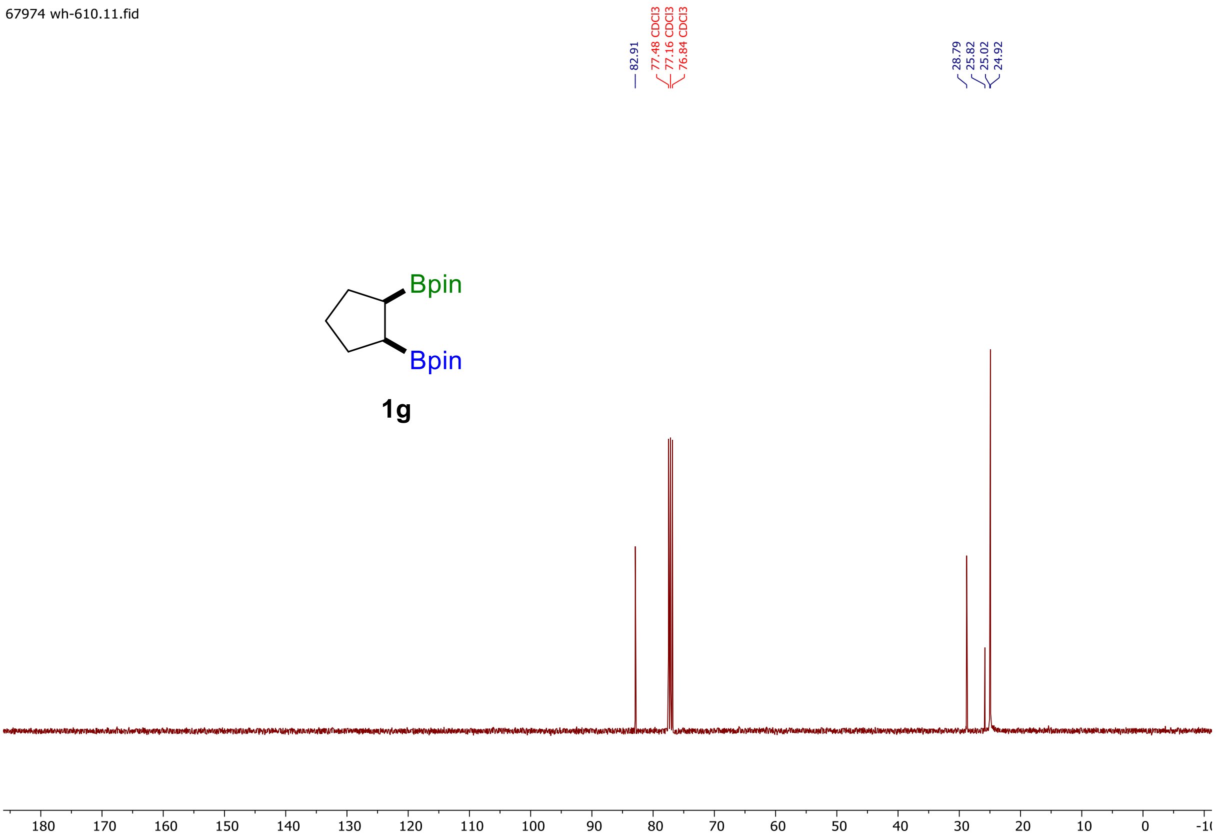

$^{11}$B NMR (128 MHz, $CDCl_3$) of **1g**

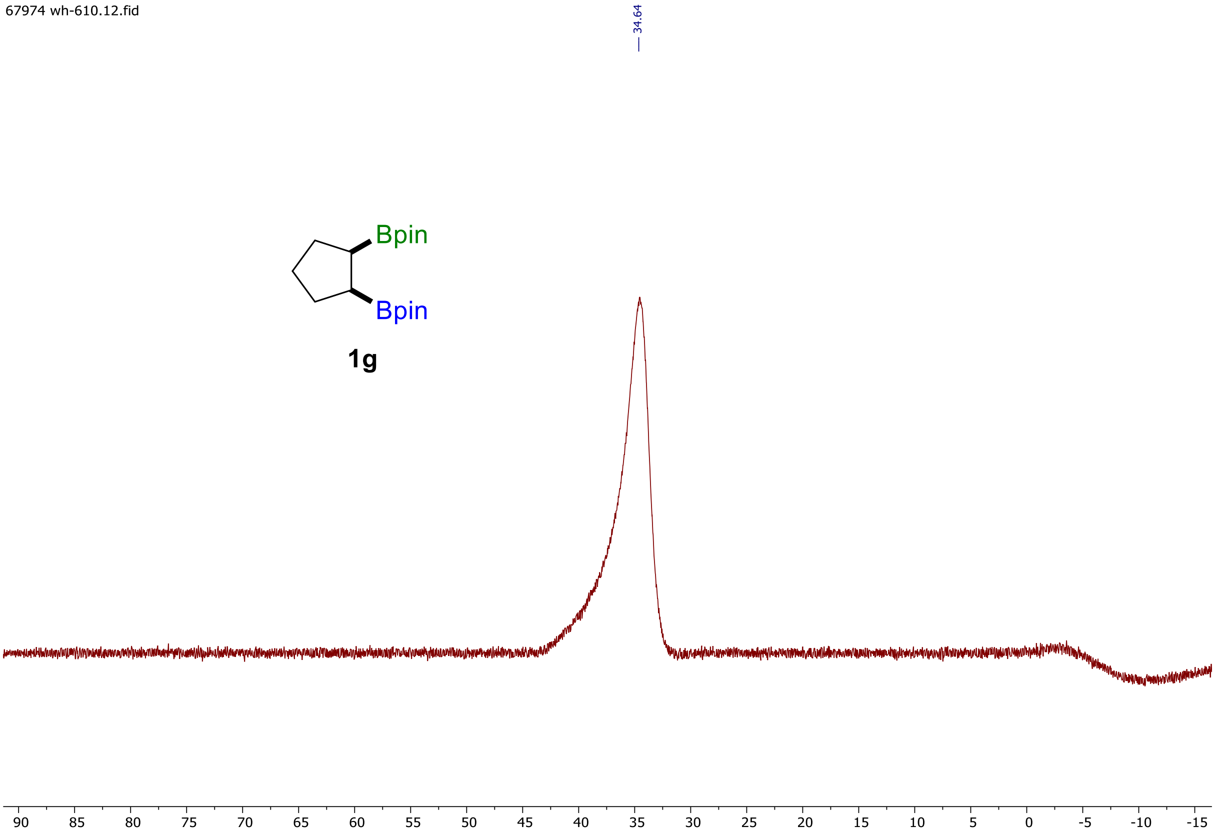


$^{1}$H NMR (400 MHz, $CDCl_3$) of **1h** (*see procedure*)

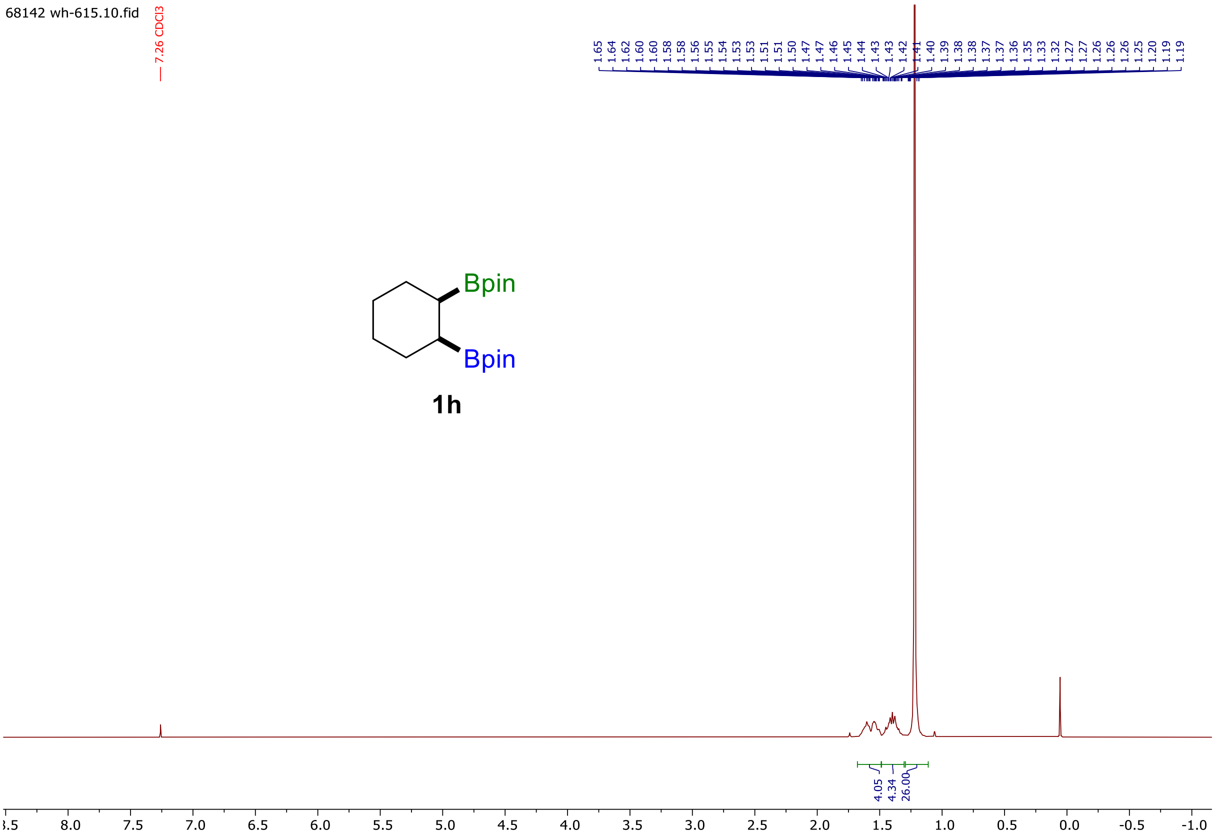

$^{13}$C NMR (101 MHz, $CDCl_3$) of **1h**

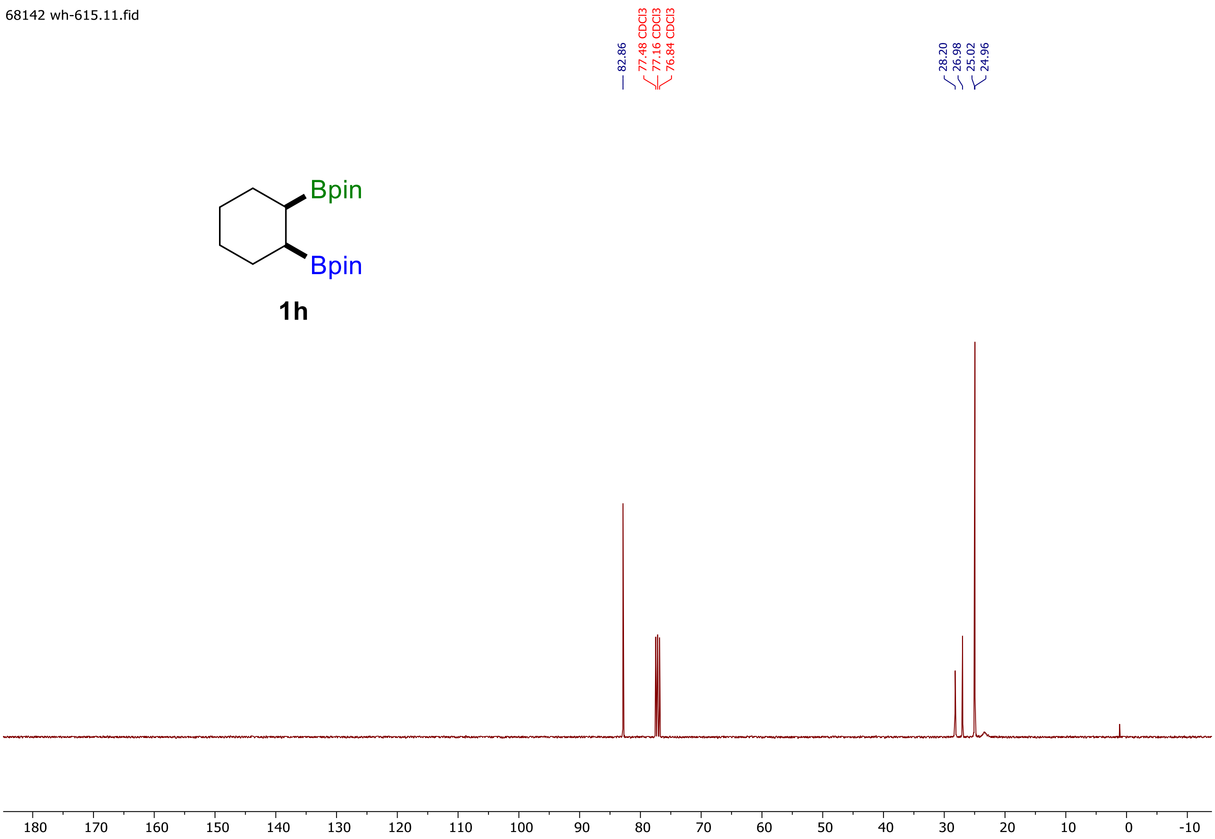


$^{11}$B NMR (128 MHz, $CDCl_3$) of **1h**

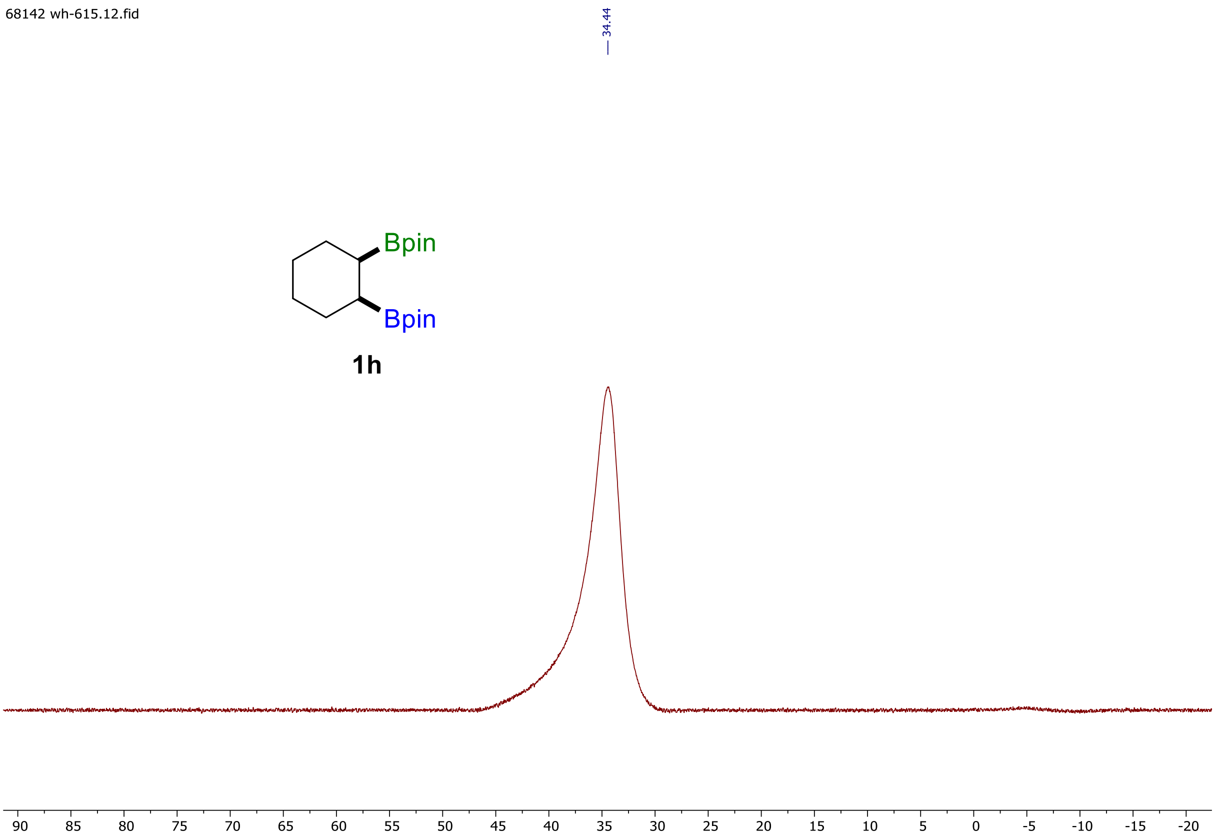

$^1$H NMR (400 MHz, $CDCl_3$) of **1i** (*see procedure*)

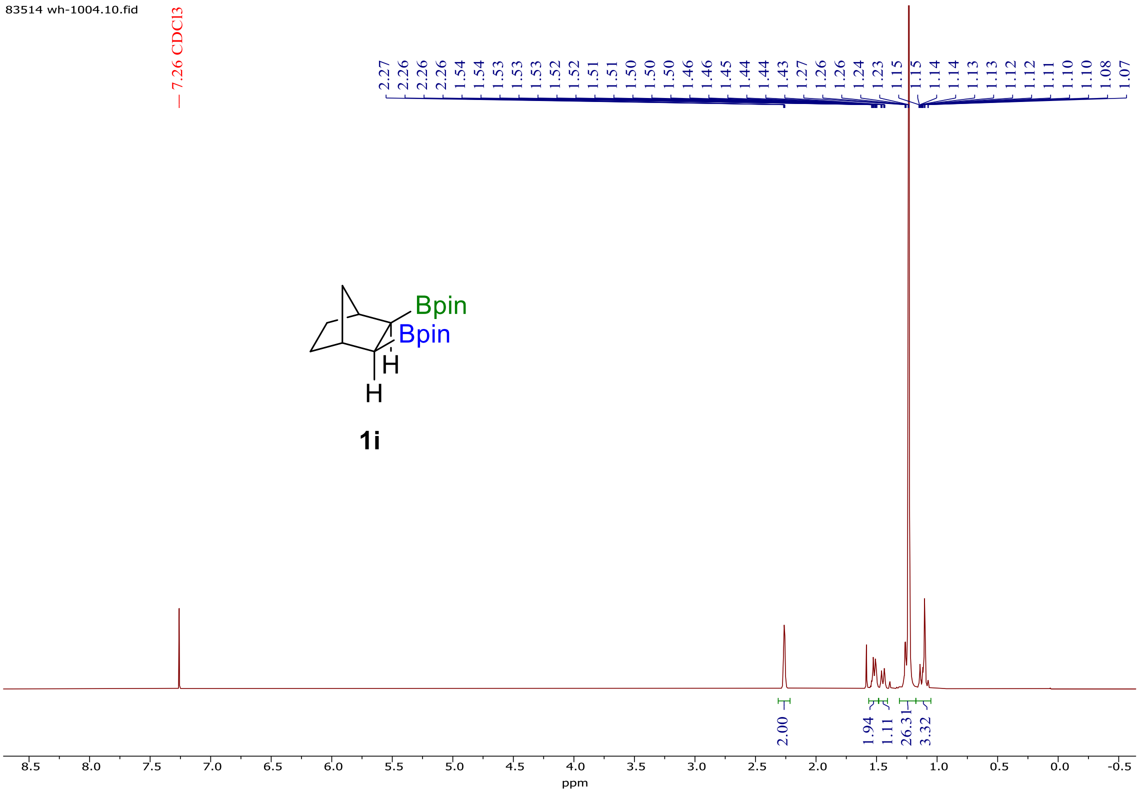


*NOESY* (400 MHz, $CDCl_3$) of **1i**

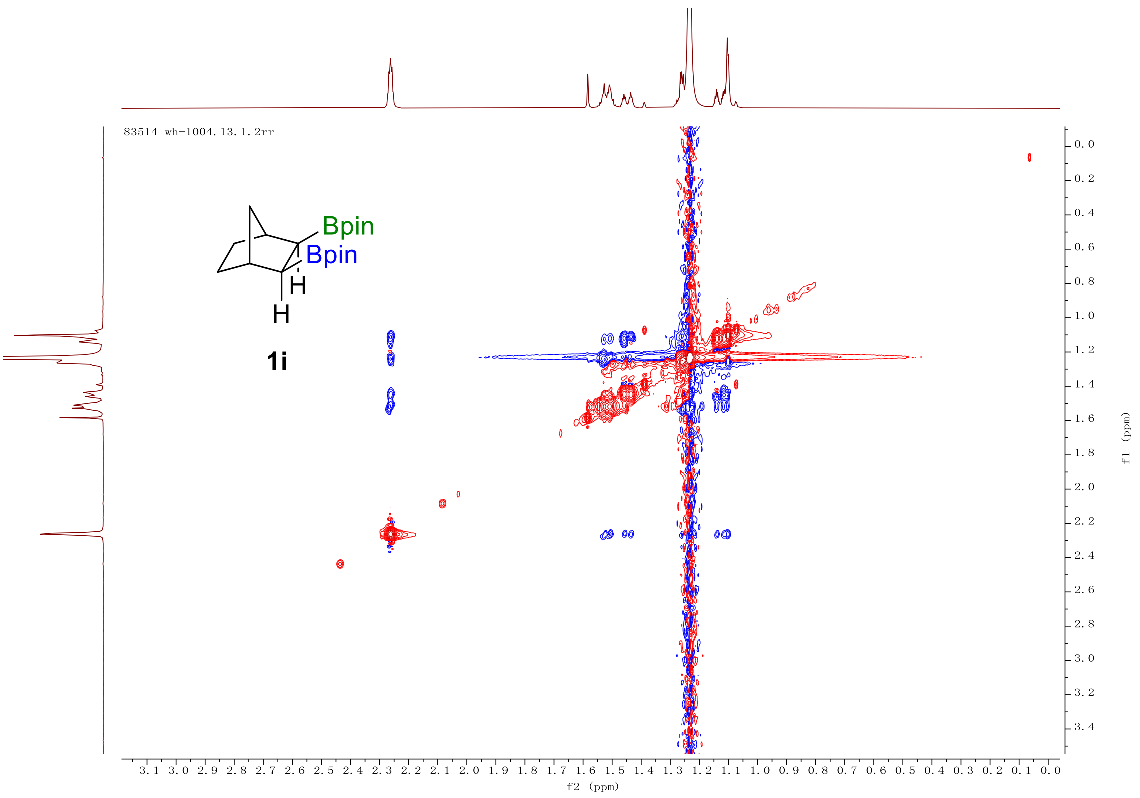

$^{13}C$ NMR (101 MHz, $CDCl_3$) of **1i**

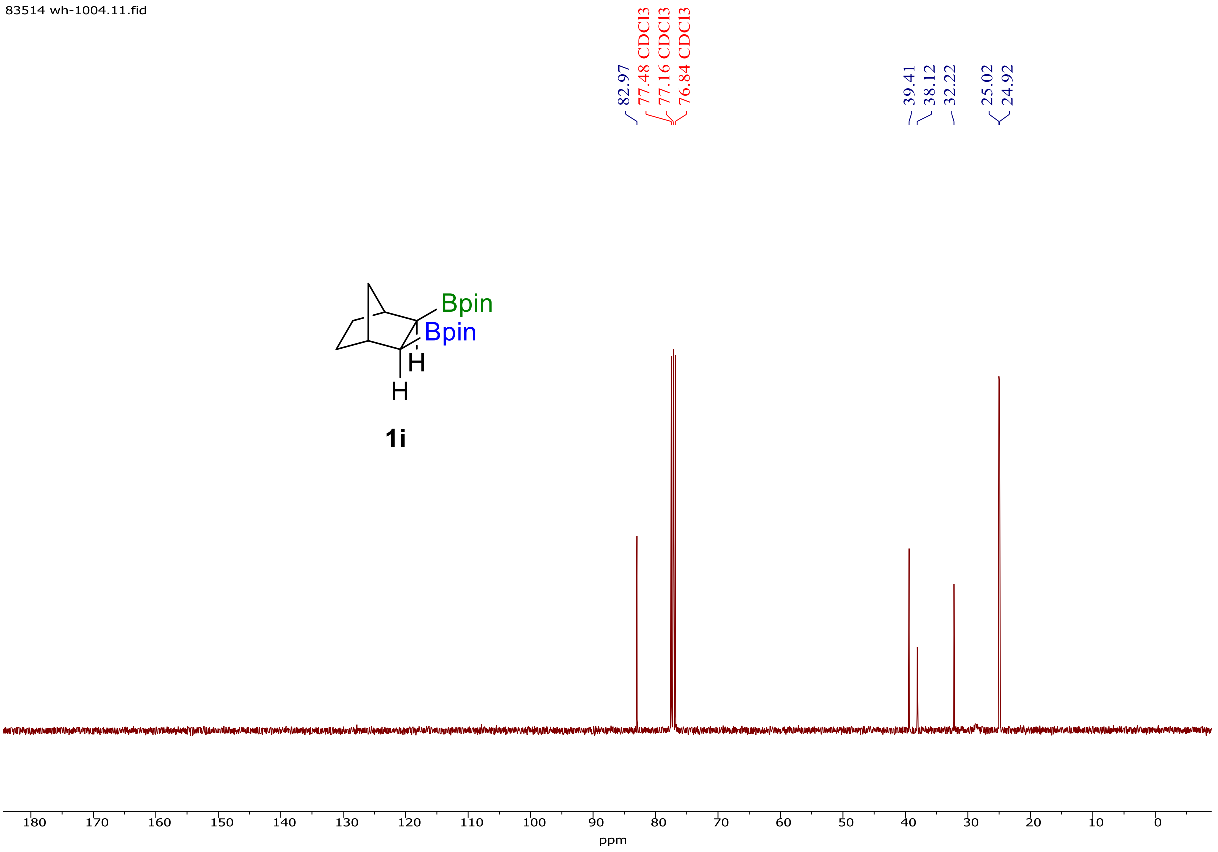


$^{11}B$ NMR (128 MHz, $CDCl_3$) of **1i**

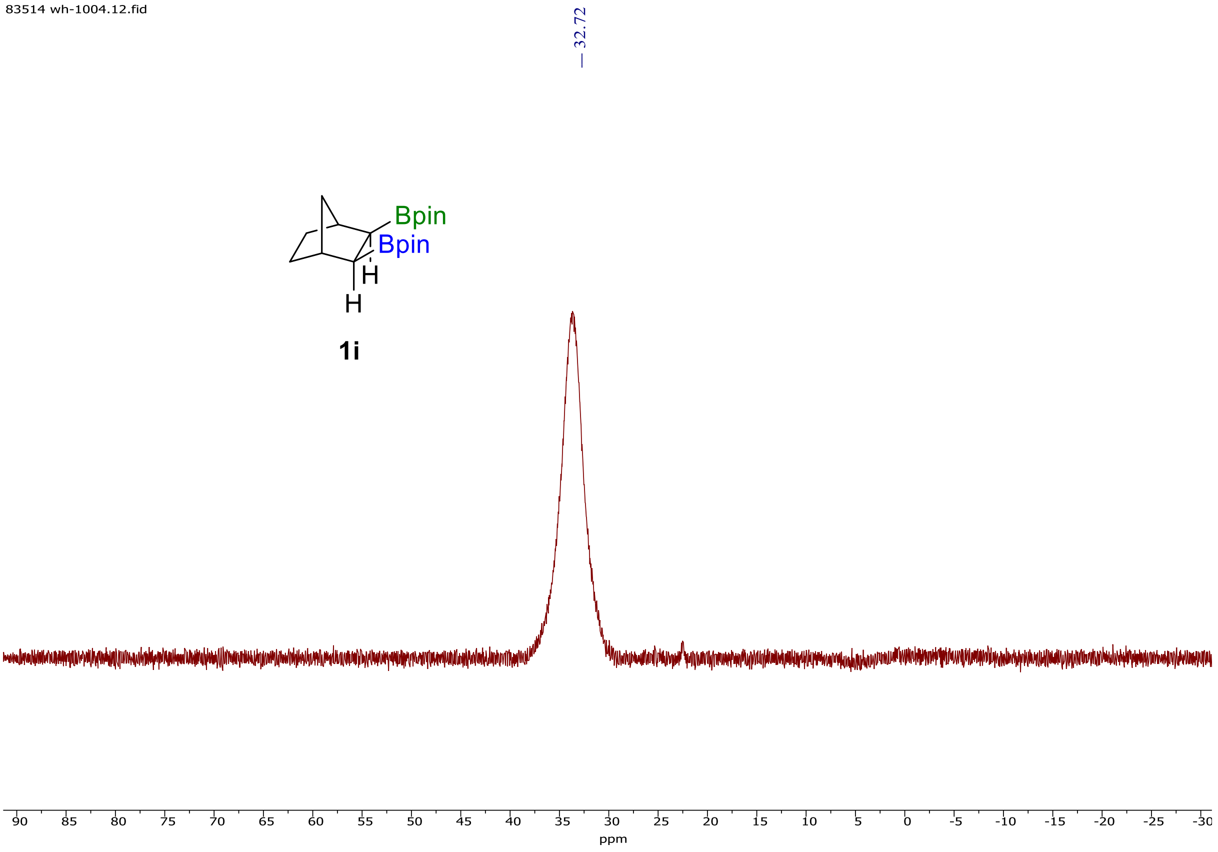

$^{1}$H NMR (400 MHz, $CDCl_3$) of **1l** (*see procedure*)

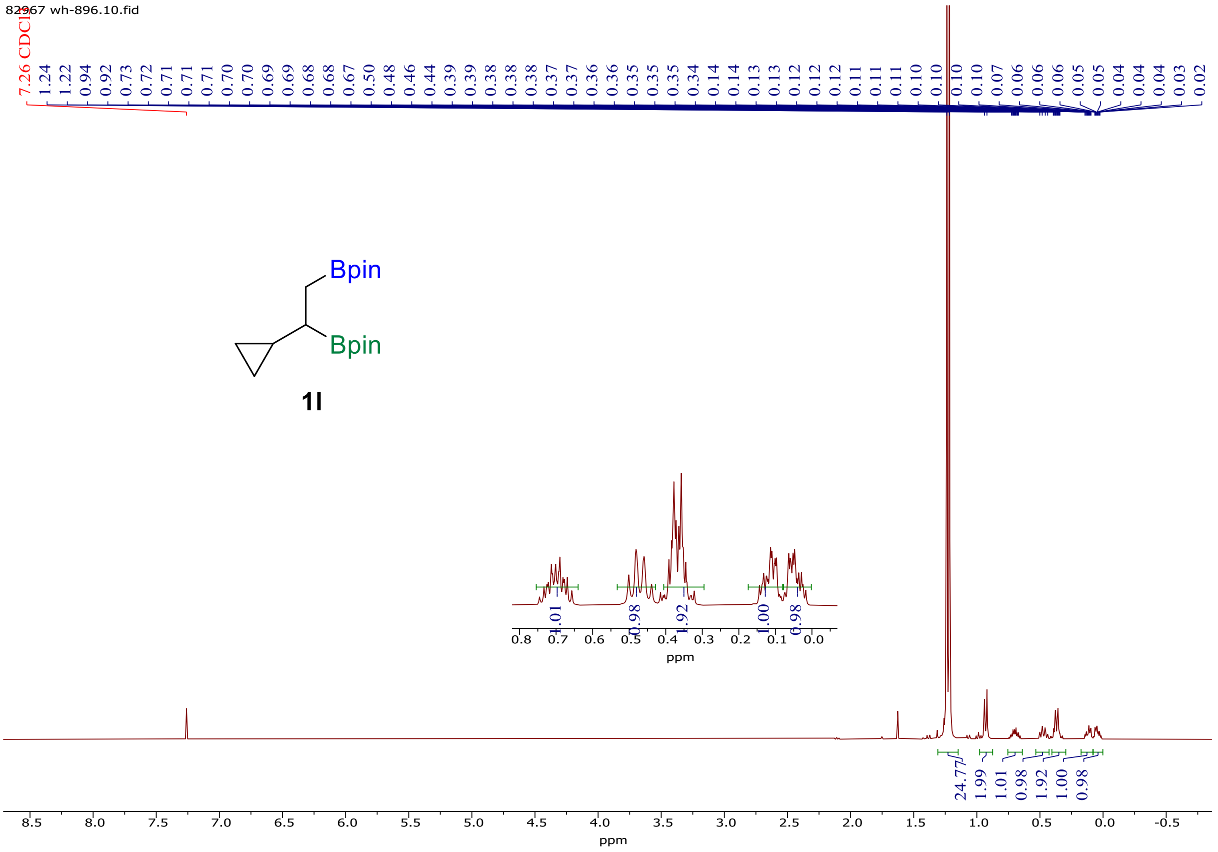

$^{13}C$ NMR (101 MHz, $CDCl_3$) of **1l**

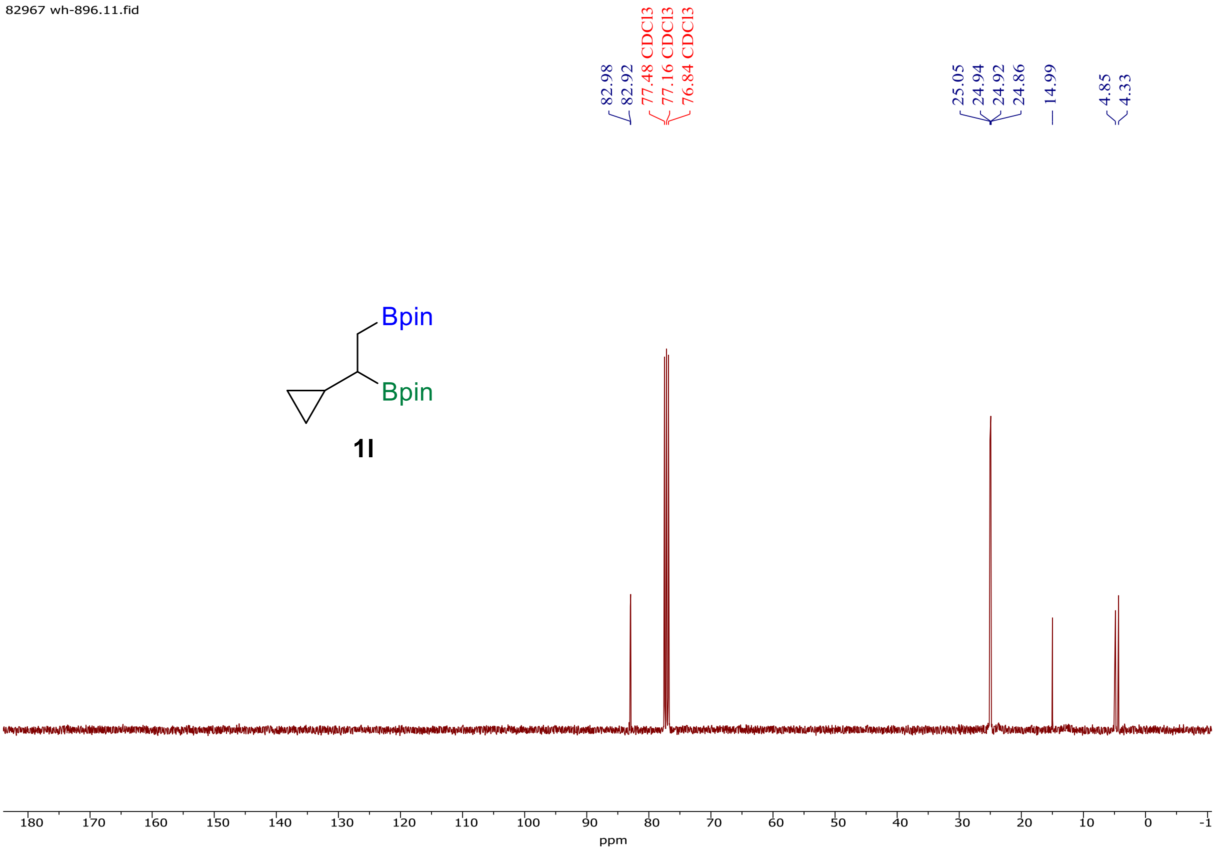

$^{11}$B NMR (128 MHz, $CDCl_3$) of **1l**

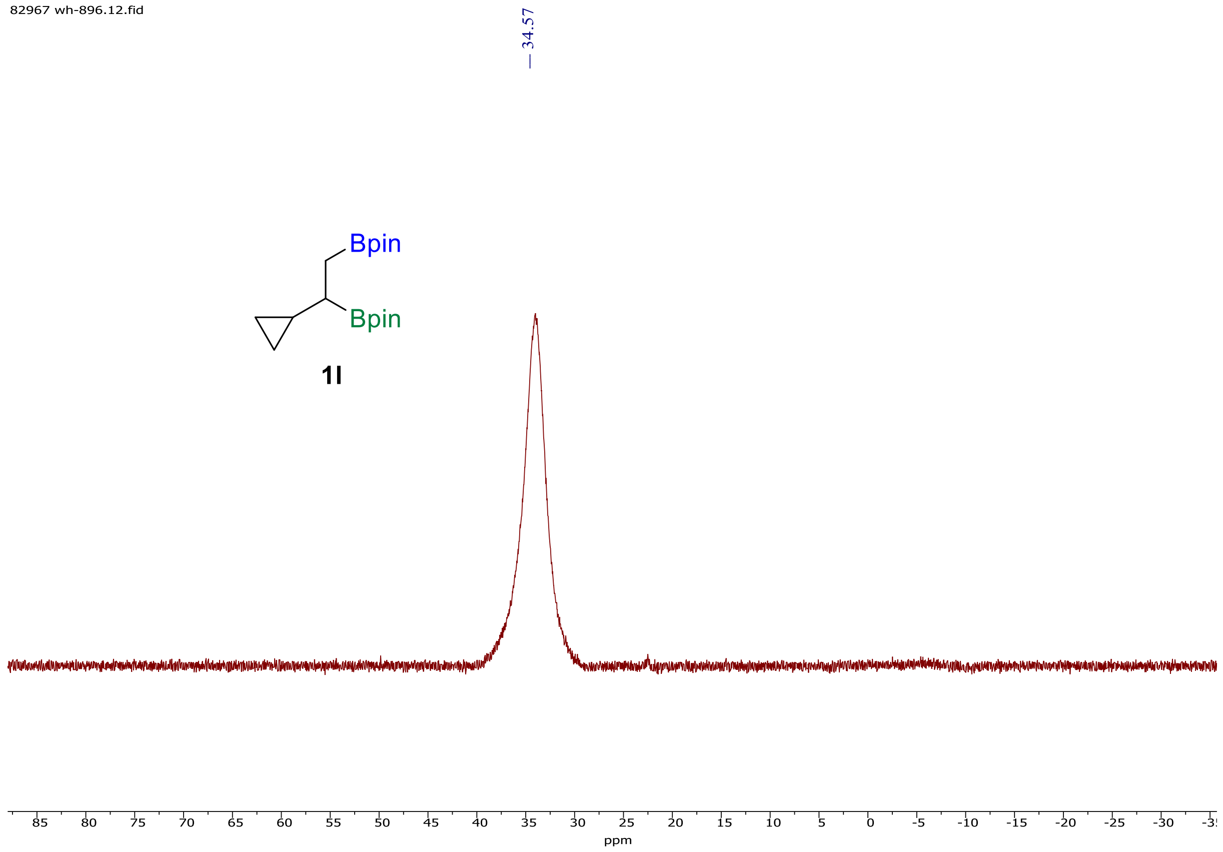

$^1$H NMR (400 MHz, $CDCl_3$) of **6aa** (*see procedure*)

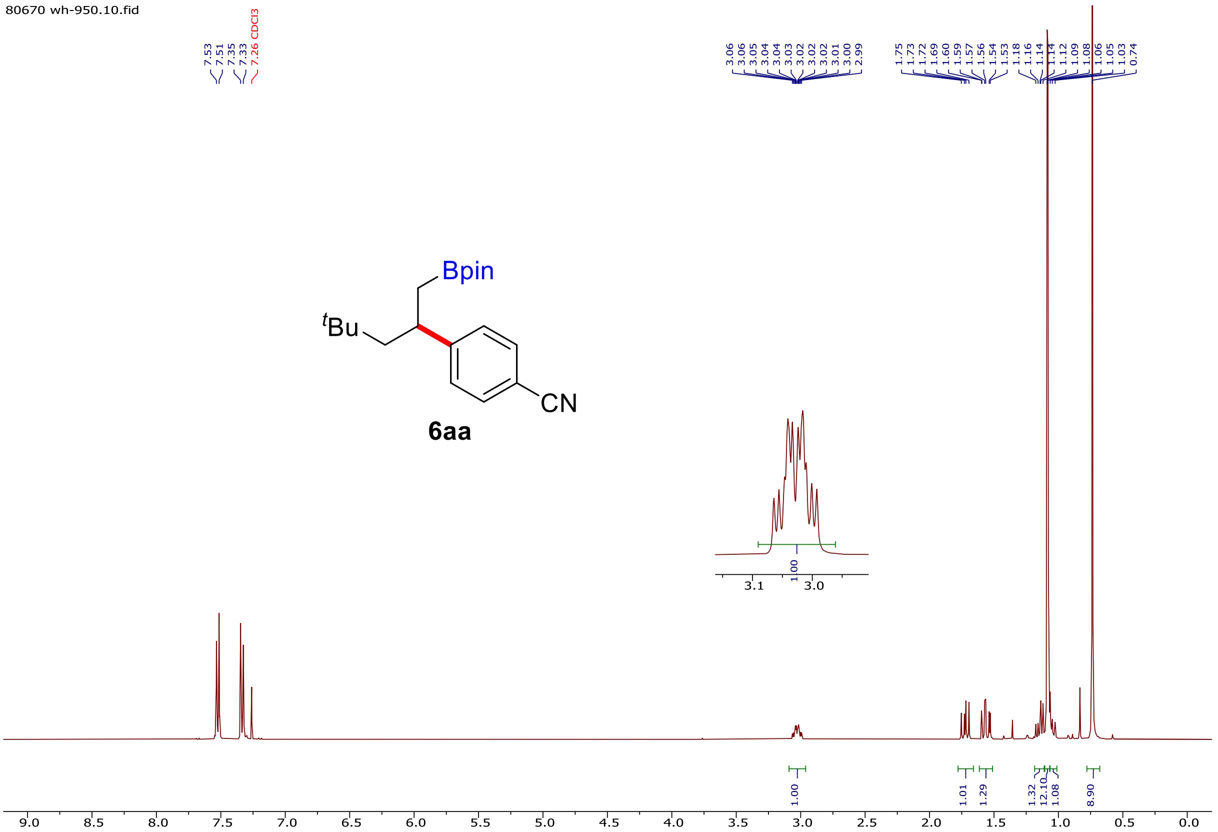

$^{13}$C NMR (101 MHz, $CDCl_3$) of **6aa**

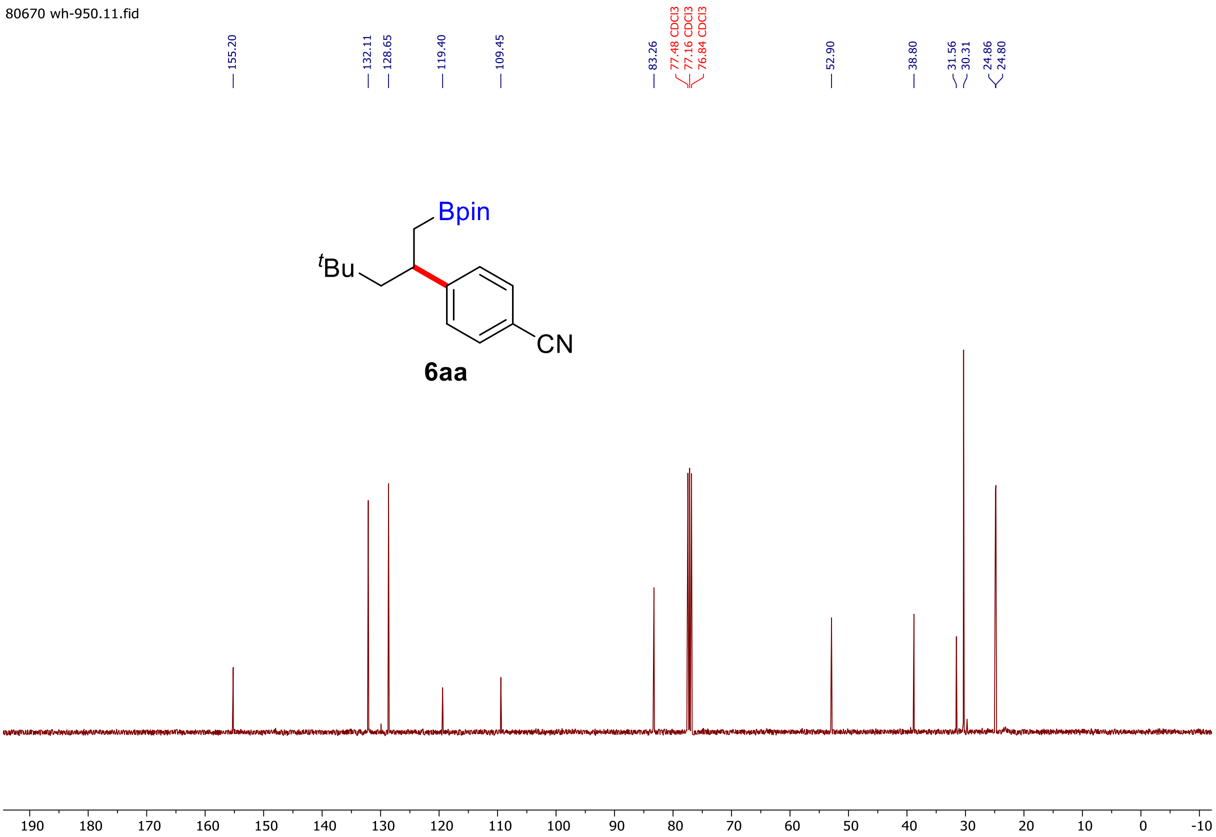

$^{11}$B NMR (128 MHz, $CDCl_3$) of **6aa**

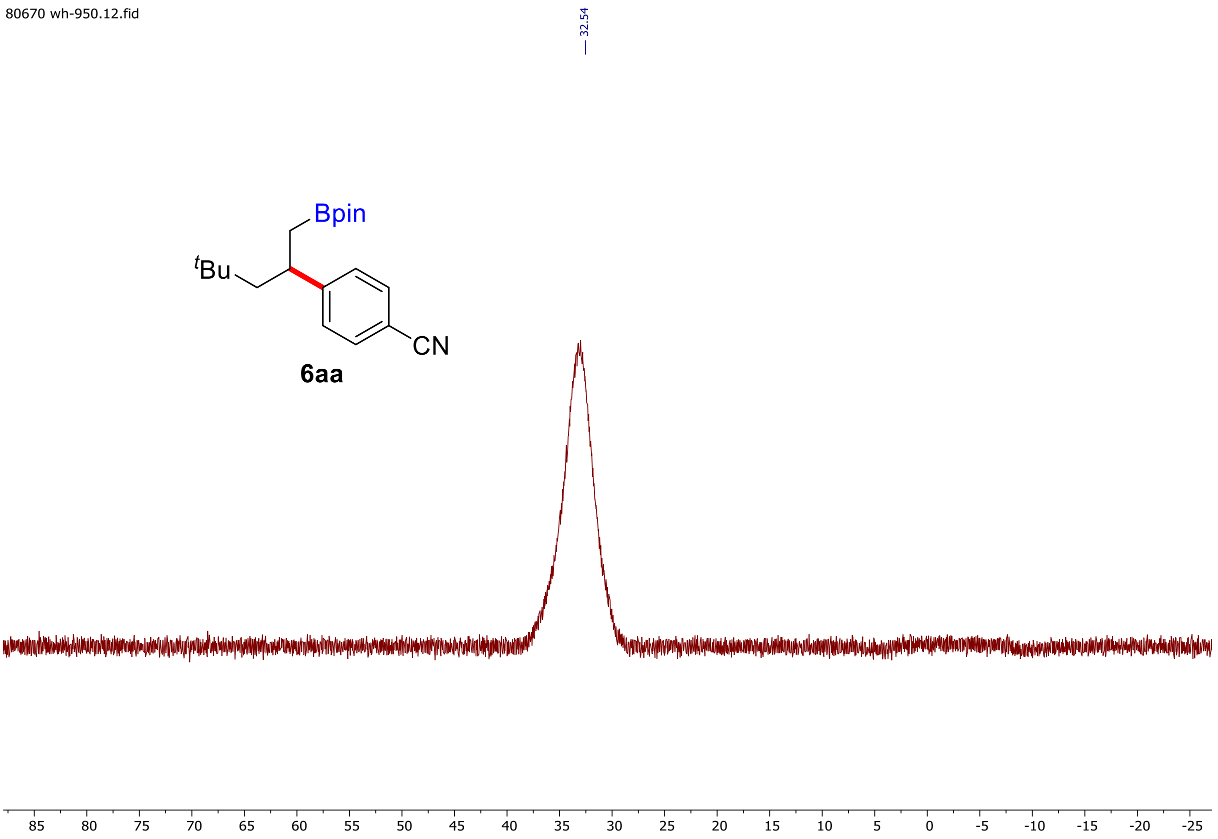

$^1$H NMR (400 MHz, $CDCl_3$) of **6ab** (*see procedure*)

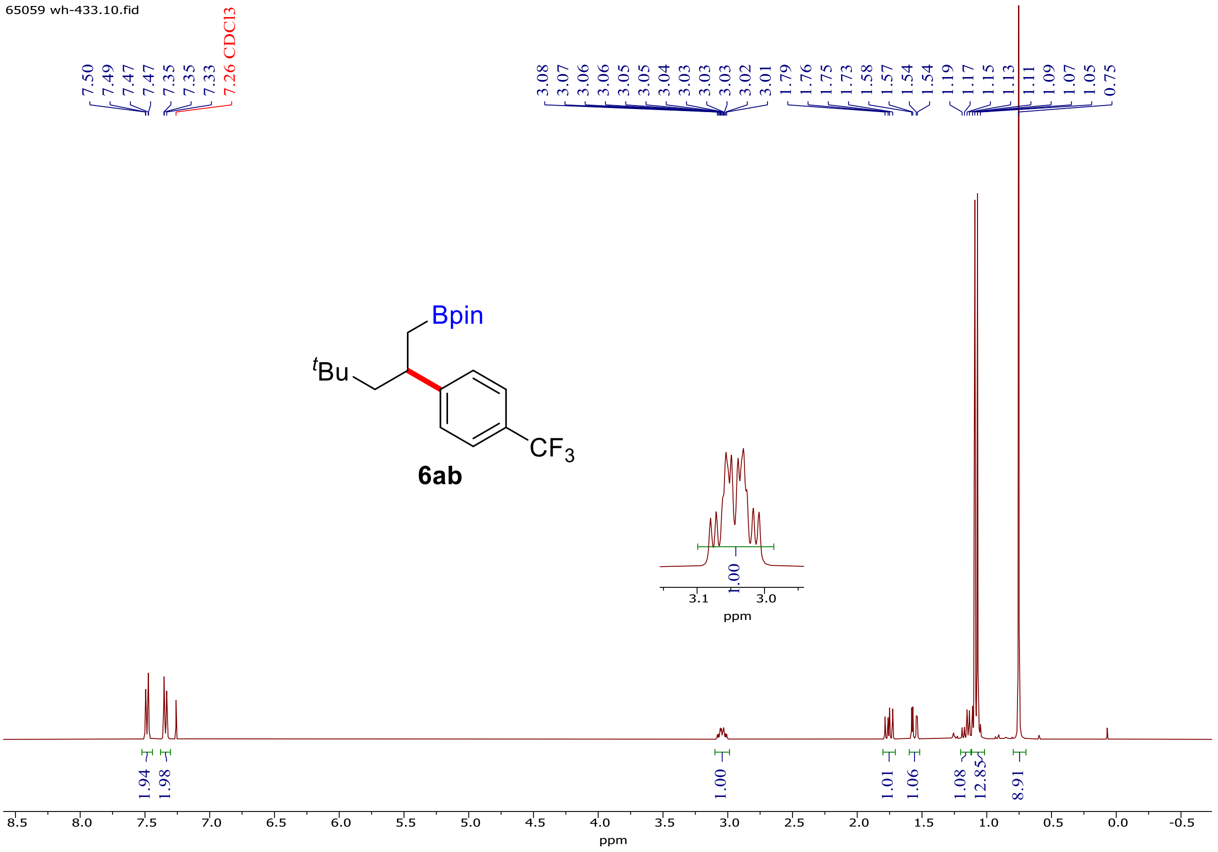

$^{13}$C NMR (101 MHz, $CDCl_3$) of **6ab**

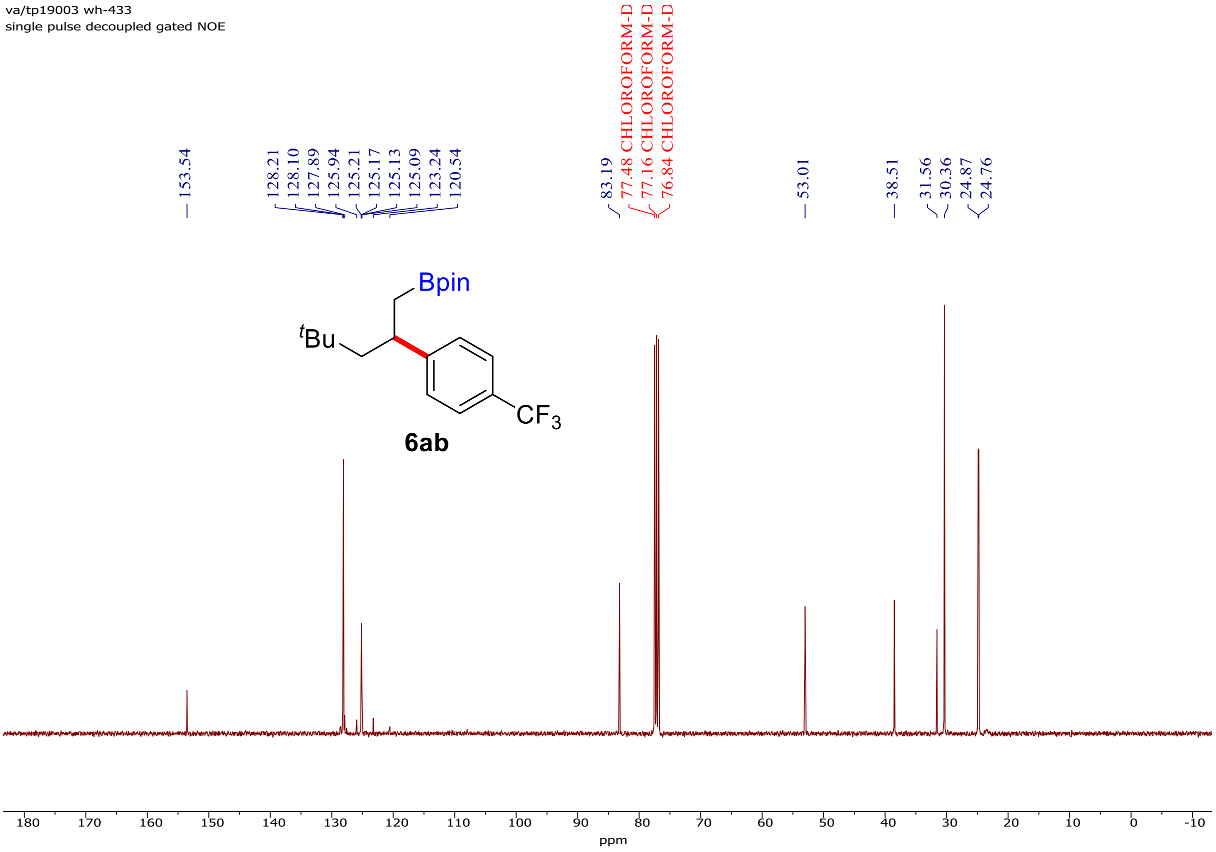

$^{19}$F NMR (377 MHz, $CDCl_3$) of **6ab**

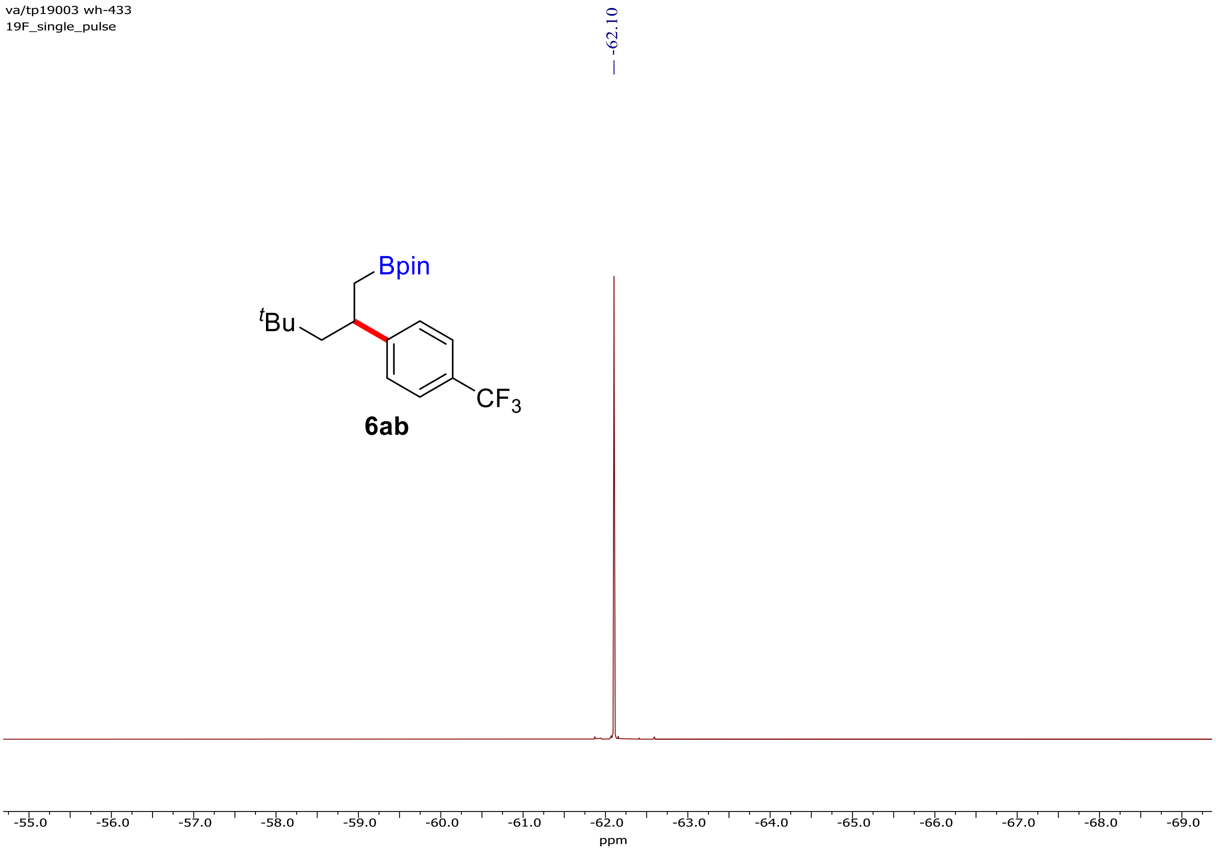

$^{11}$B NMR (128 MHz, $CDCl_3$) of **6ab**

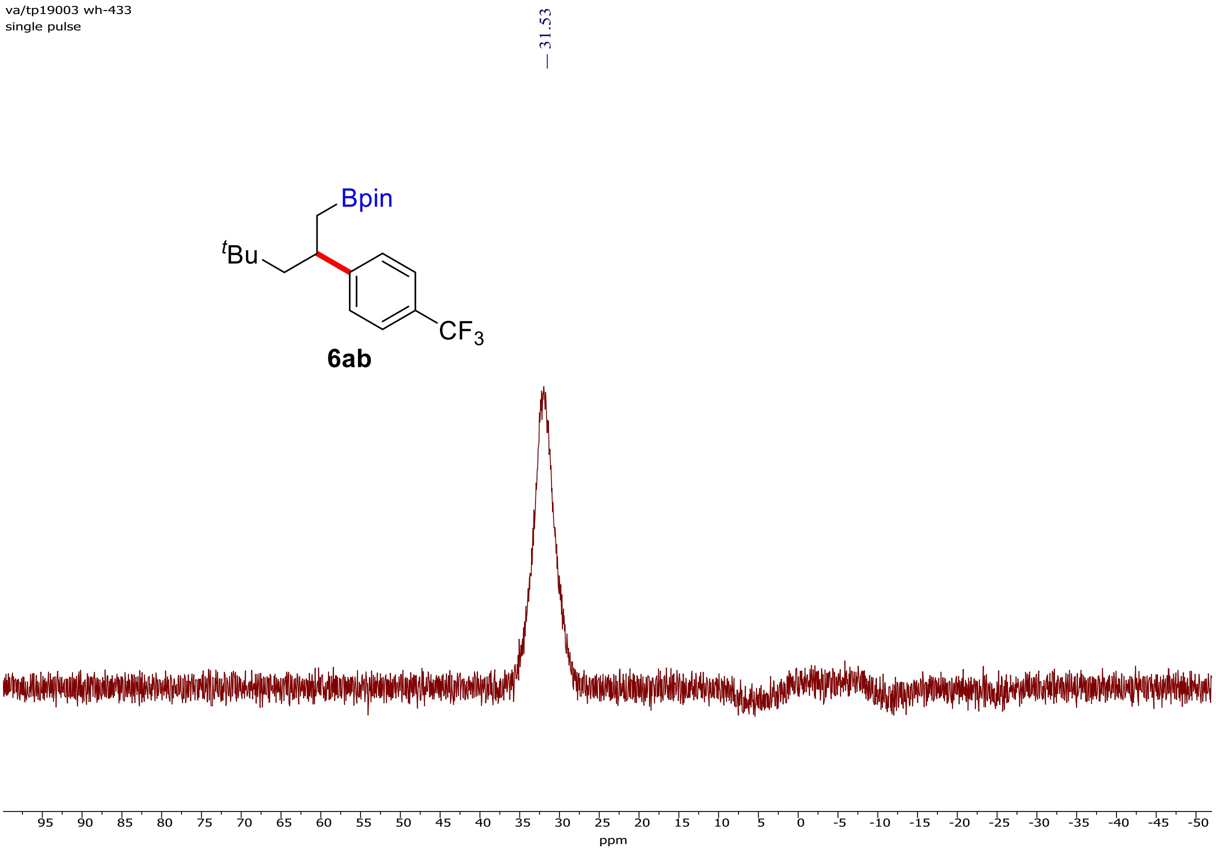

$^1$H NMR (400 MHz, $CDCl_3$) of **6ac** (*see procedure*)

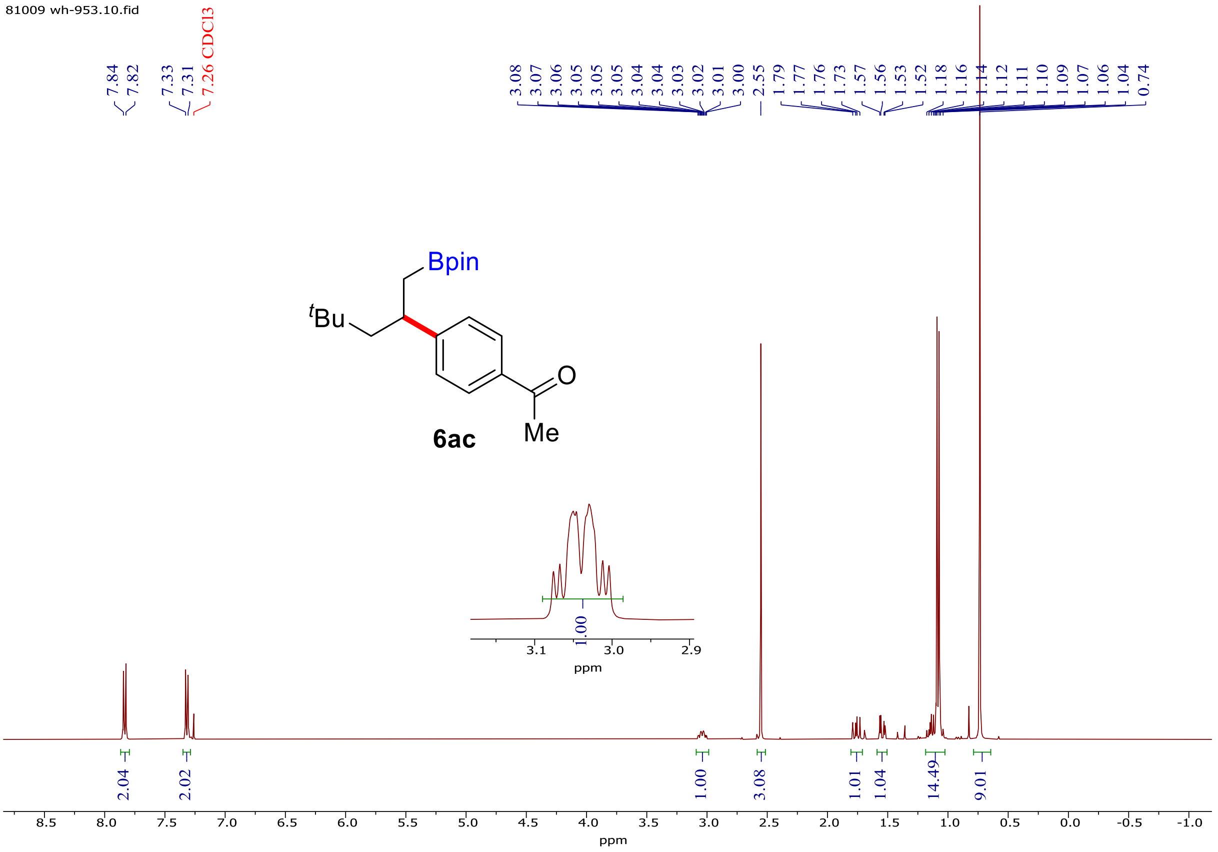

$^{13}C$ NMR (101 MHz, $CDCl_3$) of **6ac**

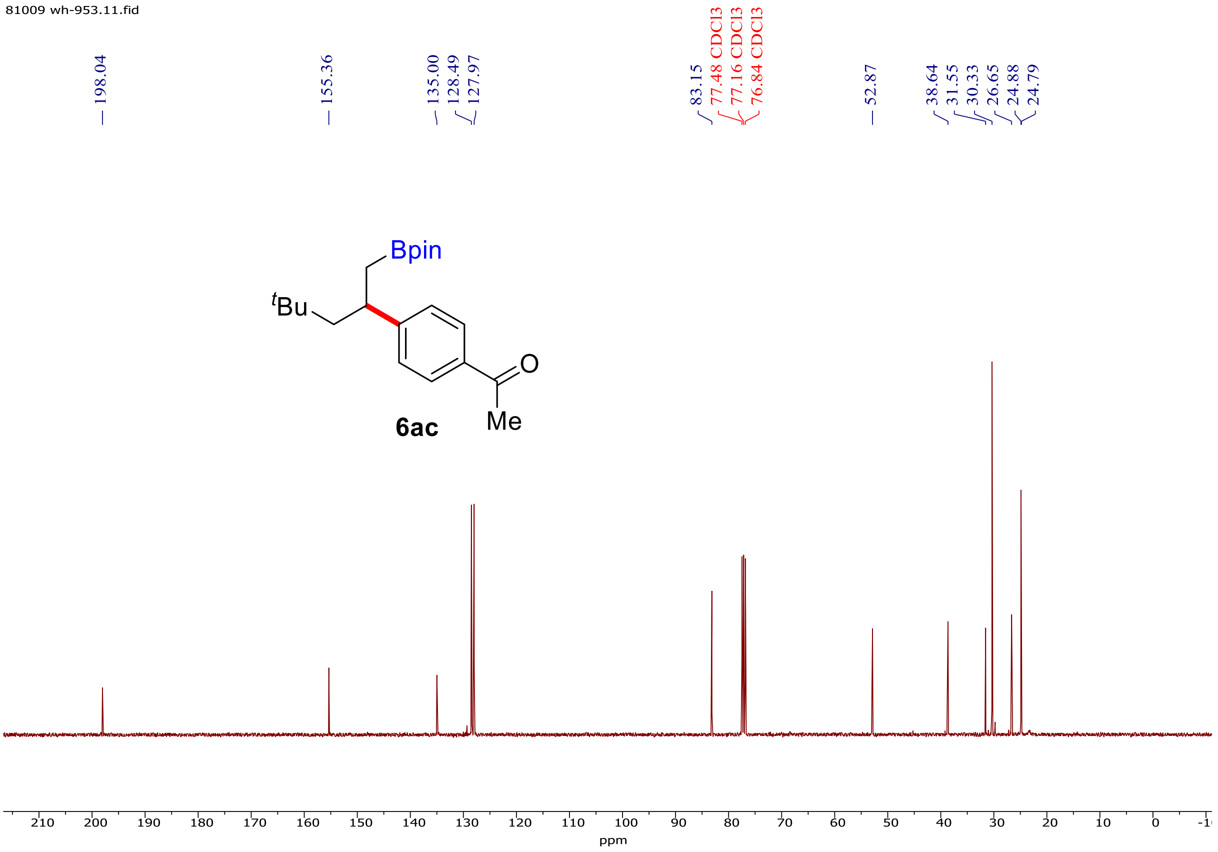

$^{11}B$ NMR (128 MHz, $CDCl_3$) of **6ac**

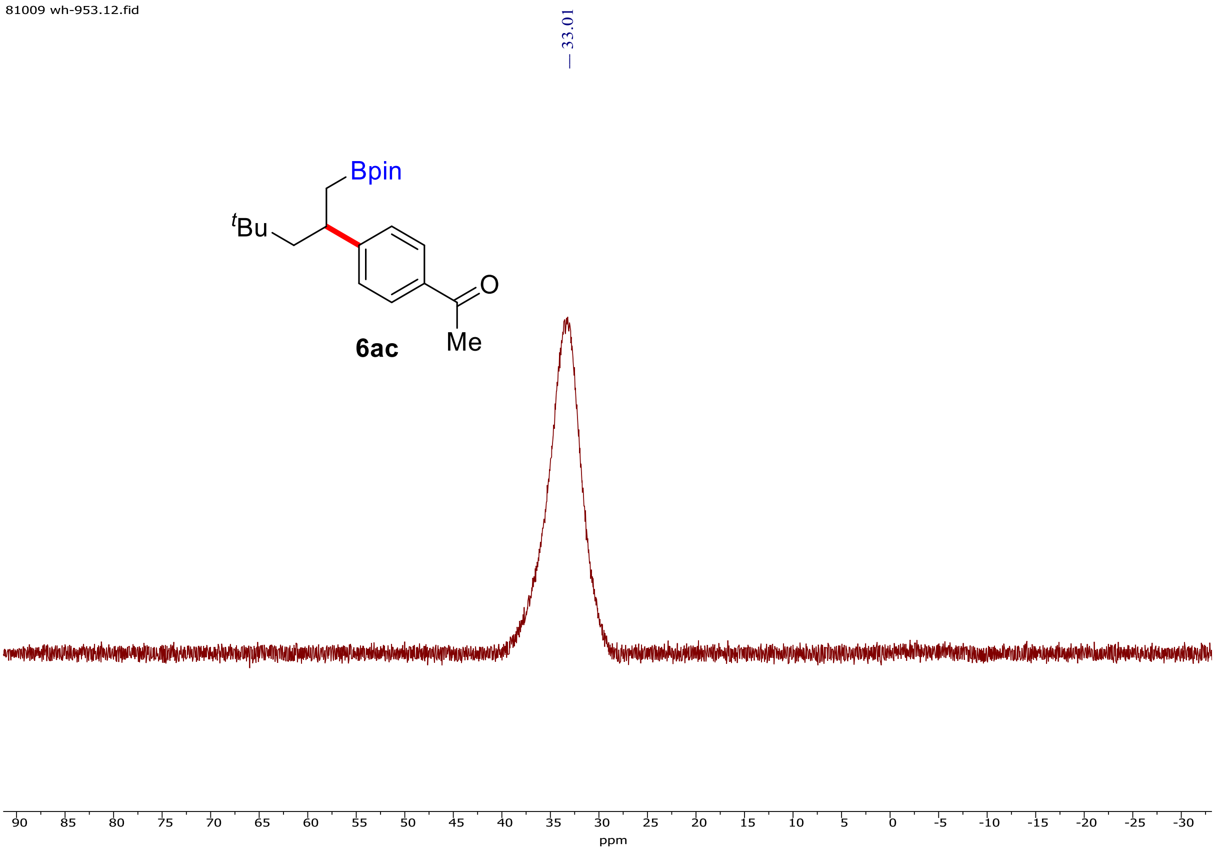

$^{1}H$ NMR (400 MHz, $CDCl_3$) of **6ad** (*see procedure*)

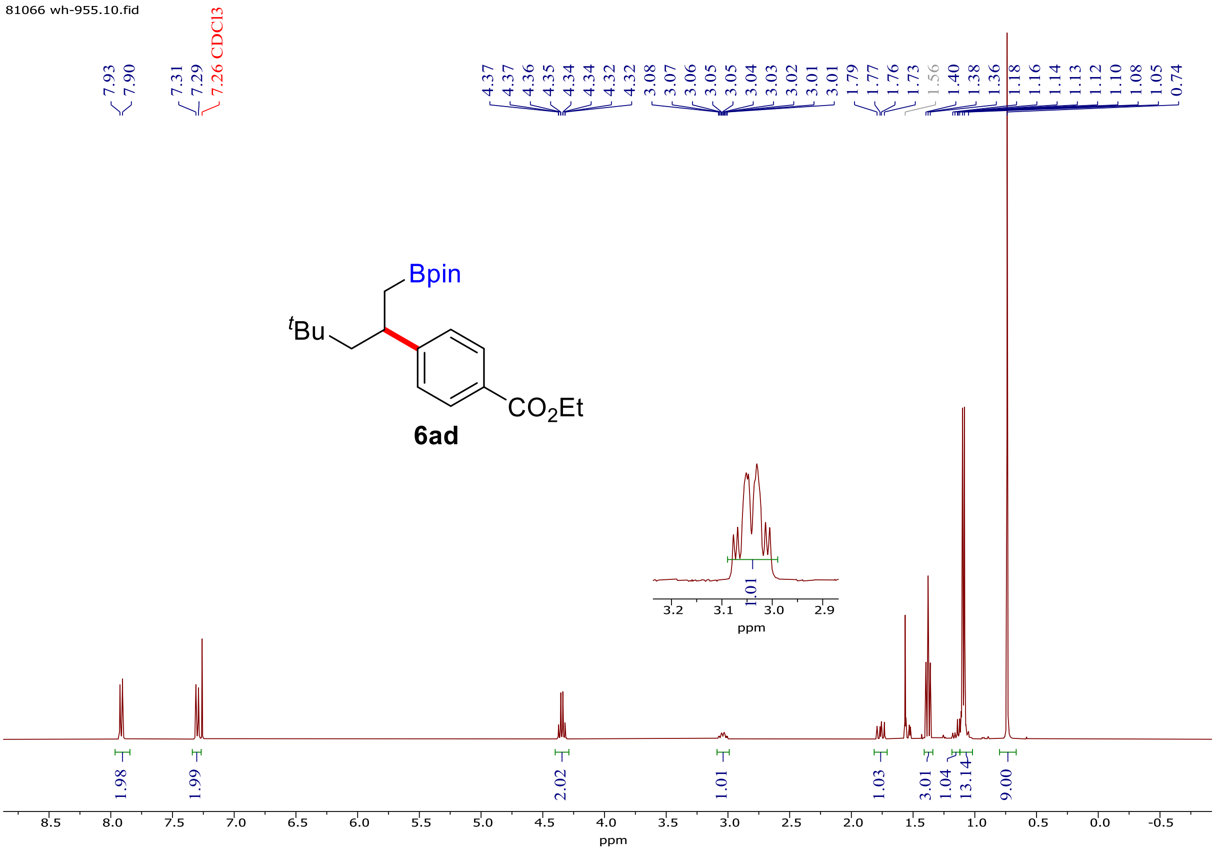

$^{13}$C NMR (101 MHz, $CDCl_3$) of **6ad**

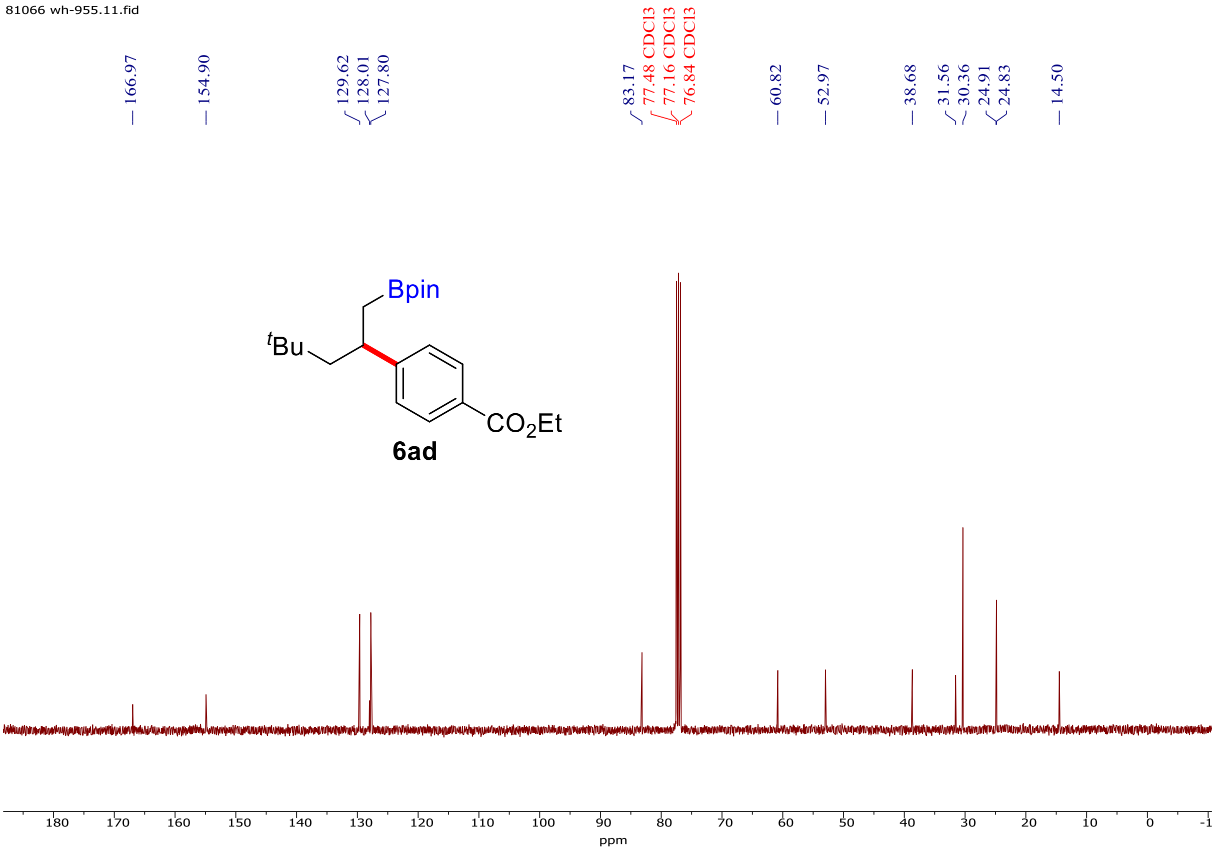

$^{11}$B NMR (128 MHz, $CDCl_3$) of **6ad**

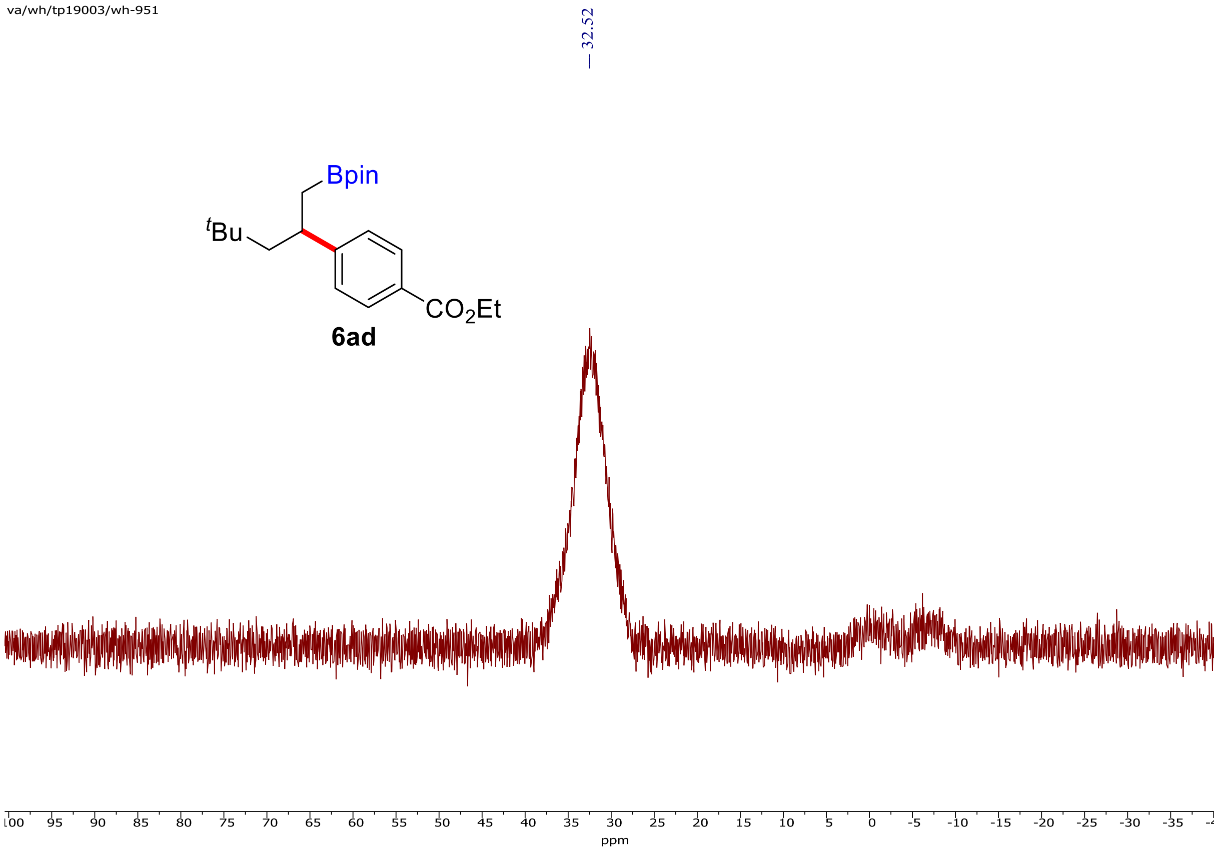

$^{1}H$ NMR (400 MHz, $CDCl_3$) of **6ae** (*see procedure*)

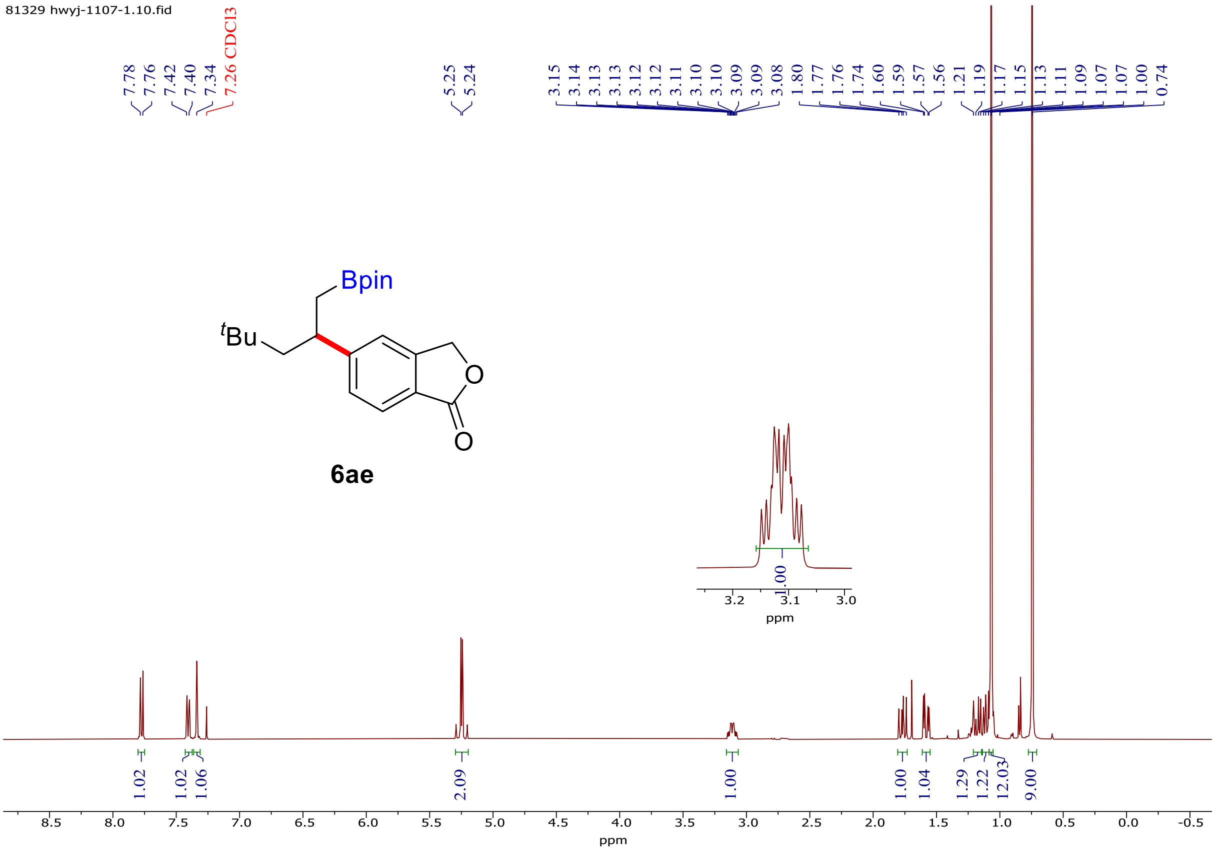


$^{13}C$ NMR (101 MHz, $CDCl_3$) of **6ae**

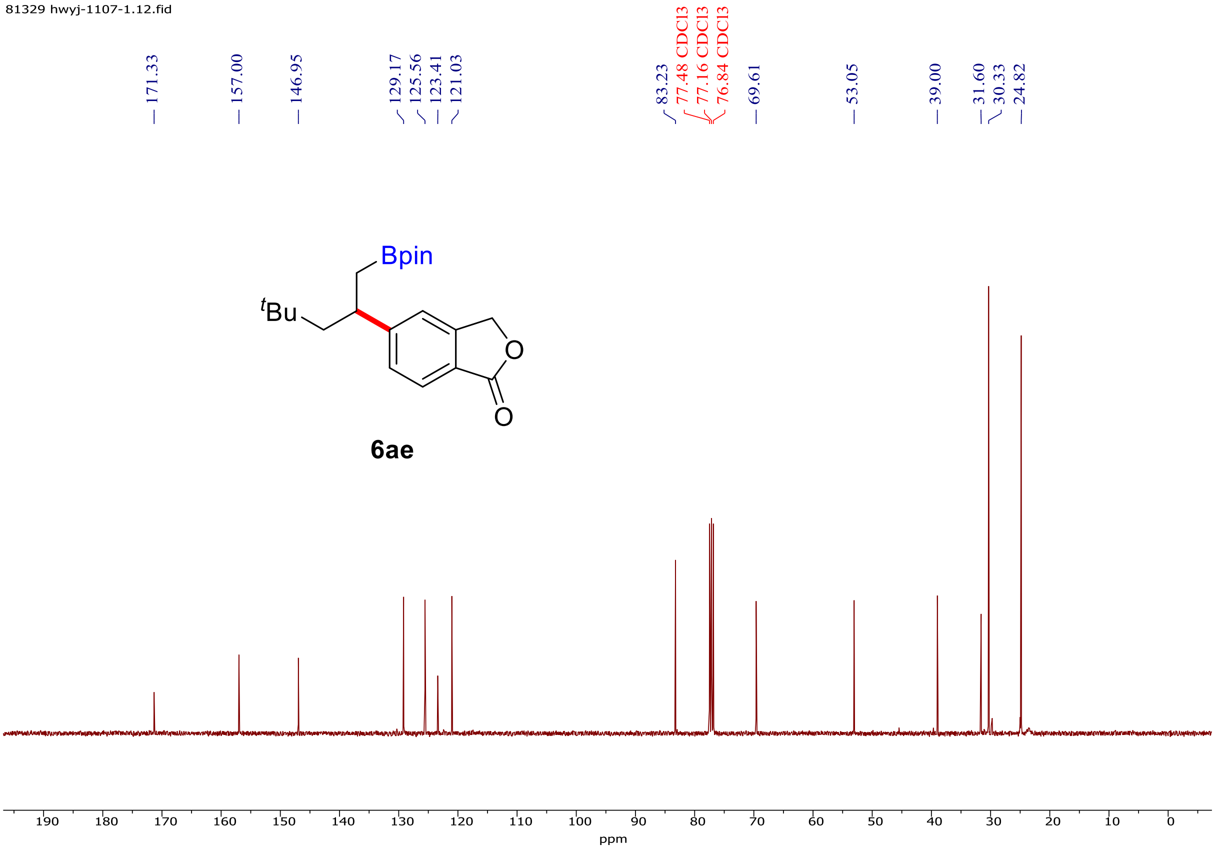

$^{11}$B NMR (128 MHz, $CDCl_3$) of **6ae**

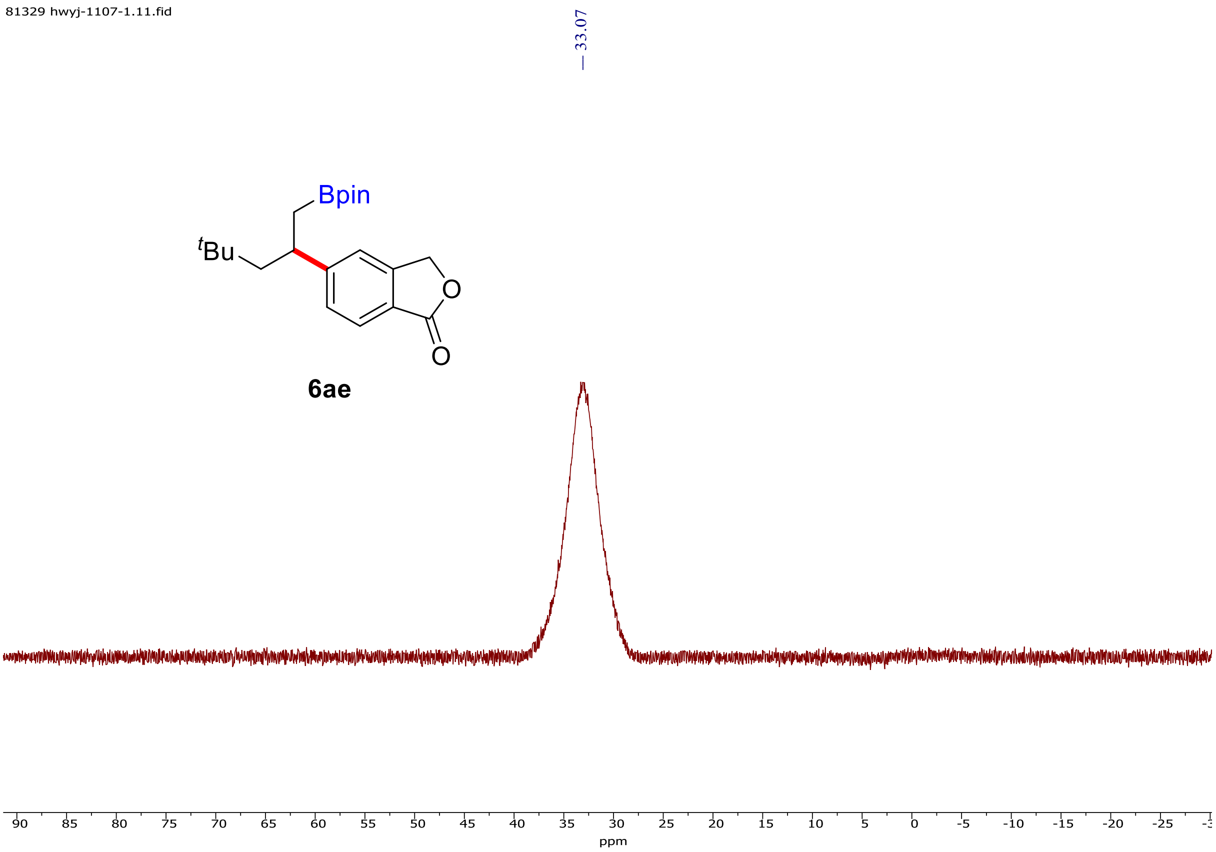


$^{1}$H NMR (400 MHz, $CDCl_3$) of **6af** (*see procedure*)

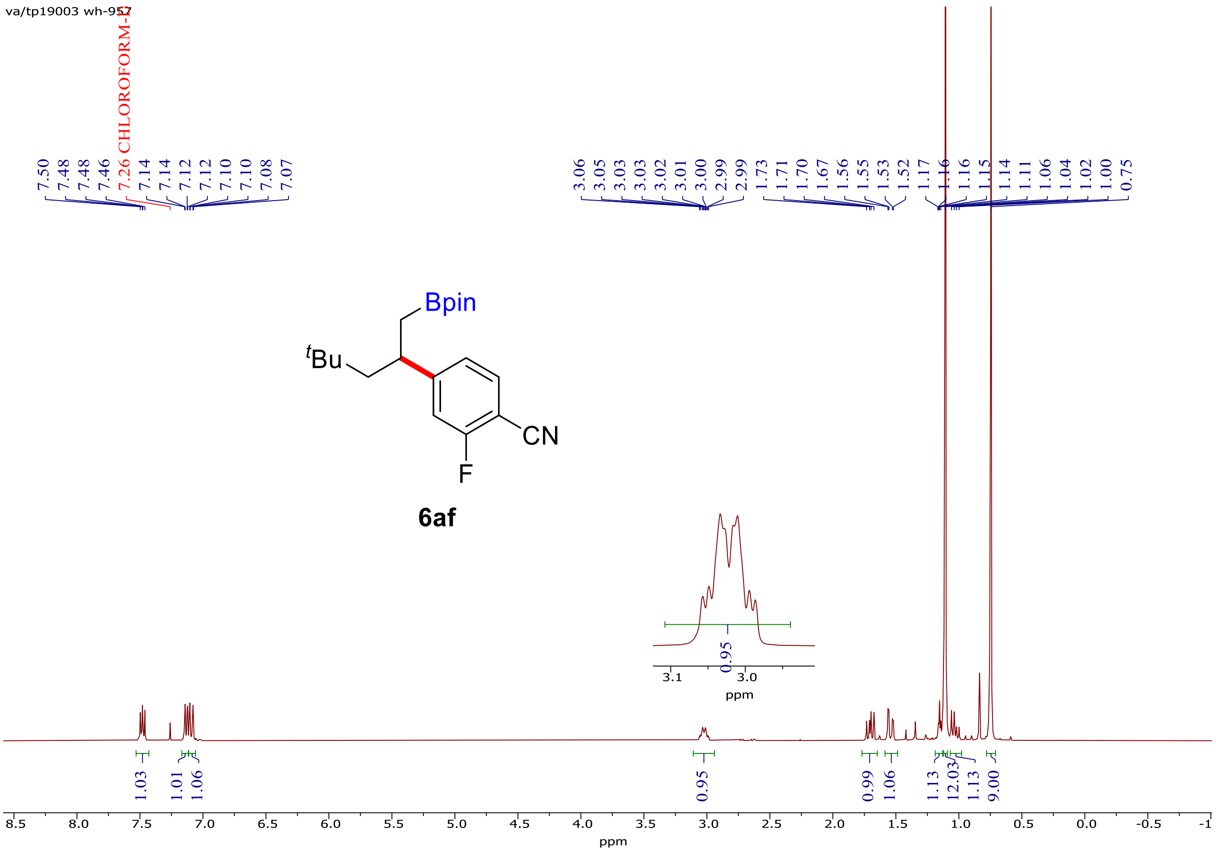

$^{13}$C NMR (101 MHz, $CDCl_3$) of **6af**

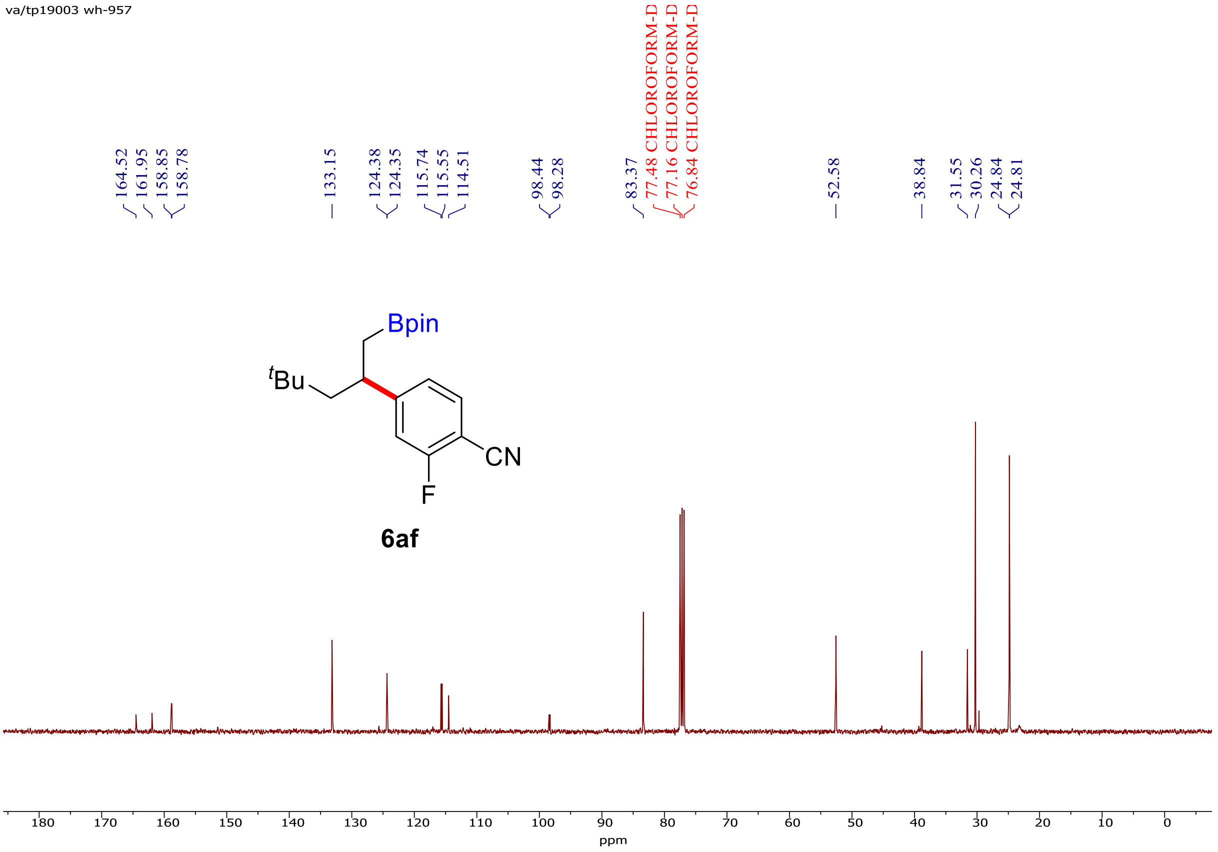

$^{19}F$ NMR (376 MHz, $CDCl_3$) of **6af**

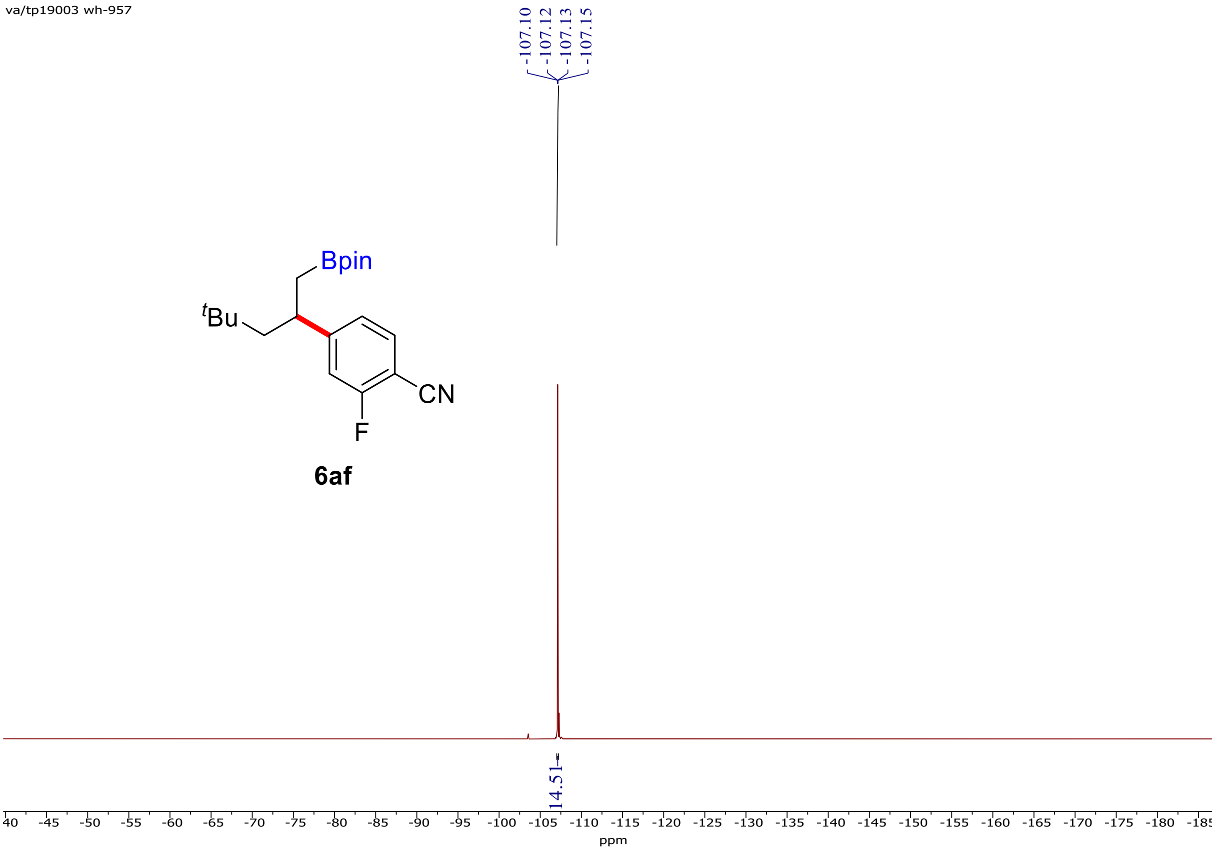

$^{11}B$ NMR (128 MHz, $CDCl_3$) of **6af**

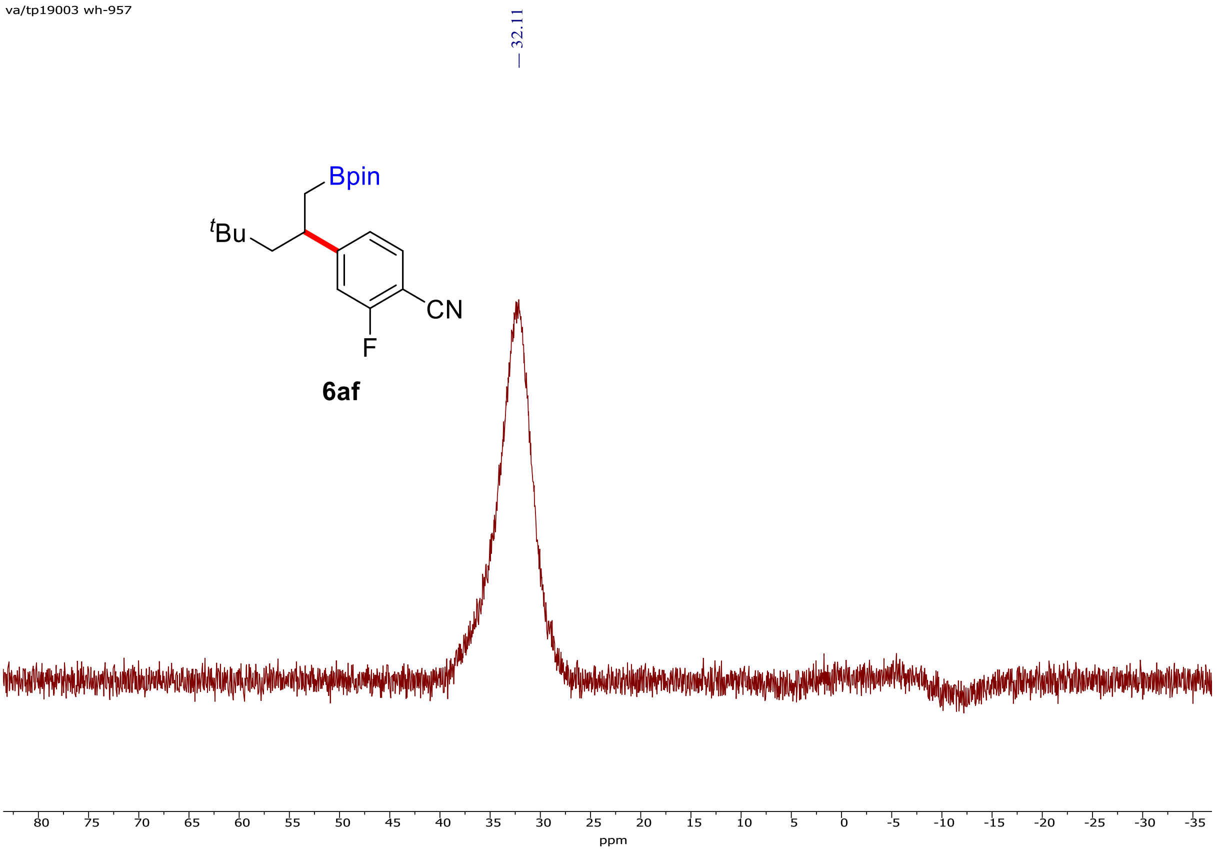

$^{1}$H NMR (400 MHz, $CDCl_3$) of **6ag** (*see procedure*)

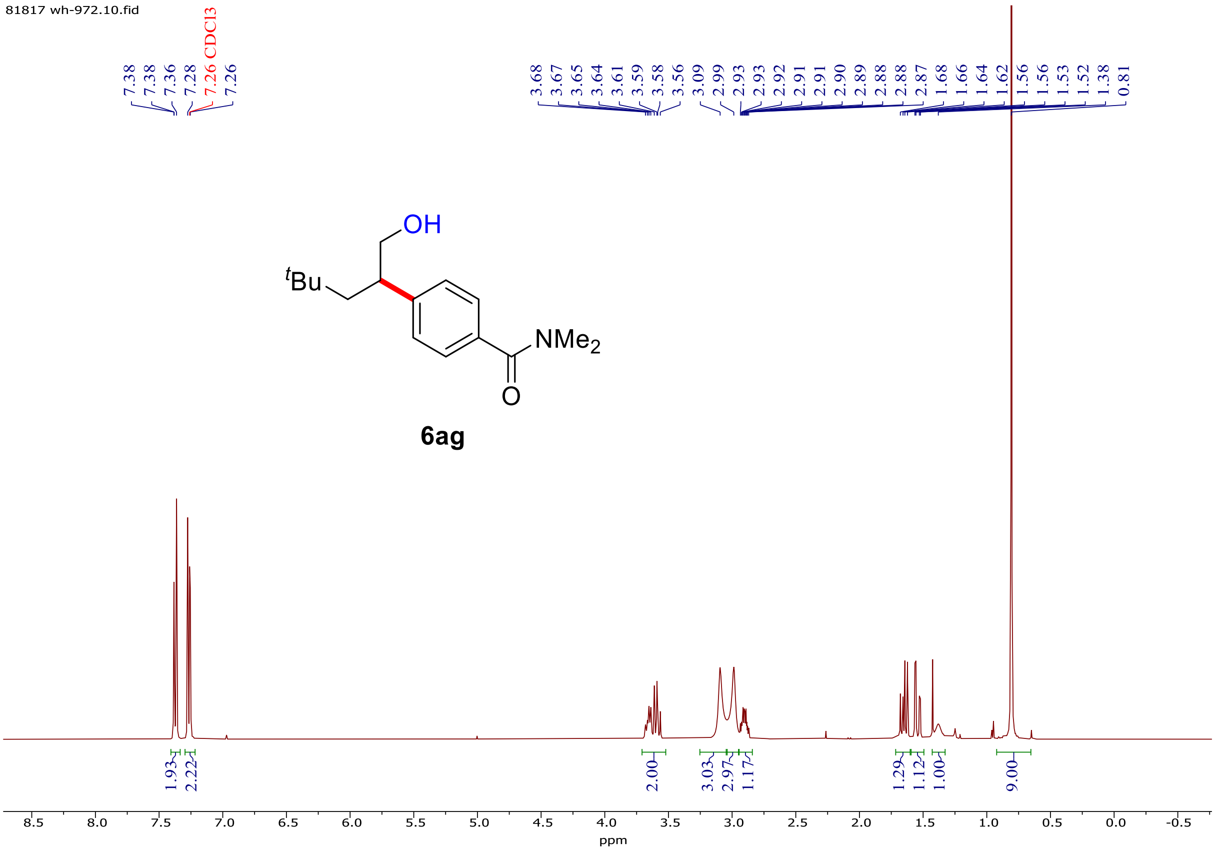

$^{13}$C NMR (101 MHz, $CDCl_3$) of **6ag**

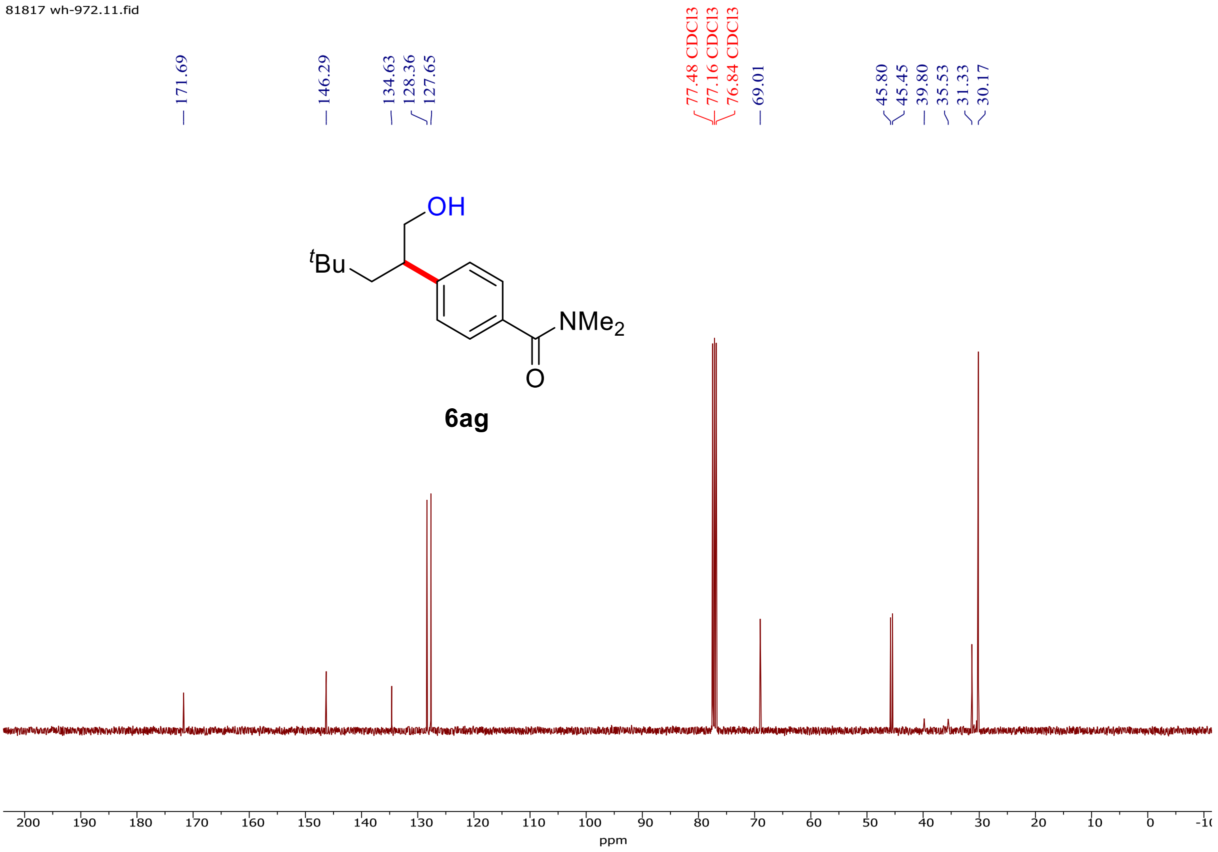

$^1$H NMR (400 MHz, $CDCl_3$) of **6ah** (*see procedure*)

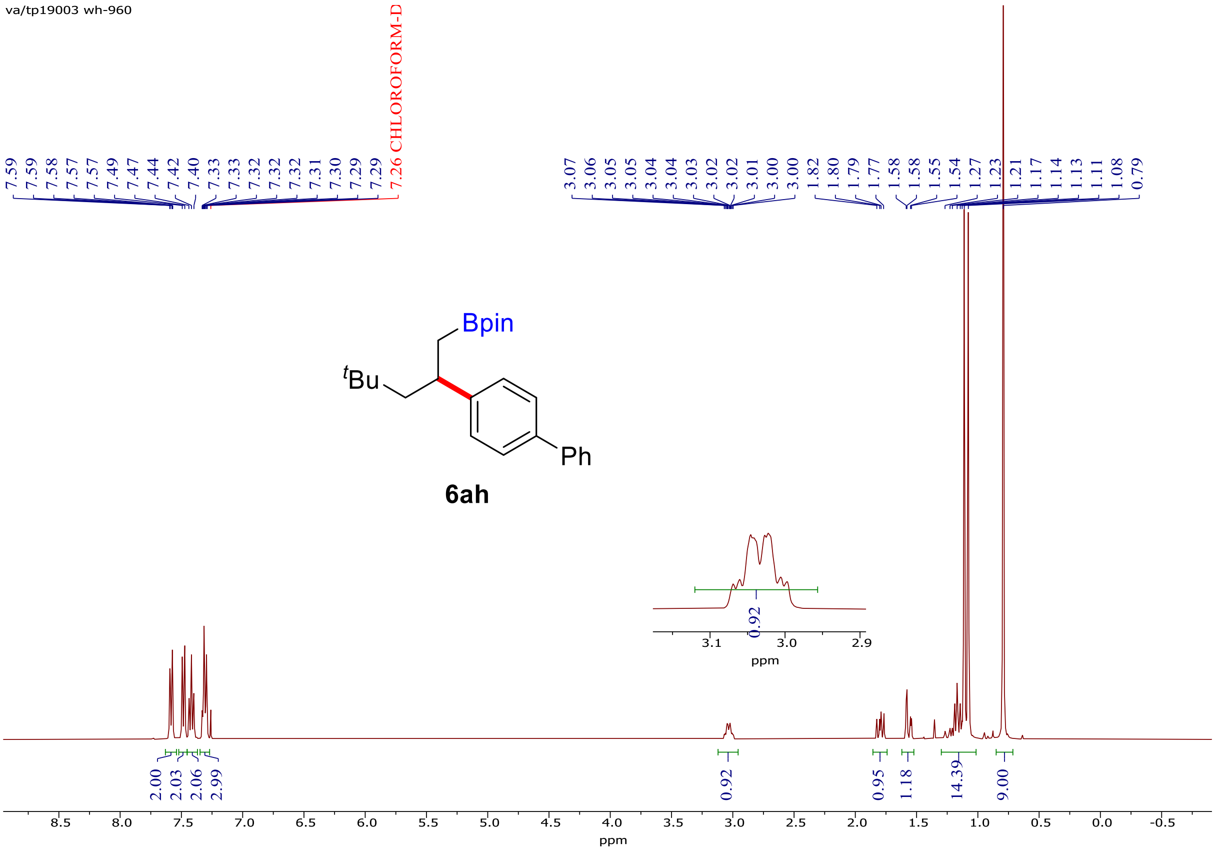

$^{13}C$ NMR (101 MHz, $CDCl_3$) of **6ah**

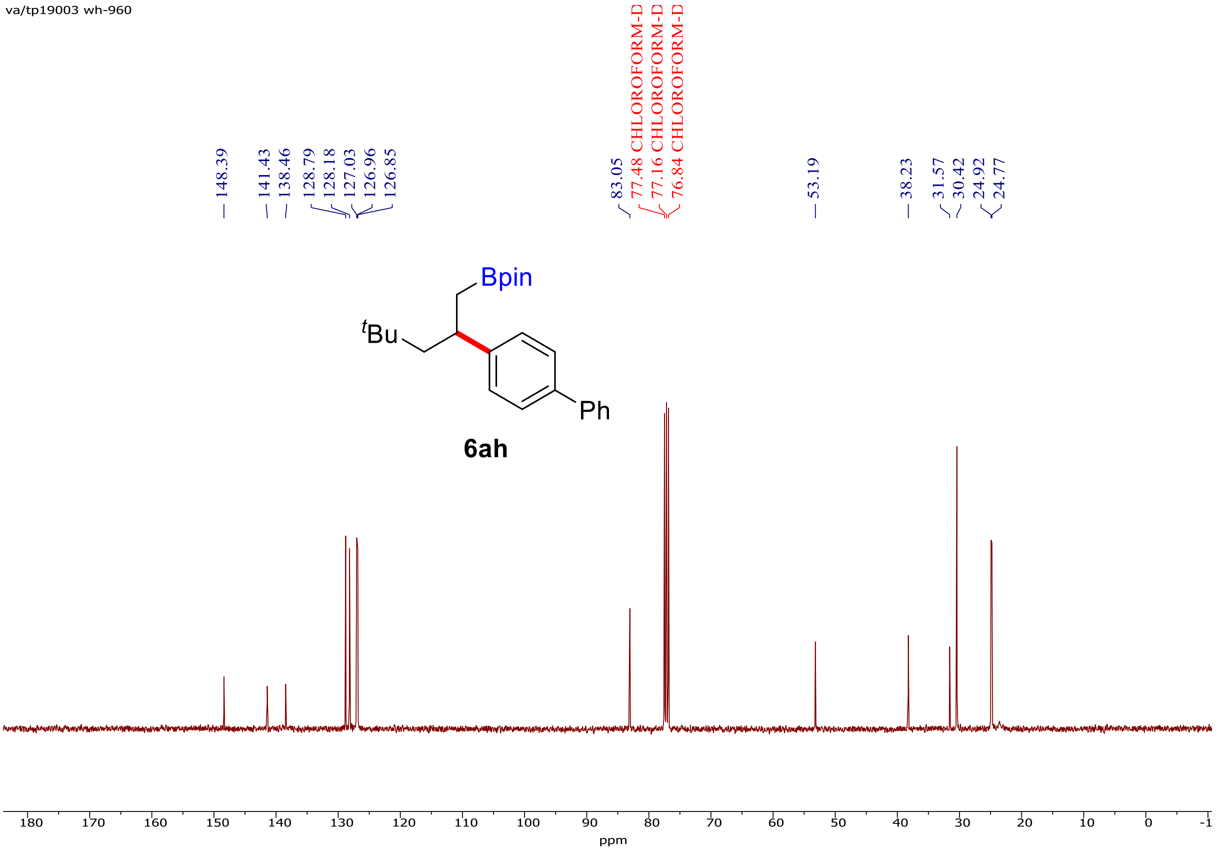

$^{11}$B NMR (128 MHz, $CDCl_3$) of **6ah**

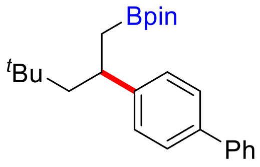


**6ah**

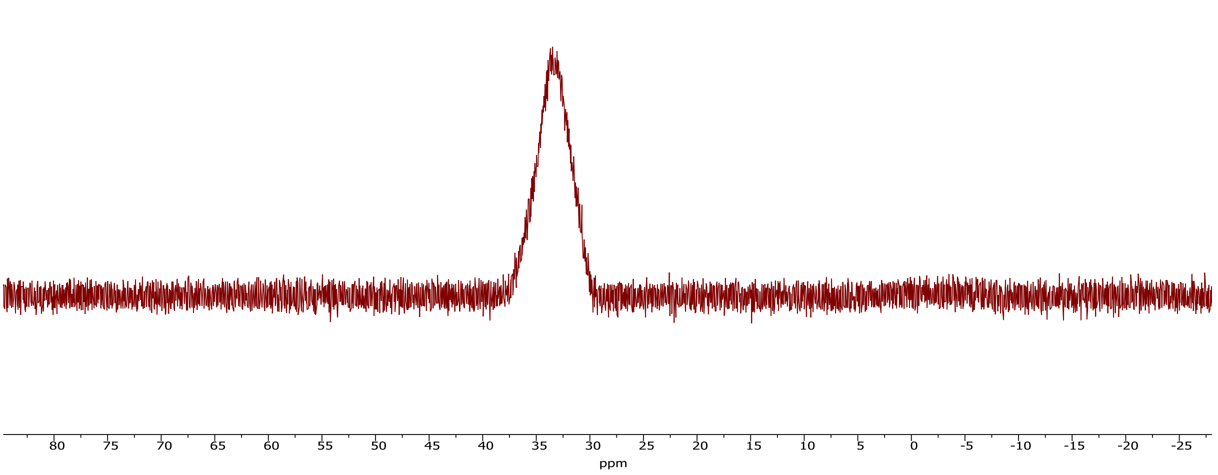

$^1$H NMR (400 MHz, $CDCl_3$) of **6ai** (*see procedure*)

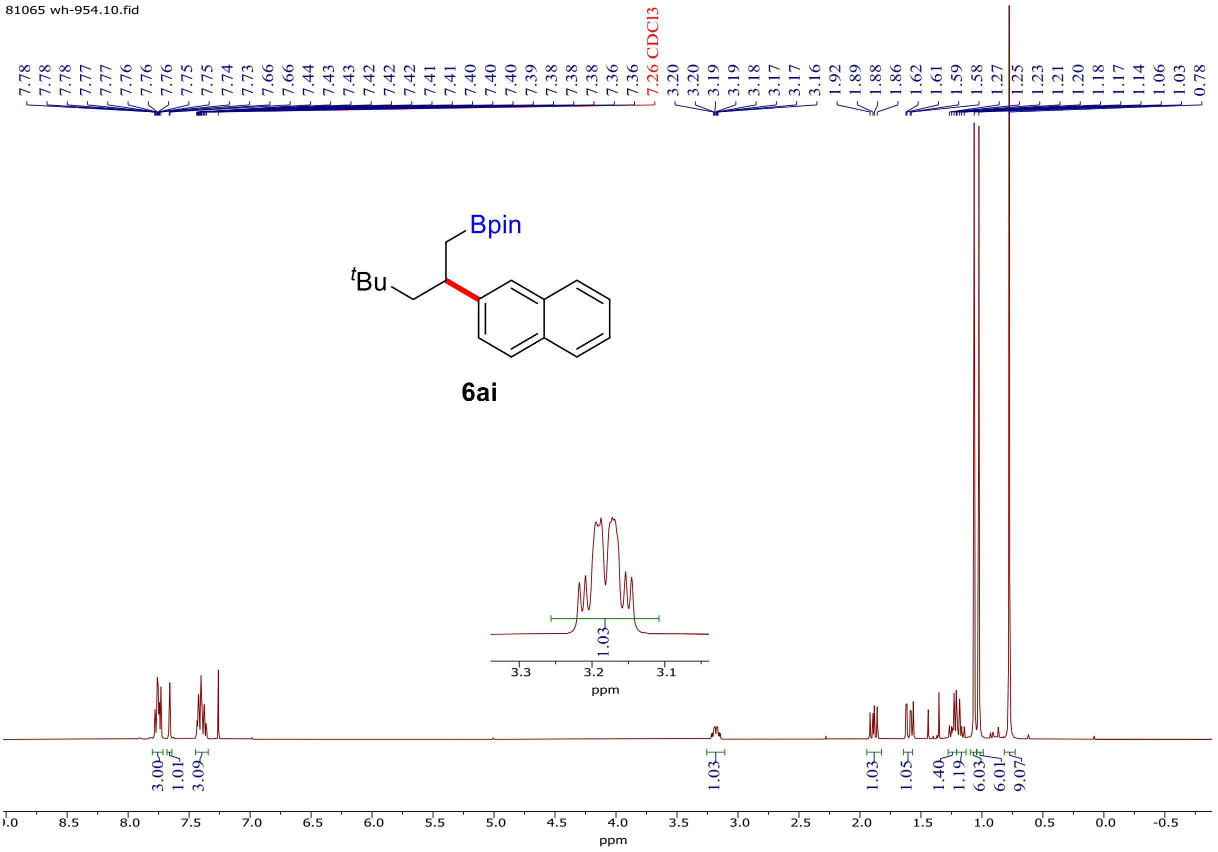

$^{13}$C NMR (101 MHz, $CDCl_3$) of **6ai**

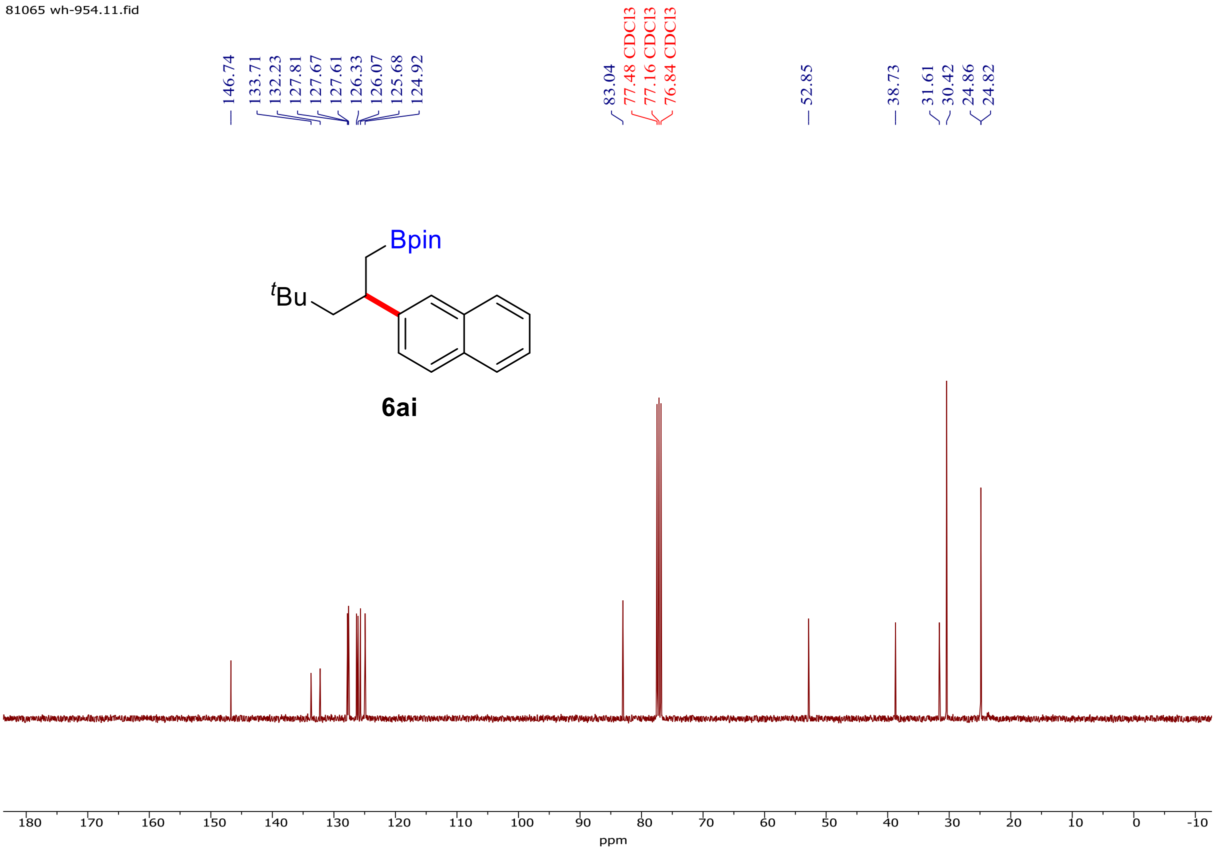

$^{11}$B NMR (128 MHz, $CDCl_3$) of **6ai**

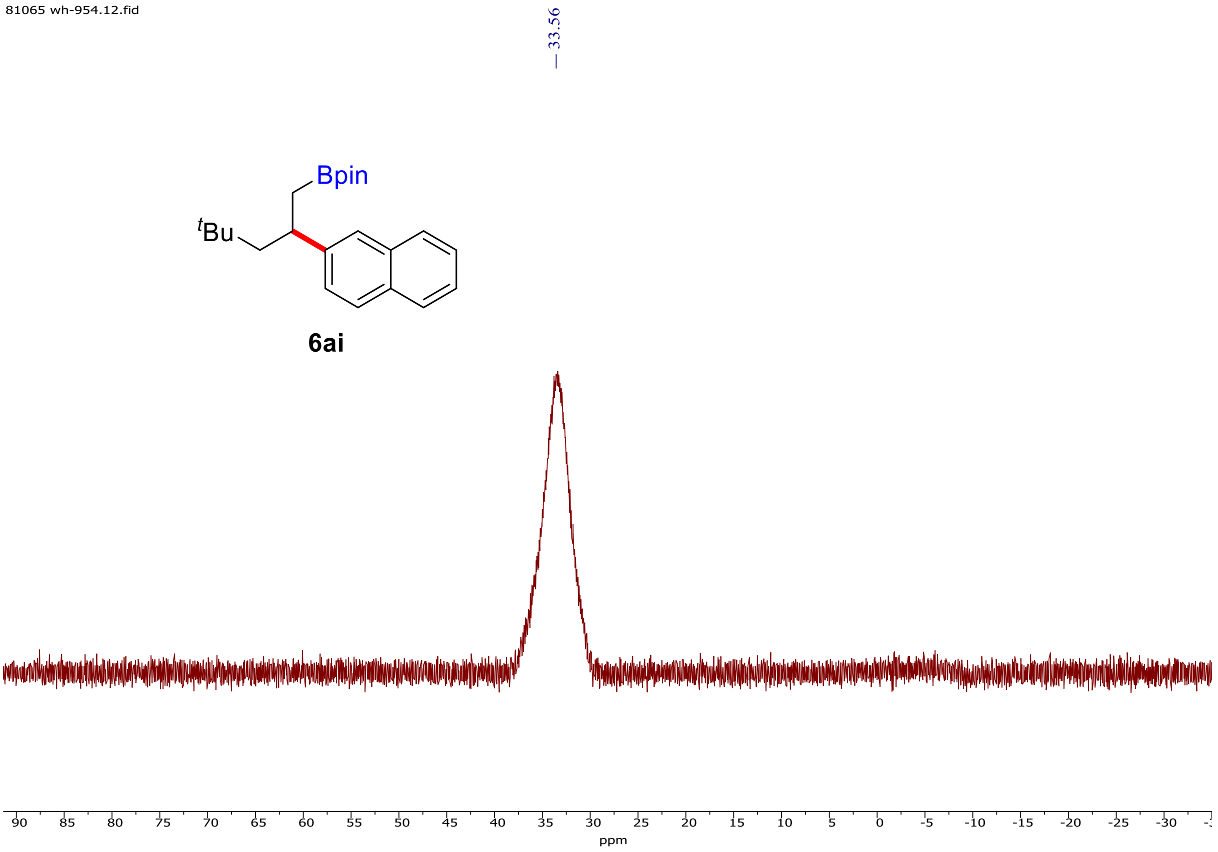


$^{1}$H NMR (400 MHz, $CDCl_3$) of **6aj** (*see procedure*)

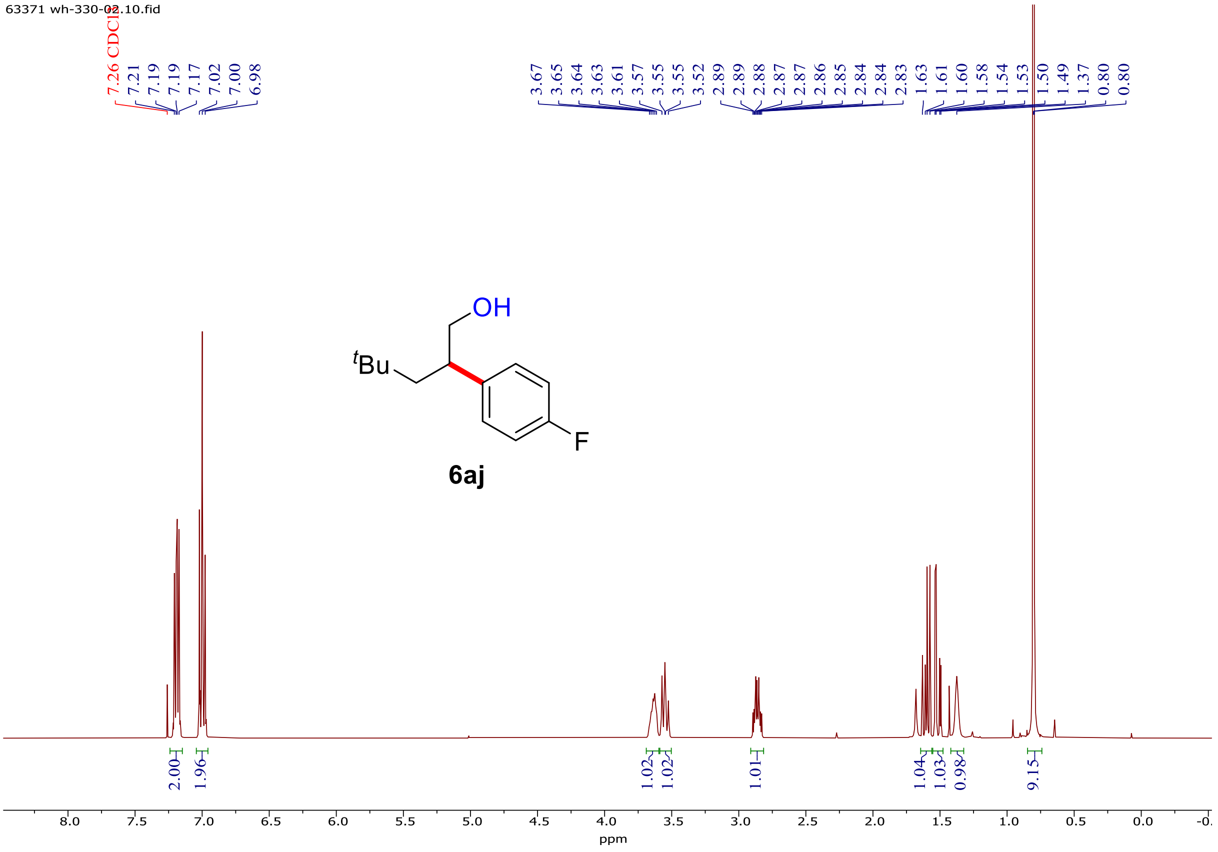

$^{13}C$ NMR (101 MHz, $CDCl_3$) of **6aj**

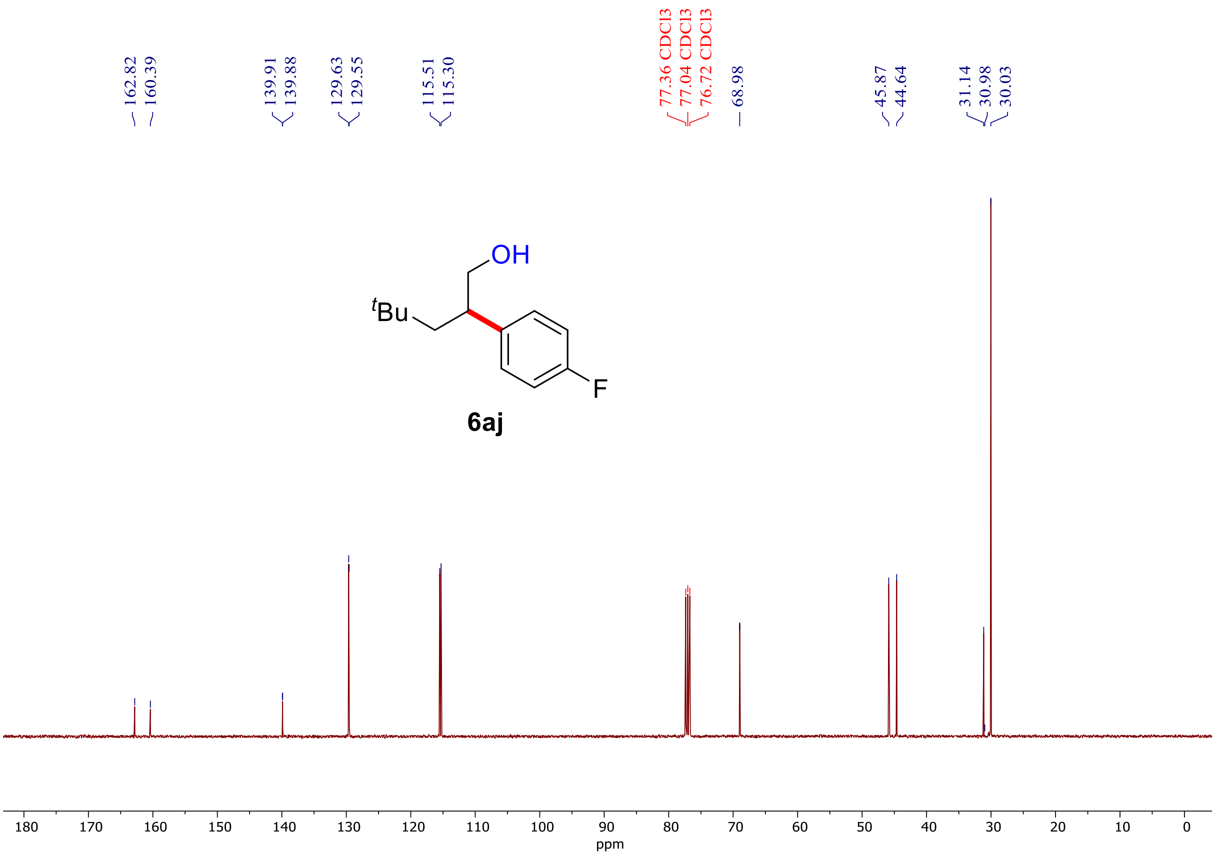


$^{19}F$ NMR (376 MHz, $CDCl_3$) of **6aj**

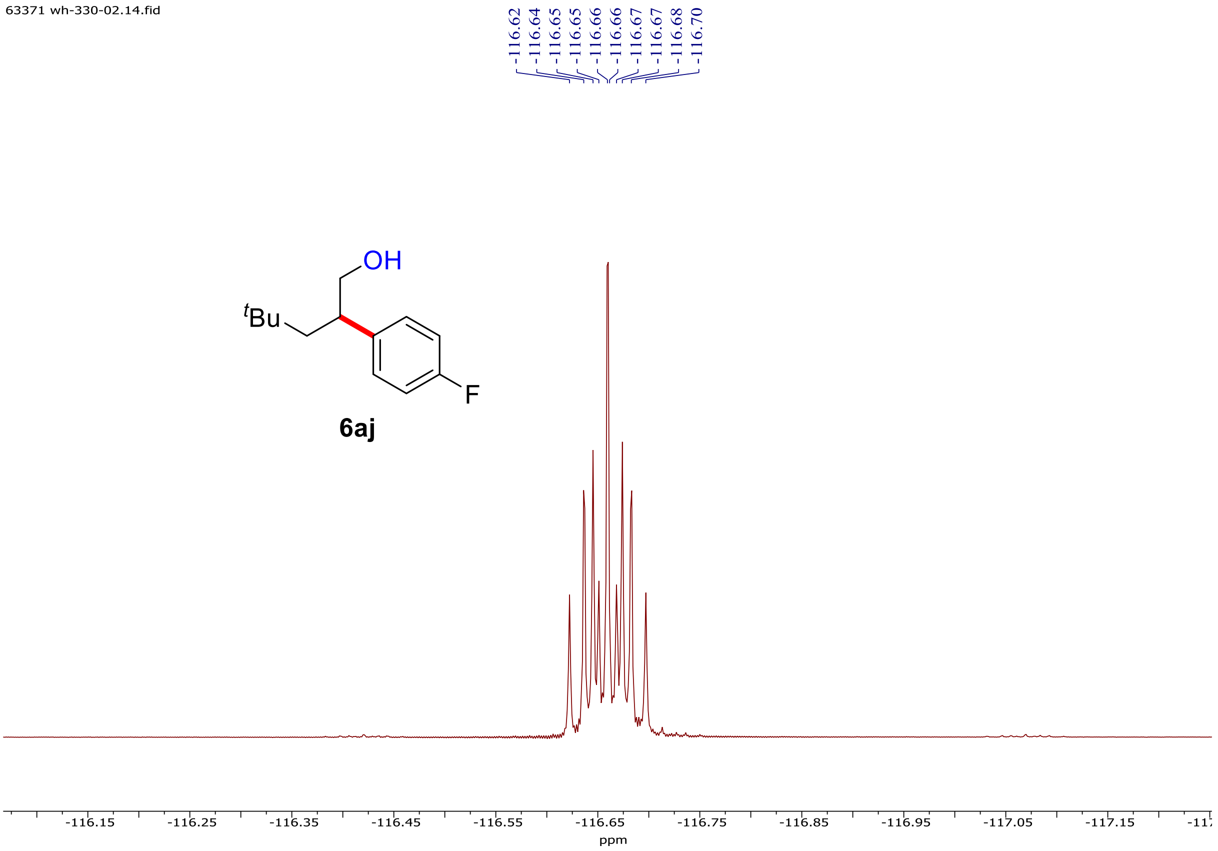

$^{1}$H NMR (400 MHz, $CDCl_3$) of **6ak** (*see procedure*)

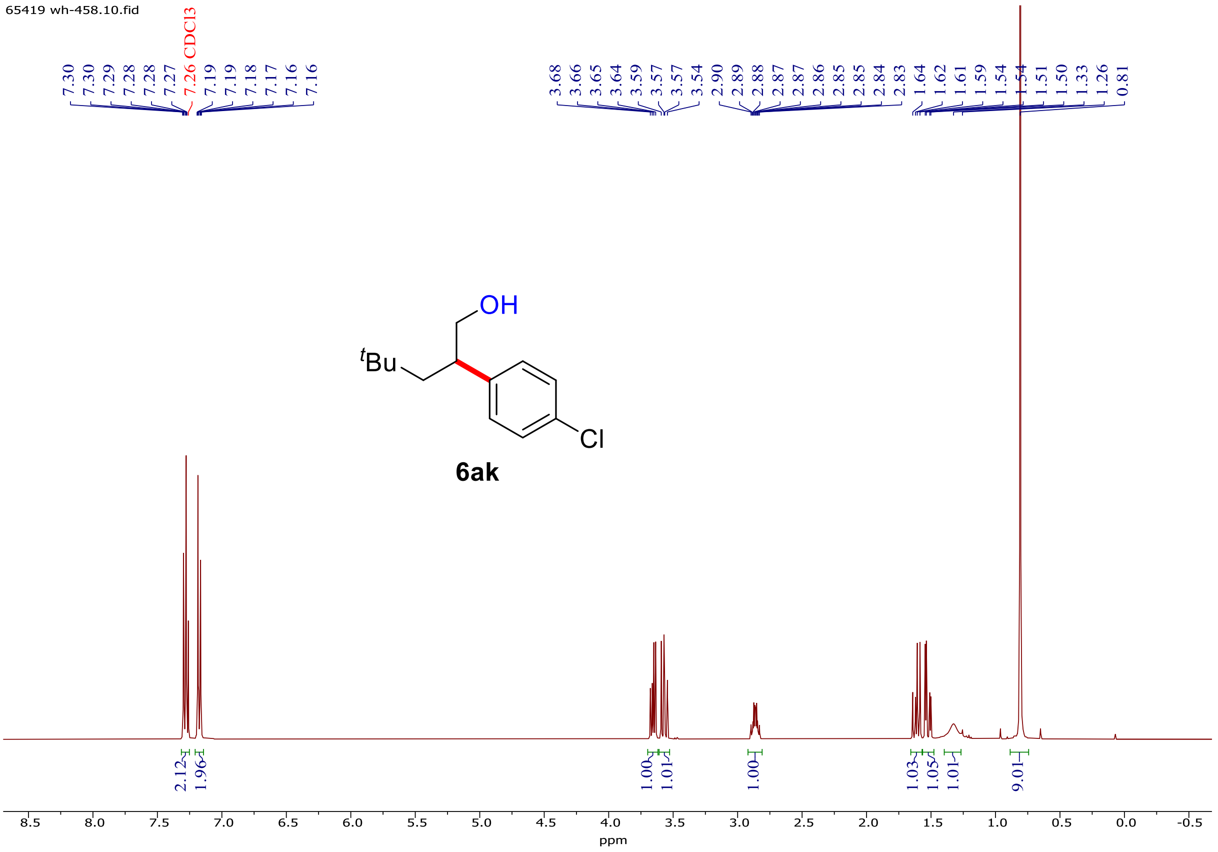


$^{13}$C NMR (101 MHz, $CDCl_3$) of **6ak**

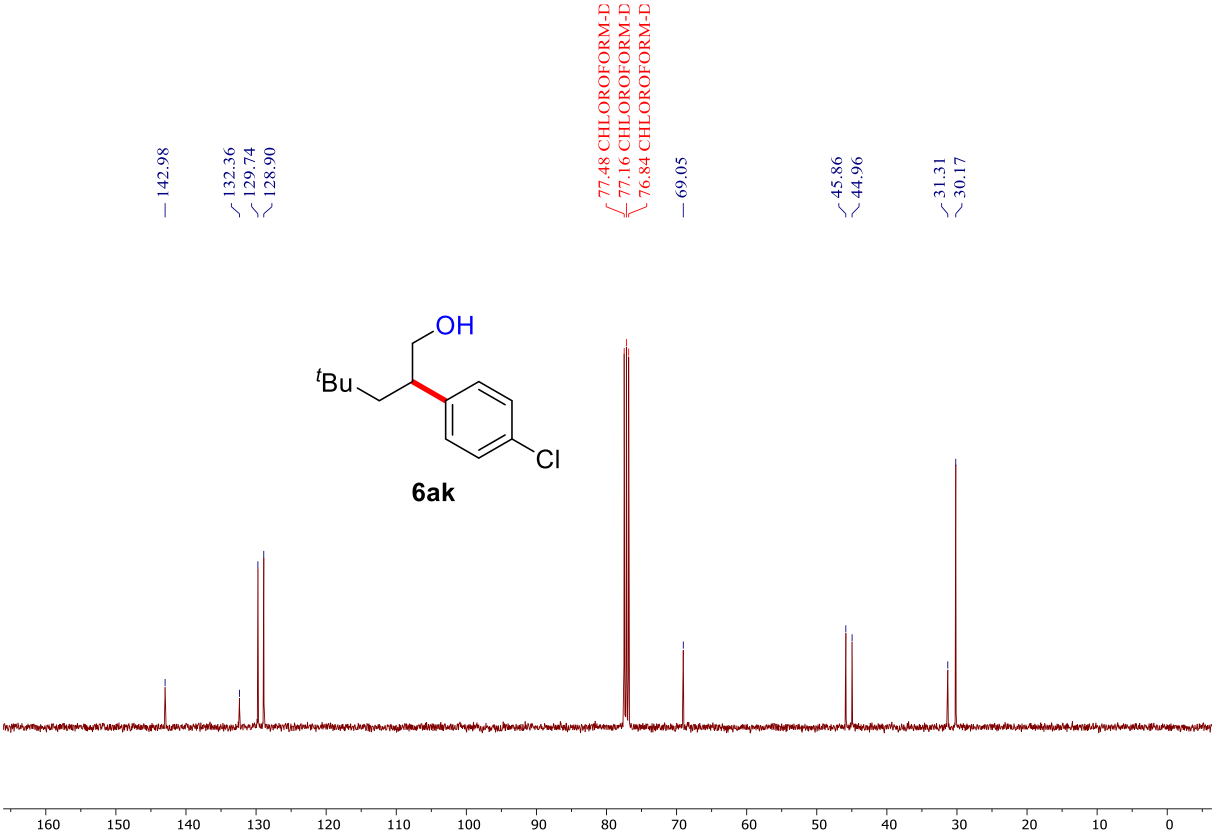

$^1$H NMR (400 MHz, $CDCl_3$) of **6al** (*see procedure*)

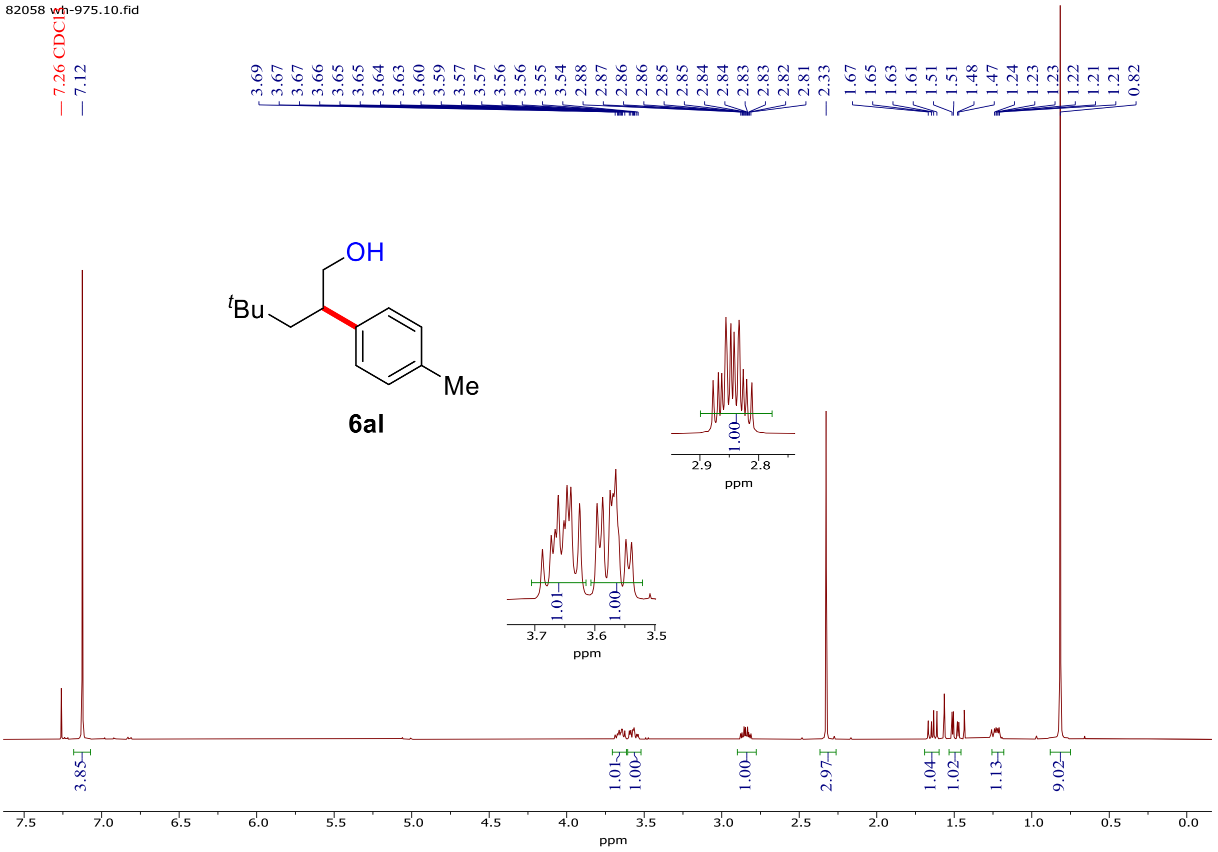

$^{13}$C NMR (101 MHz, $CDCl_3$) of **6al**

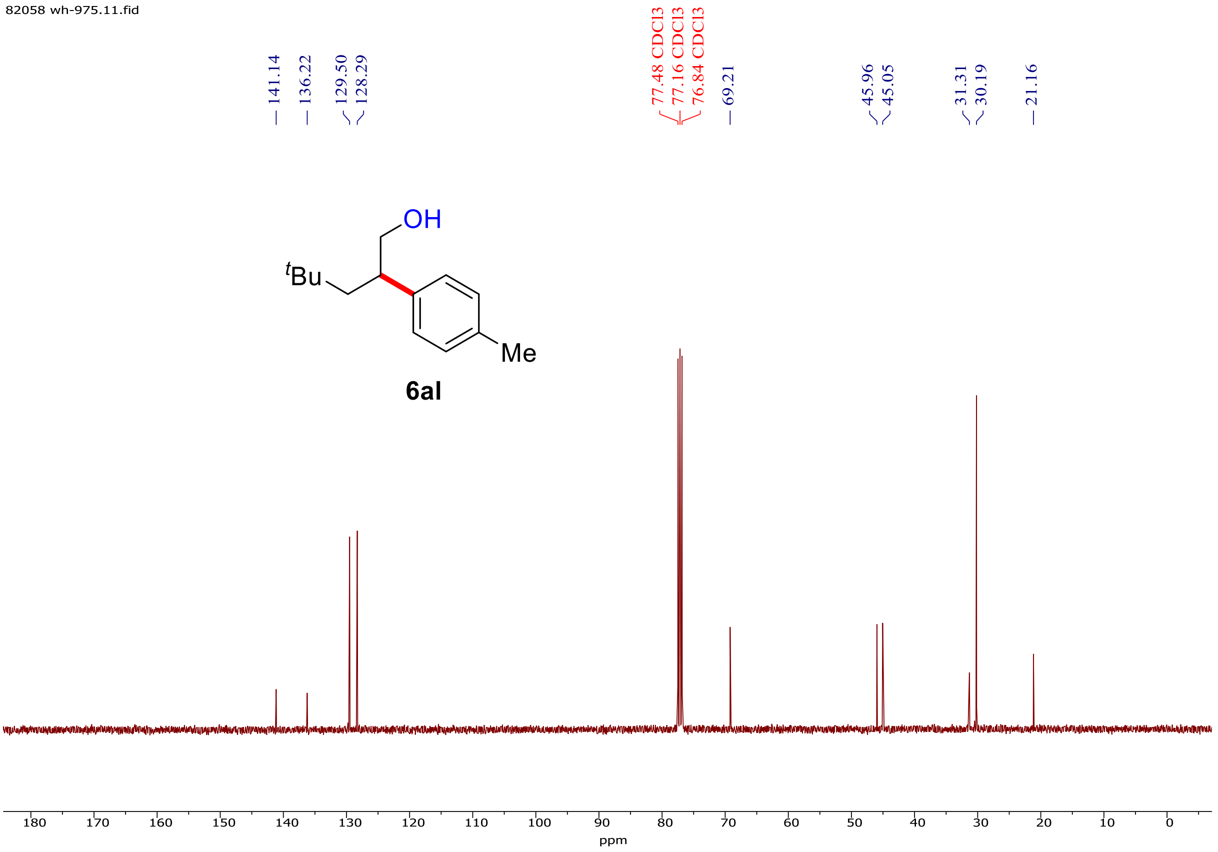

$^1$H NMR (400 MHz, $CDCl_3$) of **6am** (*see procedure*)

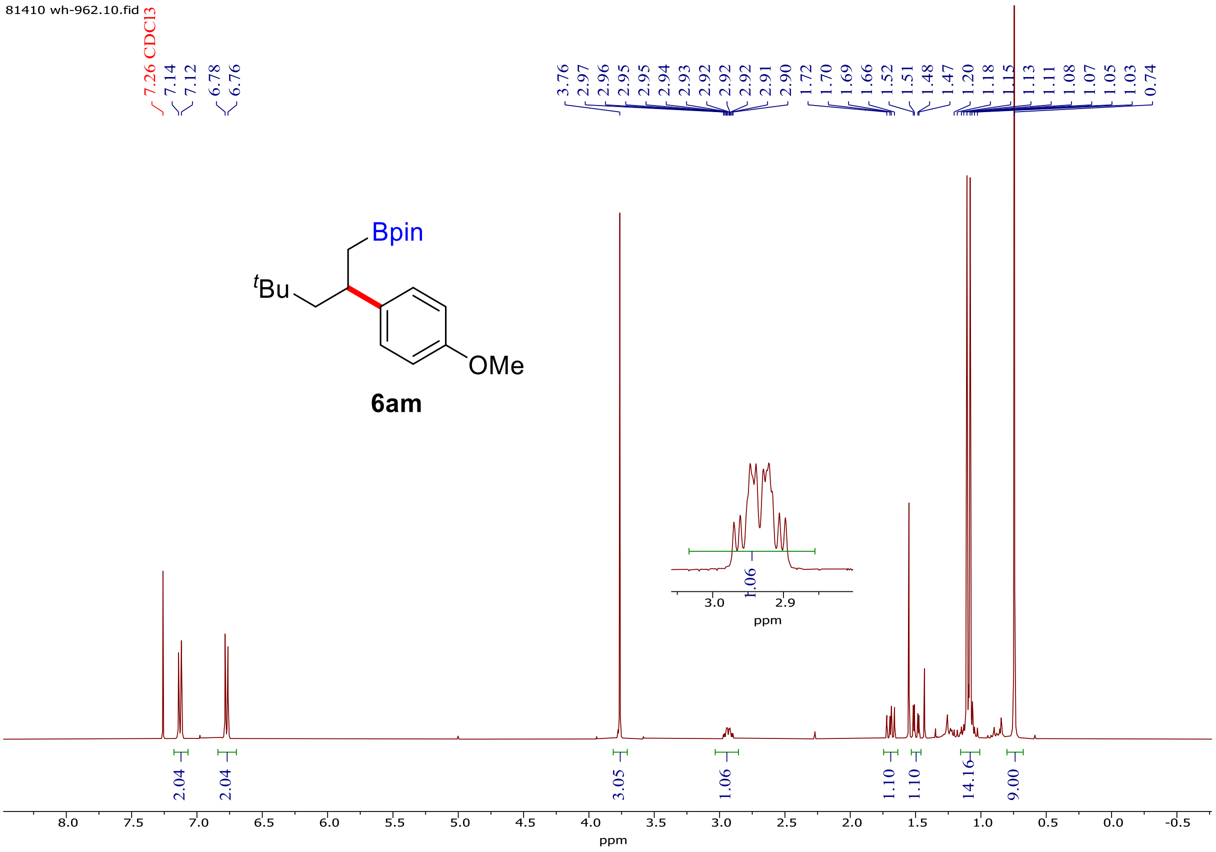

$^{13}C$ NMR (101 MHz, $CDCl_3$) of **6am**

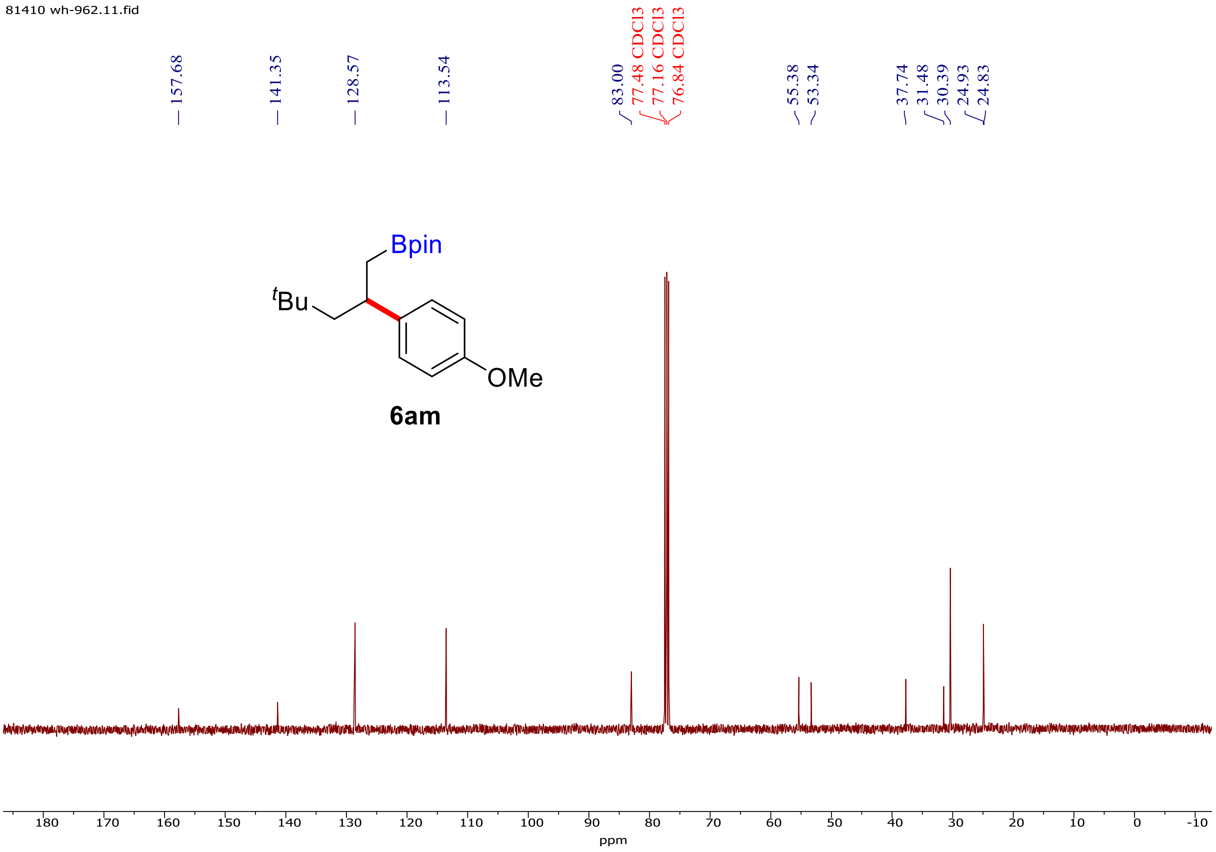


$^{11}B$ NMR (128 MHz, $CDCl_3$) of **6am**

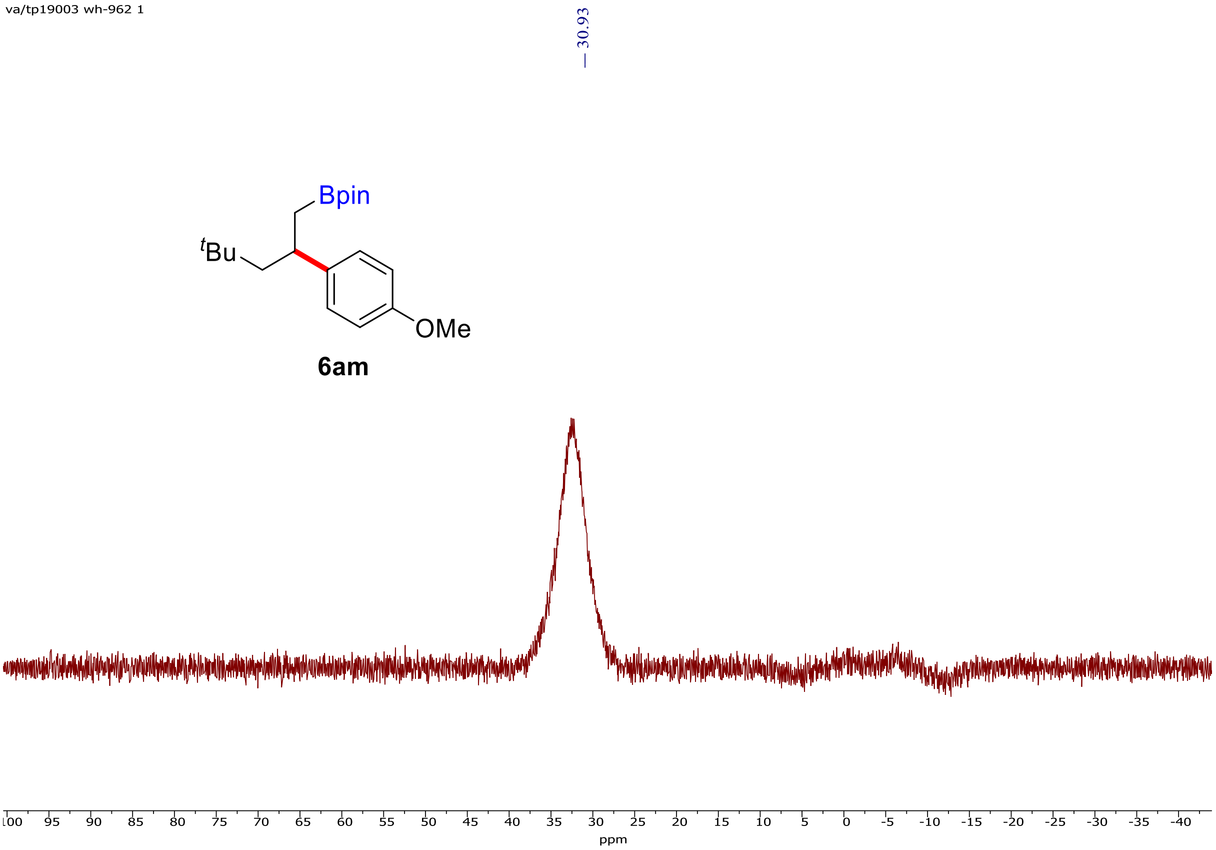

$^1$H NMR (400 MHz, $CDCl_3$) of **6an** (*see procedure*)

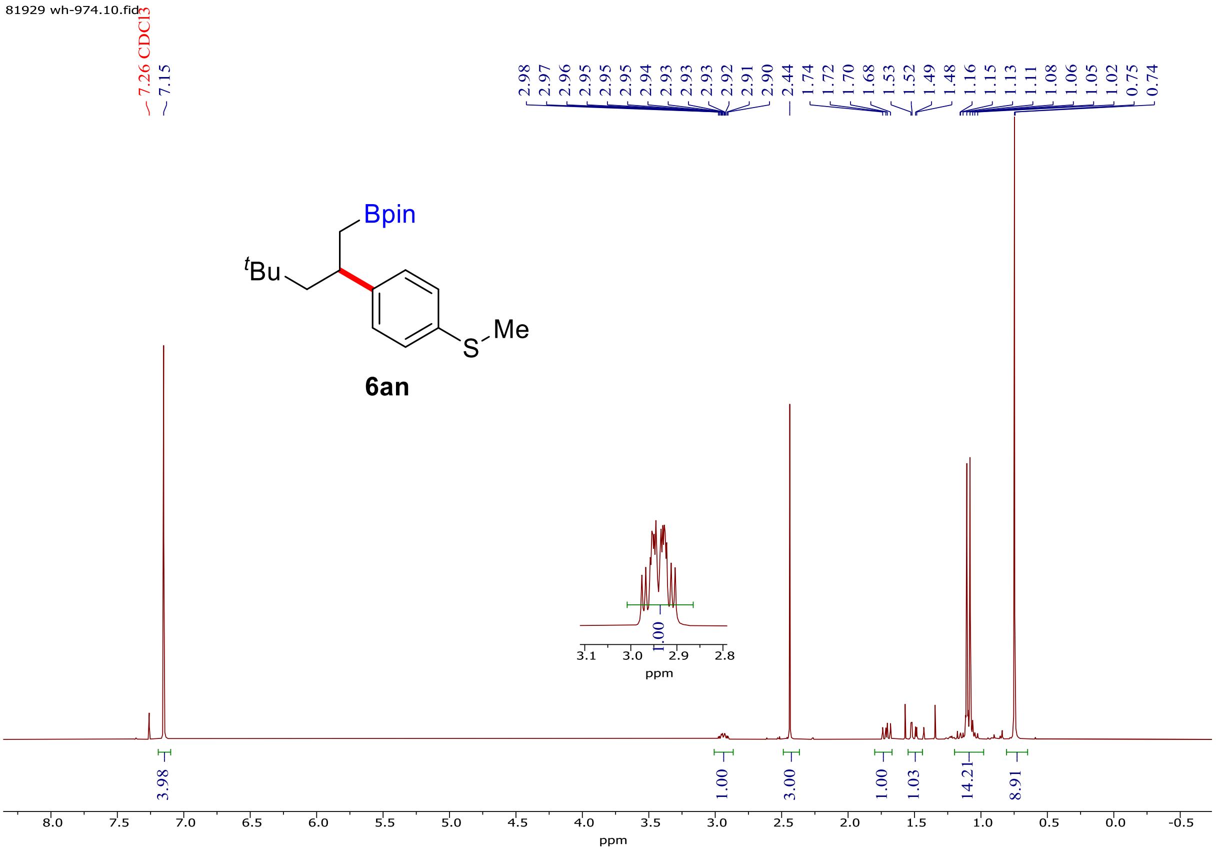

$^{13}$C NMR (101 MHz, $CDCl_3$) of **6an**

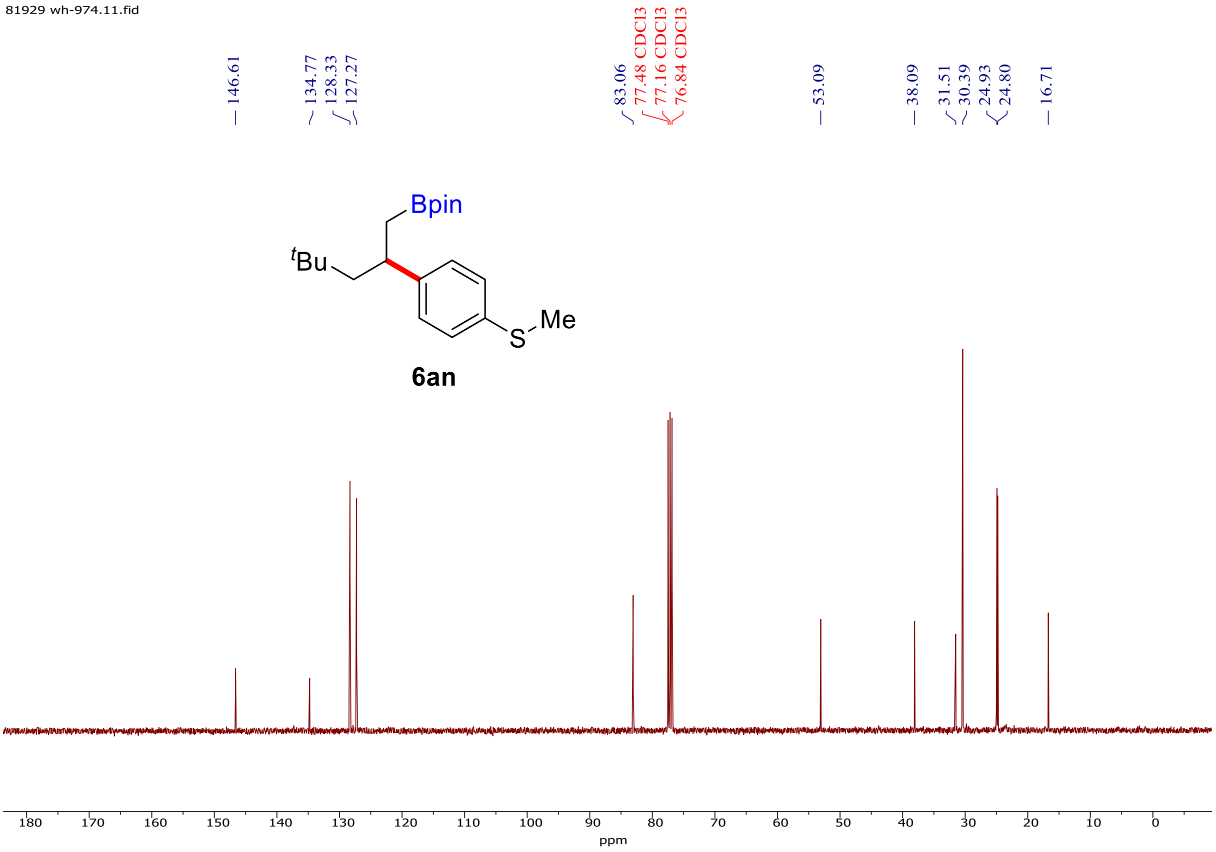

$^{11}$B NMR (128 MHz, $CDCl_3$) of **6an**

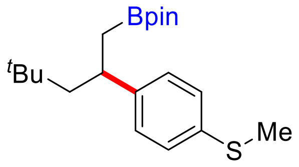


**6an**

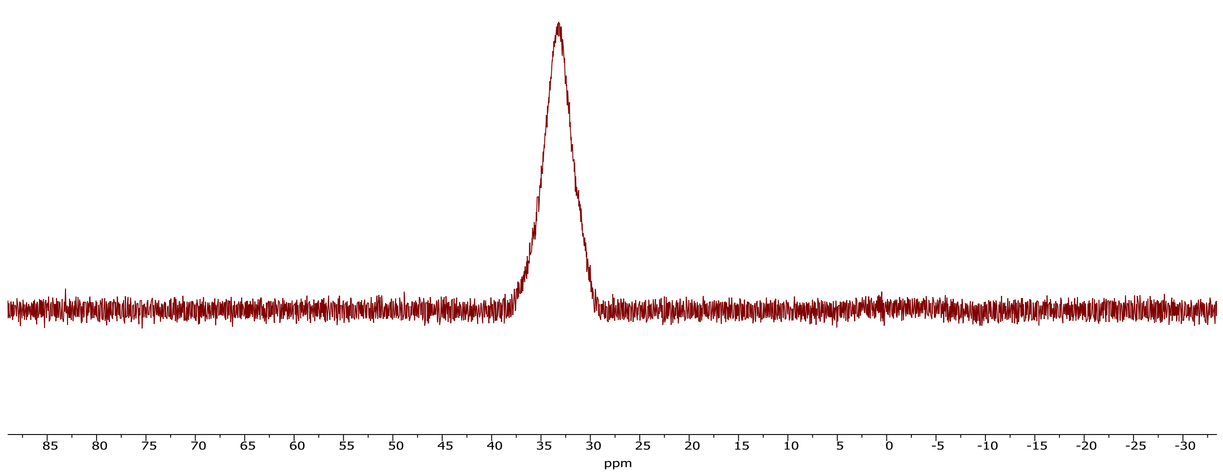

$^{1}$H NMR (400 MHz, $CDCl_3$) of **6ao** (*see procedure*)

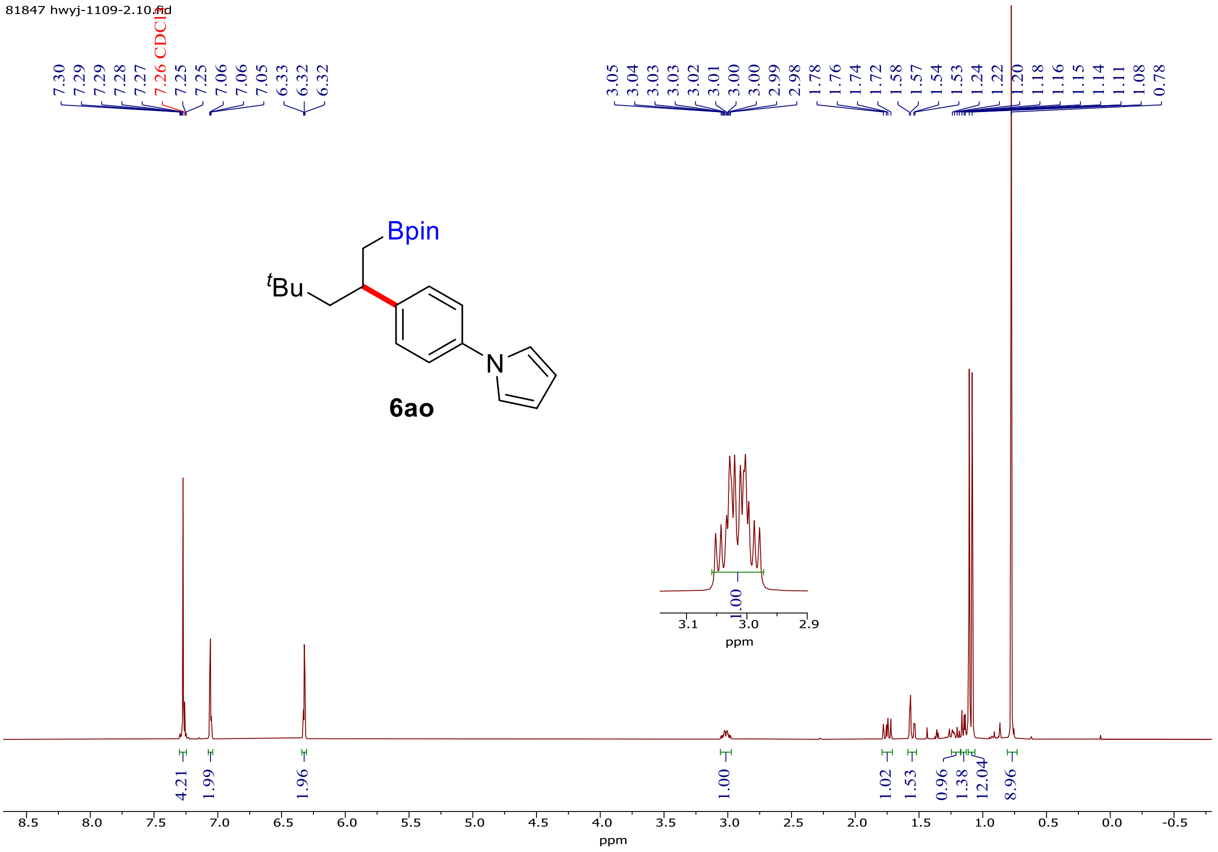

$^{13}C$ NMR (101 MHz, $CDCl_3$) of **6ao**

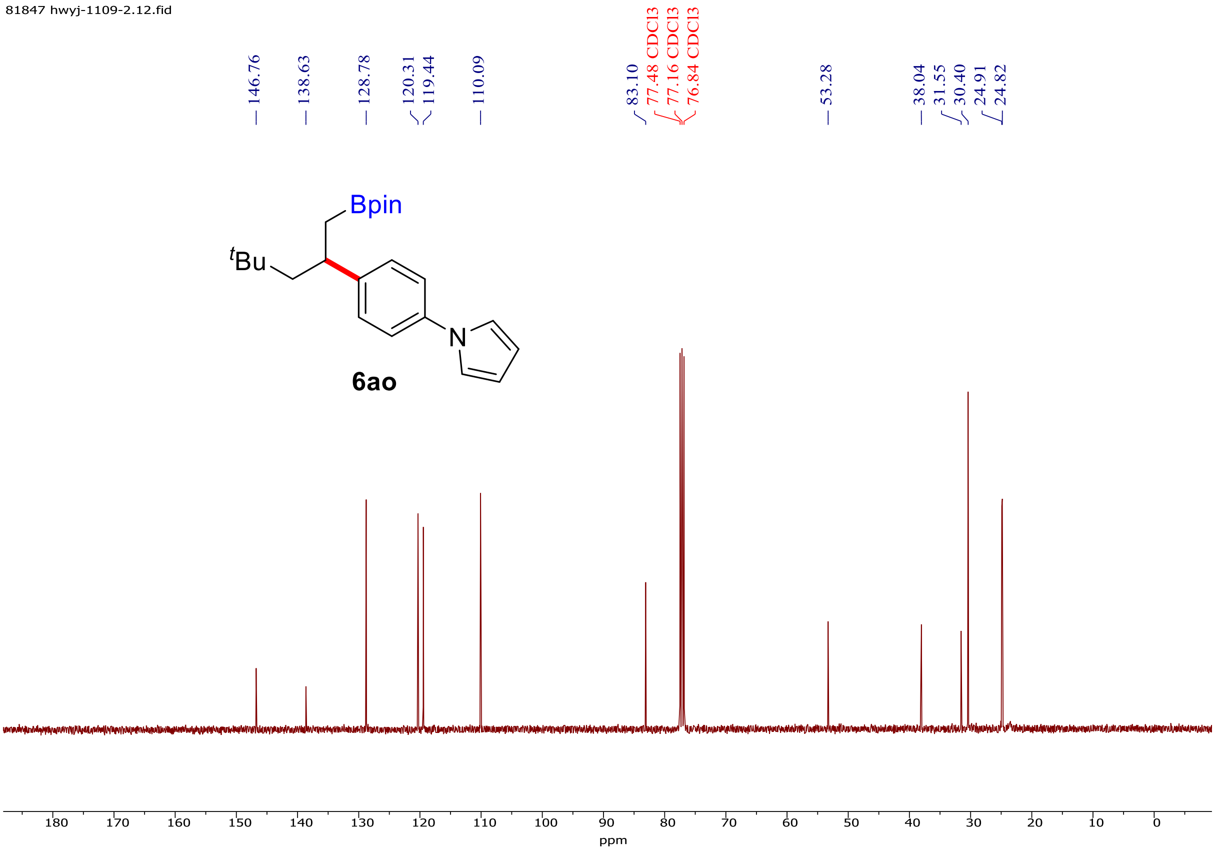

$^{11}$B NMR (128 MHz, $CDCl_3$) of **6ao**

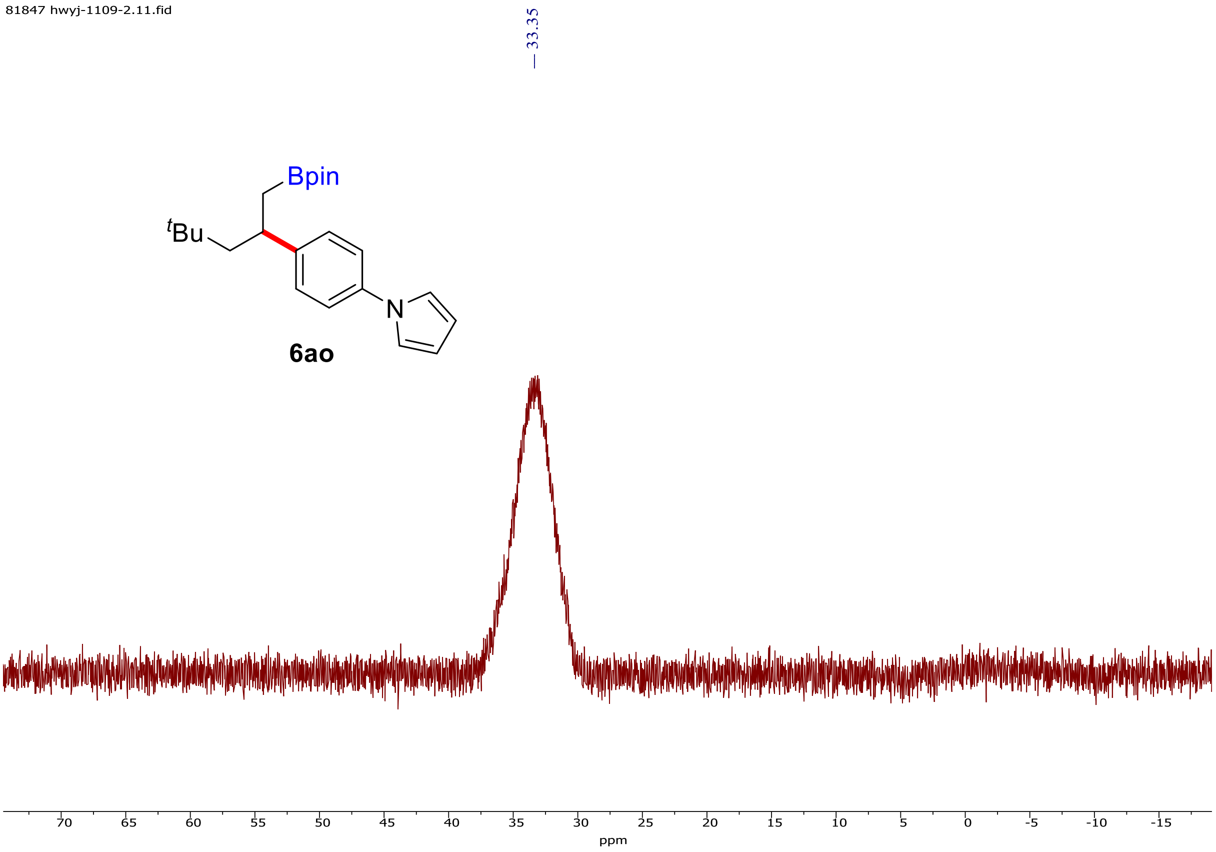


$^{1}$H NMR (400 MHz, $CDCl_3$) of **6ap** (see procedure)

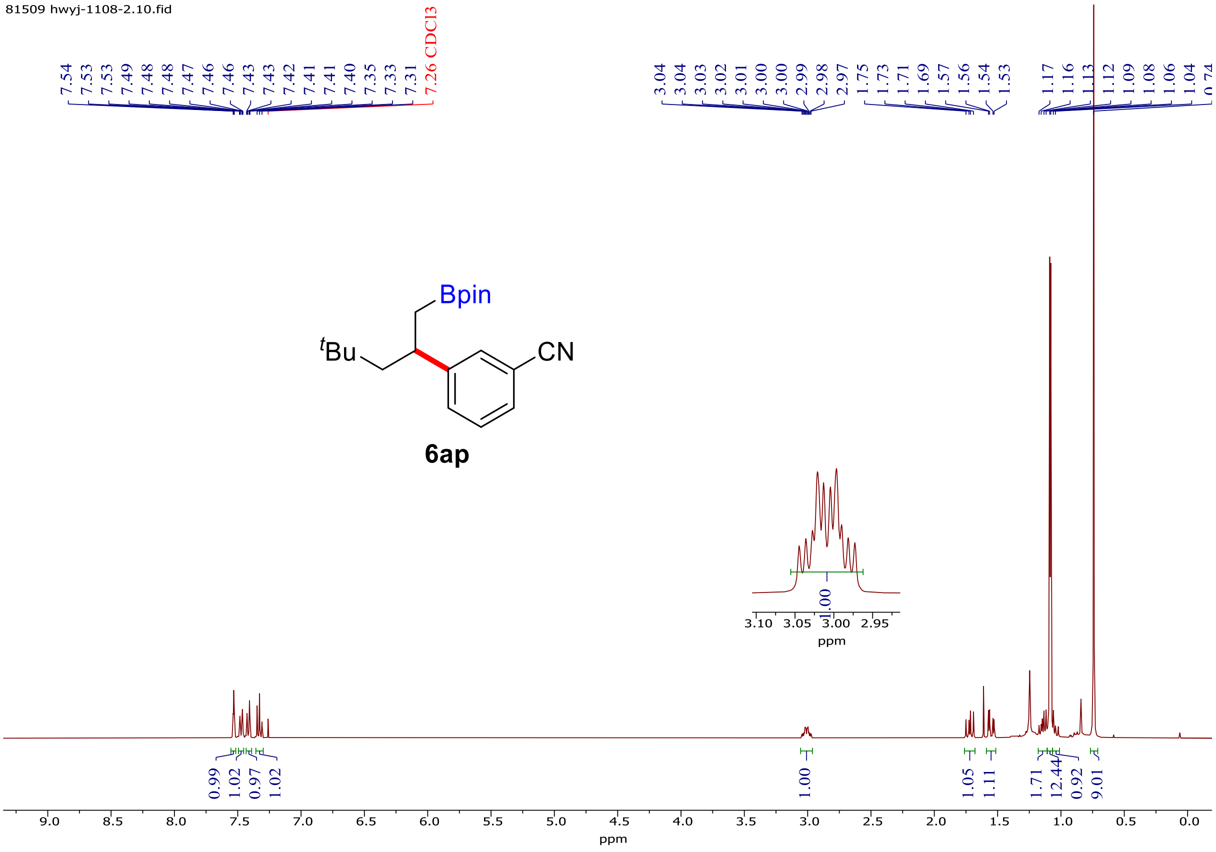

$^{13}$C NMR (101 MHz, $CDCl_3$) of **6ap**

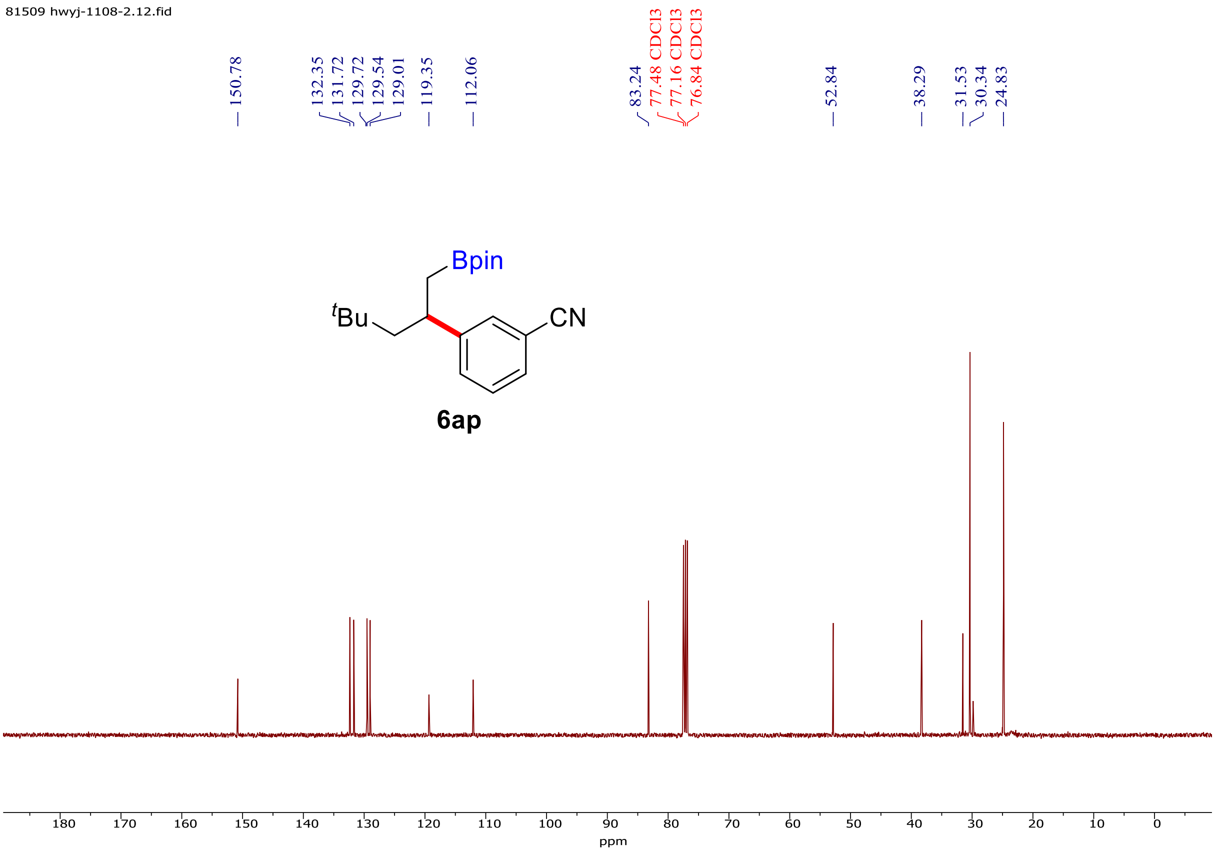


$^{11}$B NMR (128 MHz, $CDCl_3$) of **6ap**

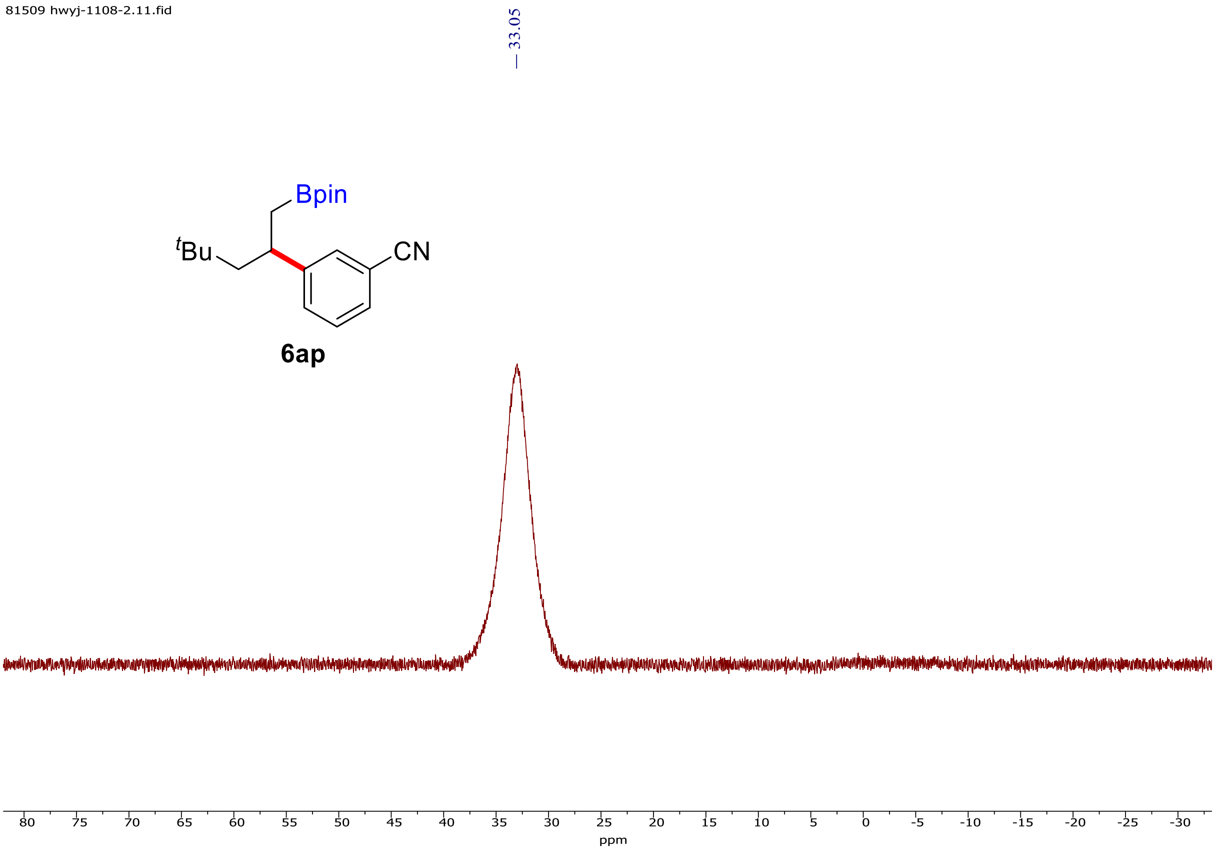

$^{1}$H NMR (400 MHz, $CDCl_3$) of **6aq** (see procedure)

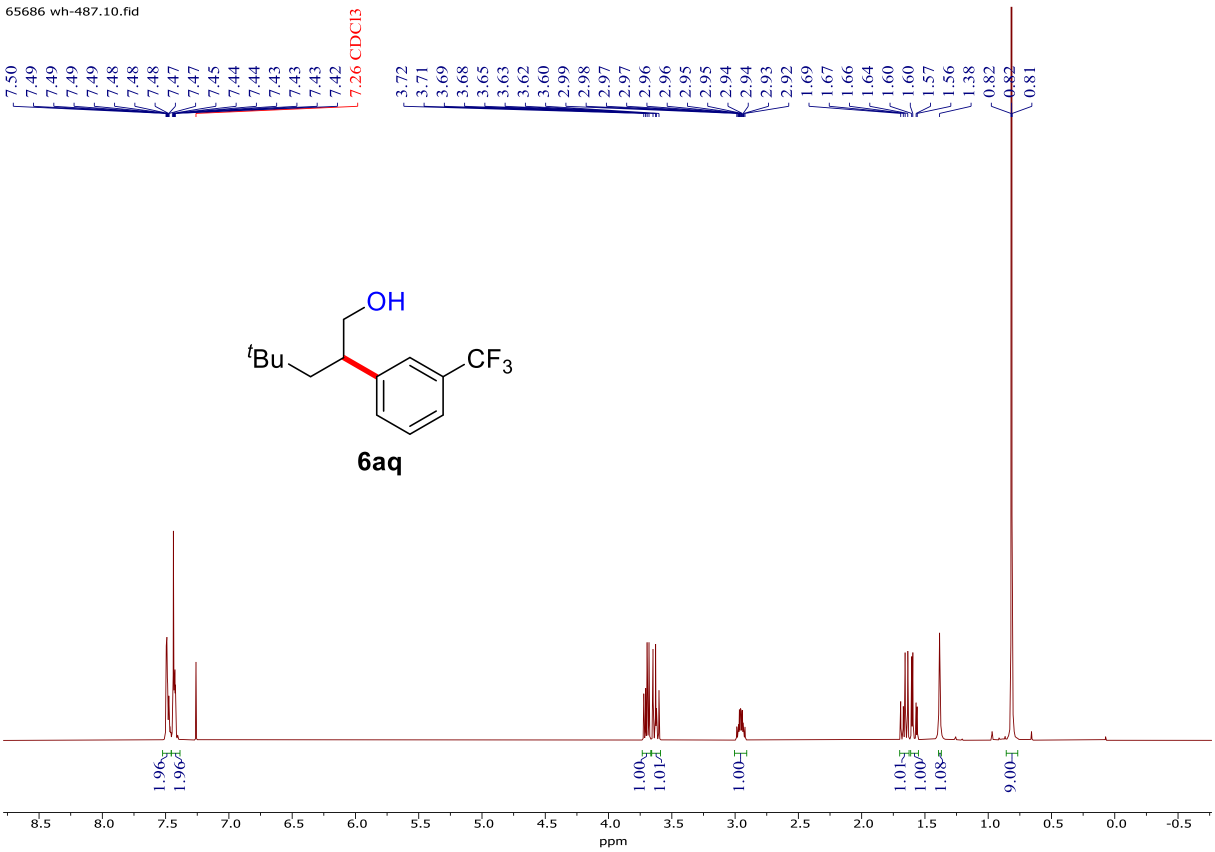


$^{13}$C NMR (101 MHz, $CDCl_3$) of **6aq**

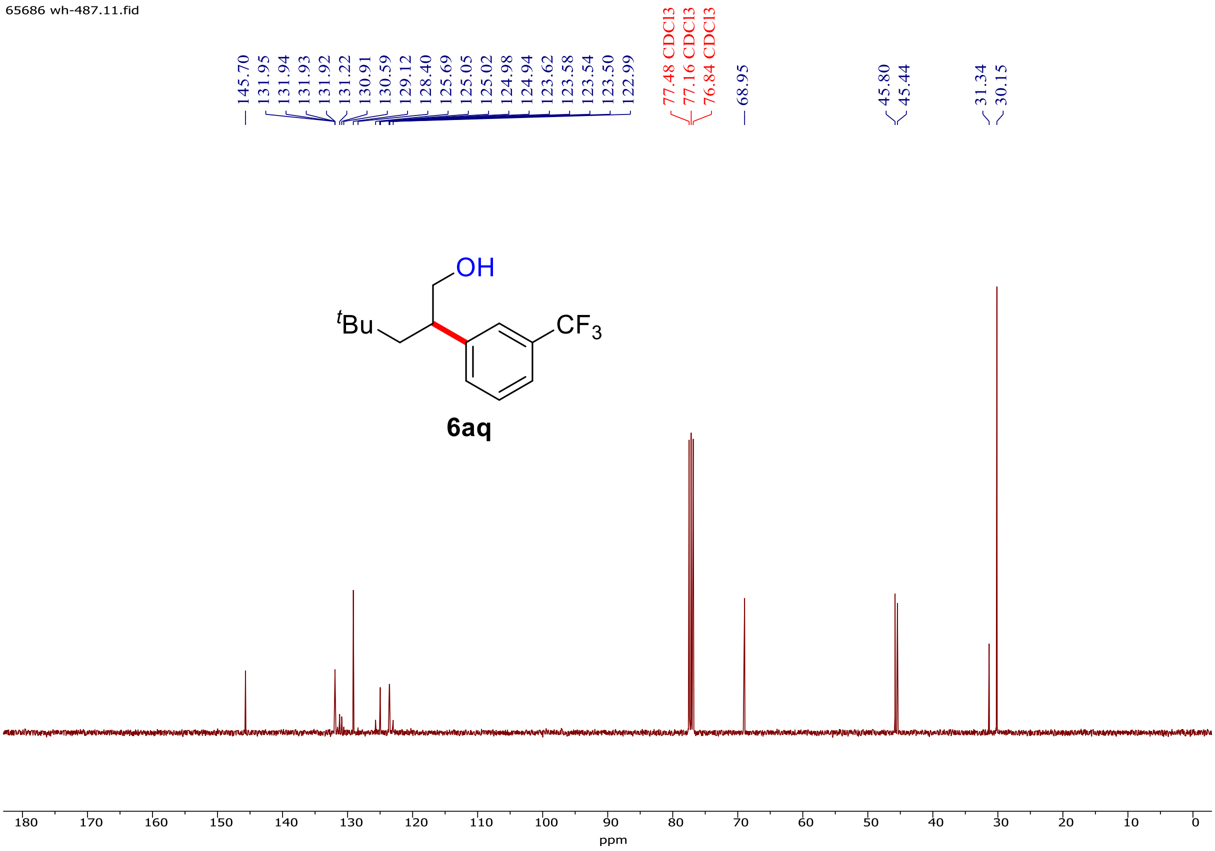

$^{19}F$ NMR (376 MHz, $CDCl_3$) of **6aq**

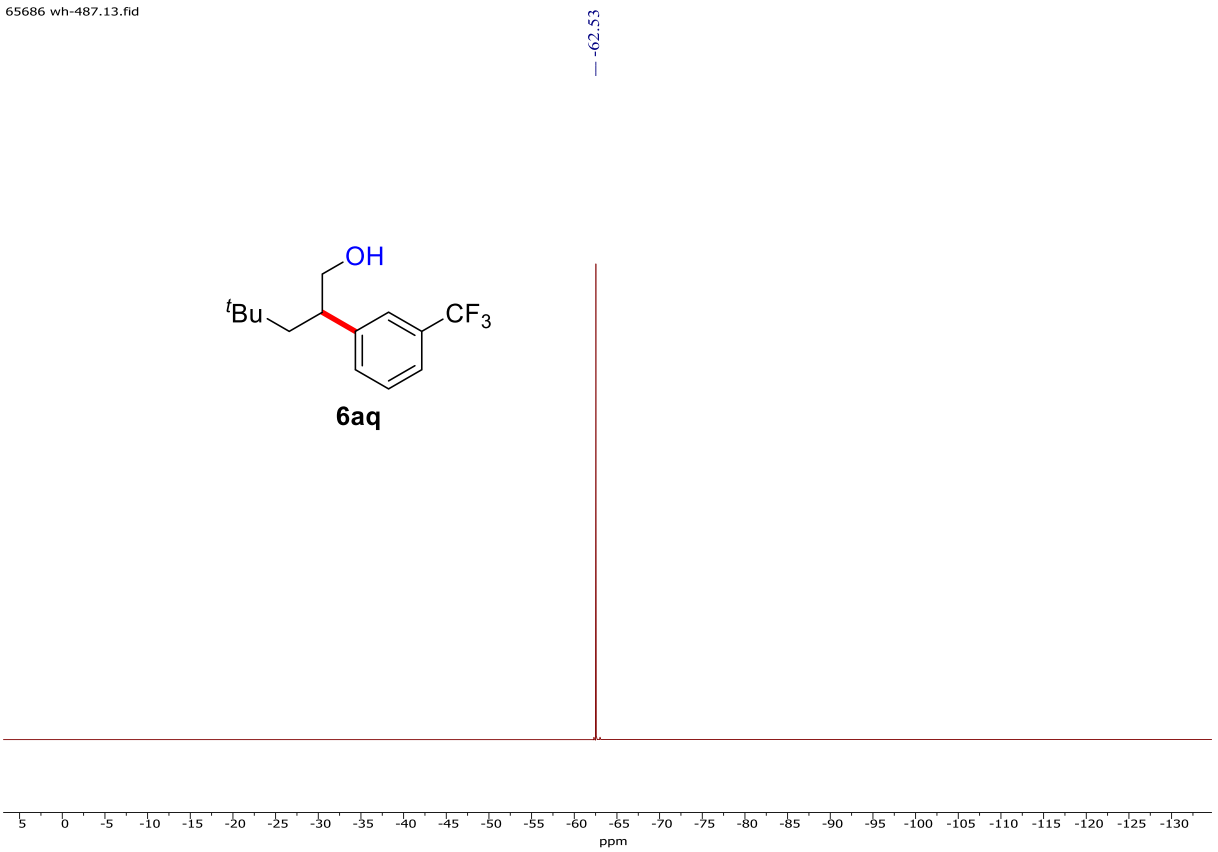


$^{1}H$ NMR (400 MHz, $CDCl_3$) of **6ar** (*see procedure*)

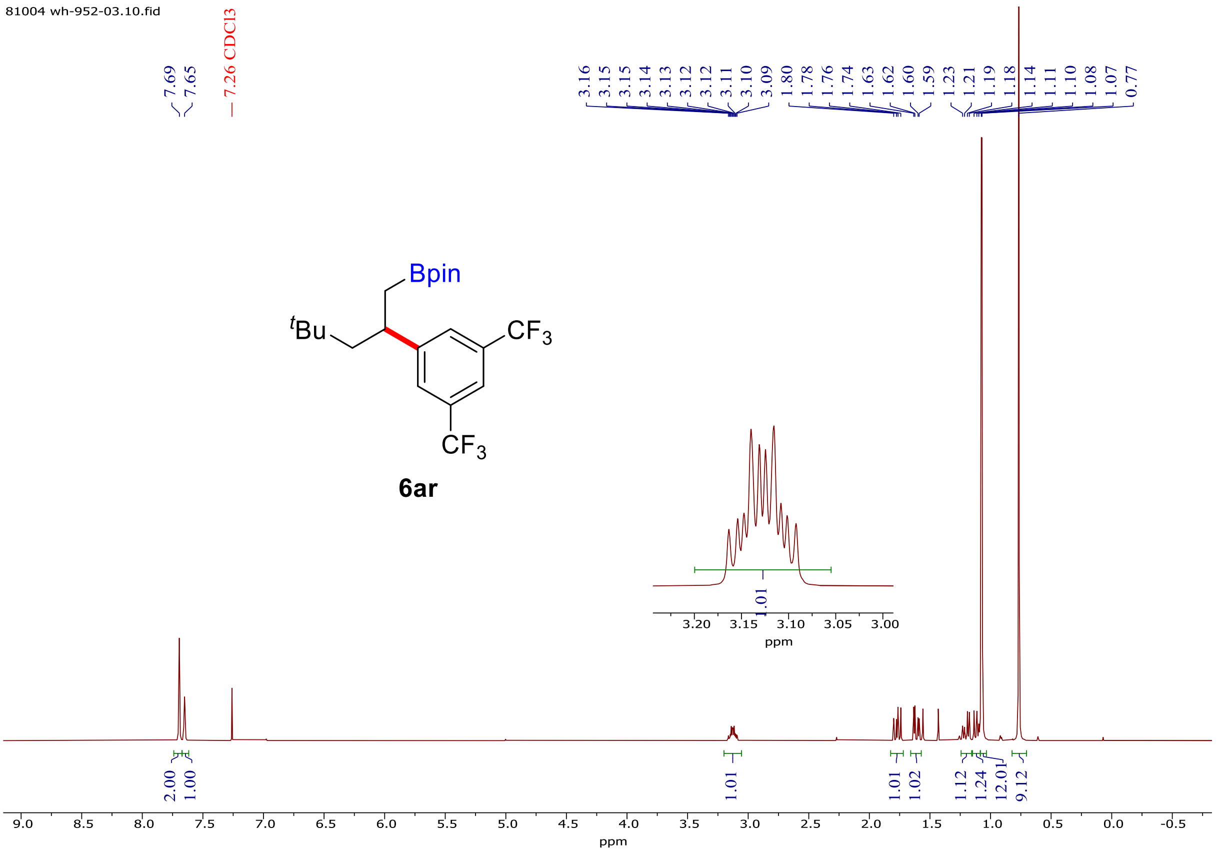

$^{13}C$ NMR (101 MHz, $CDCl_3$) of **6ar**

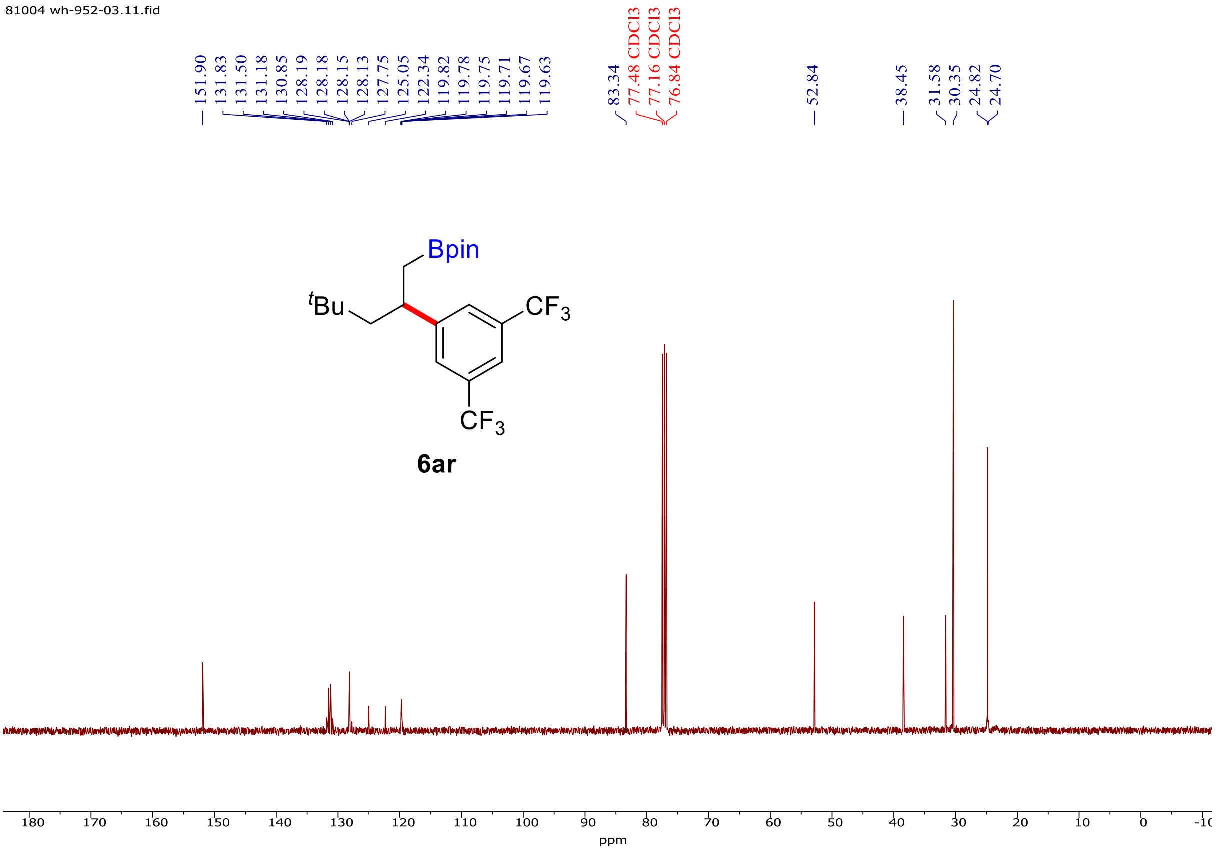

$^{19}F$ NMR (376 MHz, $CDCl_3$) of **6ar**

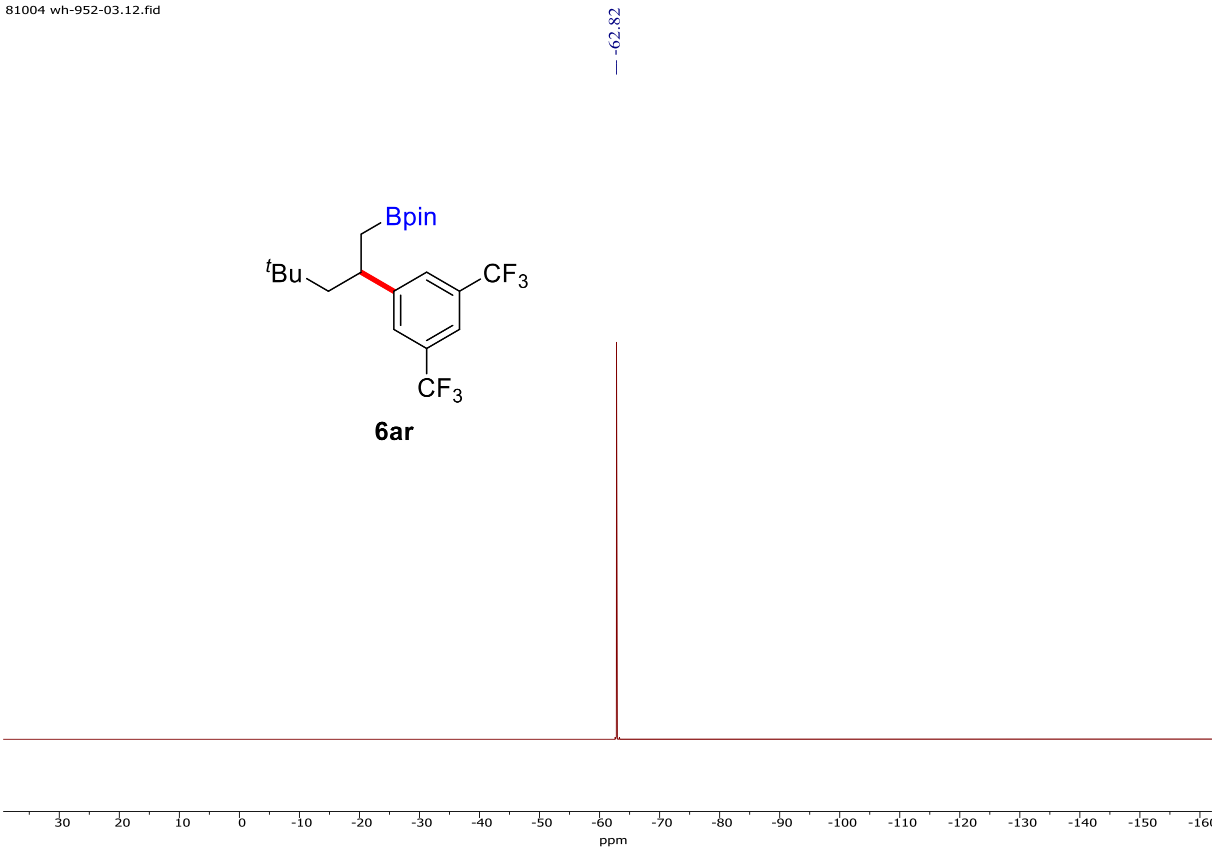

$^{11}$B NMR (128 MHz, $CDCl_3$) of **6ar**

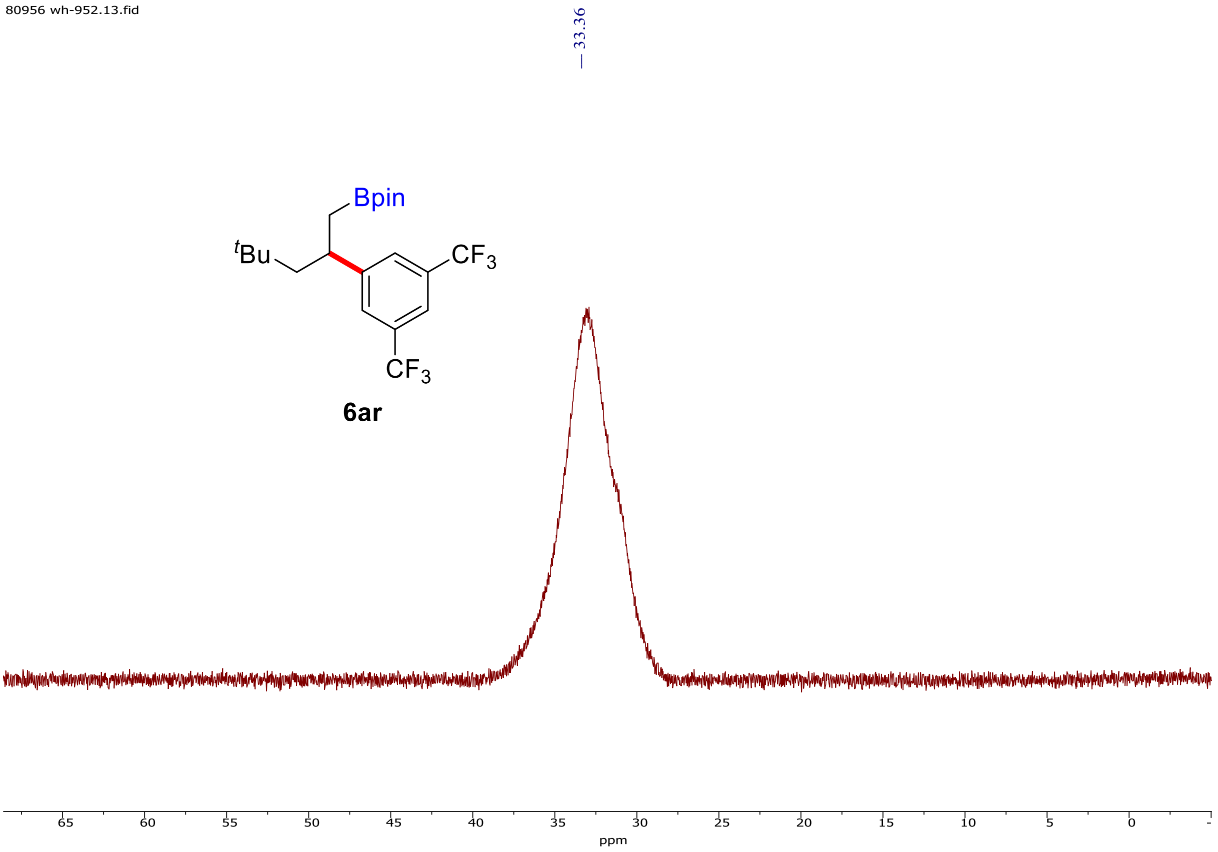

$^{1}$H NMR (400 MHz, $CDCl_3$) of **6as** (*see procedure*)

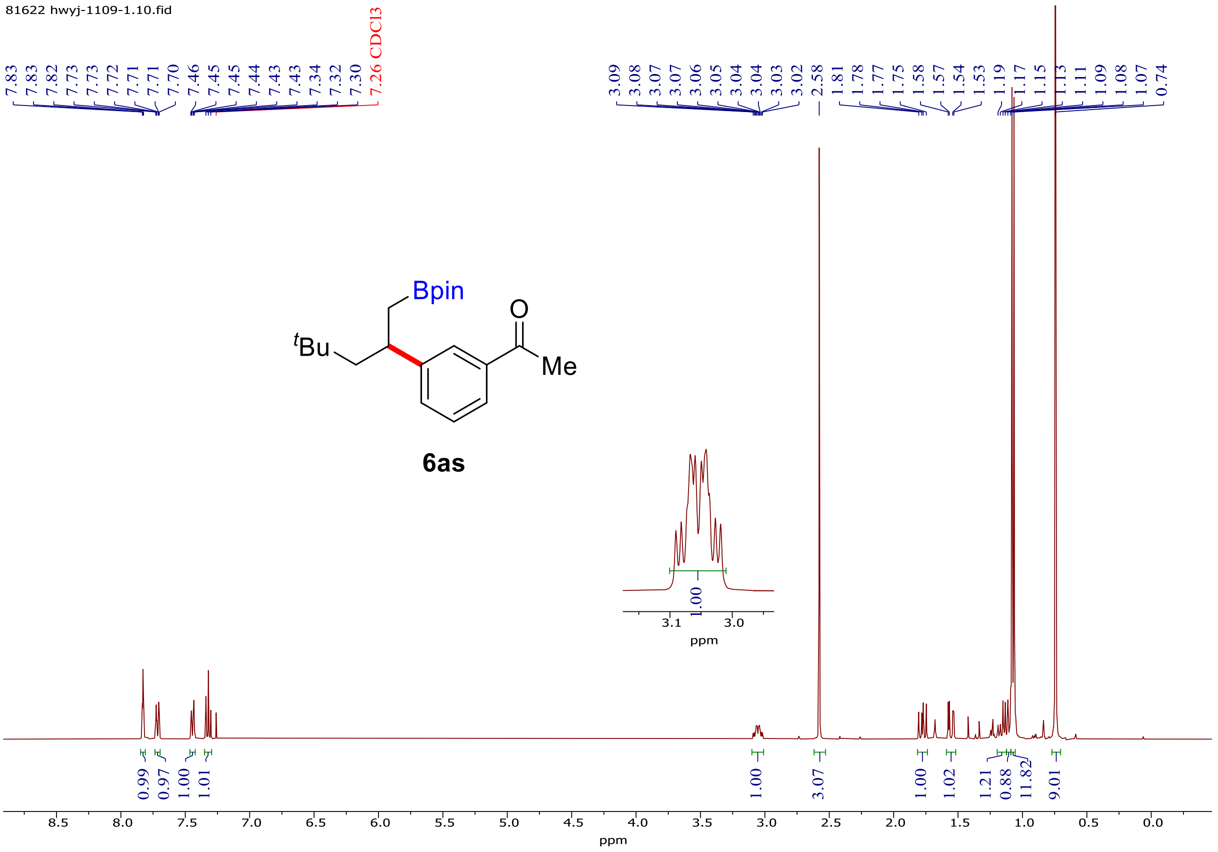

$^{13}C$ NMR (101 MHz, $CDCl_3$) of **6as**

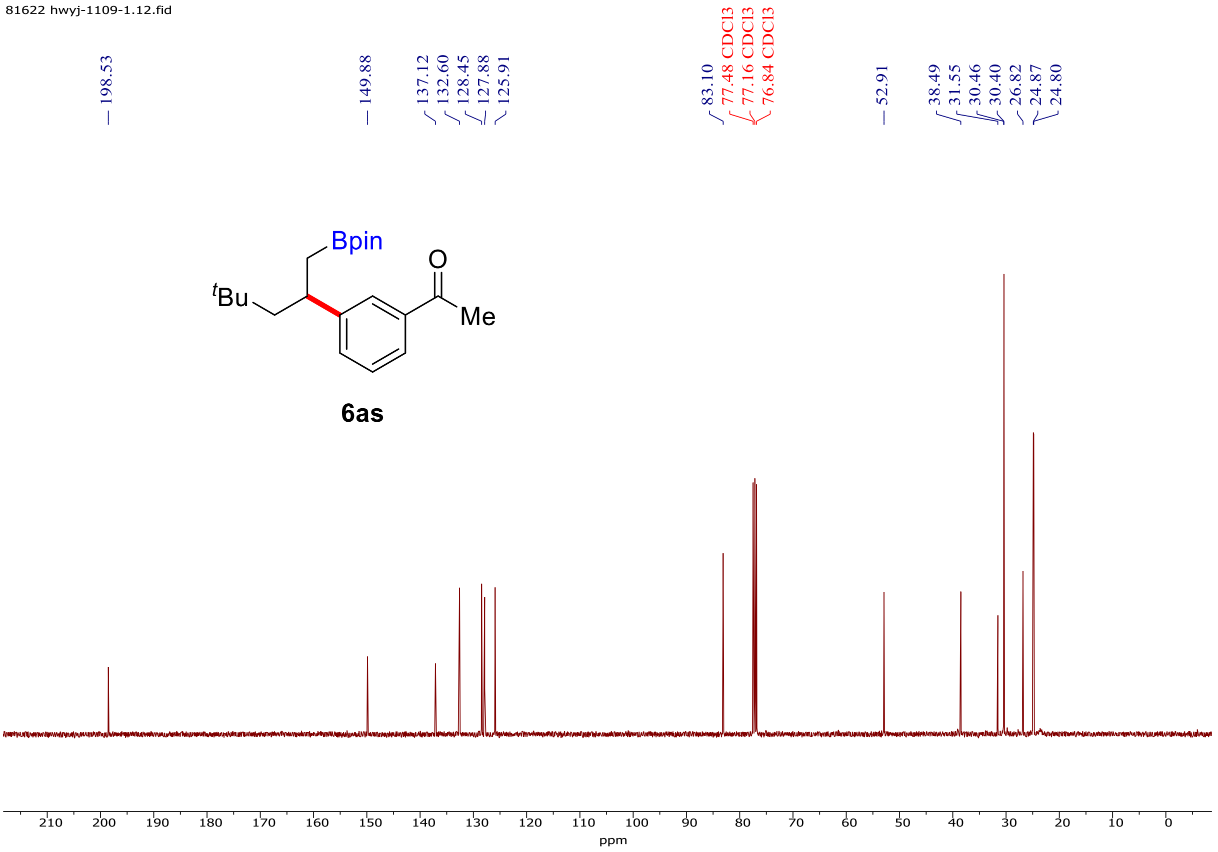

$^{11}$B NMR (128 MHz, $CDCl_3$) of **6as**

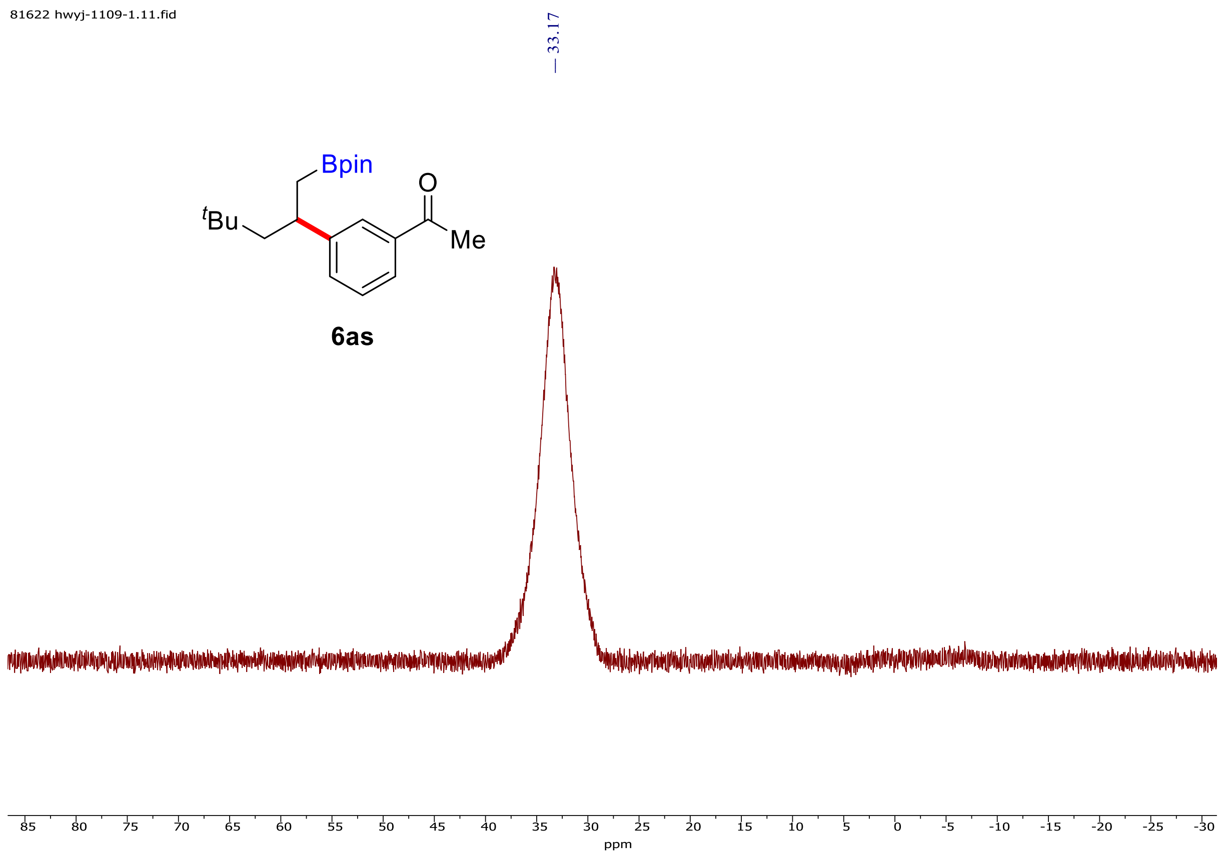

$^{1}H$ NMR (400 MHz, $CDCl_3$) of **6at** (*see procedure*)

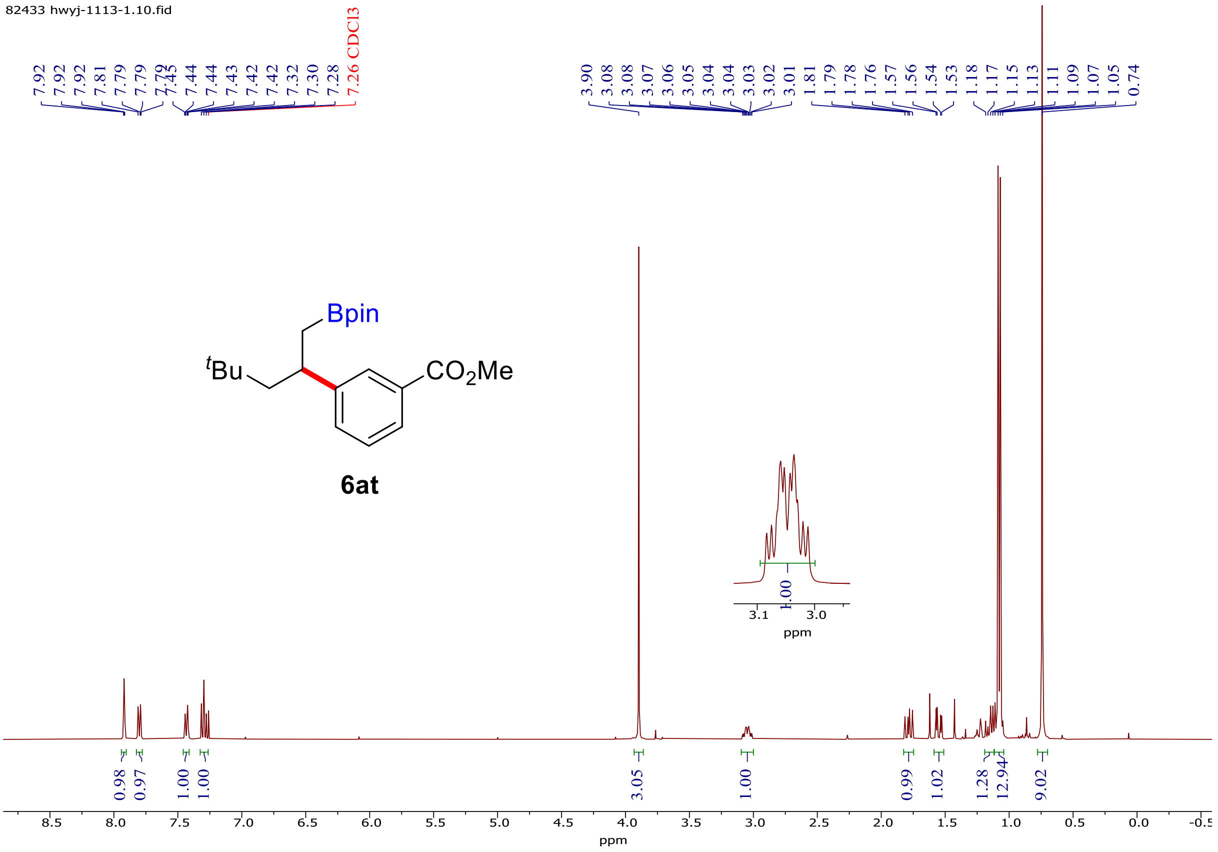

$^{13}C$ NMR (101 MHz, $CDCl_3$) of **6at**

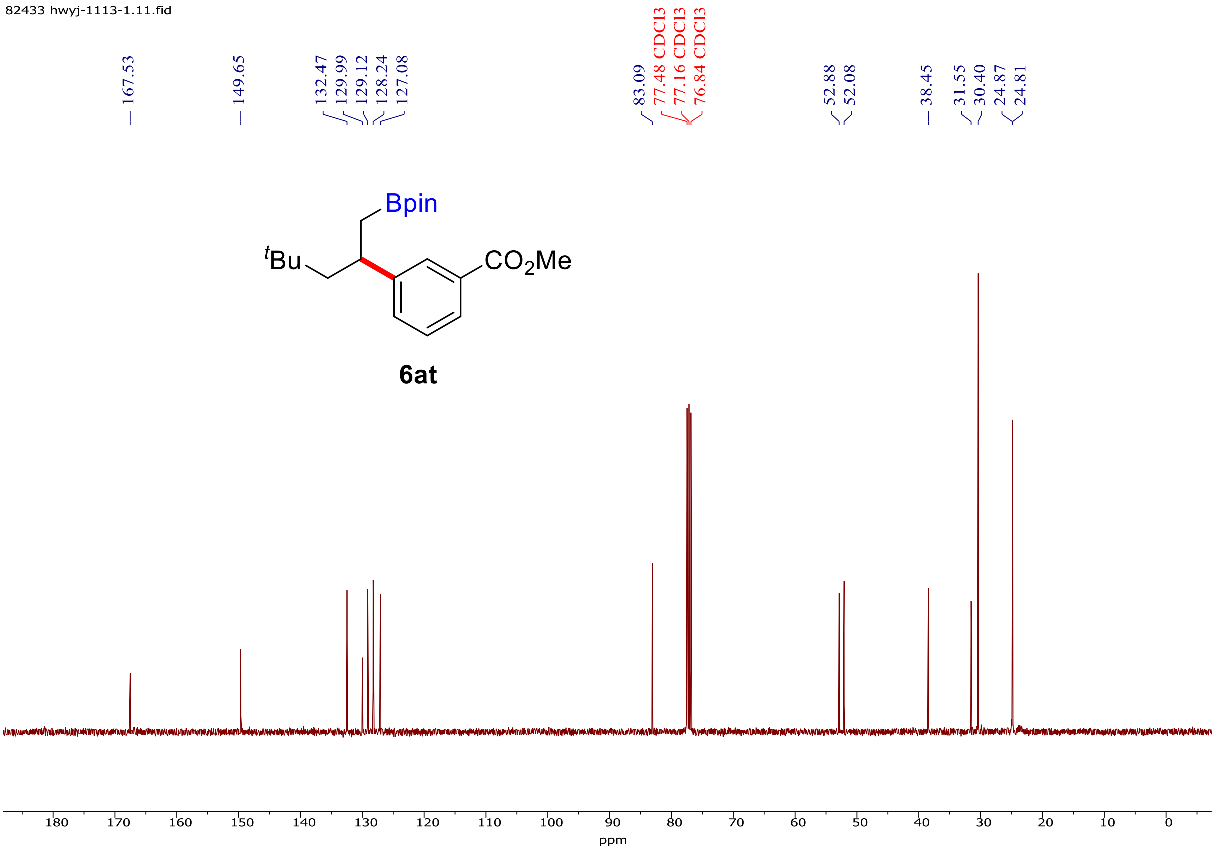

$^{11}$B NMR (128 MHz, $CDCl_3$) of **6at**

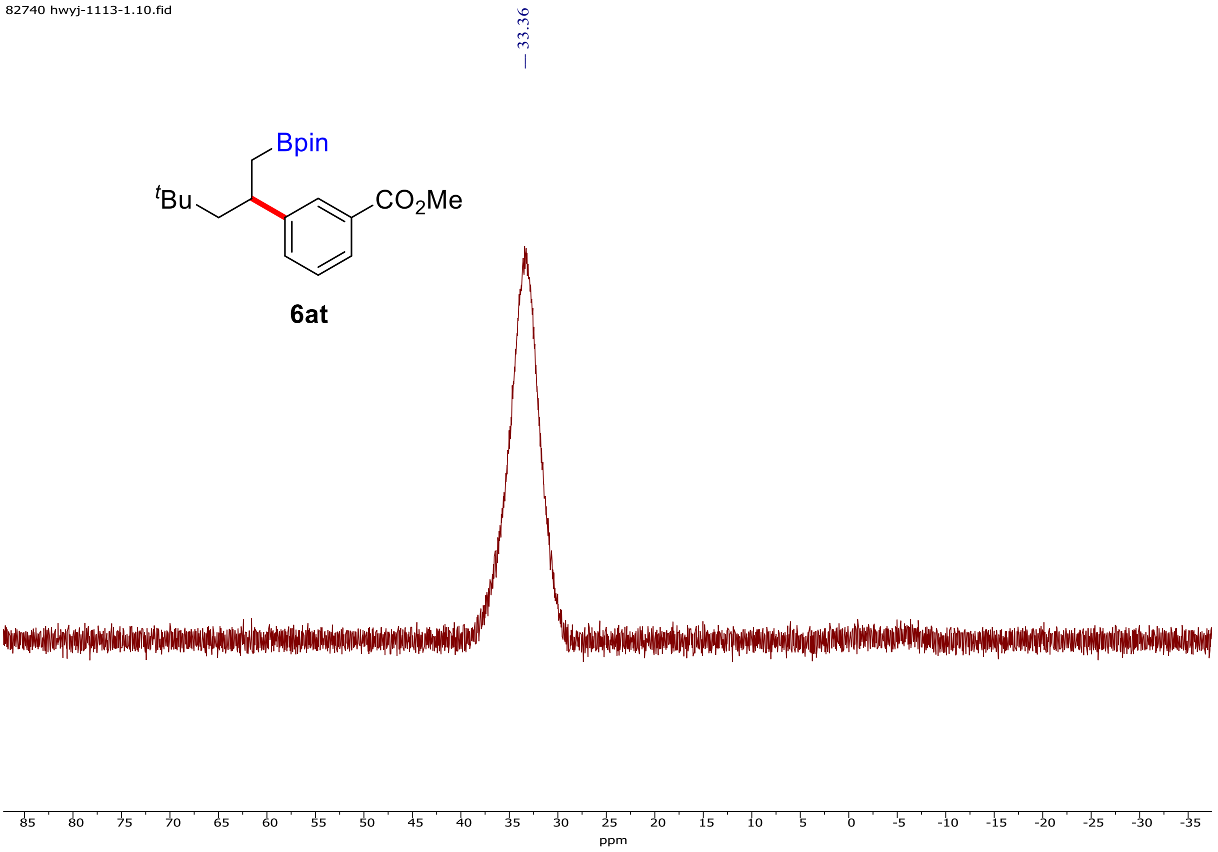


$^{1}$H NMR (400 MHz, $CDCl_3$) of **6au** (see procedure)

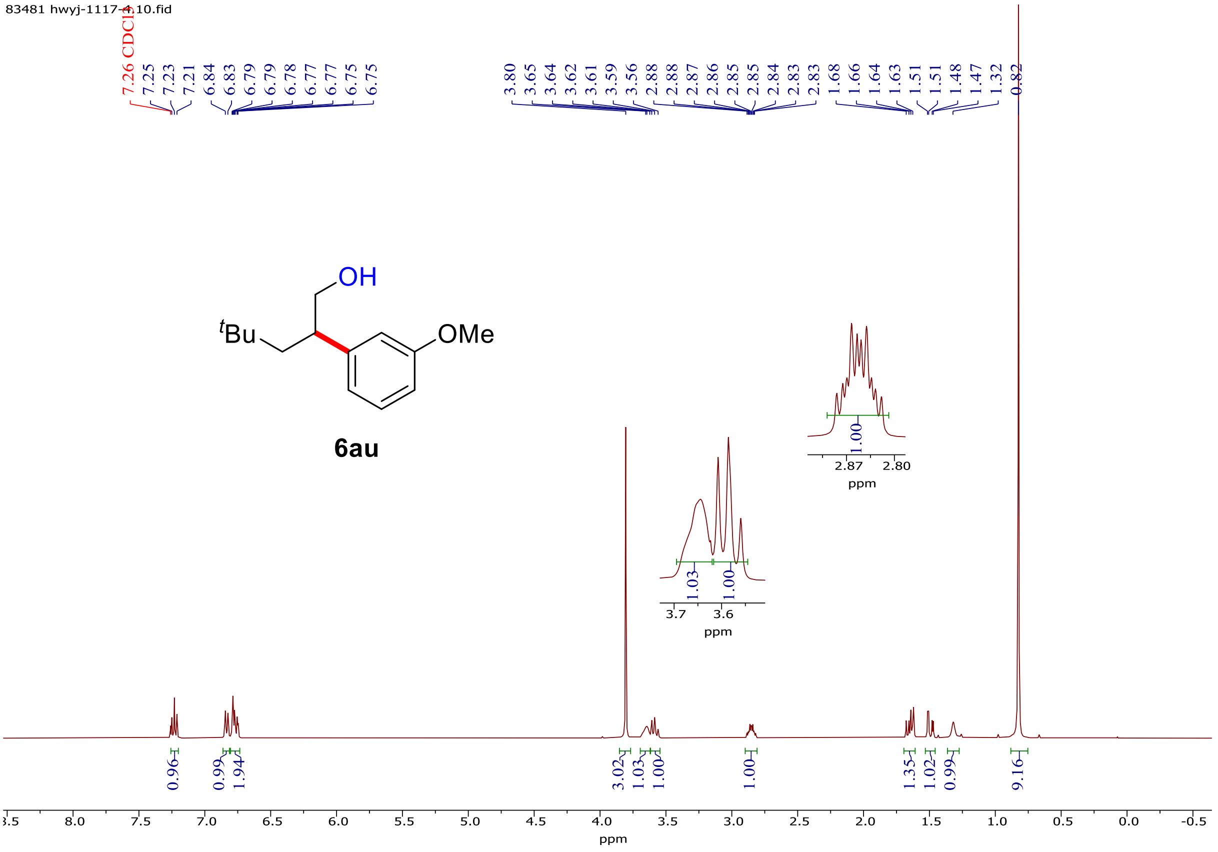

$^{13}$C NMR (101 MHz, $CDCl_3$) of **6au**

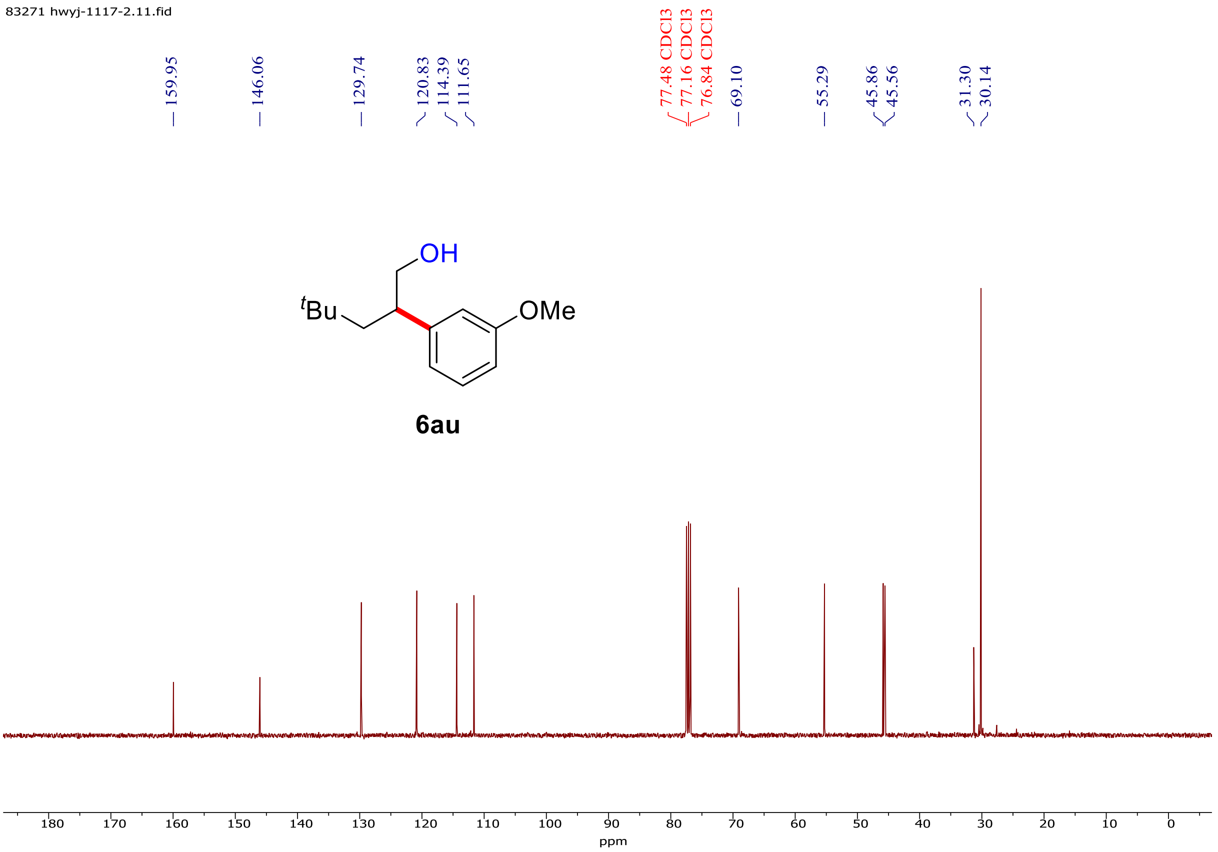


$^{1}$H NMR (400 MHz, $CDCl_3$) of **6av** (see procedure)

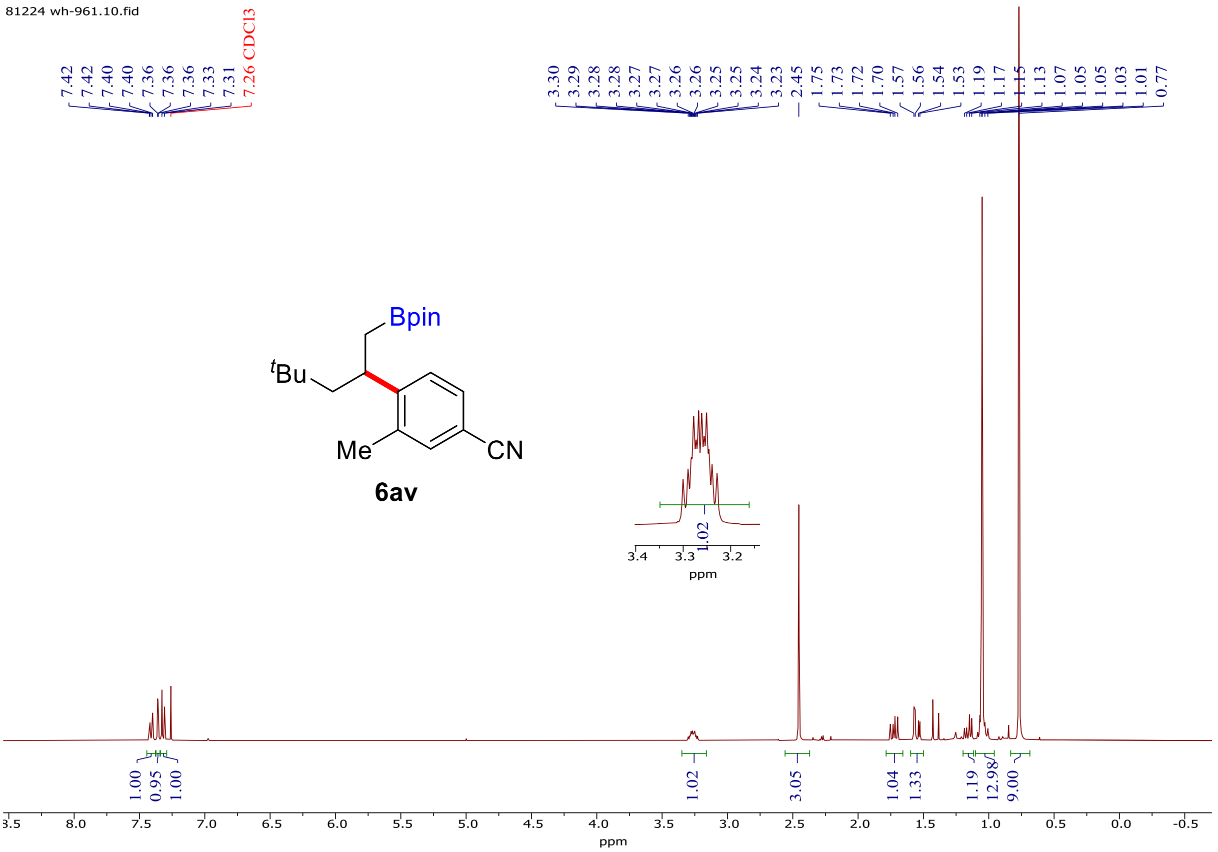

$^{13}$C NMR (101 MHz, $CDCl_3$) of **6av**

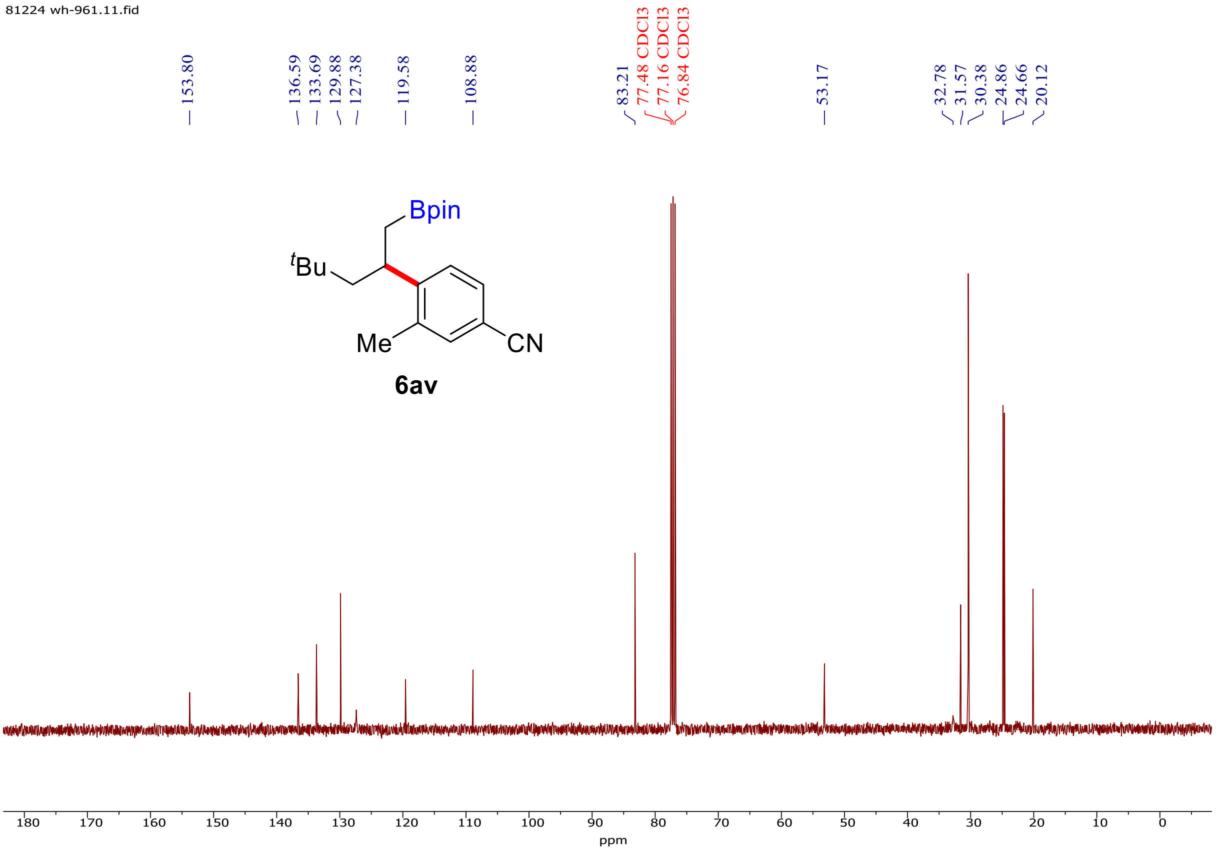

$^{11}$B NMR (128 MHz, $CDCl_3$) of **6av**

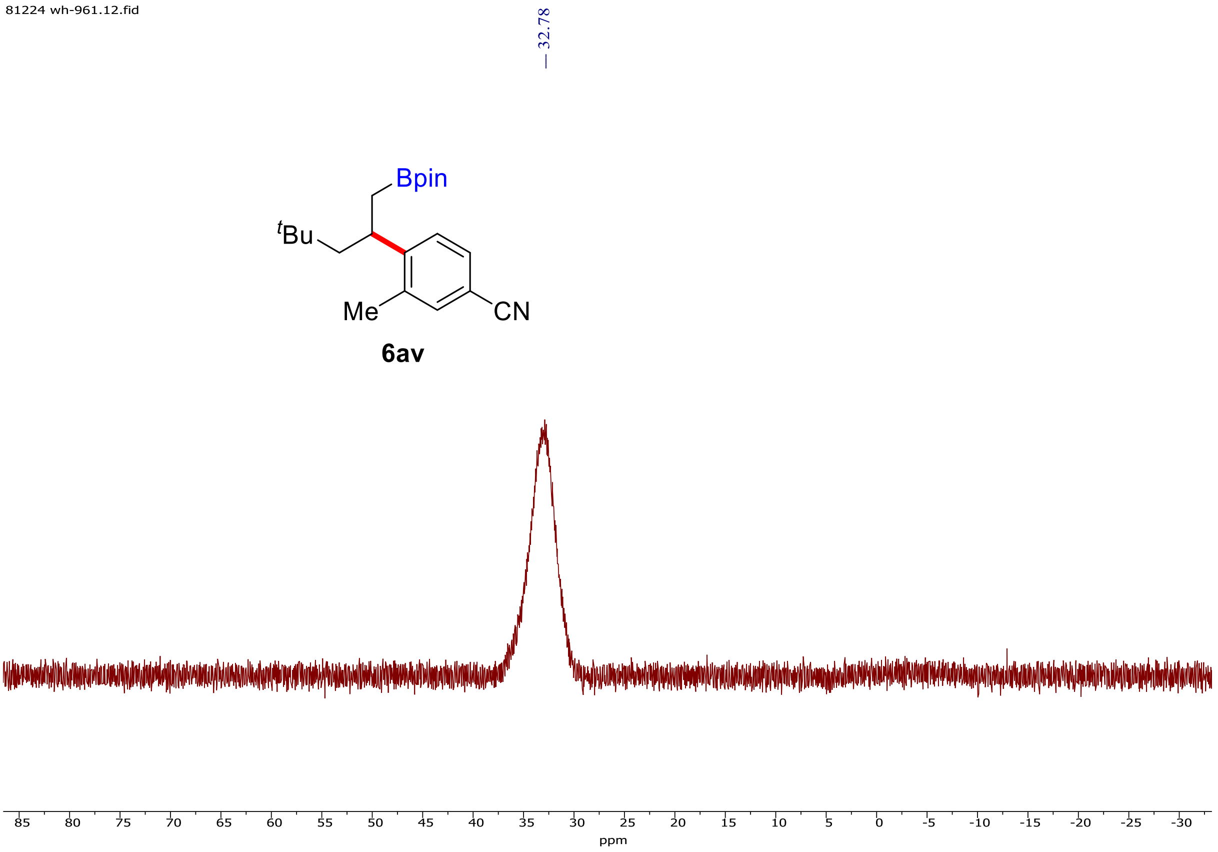

$^1$H NMR (400 MHz, $CDCl_3$) of **6aw** (*see procedure*)

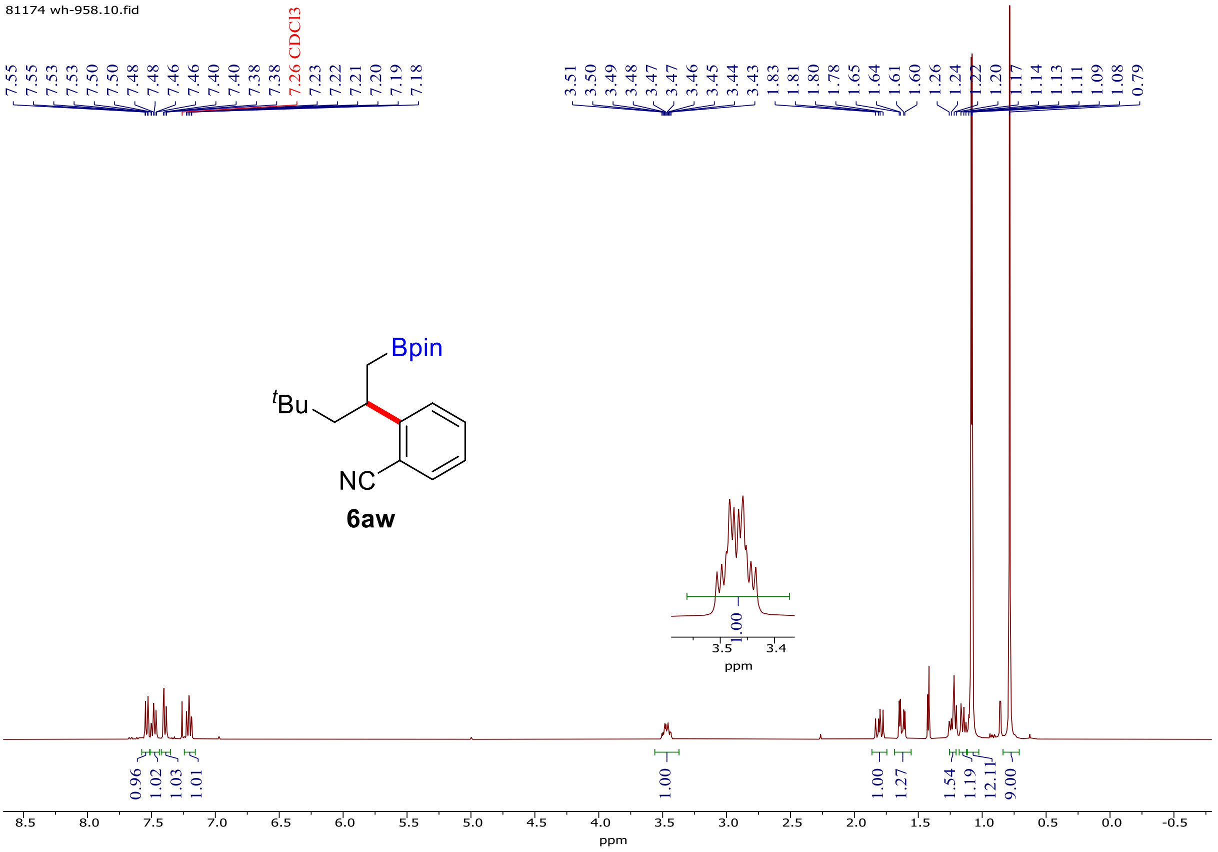

$^{13}C$ NMR (101 MHz, $CDCl_3$) of **6aw**

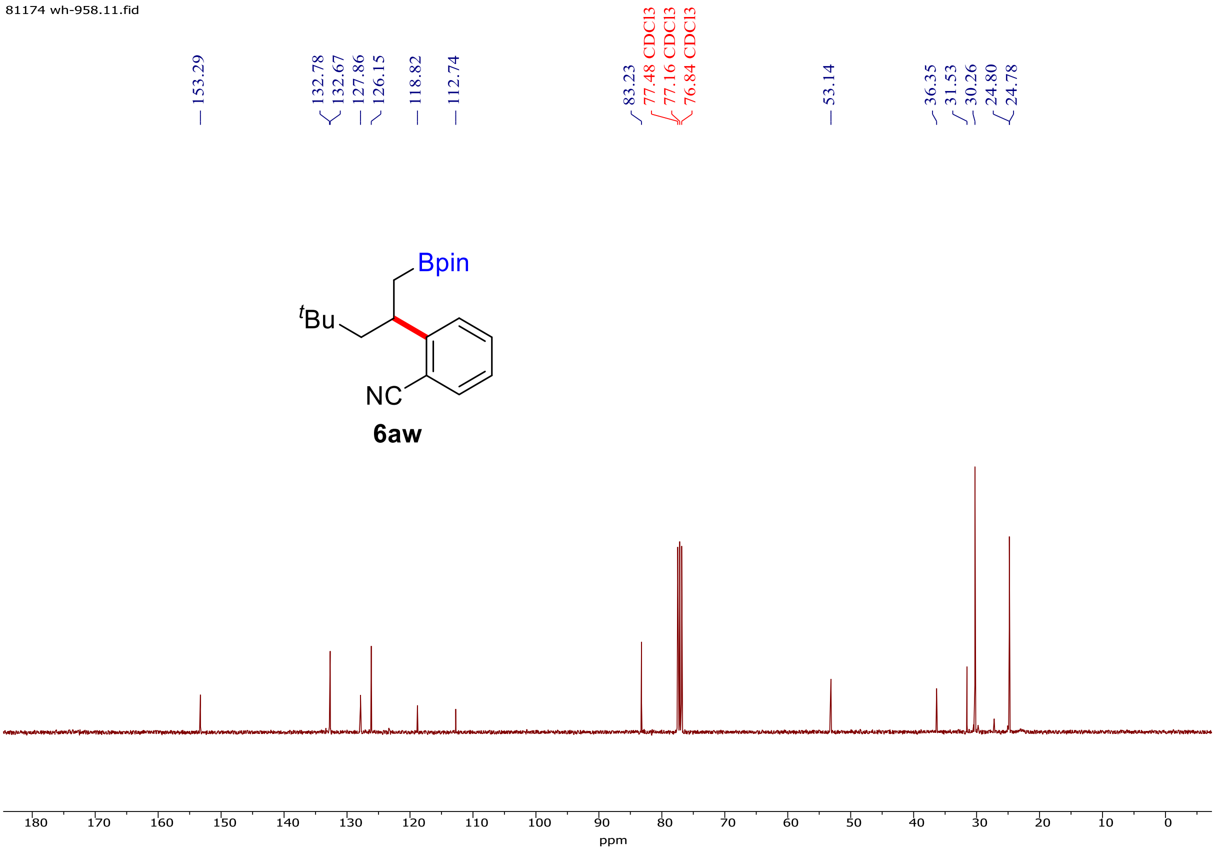

$^{11}$B NMR (128 MHz, $CDCl_3$) of **6aw**

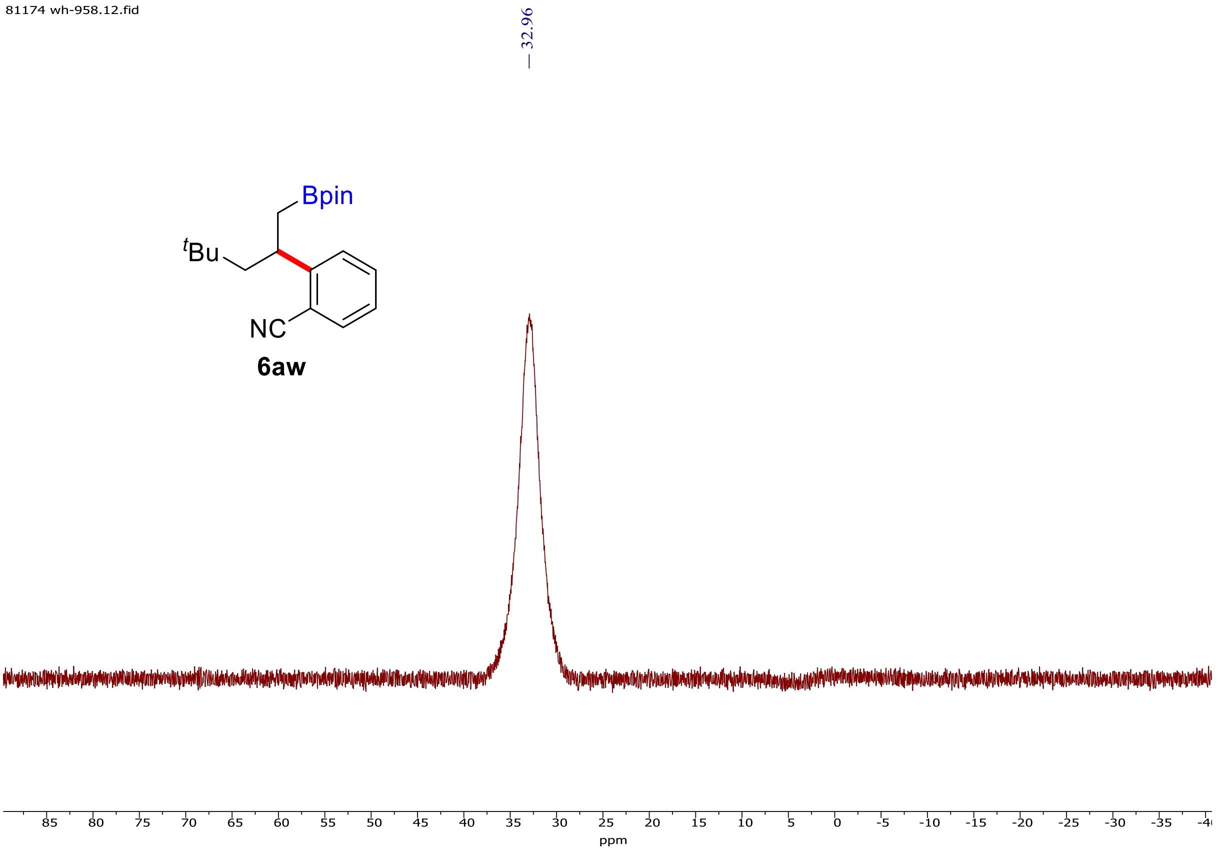

$^{1}$H NMR (400 MHz, $CDCl_3$) of **6ax** (<u>*see procedure*</u>)

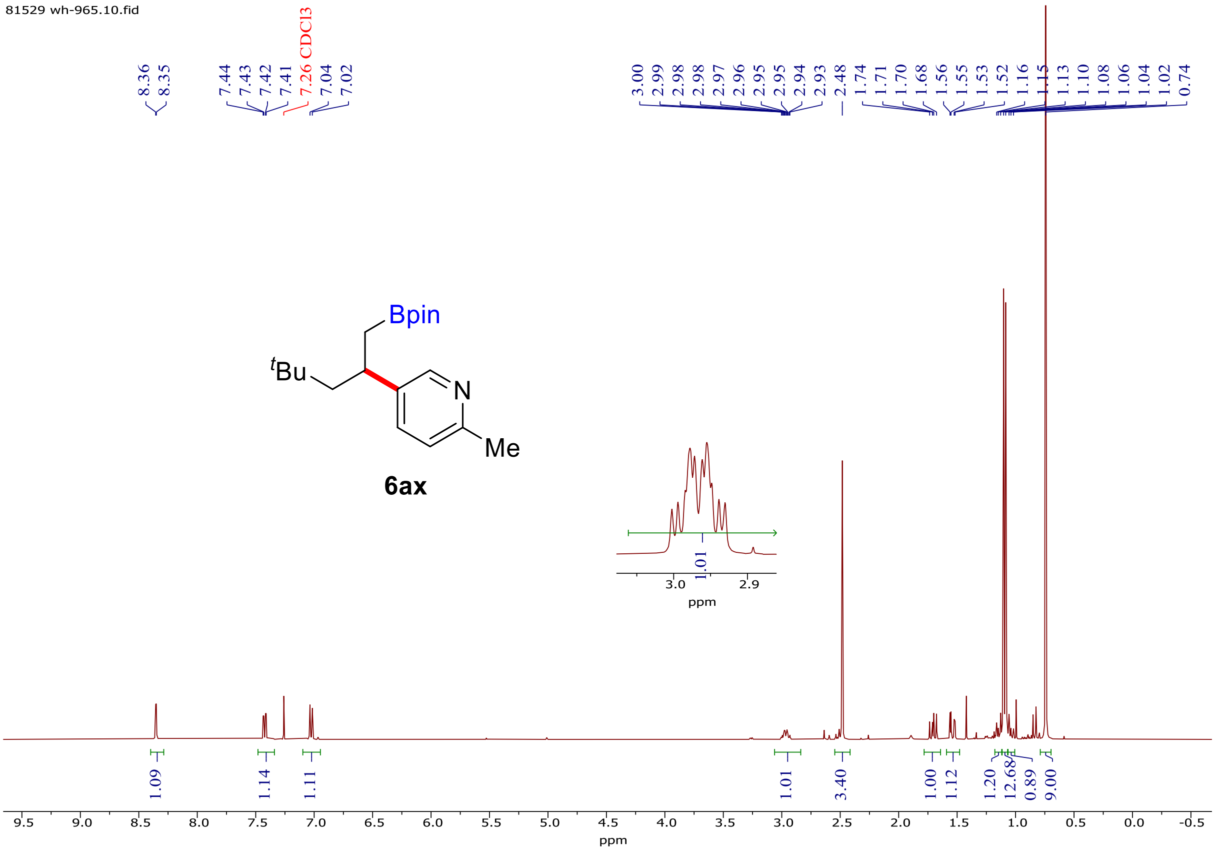

$^{13}$C NMR (101 MHz, $CDCl_3$) of **6ax**

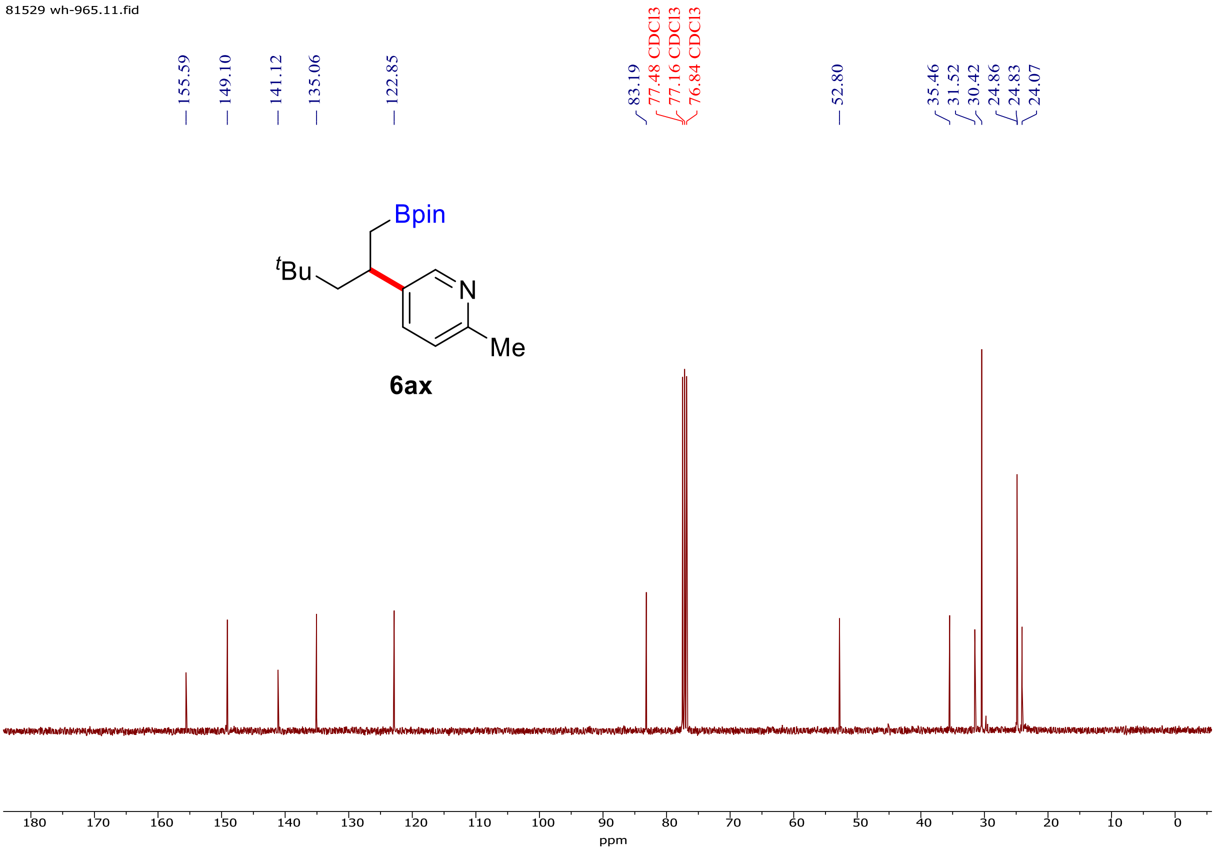

$^{11}B$ NMR (128 MHz, $CDCl_3$) of **6ax**

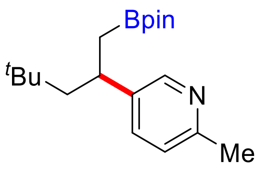


**6ax**

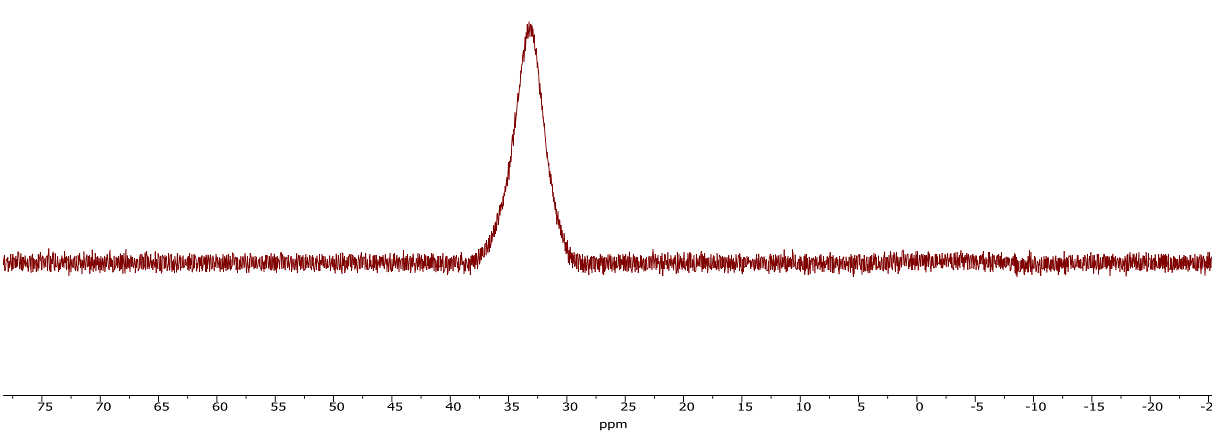

$^1$H NMR (400 MHz, $CDCl_3$) of **6ay** (*see procedure*)

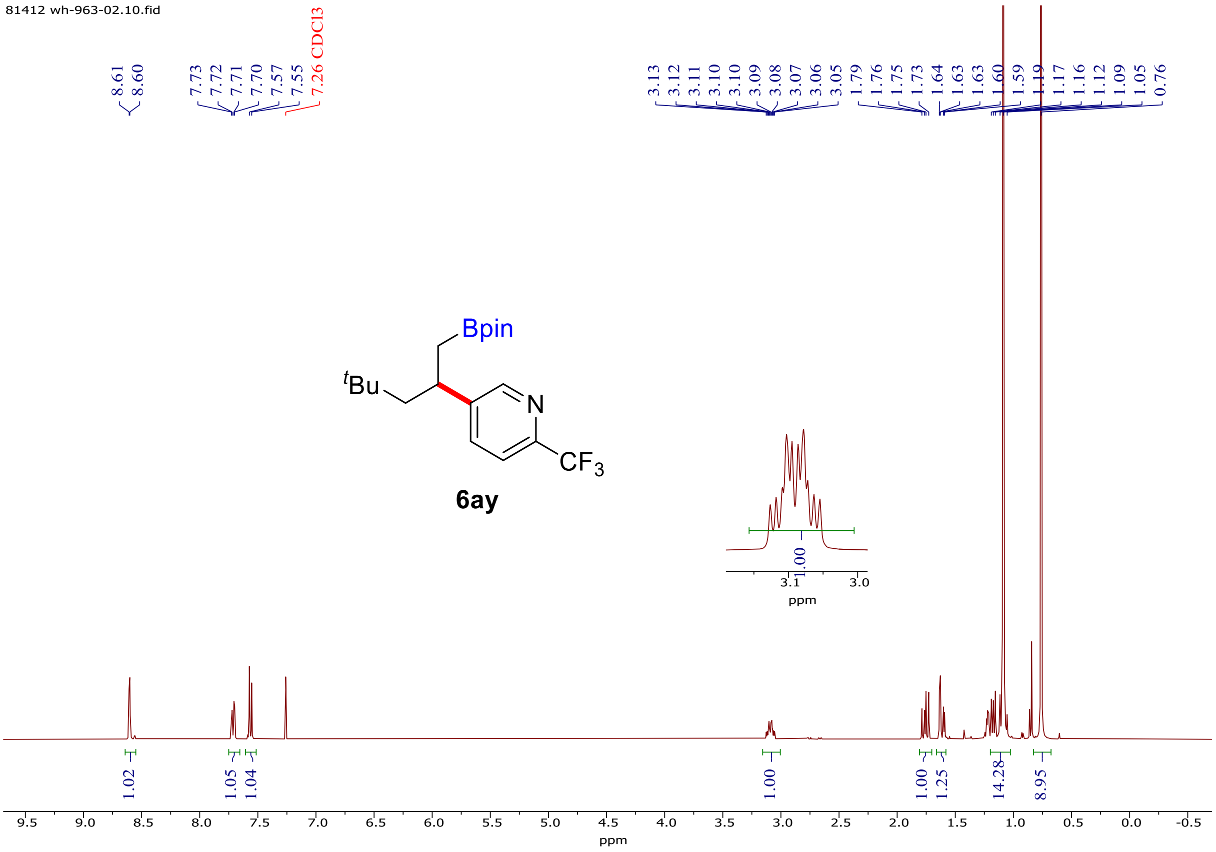

$^{13}C$ NMR (101 MHz, $CDCl_3$) of **6ay**

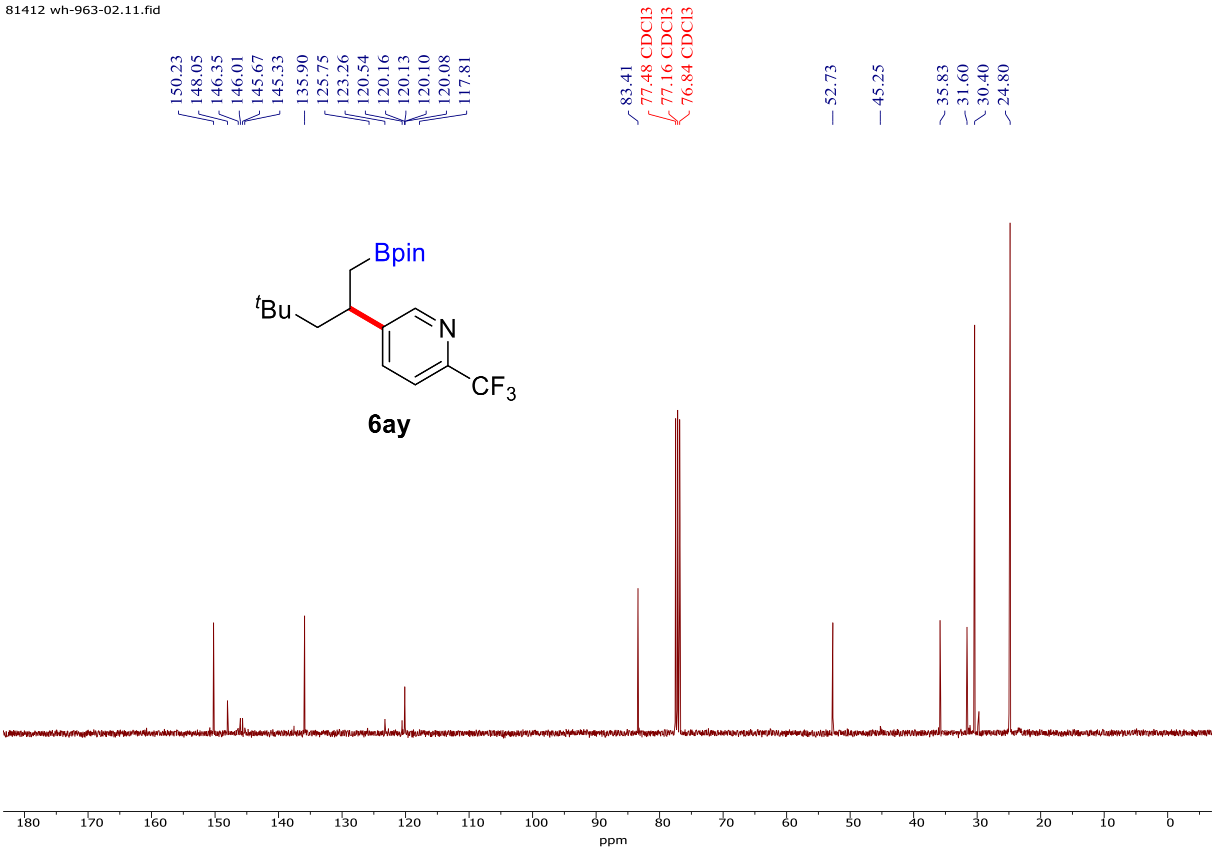

$^{19}F$ NMR (376 MHz, $CDCl_3$) of **6ay**

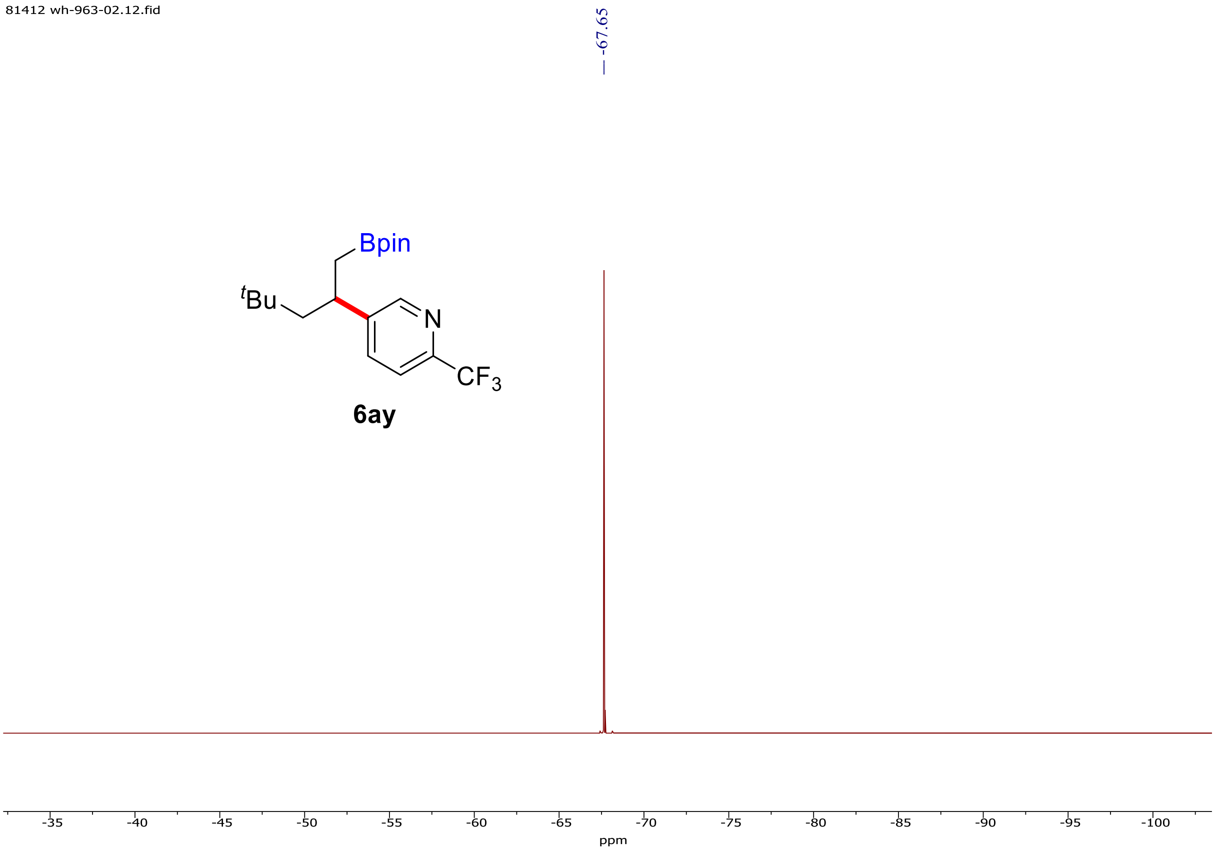

$^{11}$B NMR (128 MHz, $CDCl_3$) of **6ay**

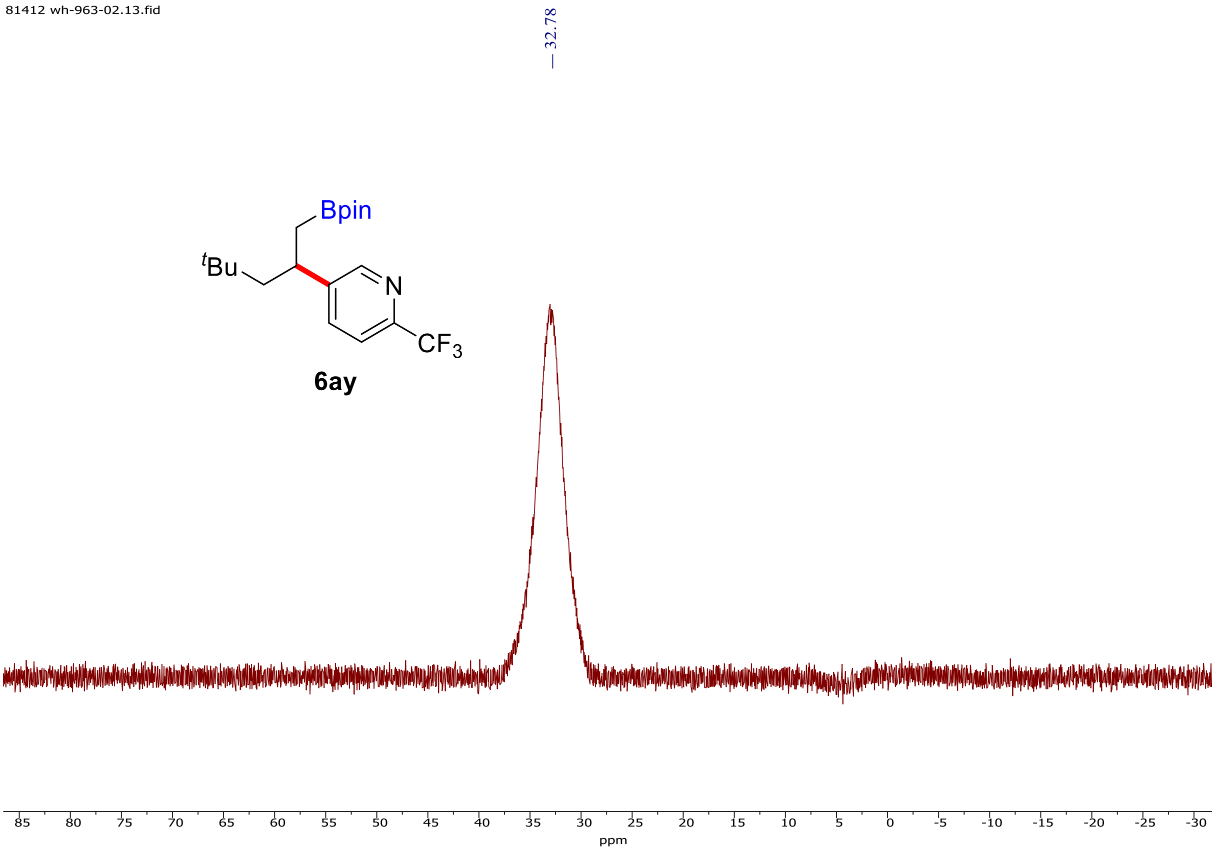

$^1$H NMR (400 MHz, $CDCl_3$) of **6az** (*see procedure*)

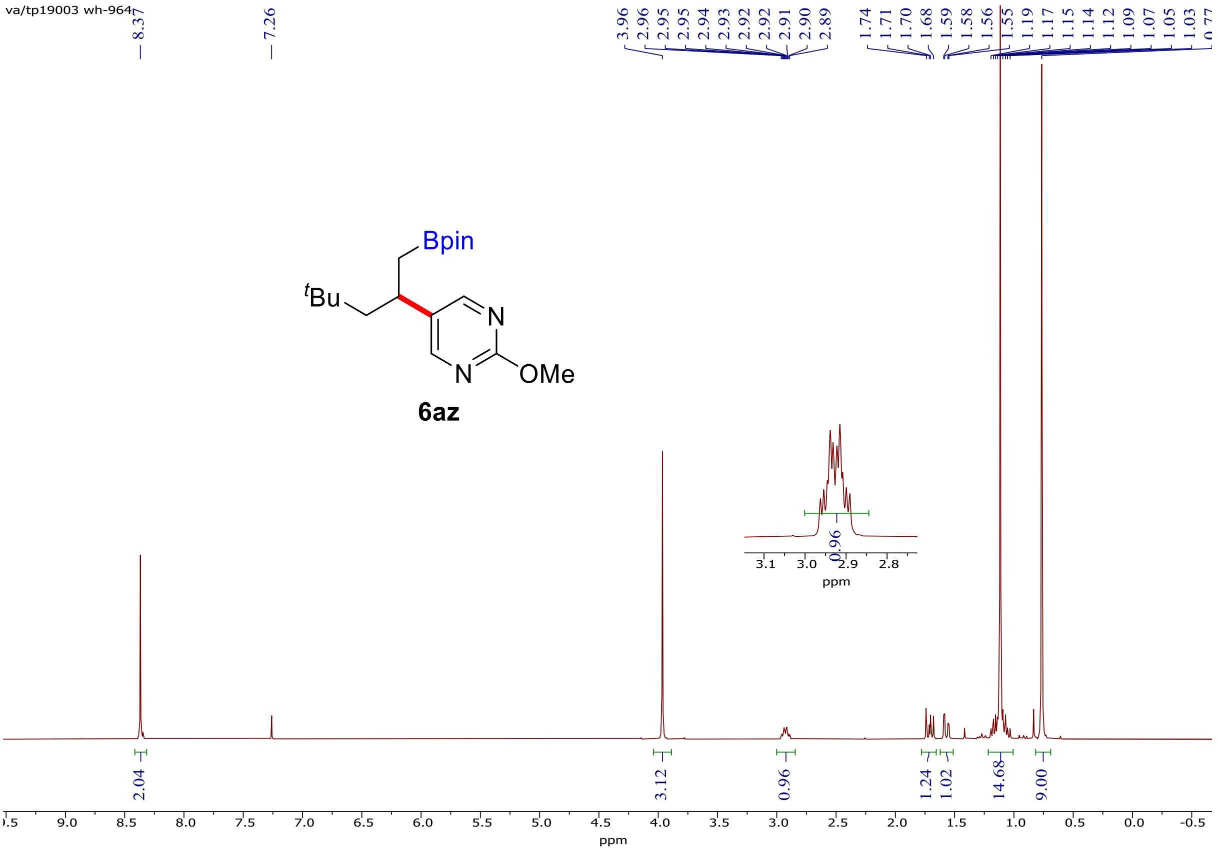

$^{13}$C NMR (101 MHz, $CDCl_3$) of **6az**

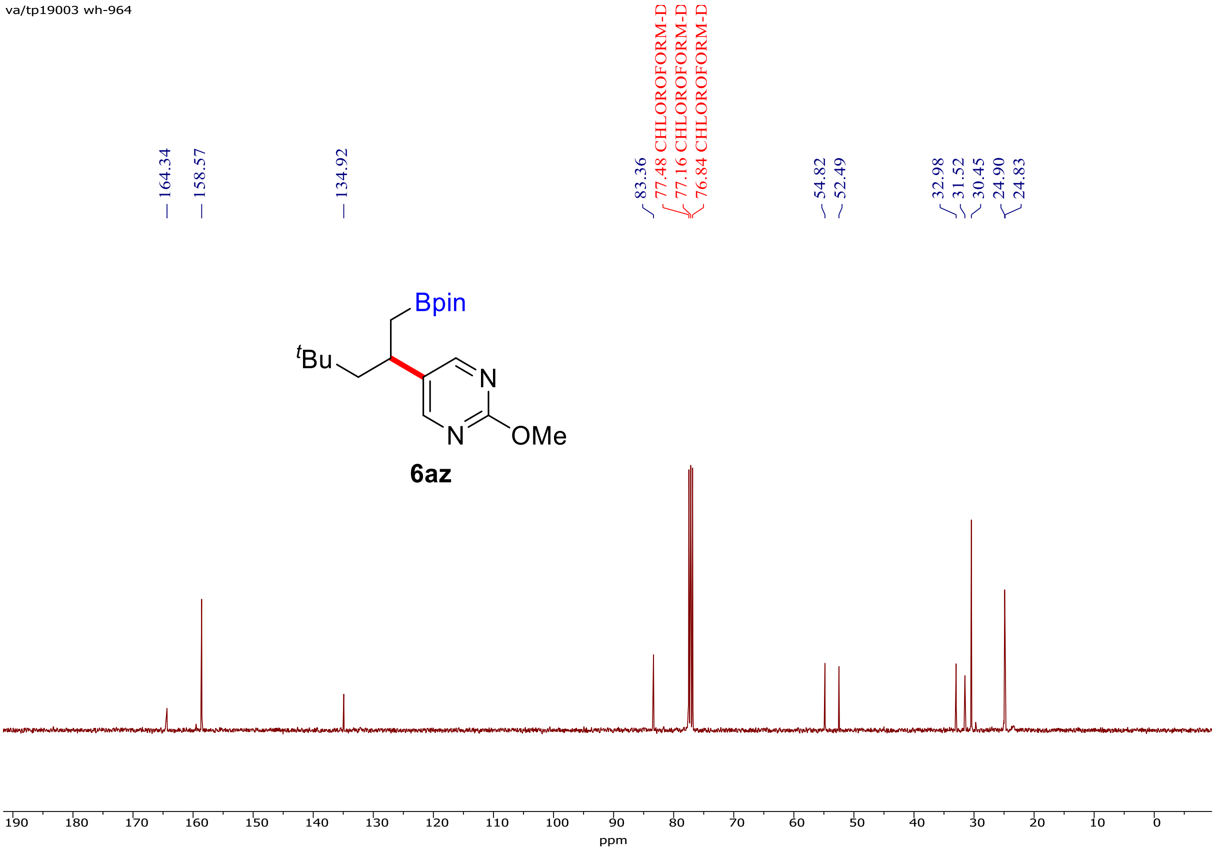

$^{11}$B NMR (128 MHz, $CDCl_3$) of **6az**

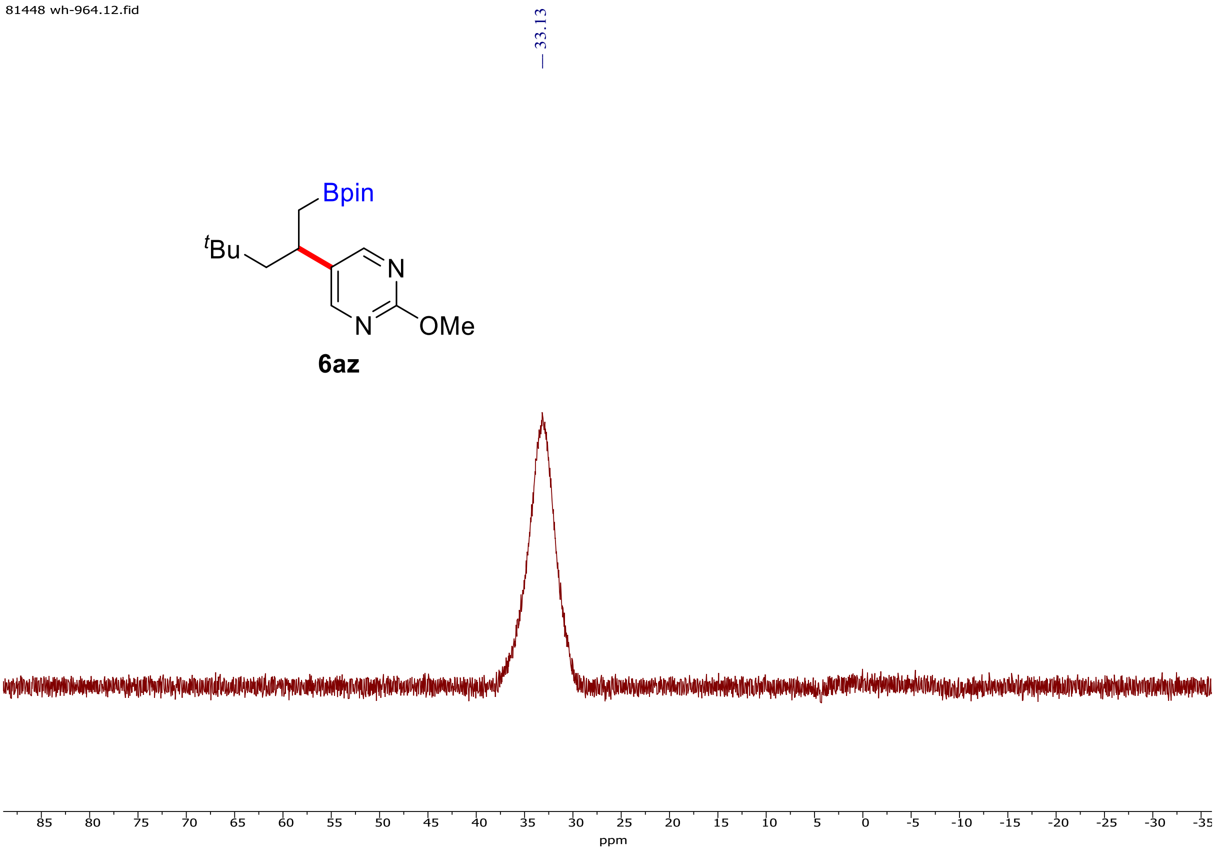

$^{1}$H NMR (400 MHz, $CDCl_3$) of **6aaa** (see procedure)

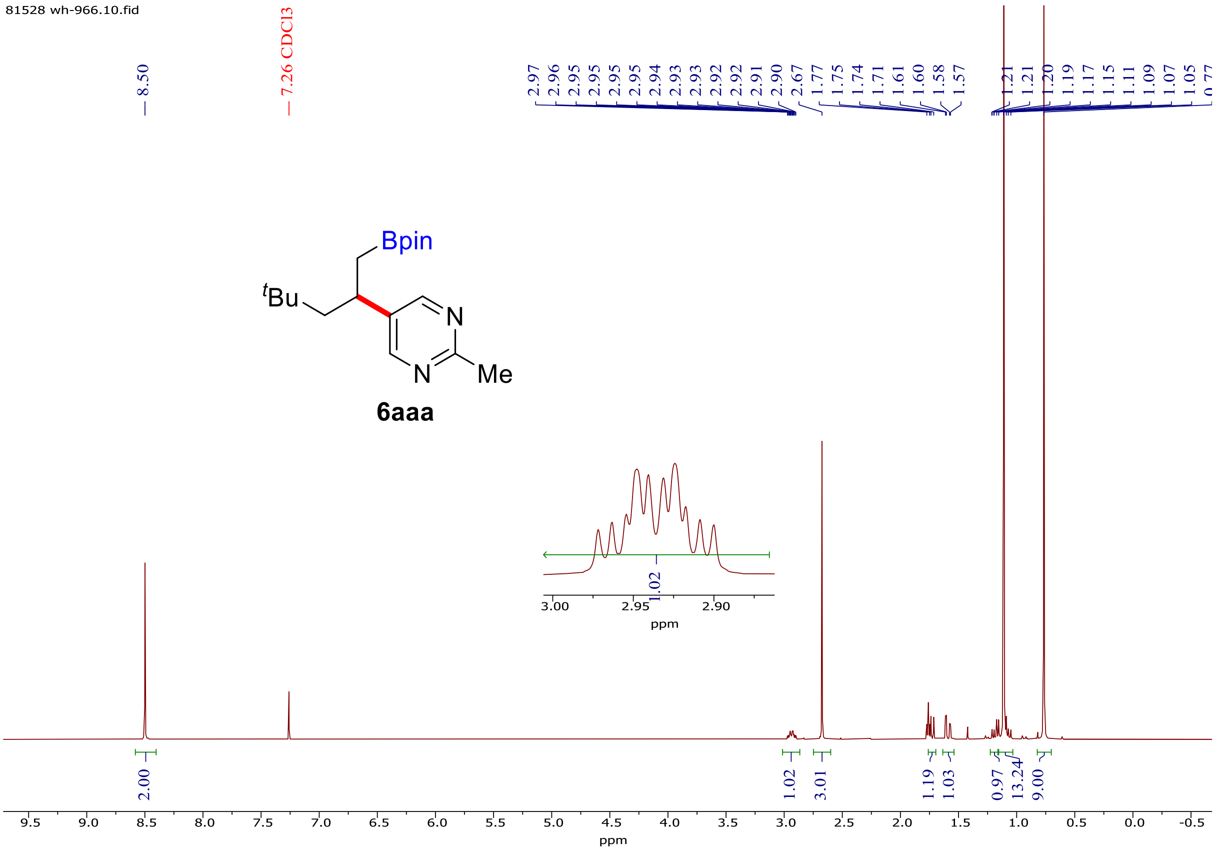

$^{13}$C NMR (101 MHz, $CDCl_3$) of **6aa'**

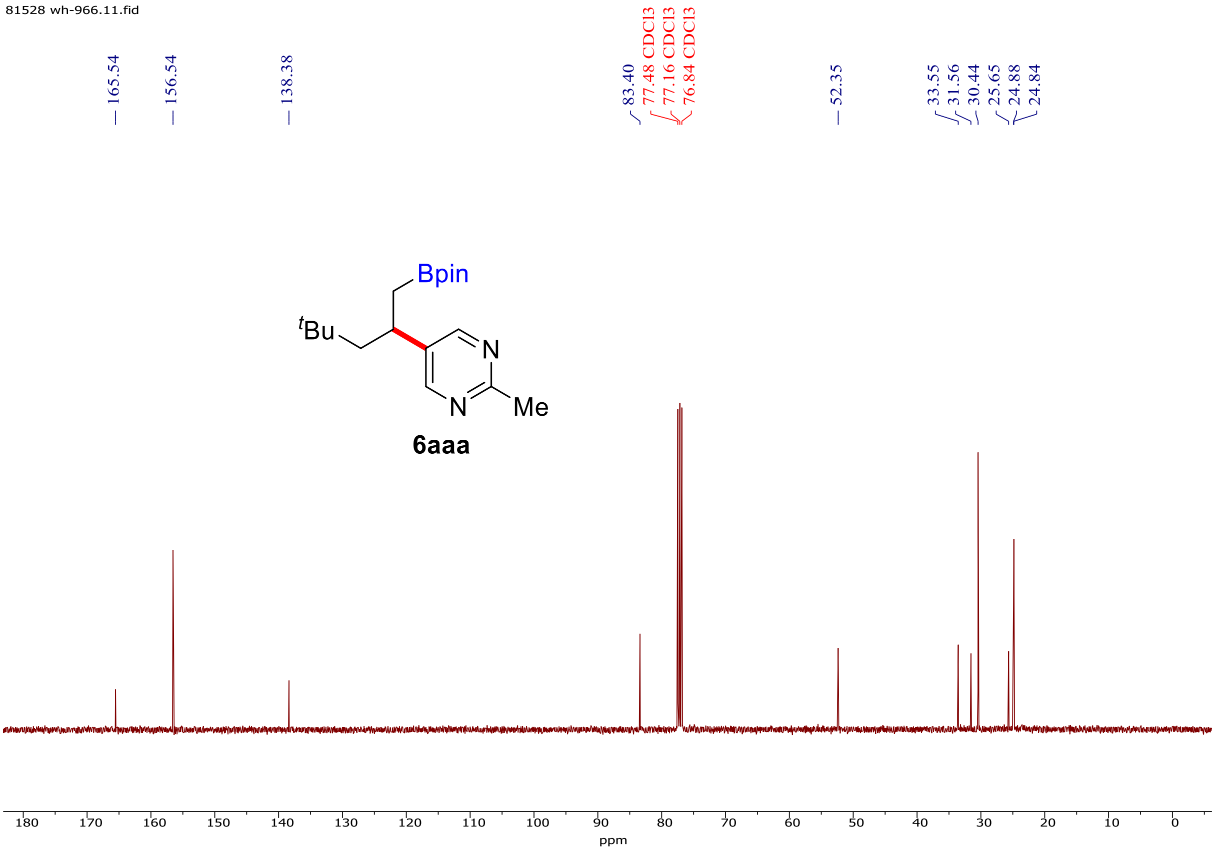

$^{11}$B NMR (128 MHz, $CDCl_3$) of **6aaa**

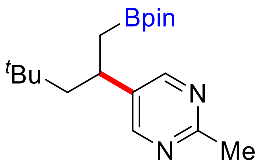


**6aaa**

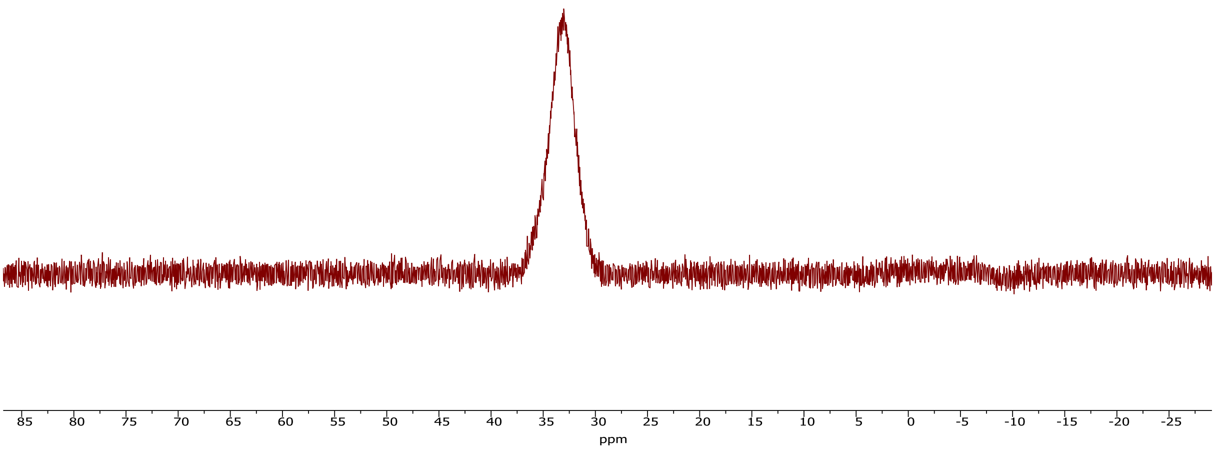

$^{1}$H NMR (400 MHz, $CDCl_3$) of **6aab** (*see procedure*)

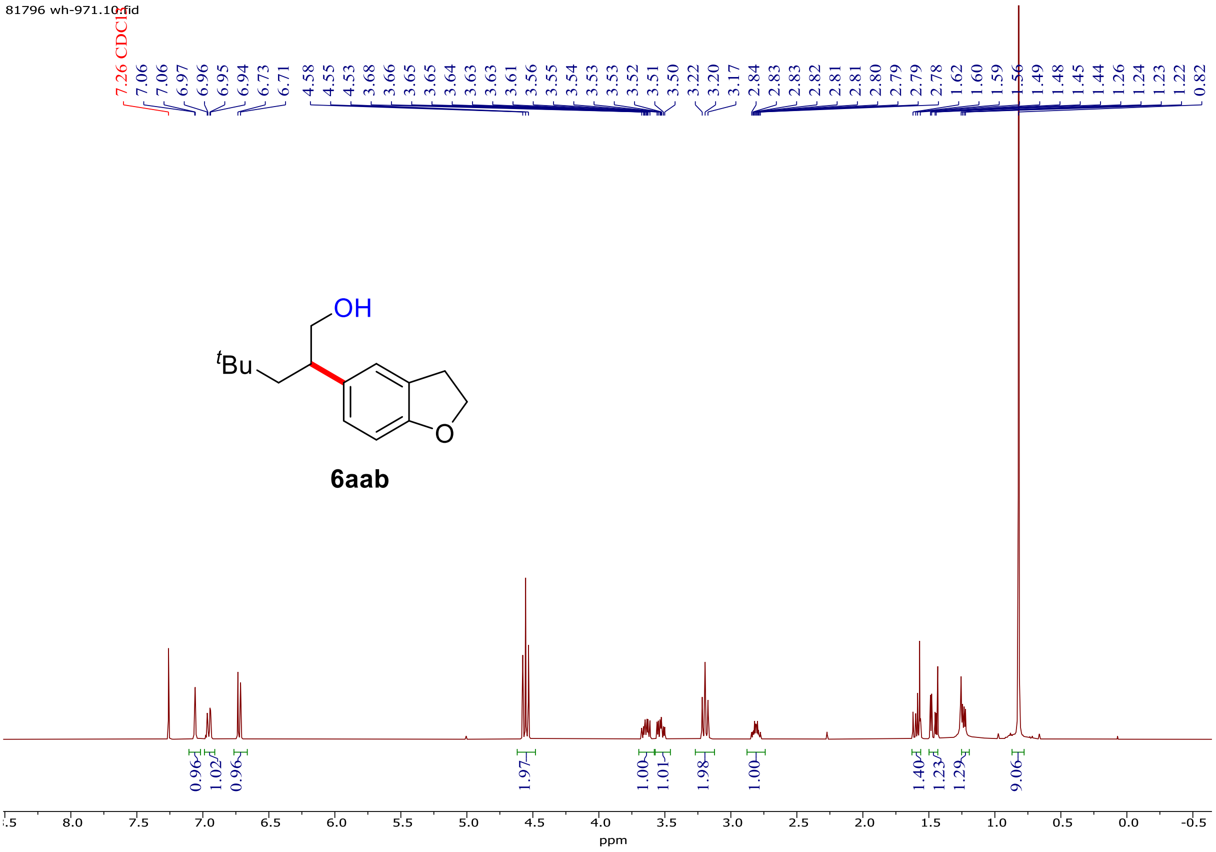

$^{13}$C NMR (101 MHz, $CDCl_3$) of **6aab**

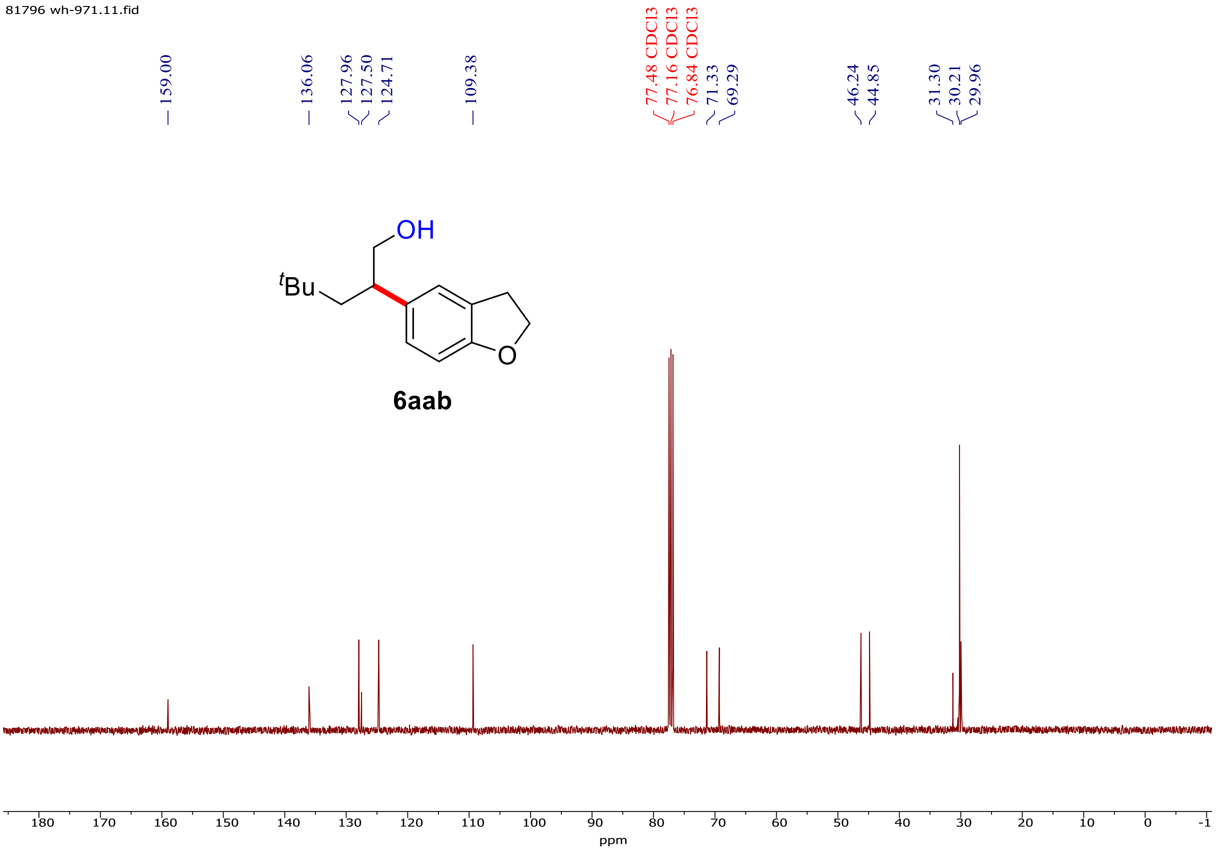

$^{1}$H NMR (400 MHz, $CDCl_3$) of **6aac** (*see procedure*)

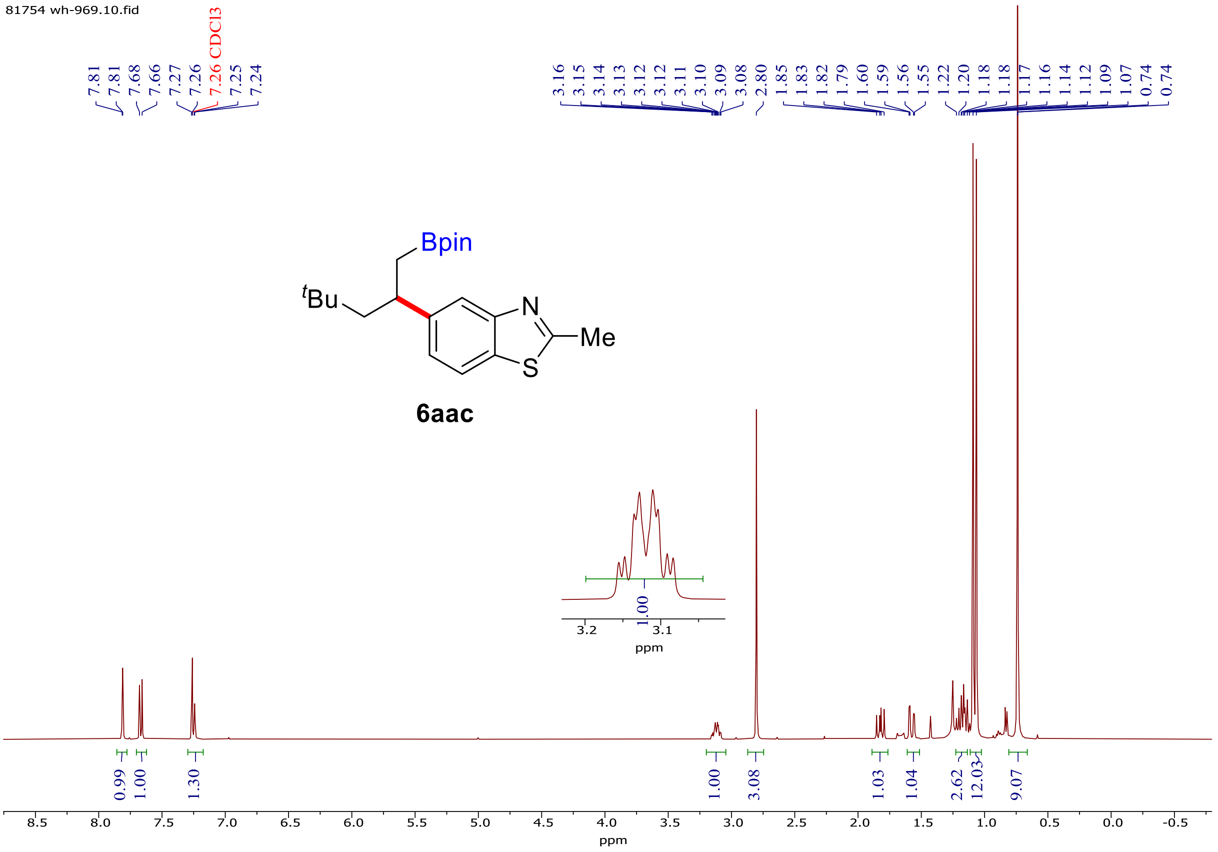

$^{13}$C NMR (101 MHz, $CDCl_3$) of **6aac**

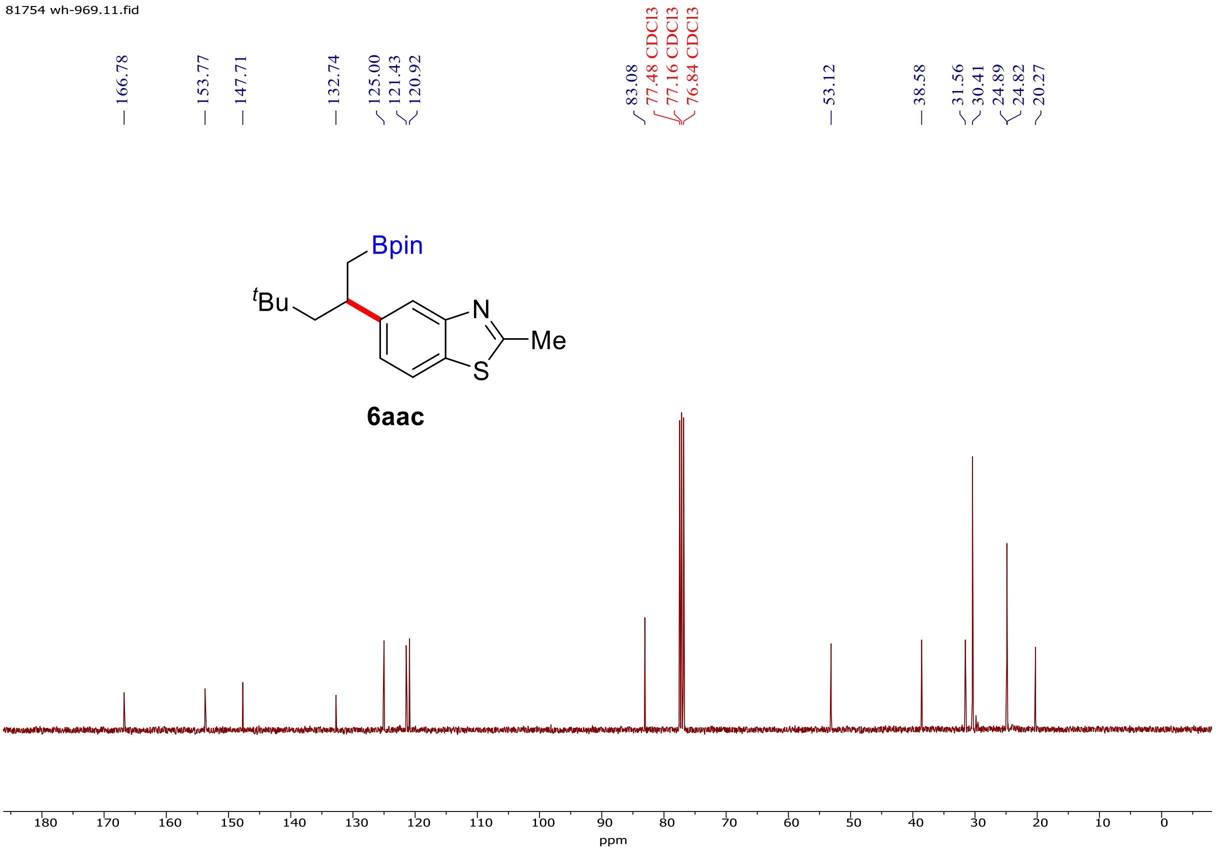

$^{11}$B NMR (128 MHz, $CDCl_3$) of **6aac**

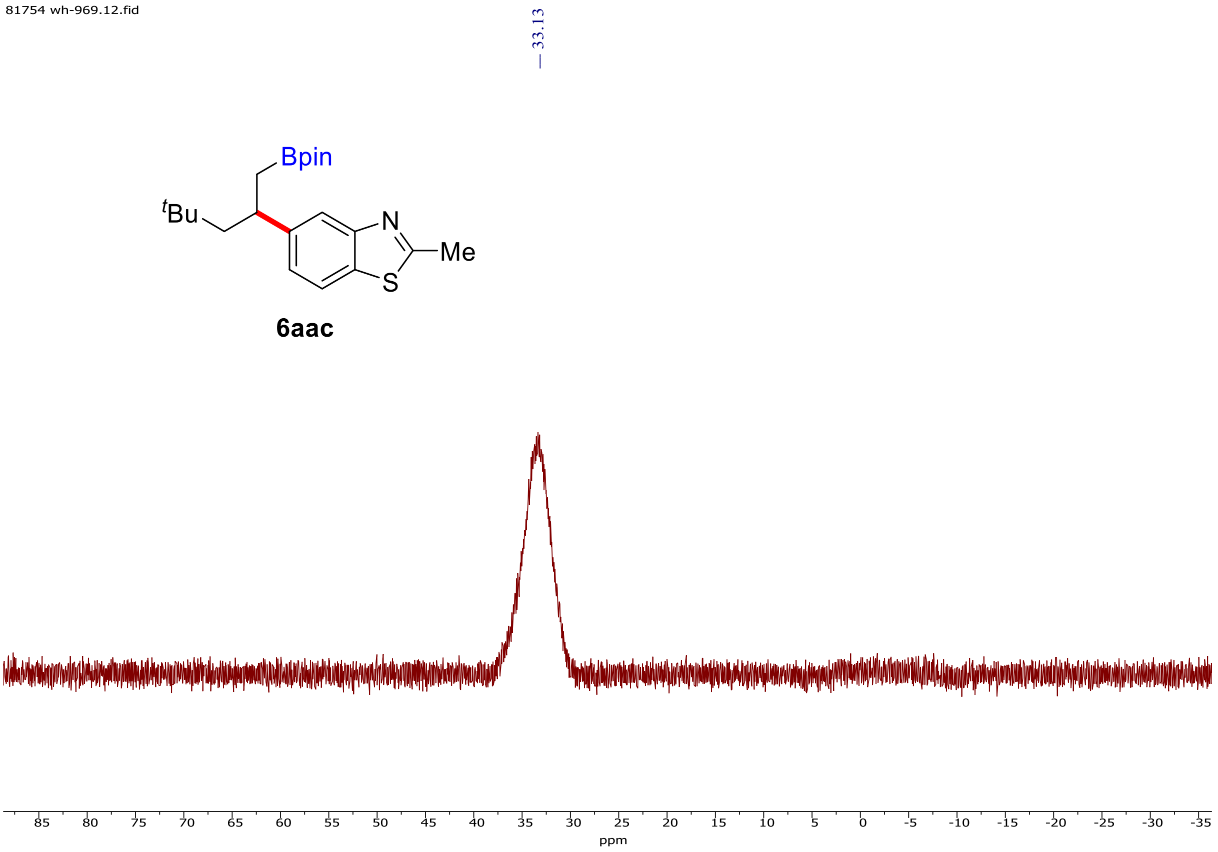


$^{1}$H NMR (400 MHz, $CDCl_3$) of **6aad** (*see procedure*)

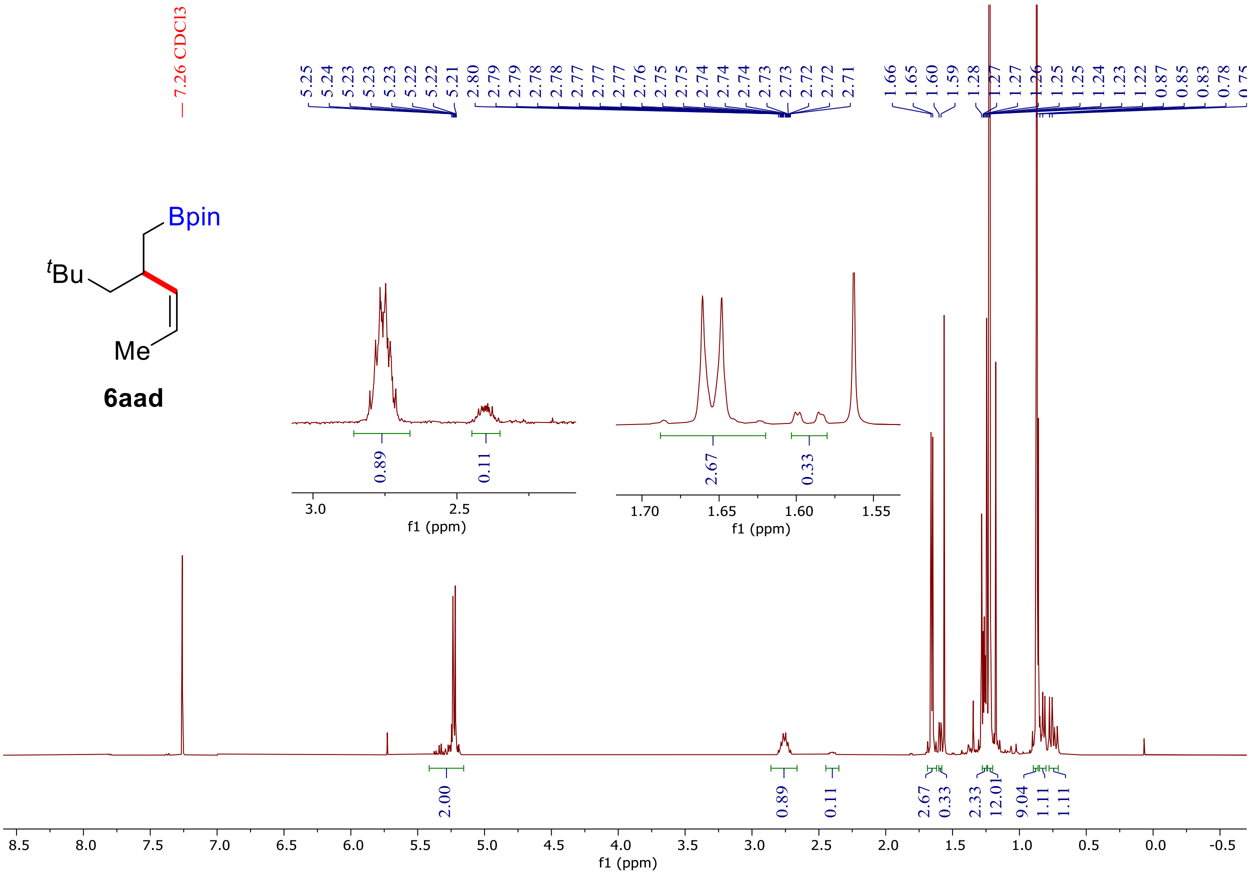

$^{13}$C NMR (400 MHz, $CDCl_3$) of **6aad**

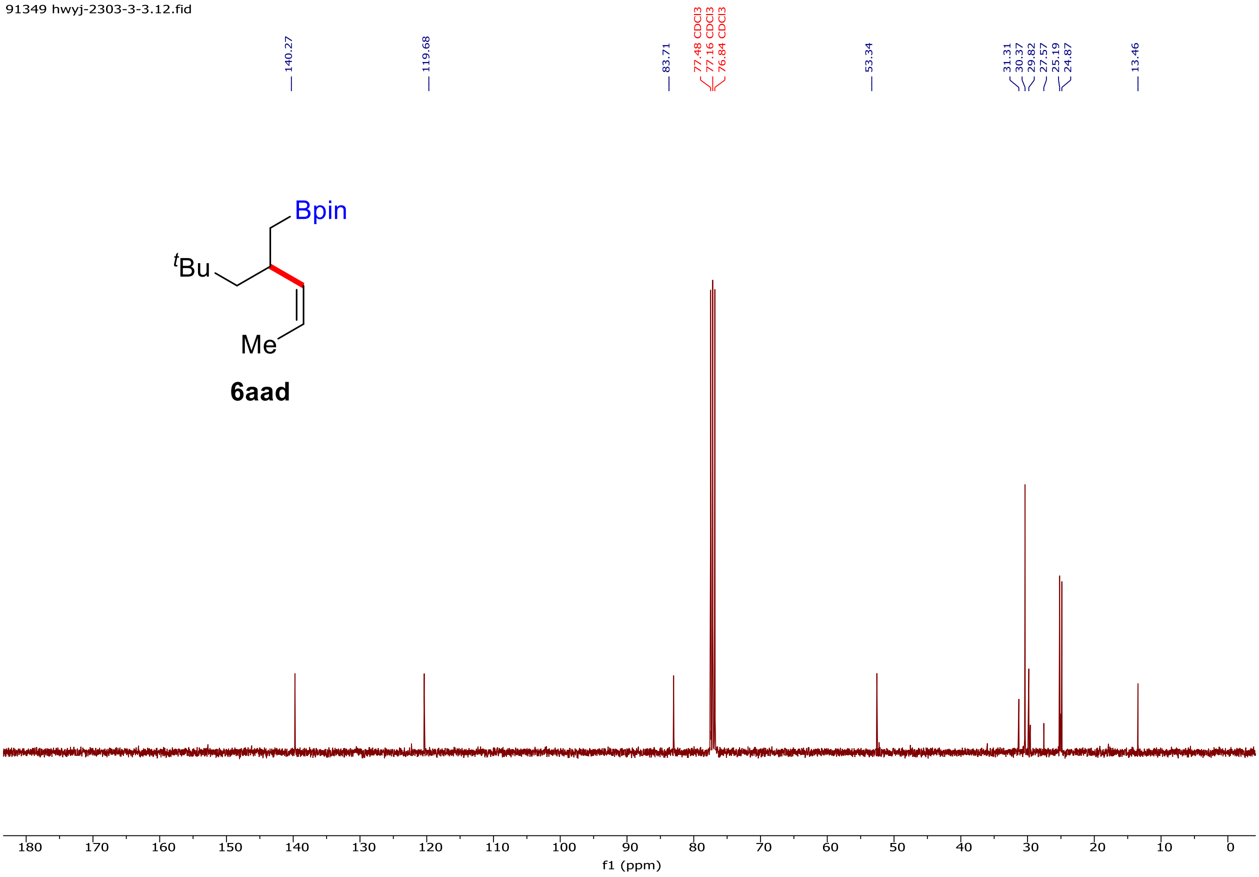


$^{11}$B NMR (400 MHz, $CDCl_3$) of **6aad**

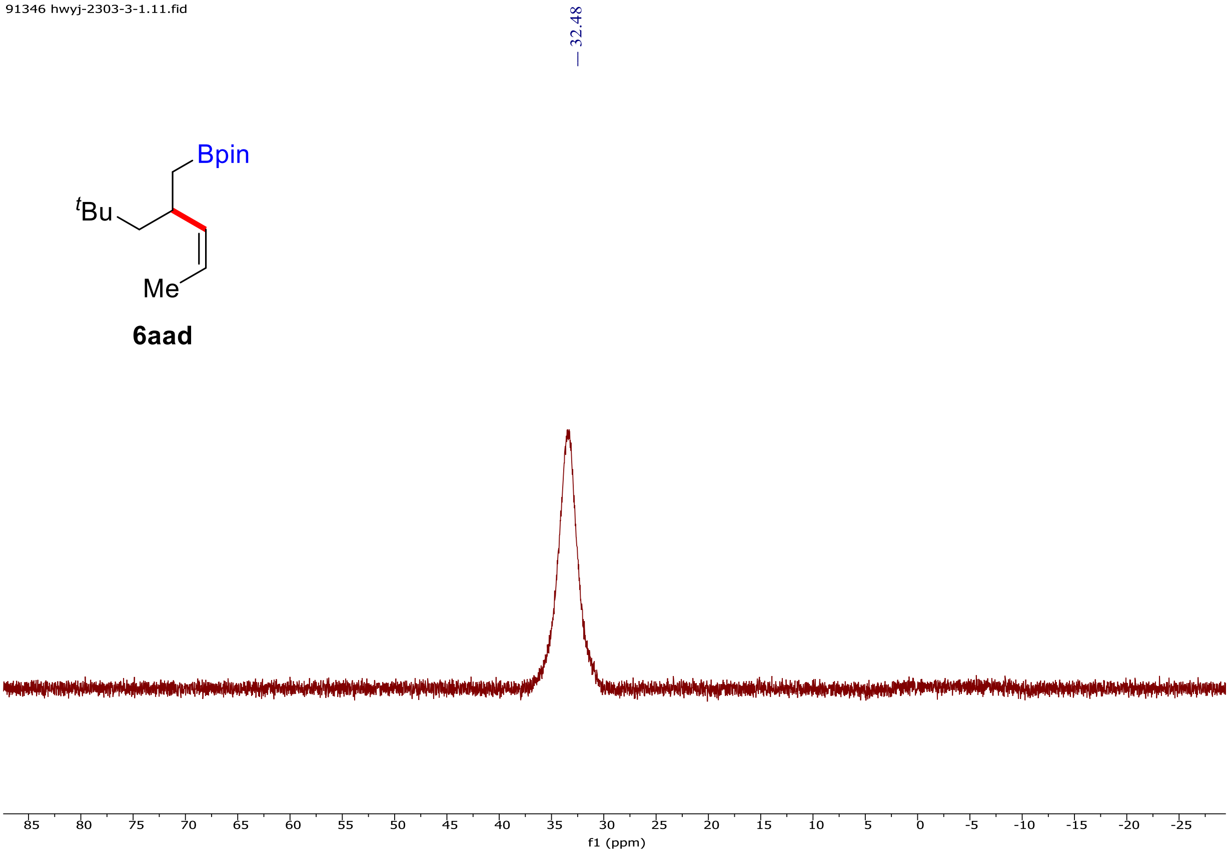

$^{1}$H NMR (400 MHz, $CDCl_3$) of **6ba** (*see procedure*)

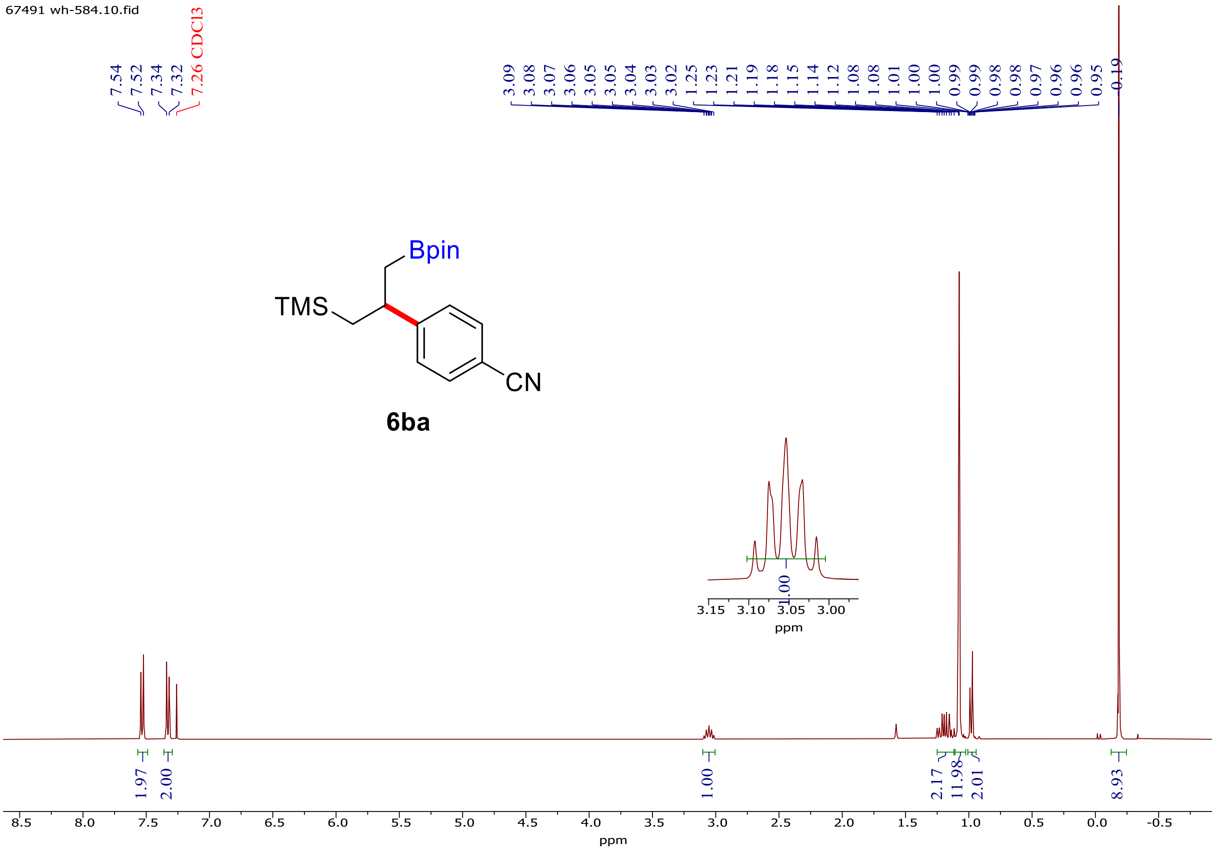

$^{13}C$ NMR (101 MHz, $CDCl_3$) of **6ba**

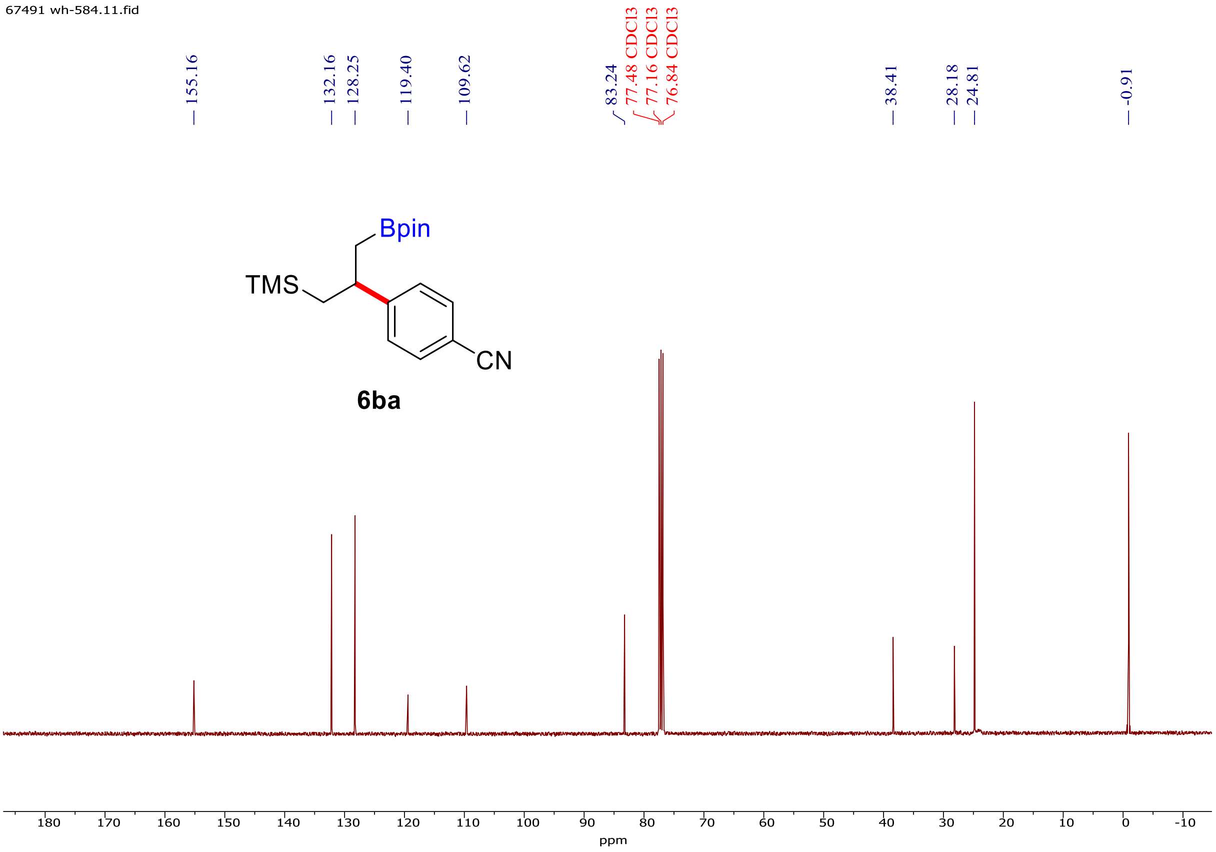

$^{11}$B NMR (128 MHz, $CDCl_3$) of **6ba**

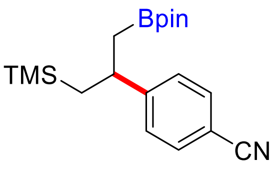


**6ba**

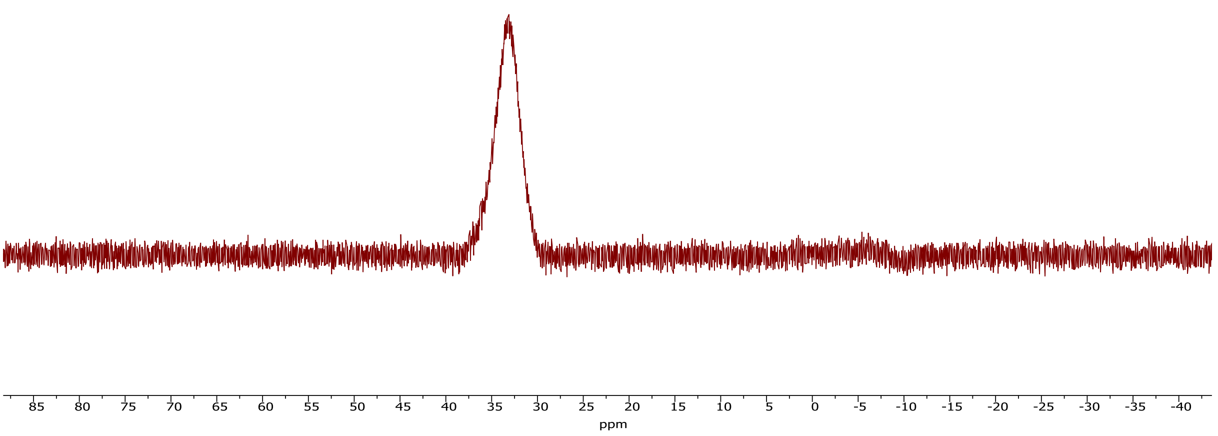

$^{1}$H NMR (400 MHz, $CDCl_3$) of **6ca** (*see procedure*)

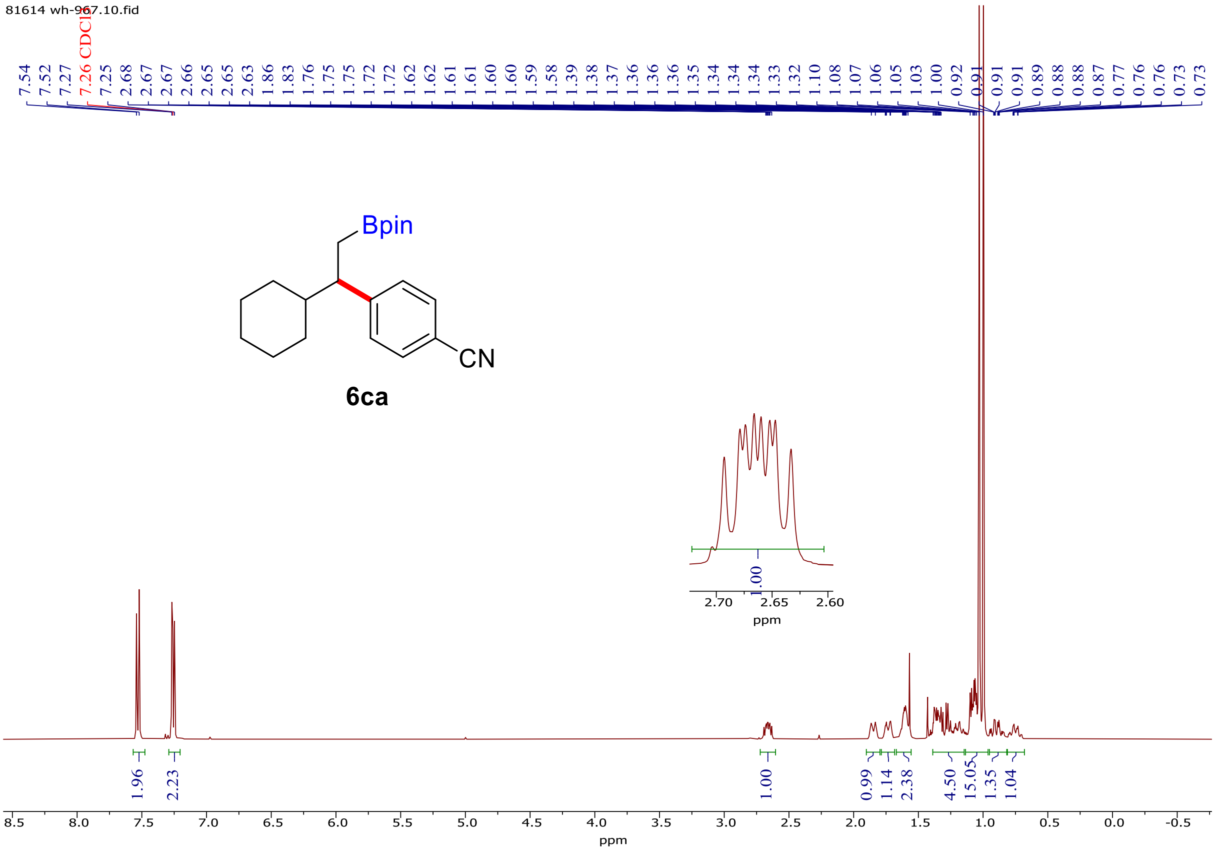

$^{13}$C NMR (101 MHz, $CDCl_3$) of **6ca**

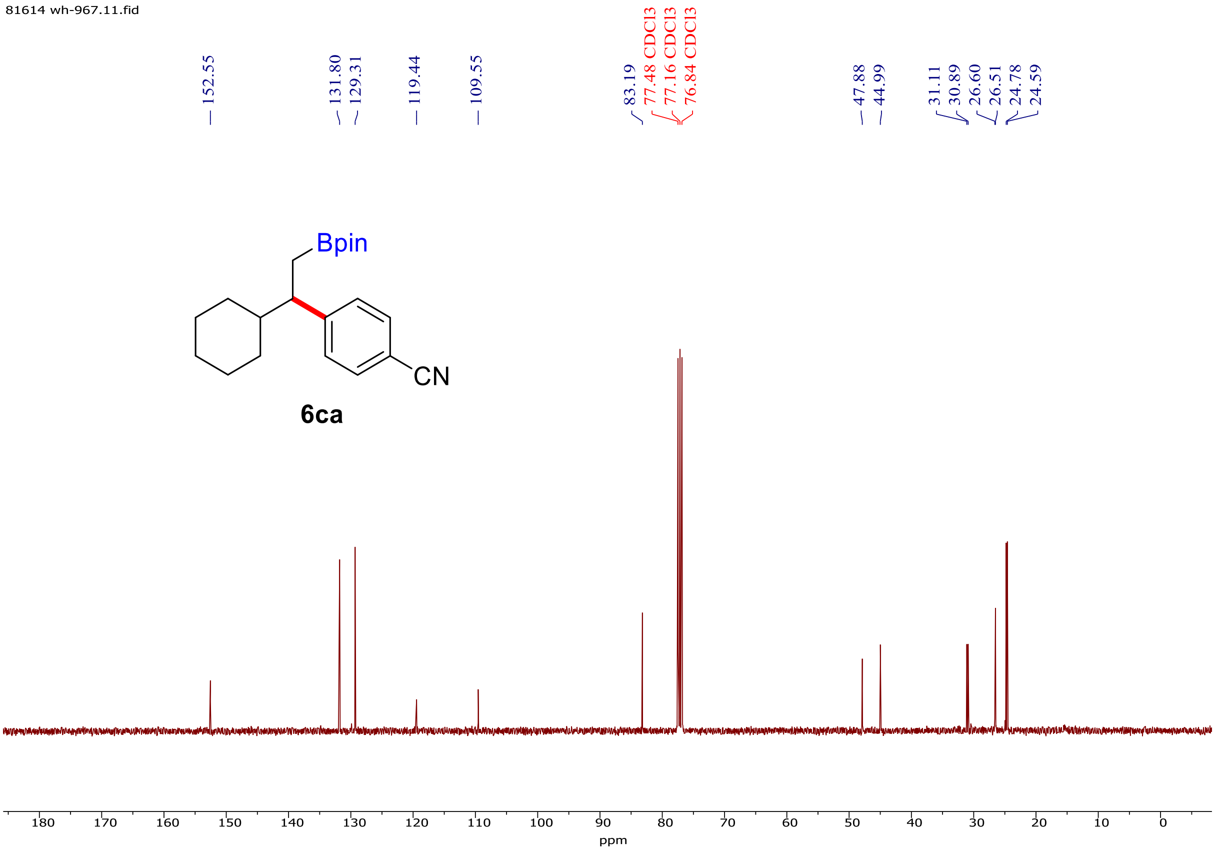

$^{11}$B NMR (128 MHz, $CDCl_3$) of **6ca**

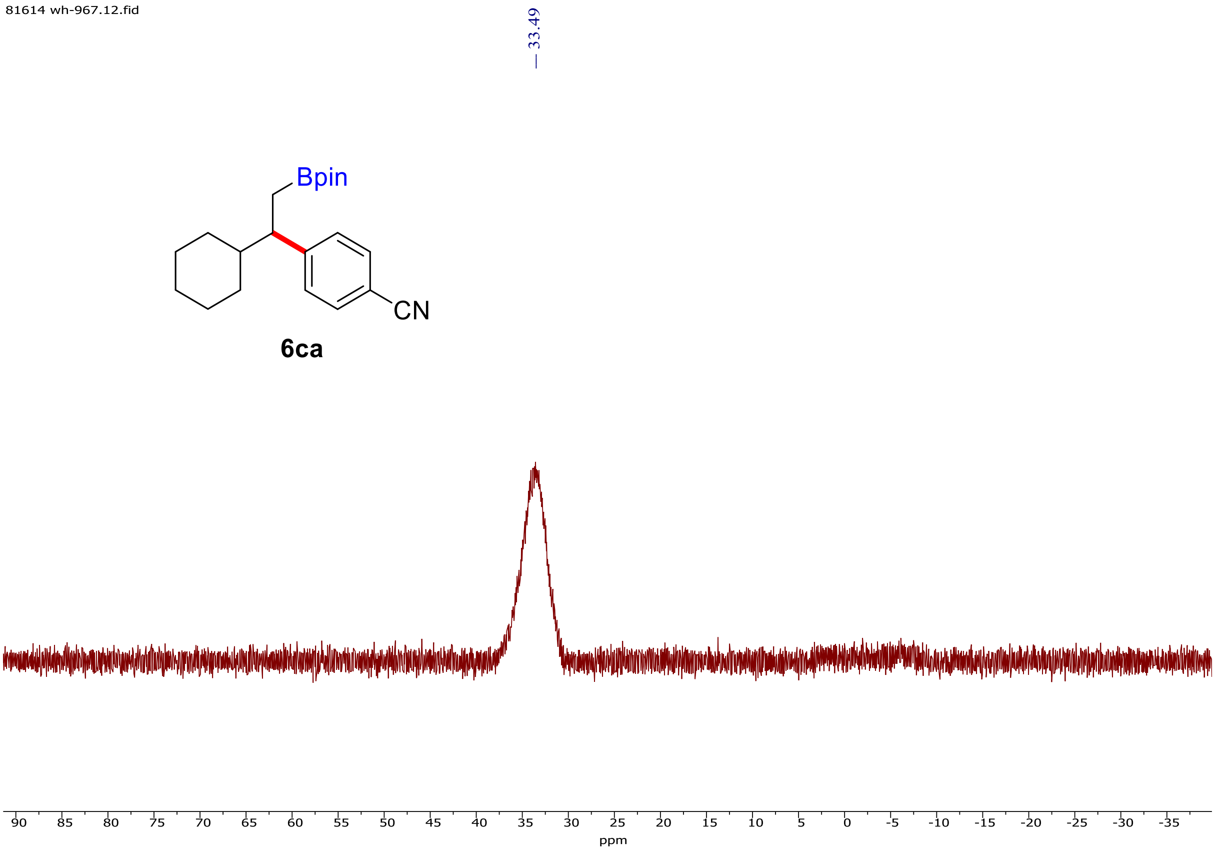


$^{1}$H NMR (400 MHz, $CDCl_3$) of **6da** (*see procedure*)

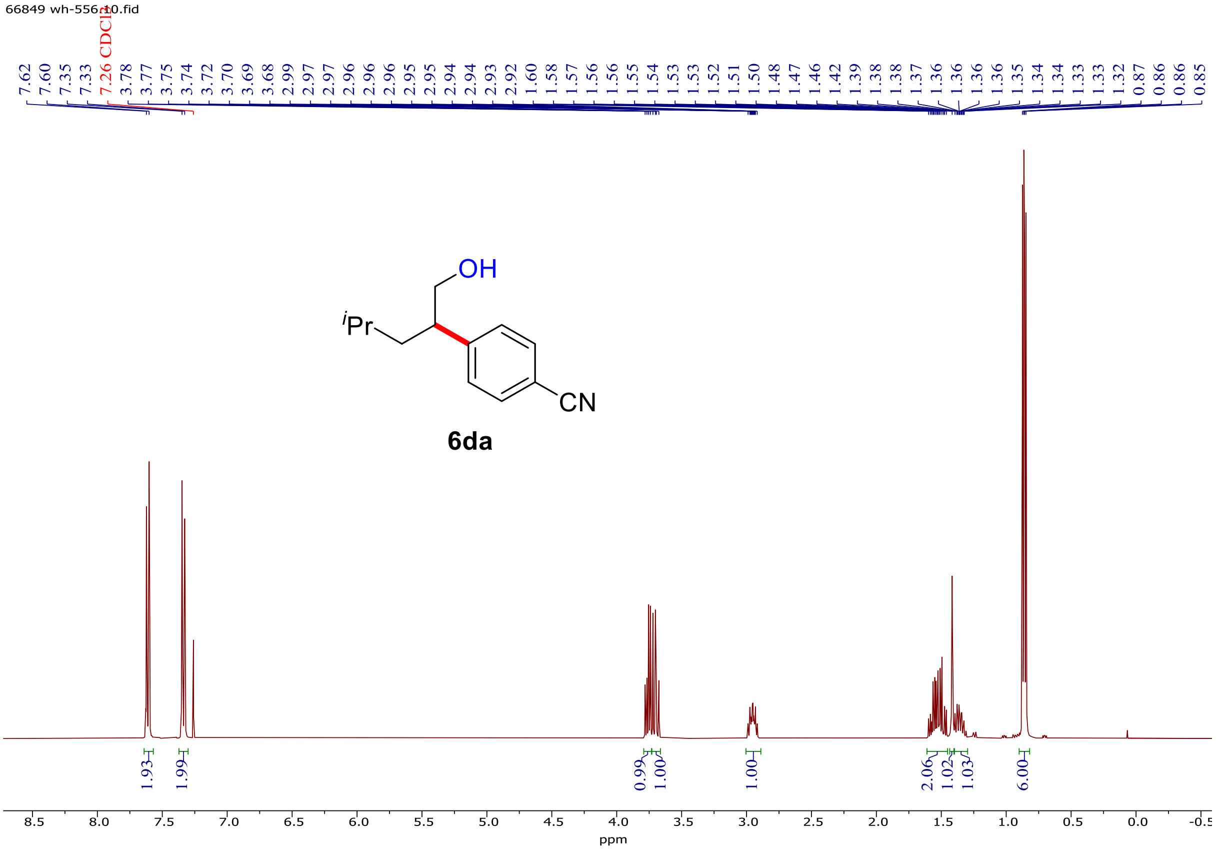

$^{13}$C NMR (101 MHz, $CDCl_3$) of **6da**

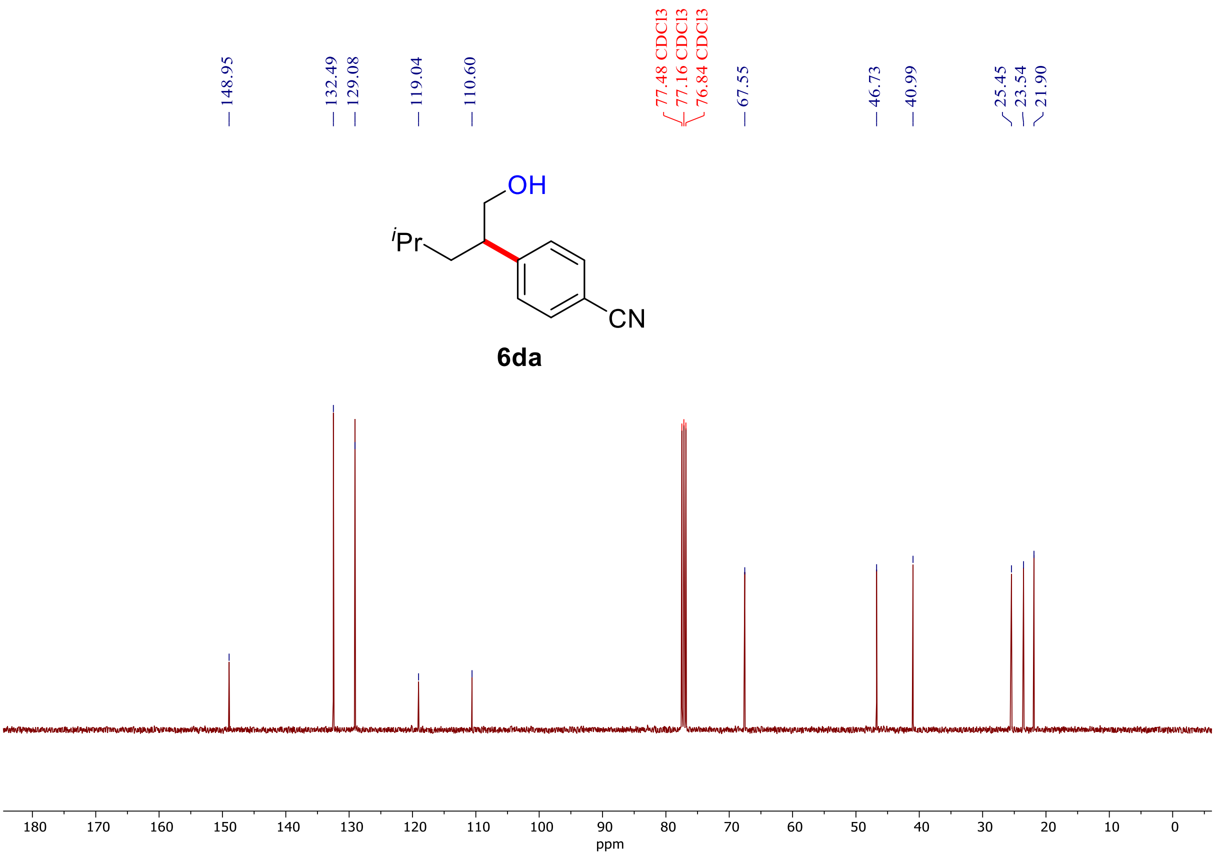


$^{1}$H NMR (400 MHz, $CDCl_3$) of **6ea** (*see procedure*)

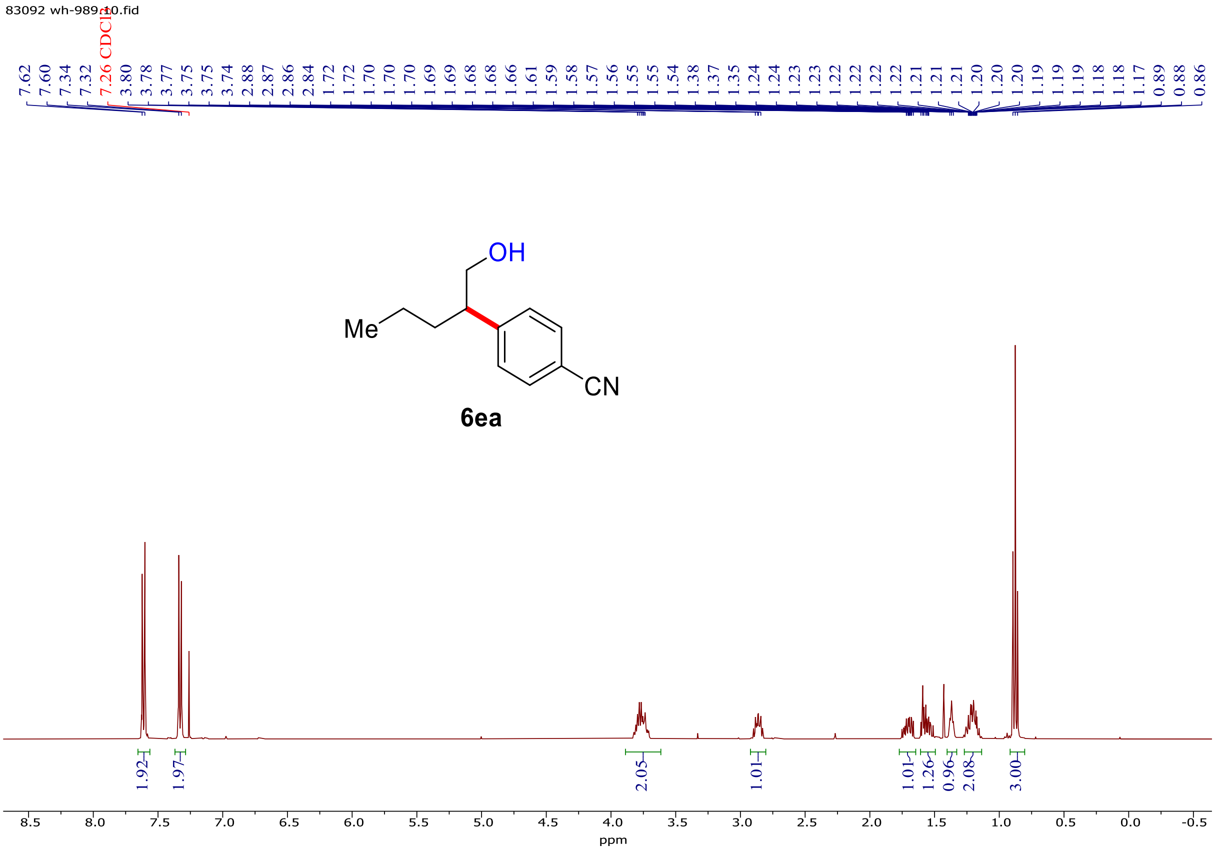

$^{13}$C NMR (101 MHz, $CDCl_3$) of **6ea**

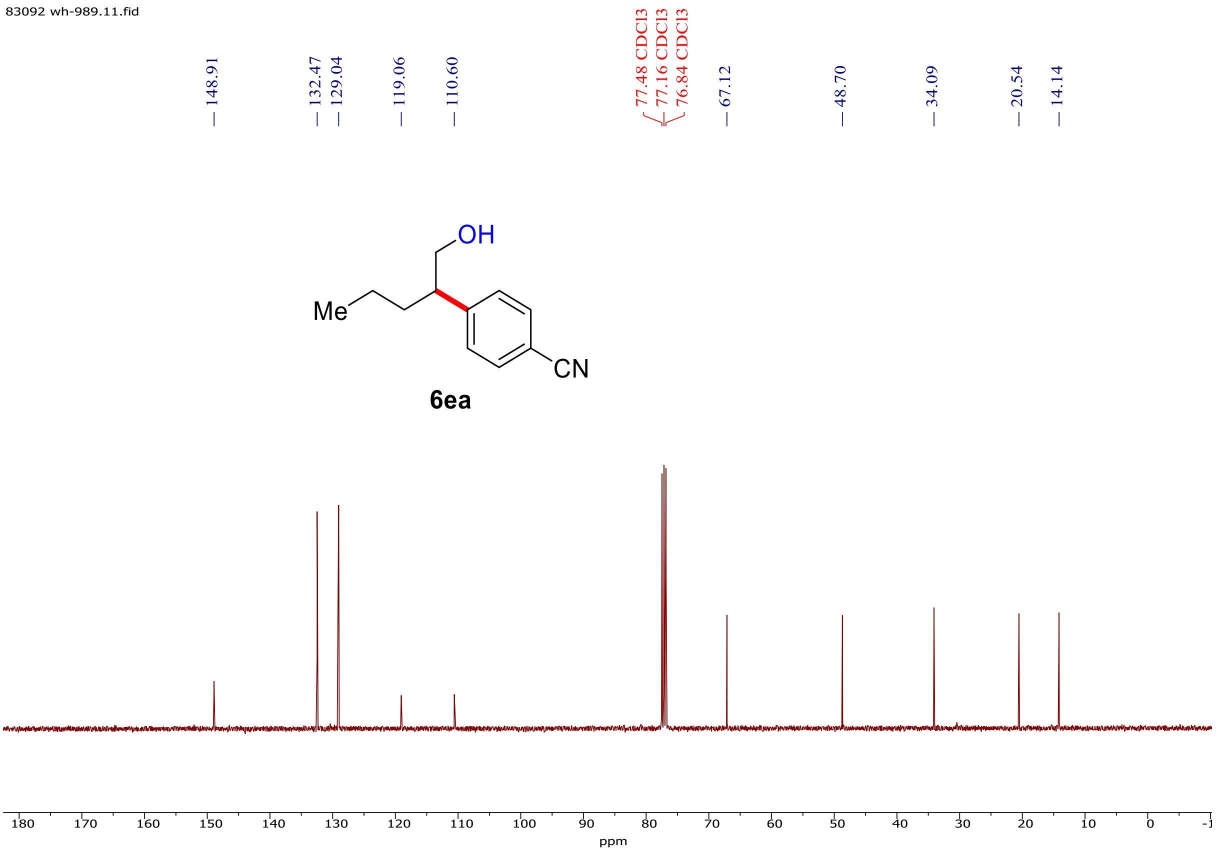


$^{1}$H NMR (400 MHz, $CDCl_3$) of **6ea′** (<u>*see procedure*</u>)

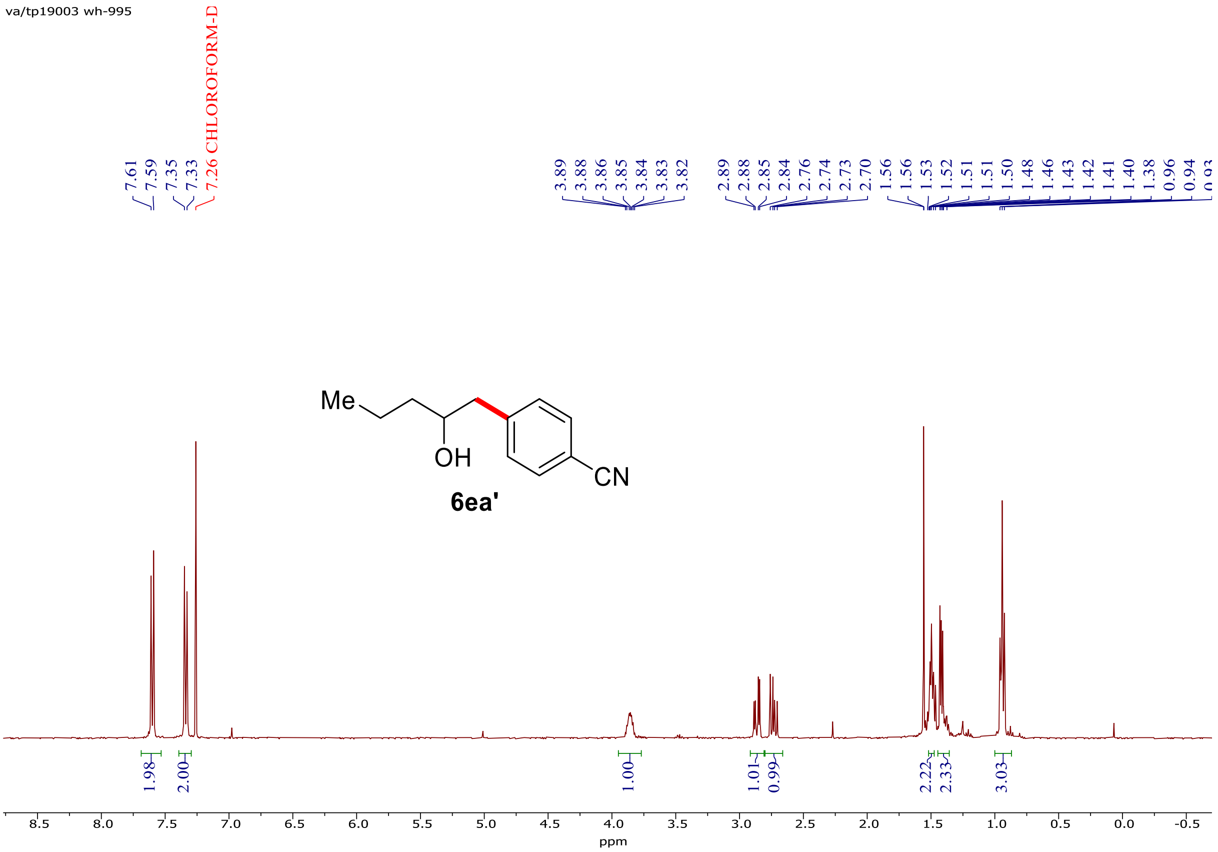

$^{13}$C NMR (101 MHz, $CDCl_3$) of **6ea′**

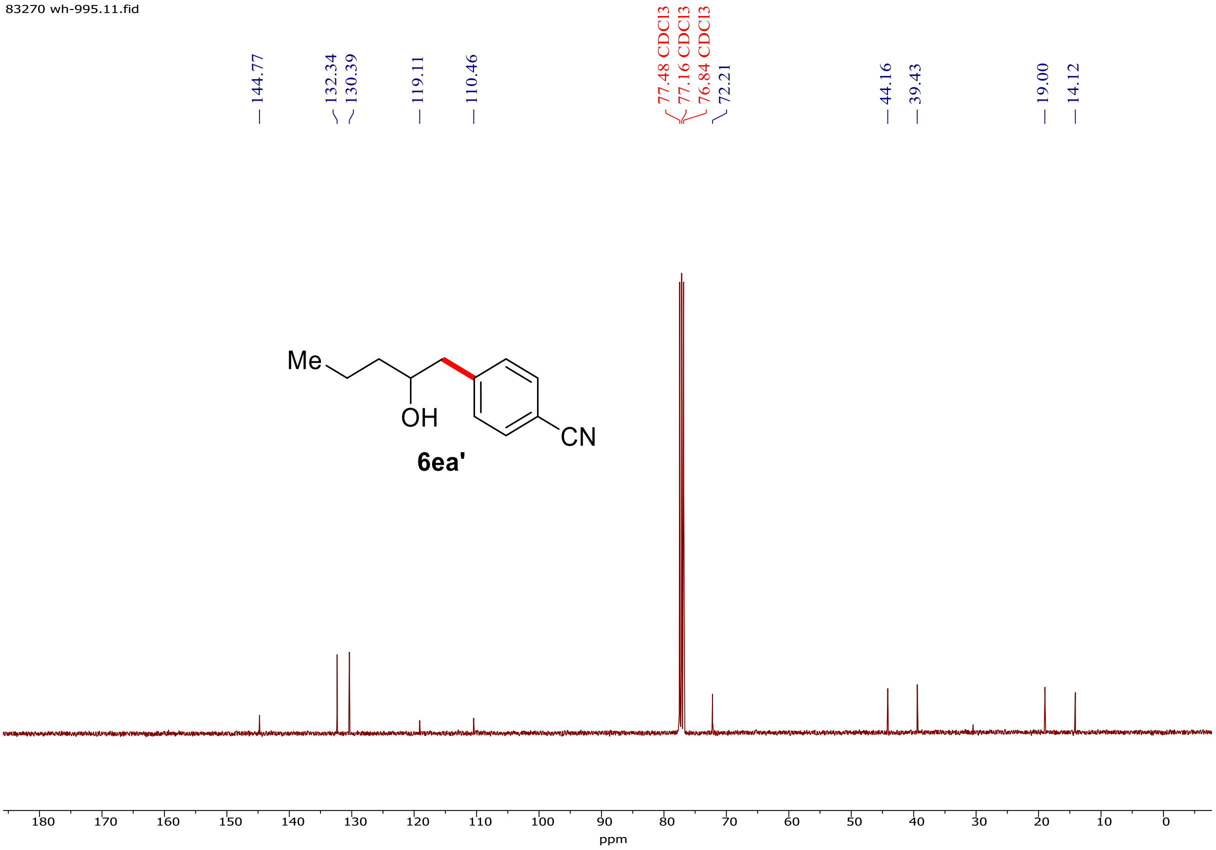


$^{1}$H NMR (400 MHz, $CDCl_3$) of **6fa** (*see procedure*)

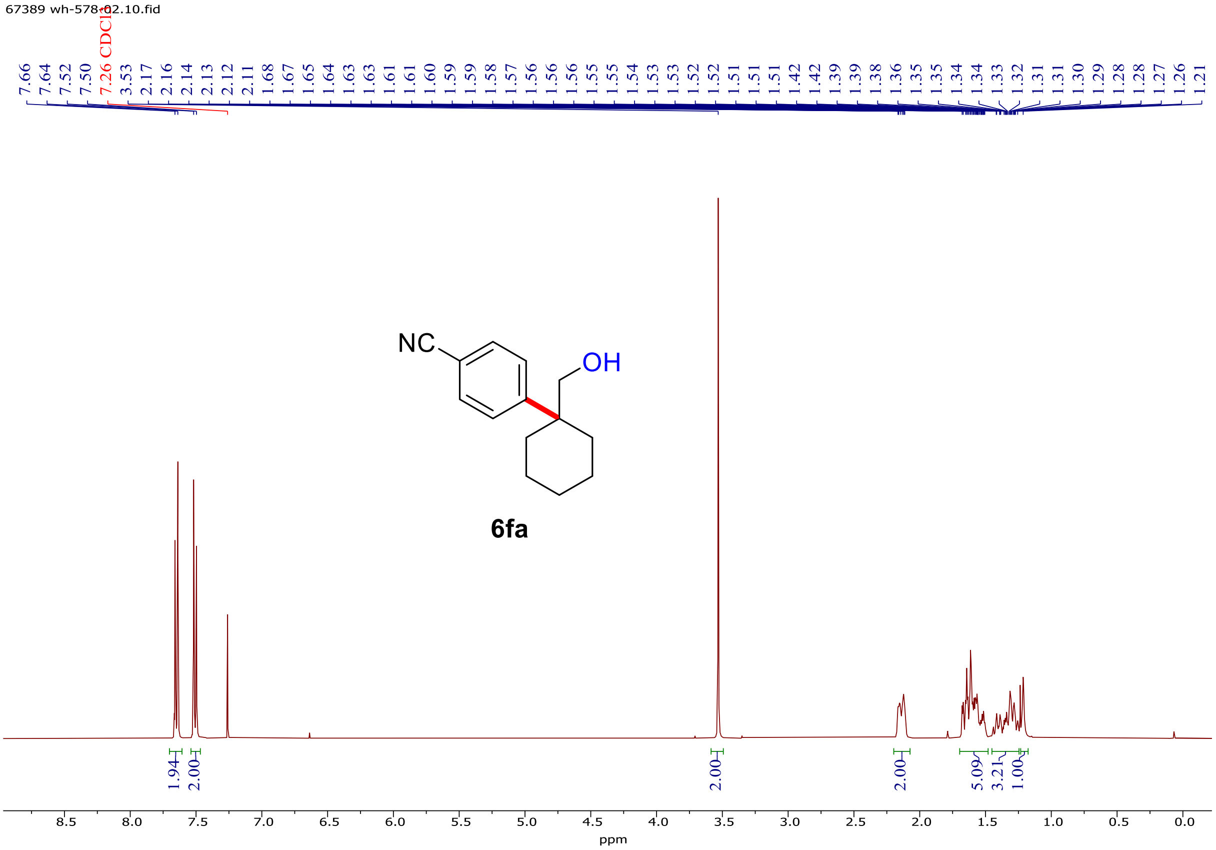

$^{13}$C NMR (101 MHz, $CDCl_3$) of **6fa**

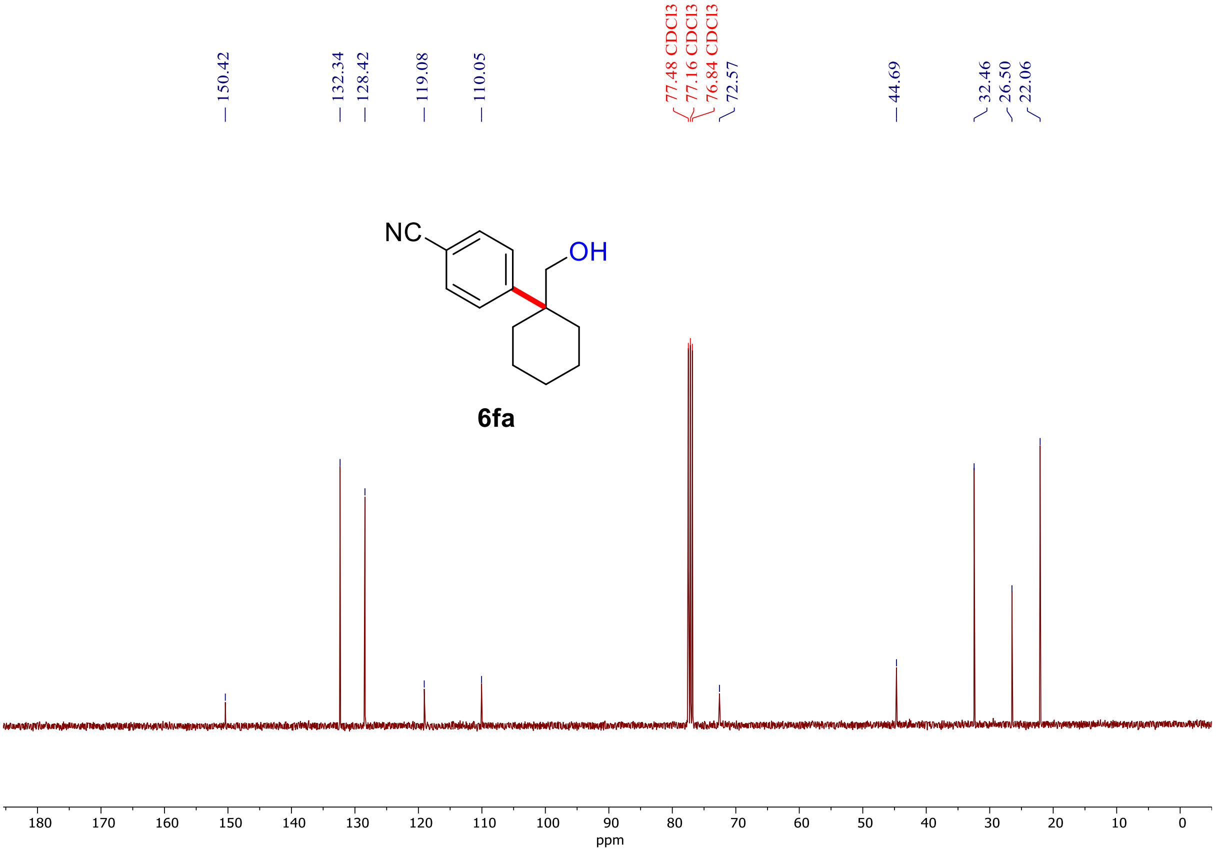


$^1$H NMR (400 MHz, $CDCl_3$) of **(*trans*)-6ga** (<u>*see procedure*</u>)

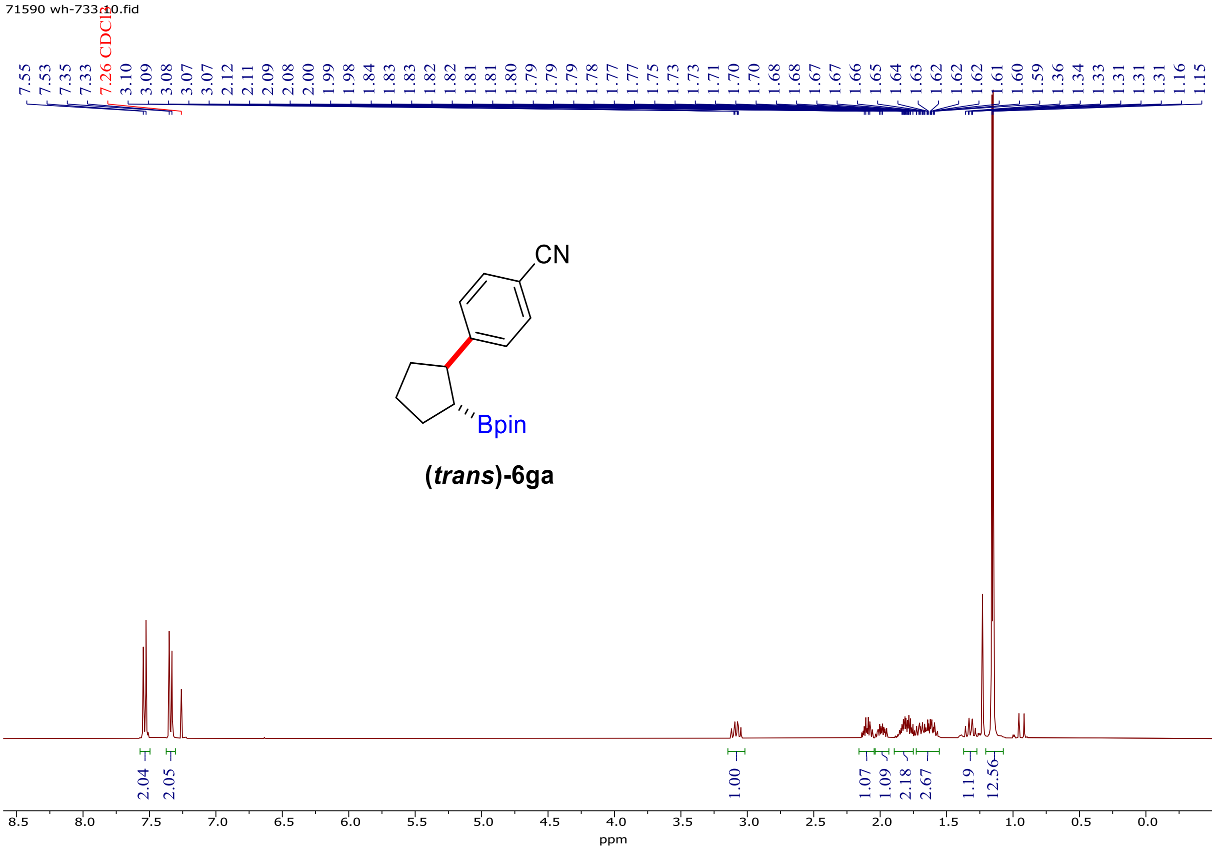

$^{13}$C NMR (101 MHz, $CDCl_3$) of **(*trans*)-6ga**

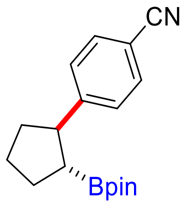


**(*trans*)-6ga**

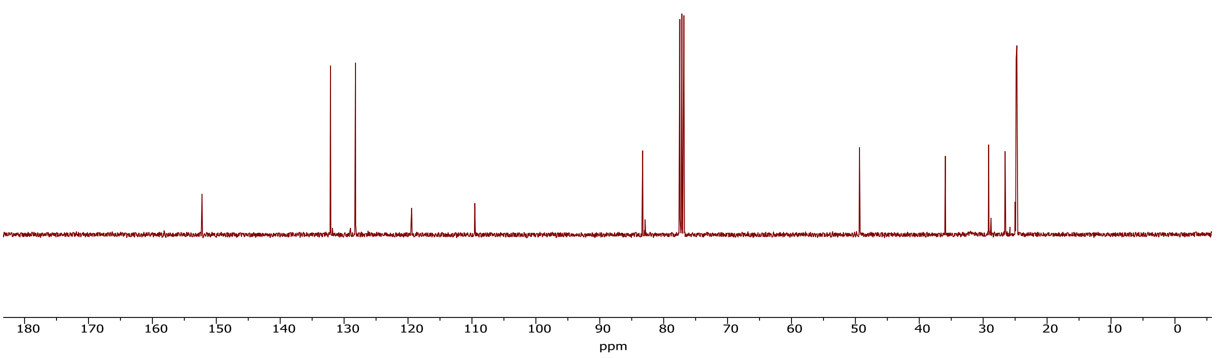

$^{11}B$ NMR (128 MHz, $CDCl_3$) of **(*trans*)-6ga**

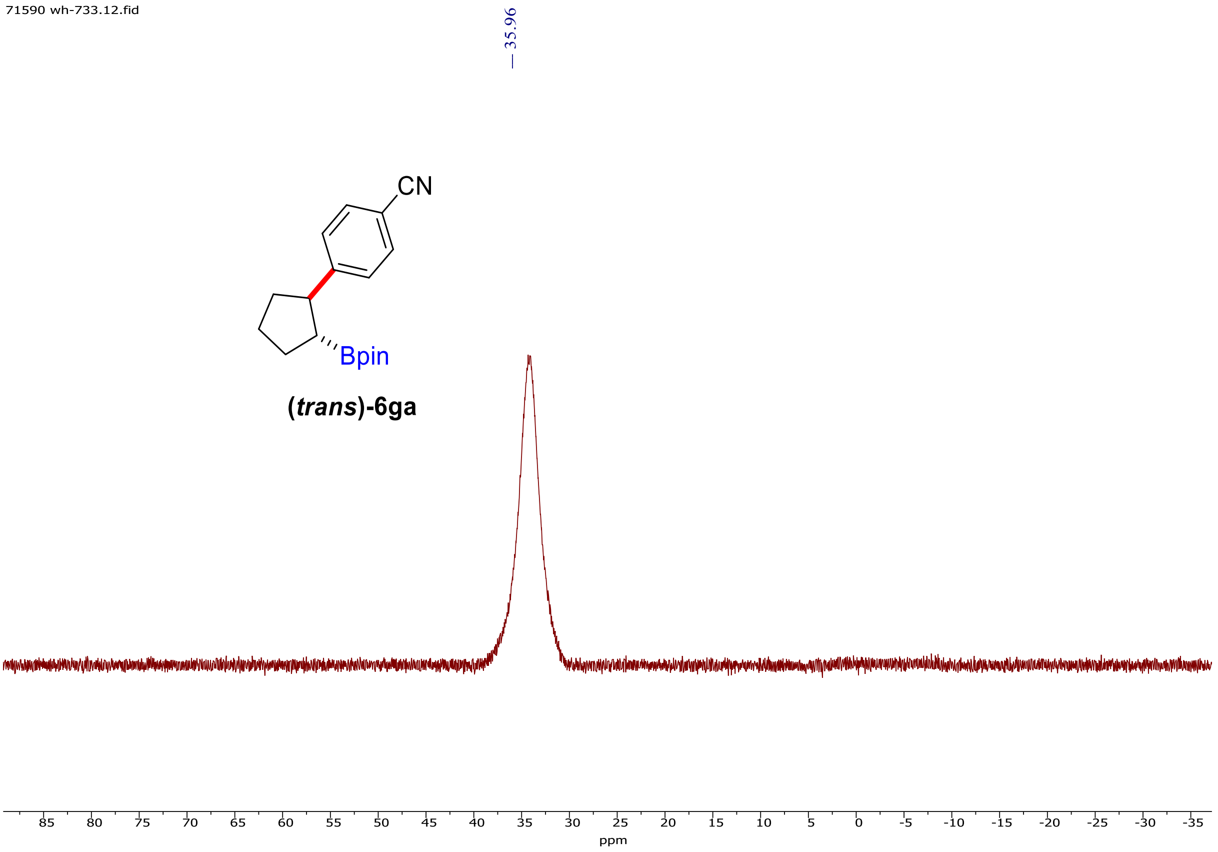


$^{1}H$ NMR (400 MHz, $CDCl_3$) of **(*trans*)-6ha** (see procedure)

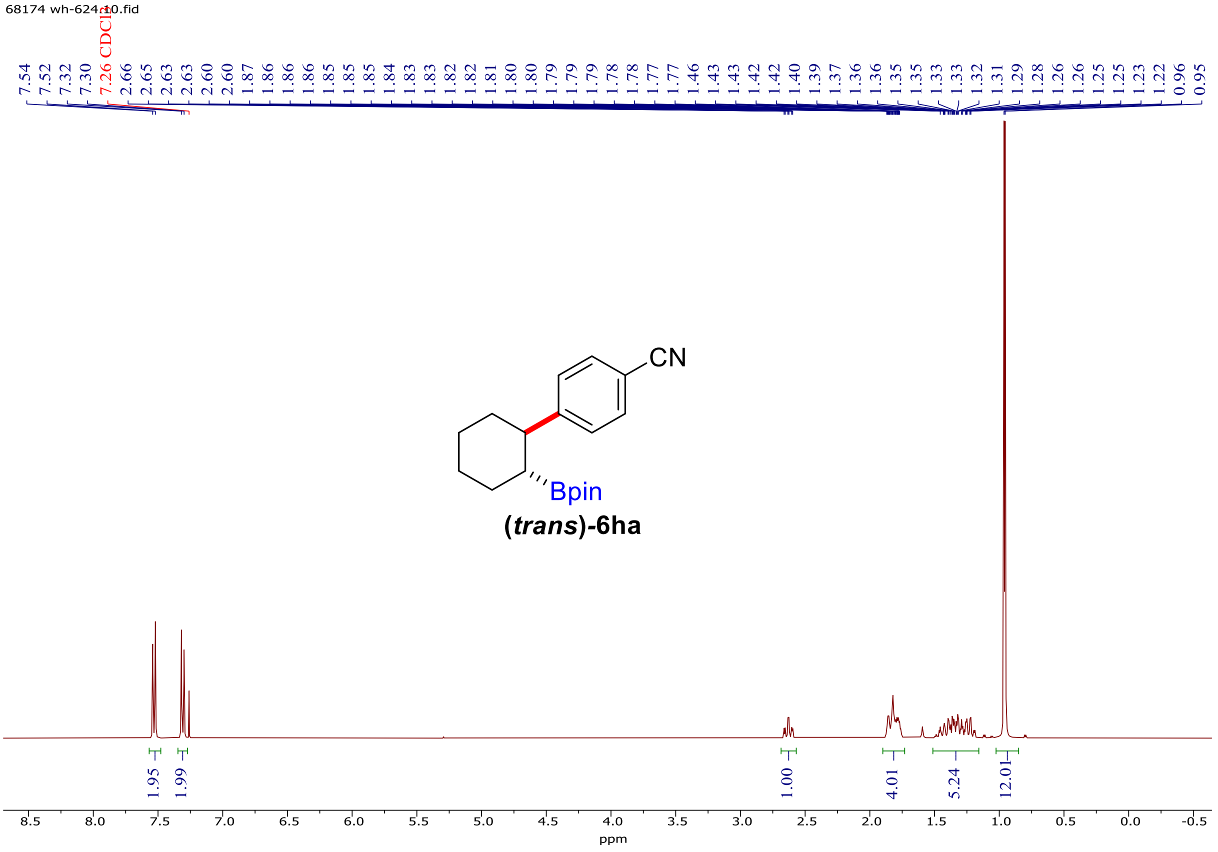

*NOESY* (400 MHz, $CDCl_3$) of **(*trans*)-6ha**

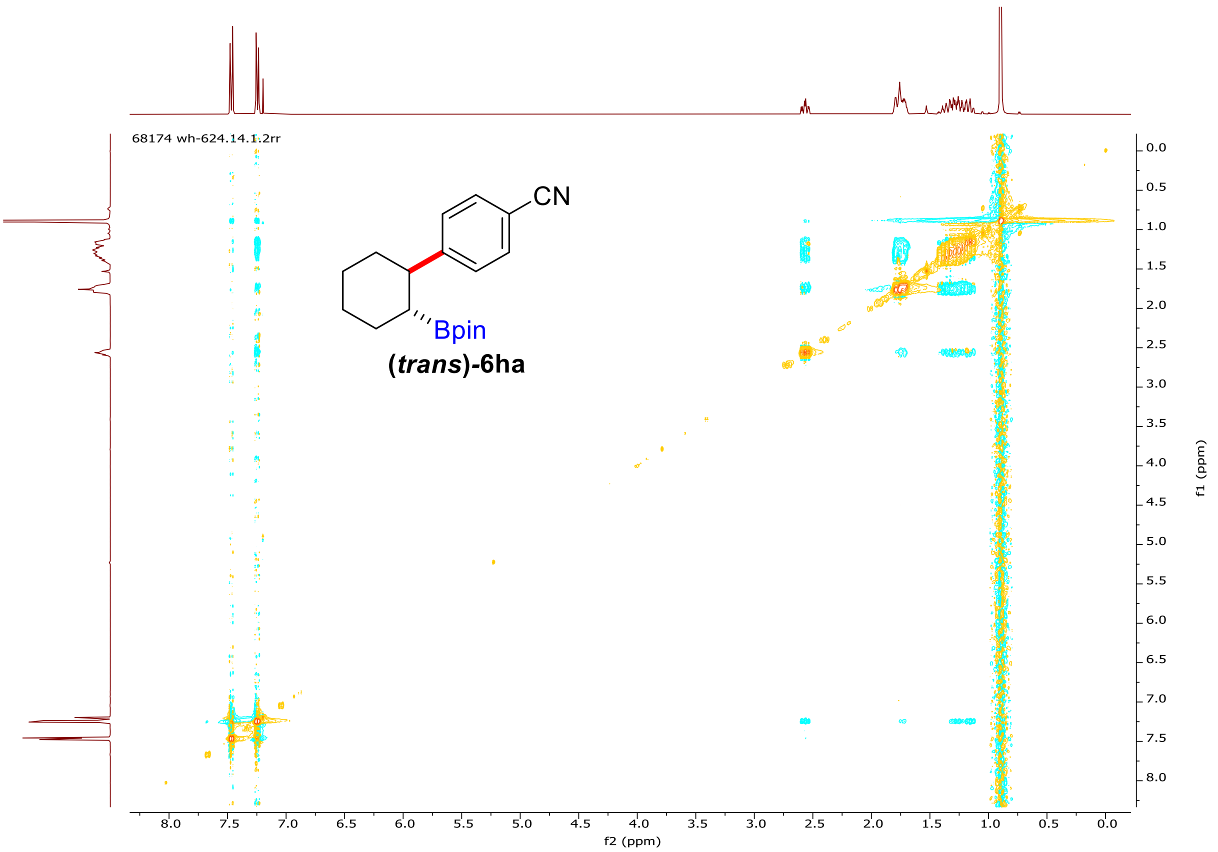


$^{13}C$ NMR (101 MHz, $CDCl_3$) of **(*trans*)-6ha**

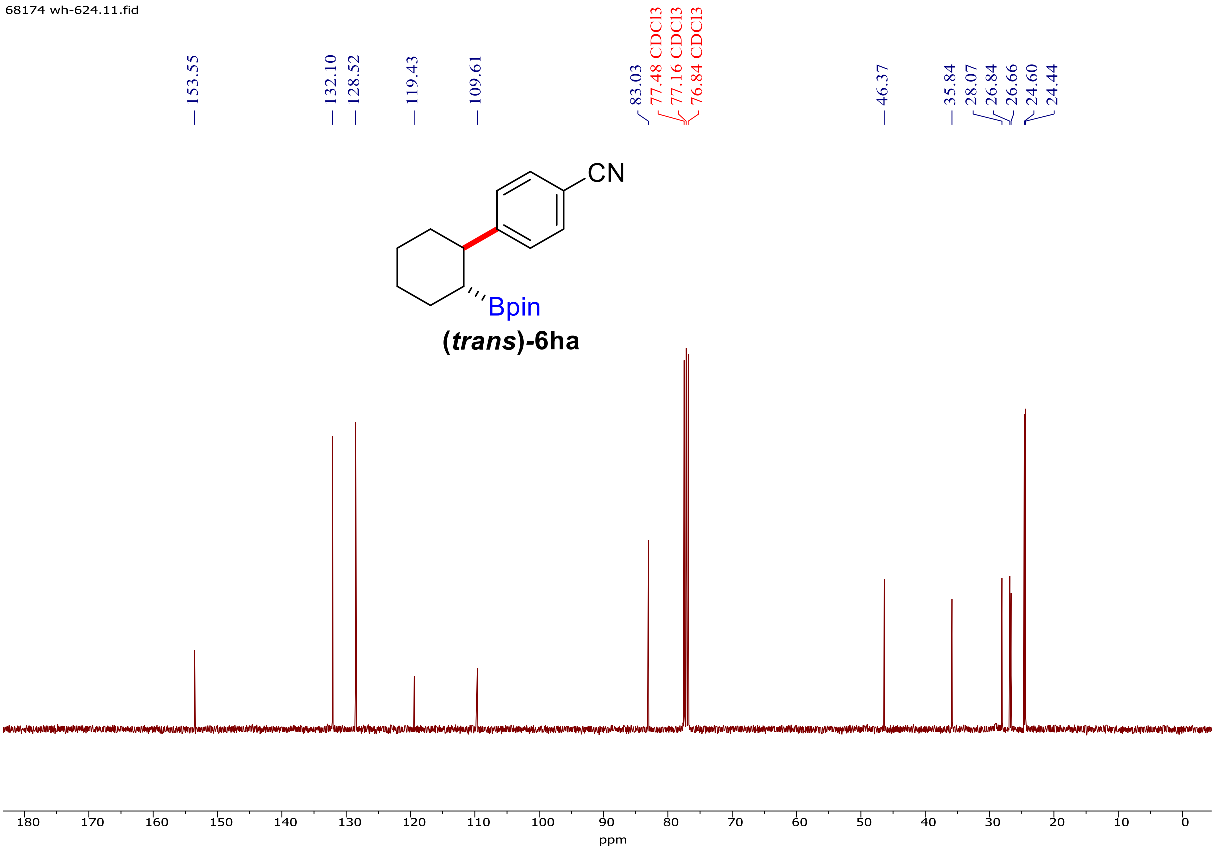

$^{11}B$ NMR (128 MHz, $CDCl_3$) of **(*trans*)-6ha**

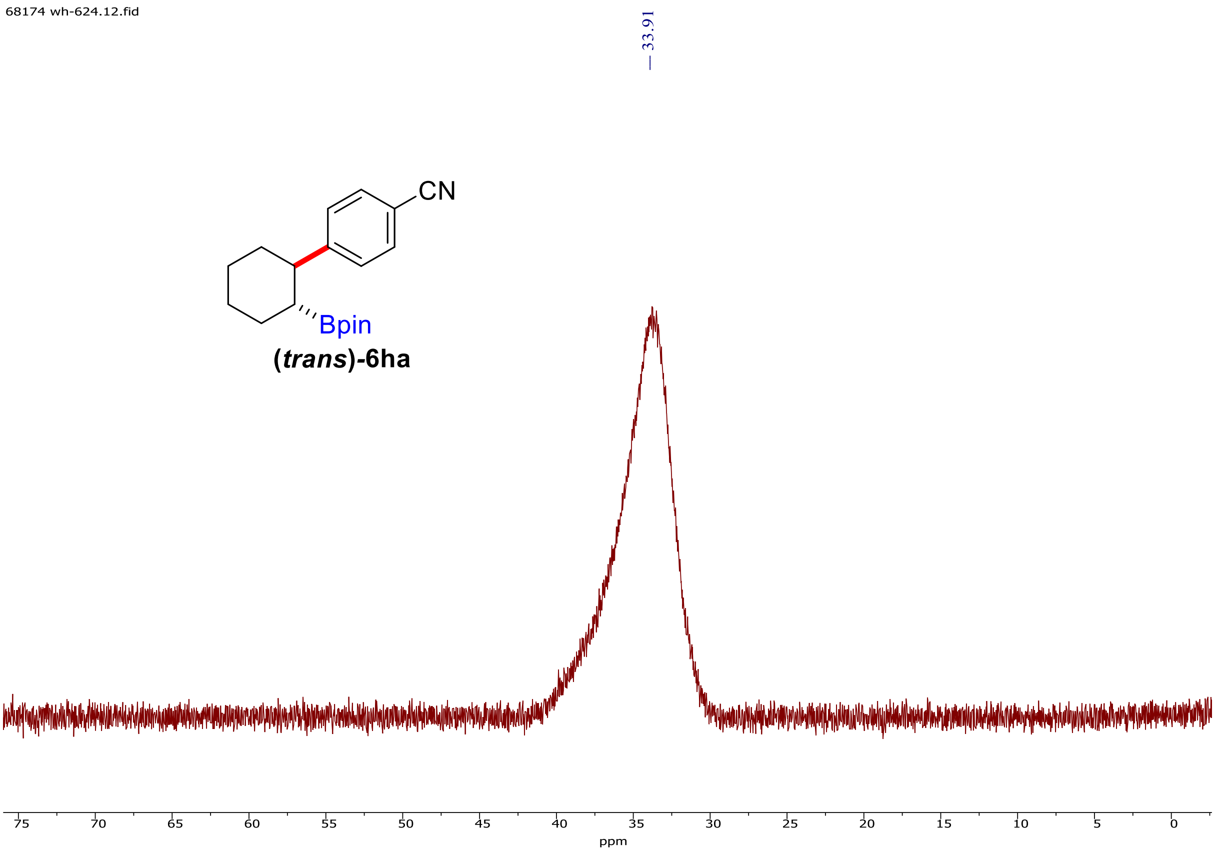


$^{1}H$ NMR (400 MHz, $CDCl_3$) of **6ia** (*see procedure*)

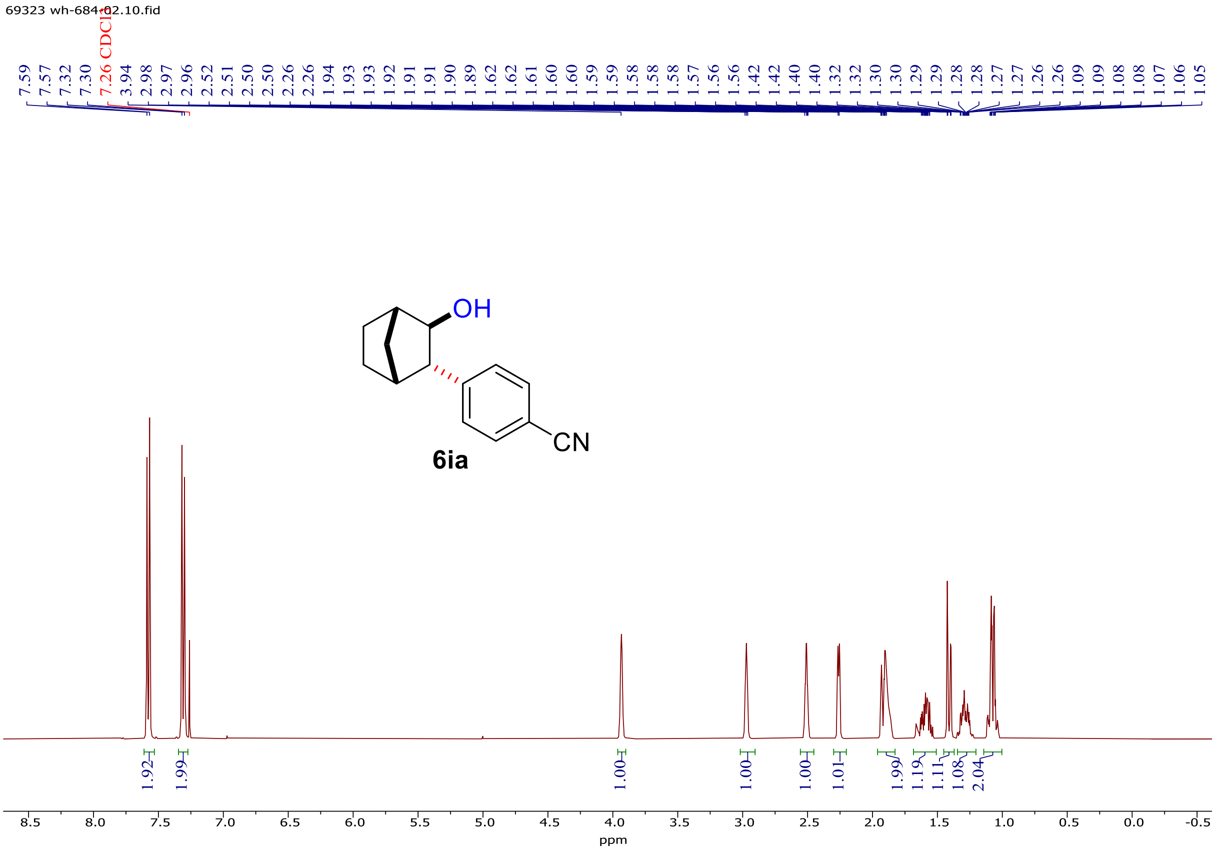

$^{13}$C NMR (101 MHz, CDCl$_3$) of **6ia**

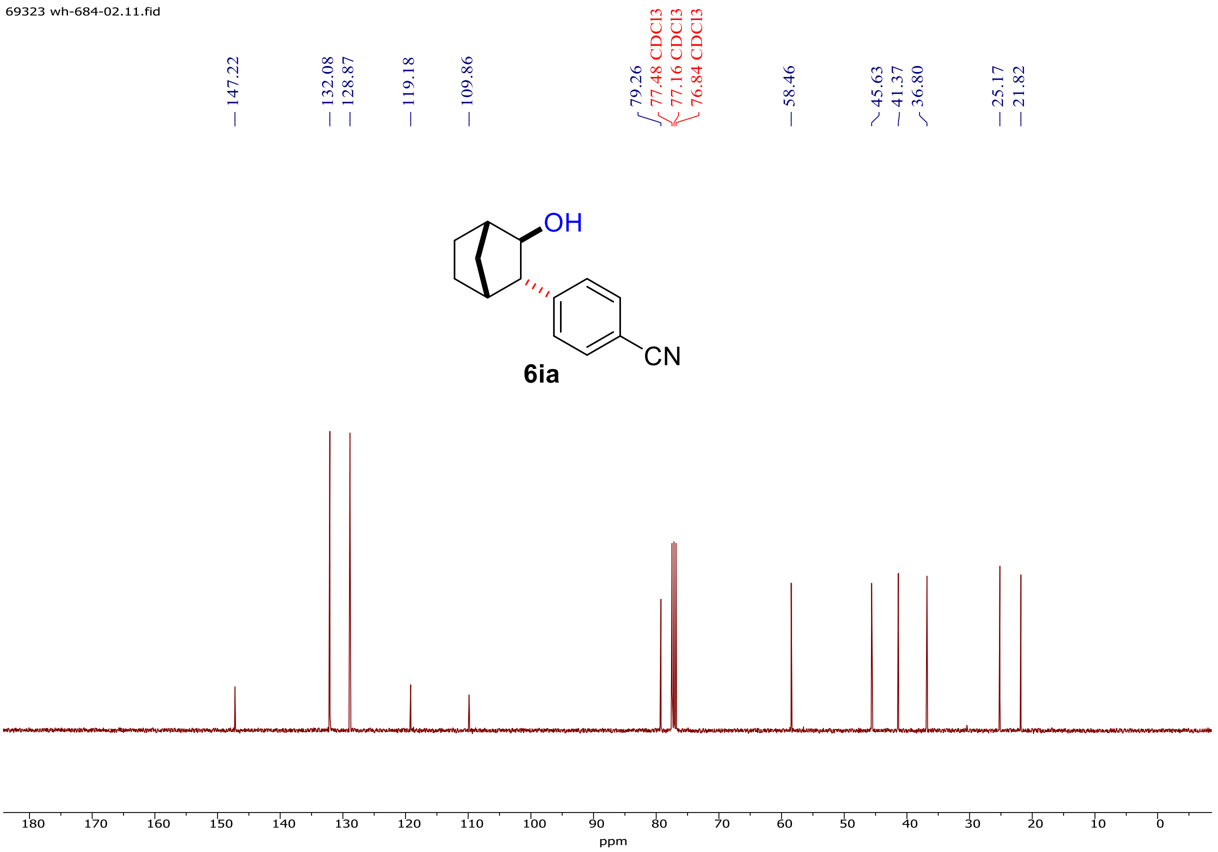


$^{1}$H NMR (400 MHz, CDCl$_3$) of **6ja** (*see procedure*)

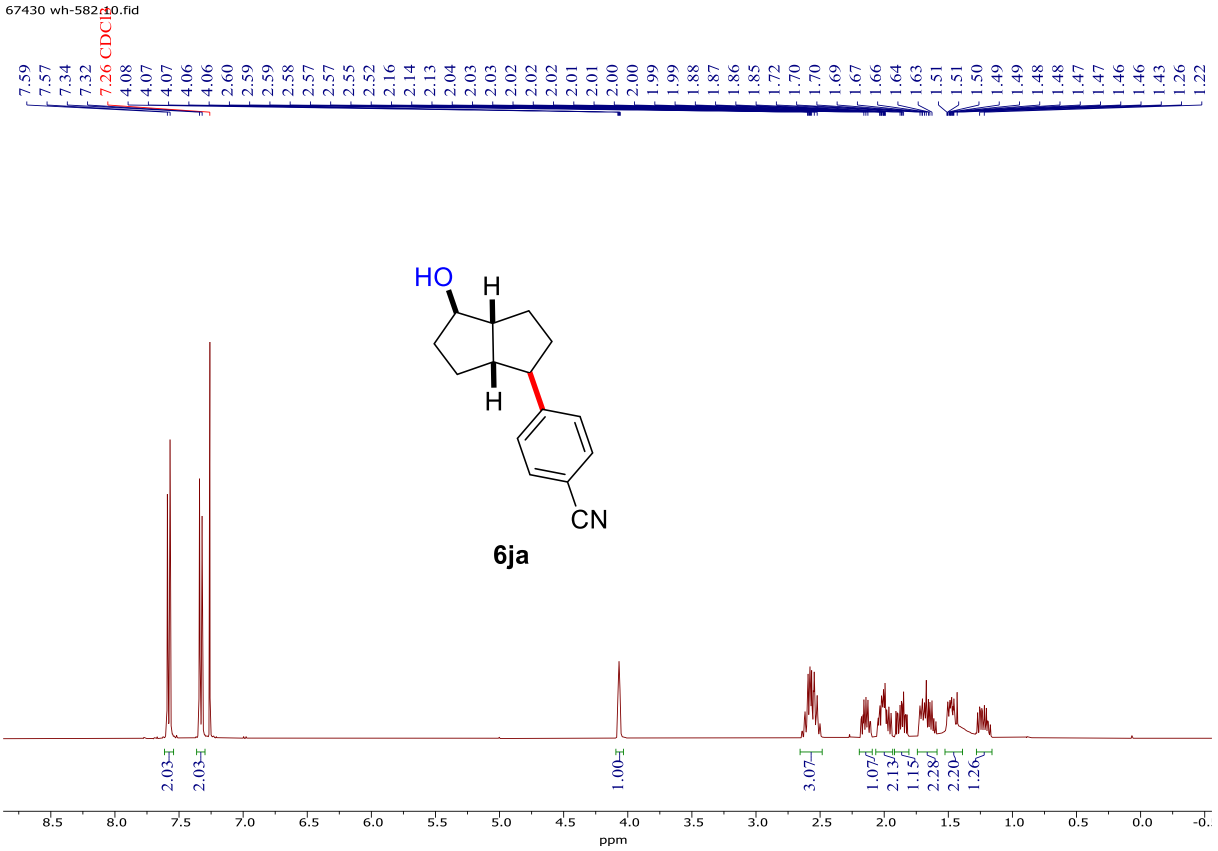

$^{13}$C NMR (101 MHz, $CDCl_3$) of **6ja**

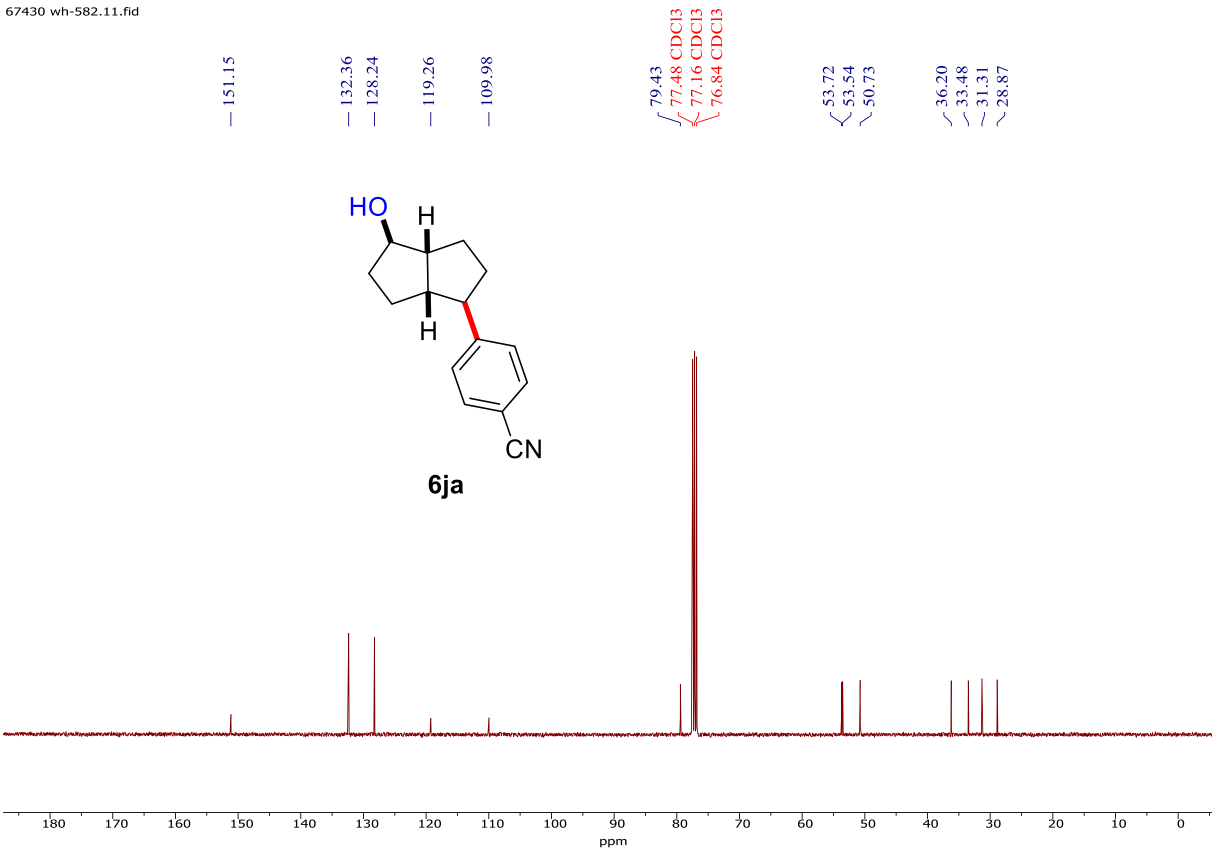


$^{1}$H NMR (400 MHz, $CDCl_3$) of **6ka** (*see procedure*)

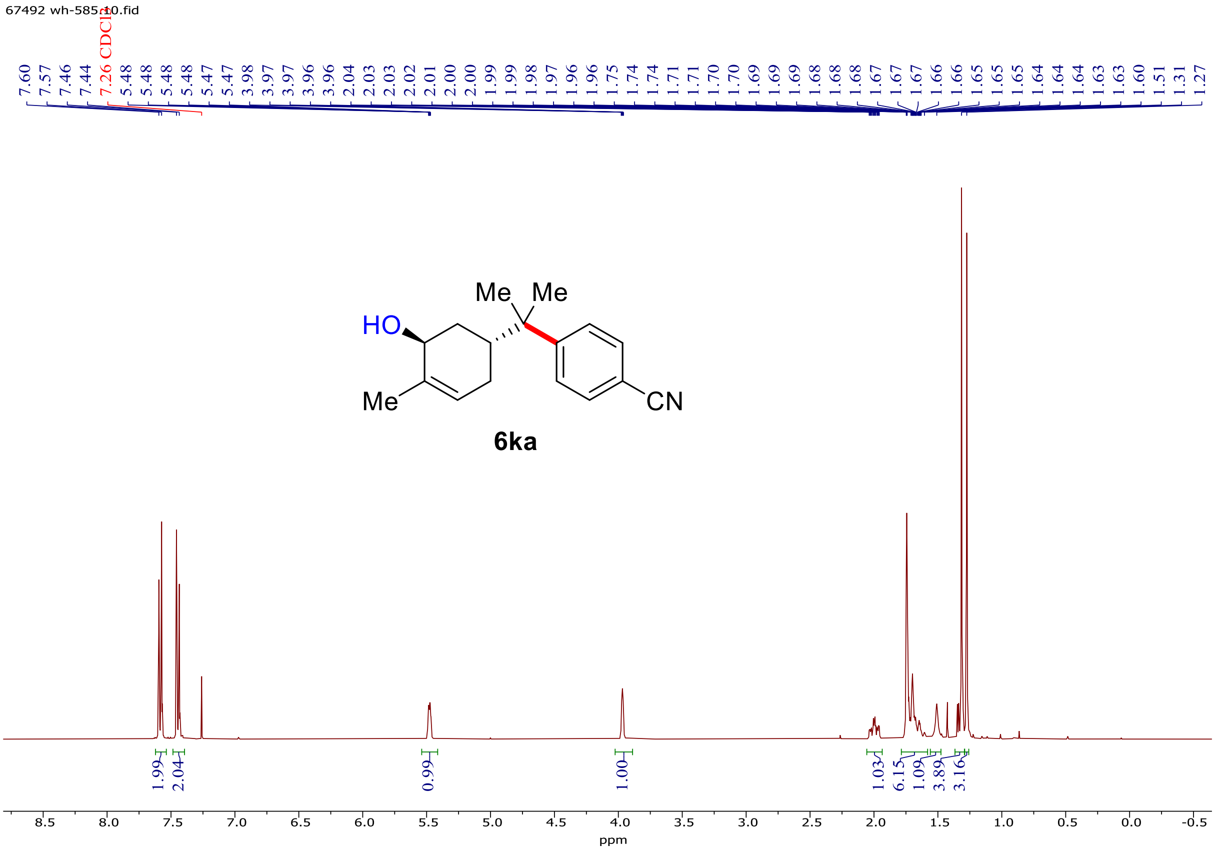

$^{13}C$ NMR (101 MHz, $CDCl_3$) of **6ka**

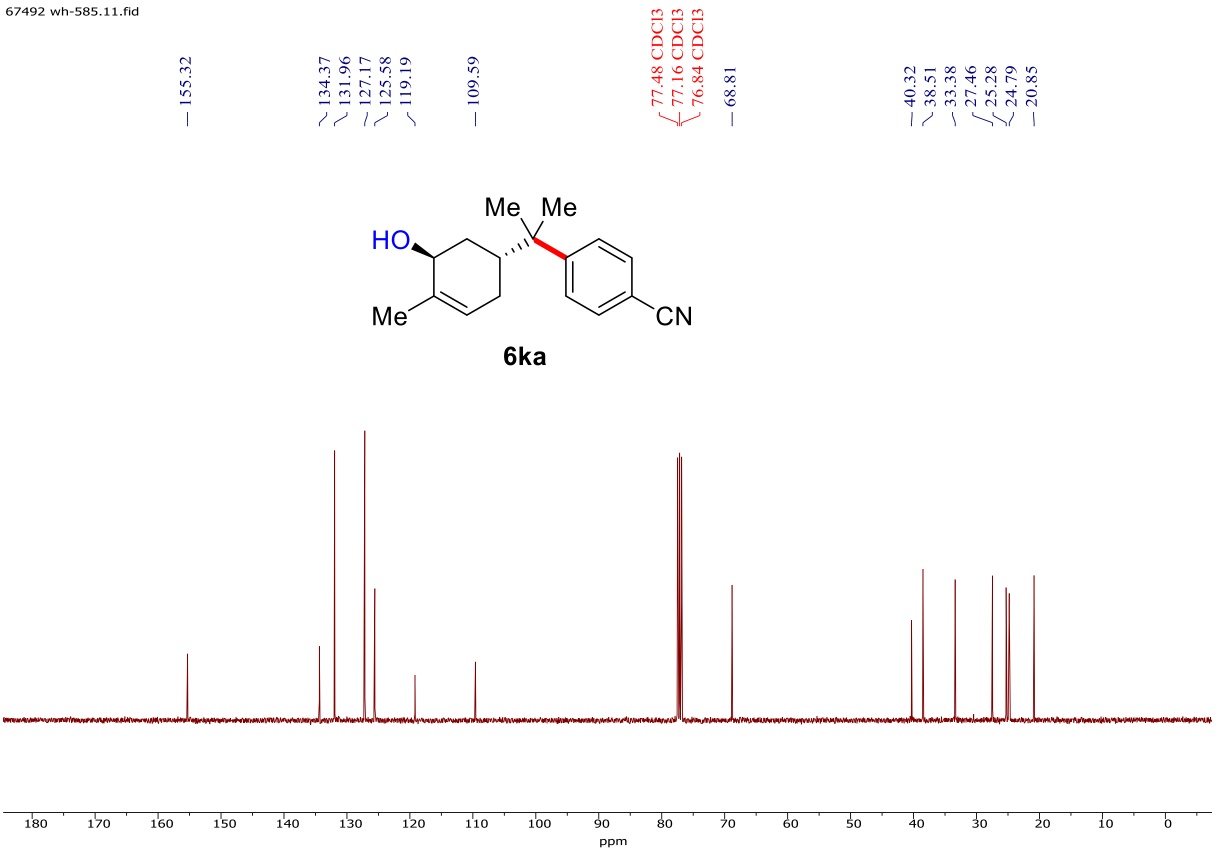


$^{1}H$ NMR (400 MHz, $CDCl_3$) of **6la** (*see procedure*)

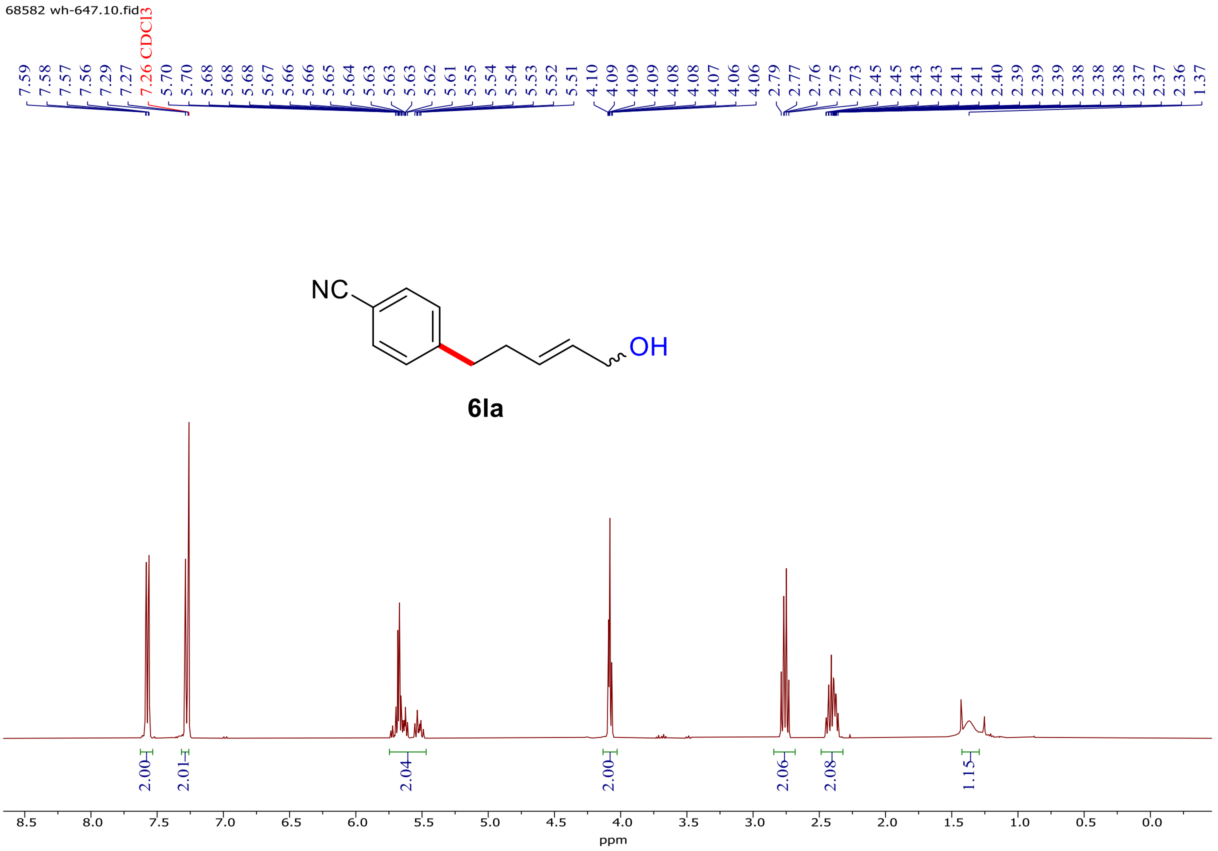

## $^{13}$C NMR (101 MHz, $CDCl_3$) of **6la**

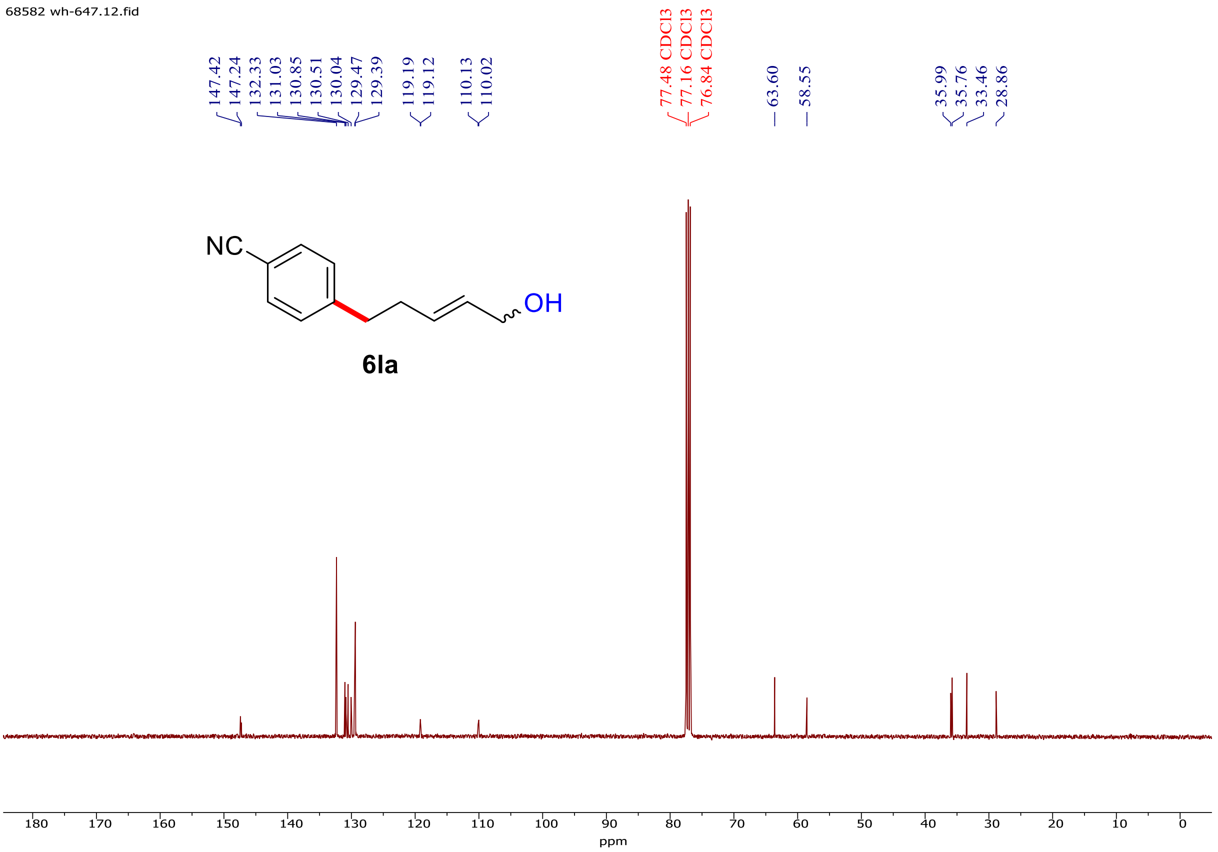


## $^{1}$H NMR (400 MHz, $CDCl_3$) of **(*cis*)-6ga** (*see procedure*)

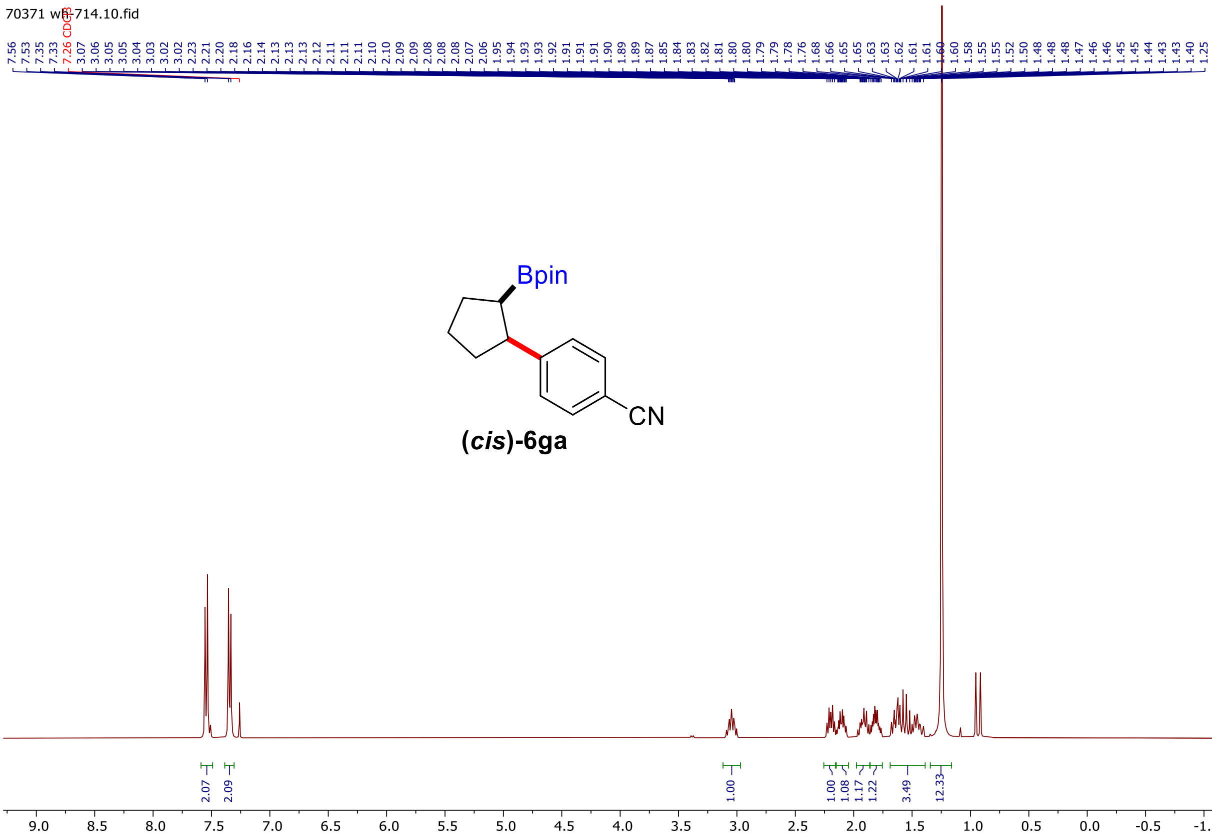

$^{13}$C NMR (101 MHz, $CDCl_3$) of **(*cis*)-6ga**

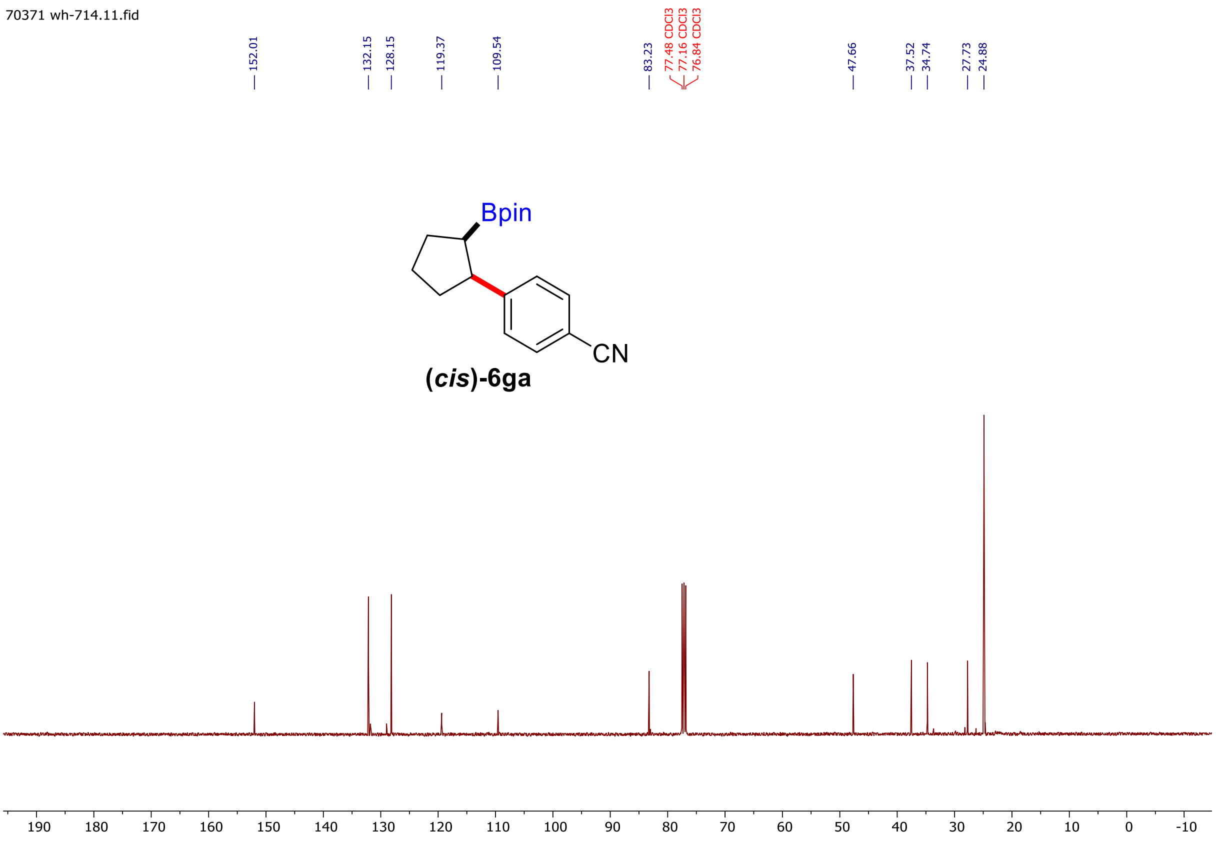


$^{11}$B NMR (128 MHz, $CDCl_3$) of **(*cis*)-6ga**

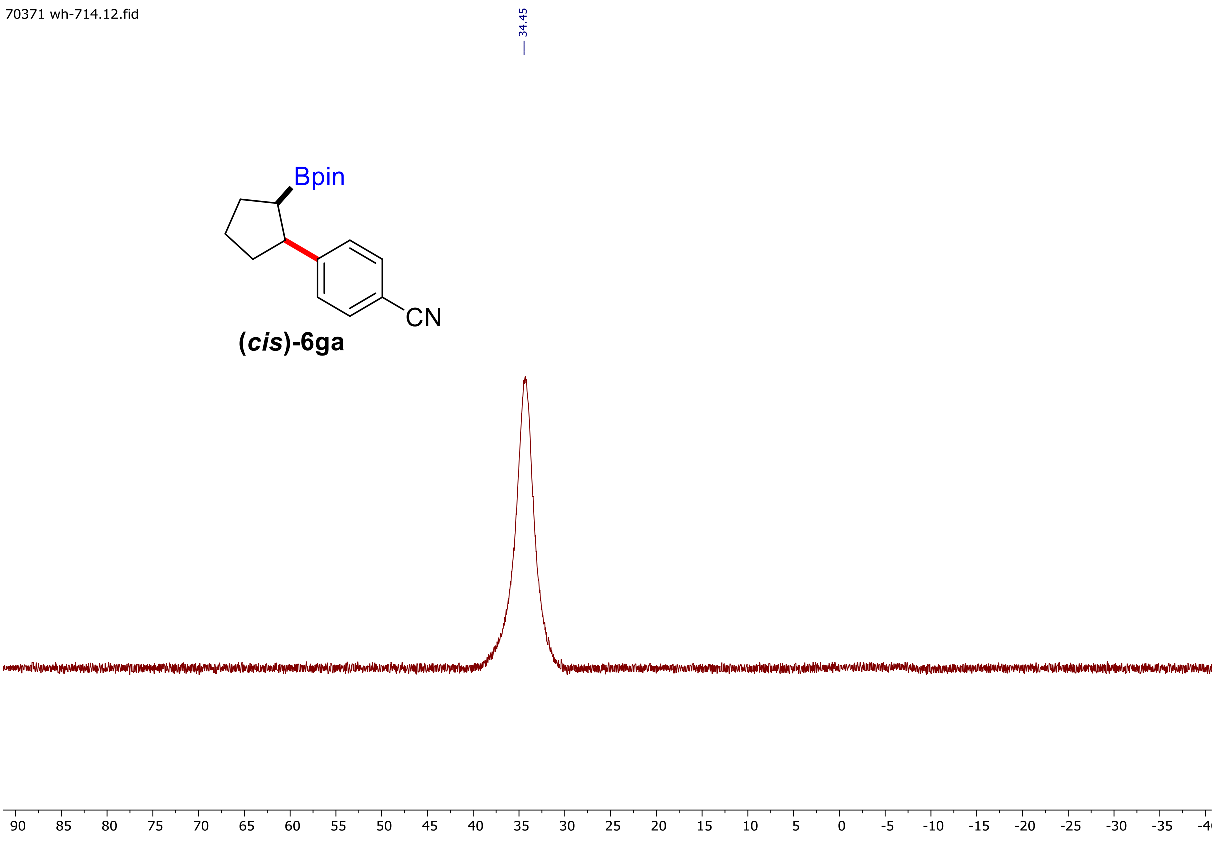

$^{1}$H NMR (400 MHz, $CDCl_3$) of **8a** (*see procedure*)

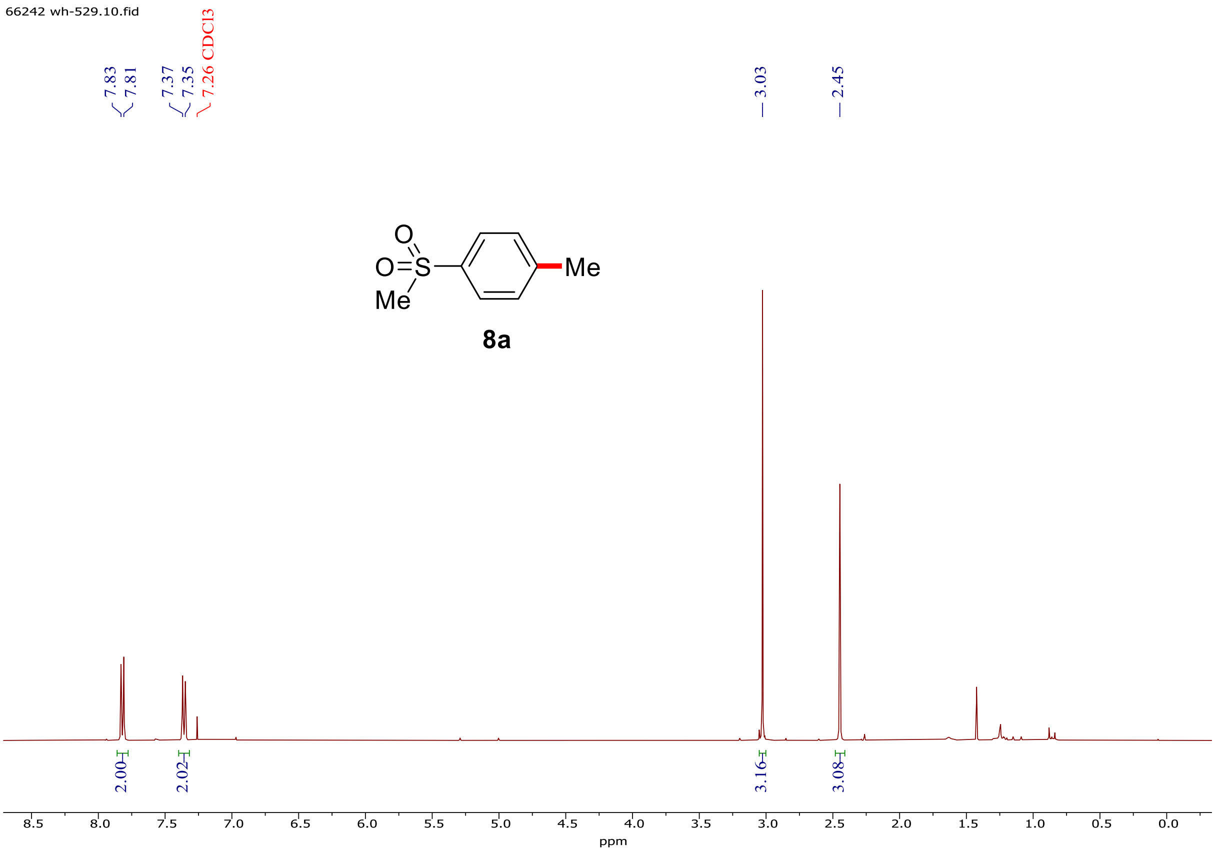


$^{13}$C NMR (101 MHz, $CDCl_3$) of **8a**

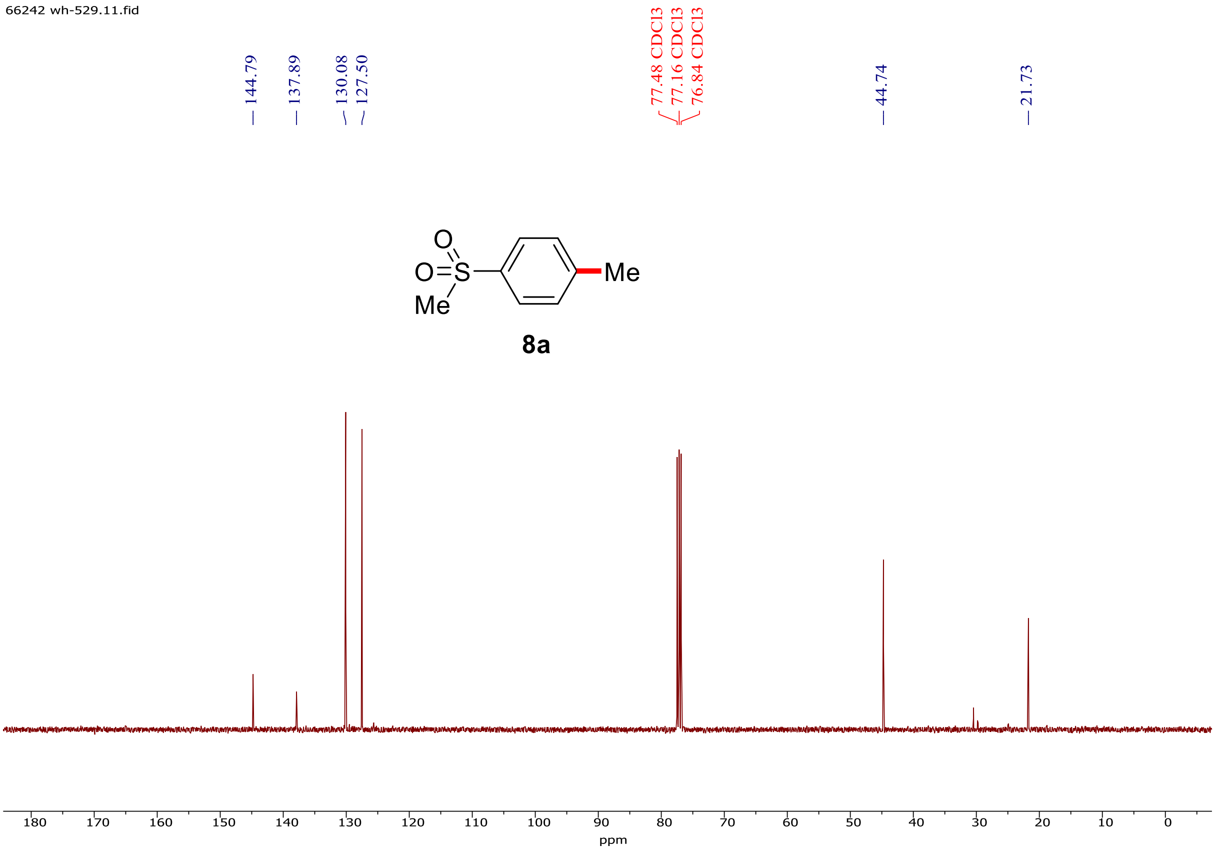

$^{1}$H NMR (400 MHz, $CDCl_3$) of **8b** (*see procedure*)

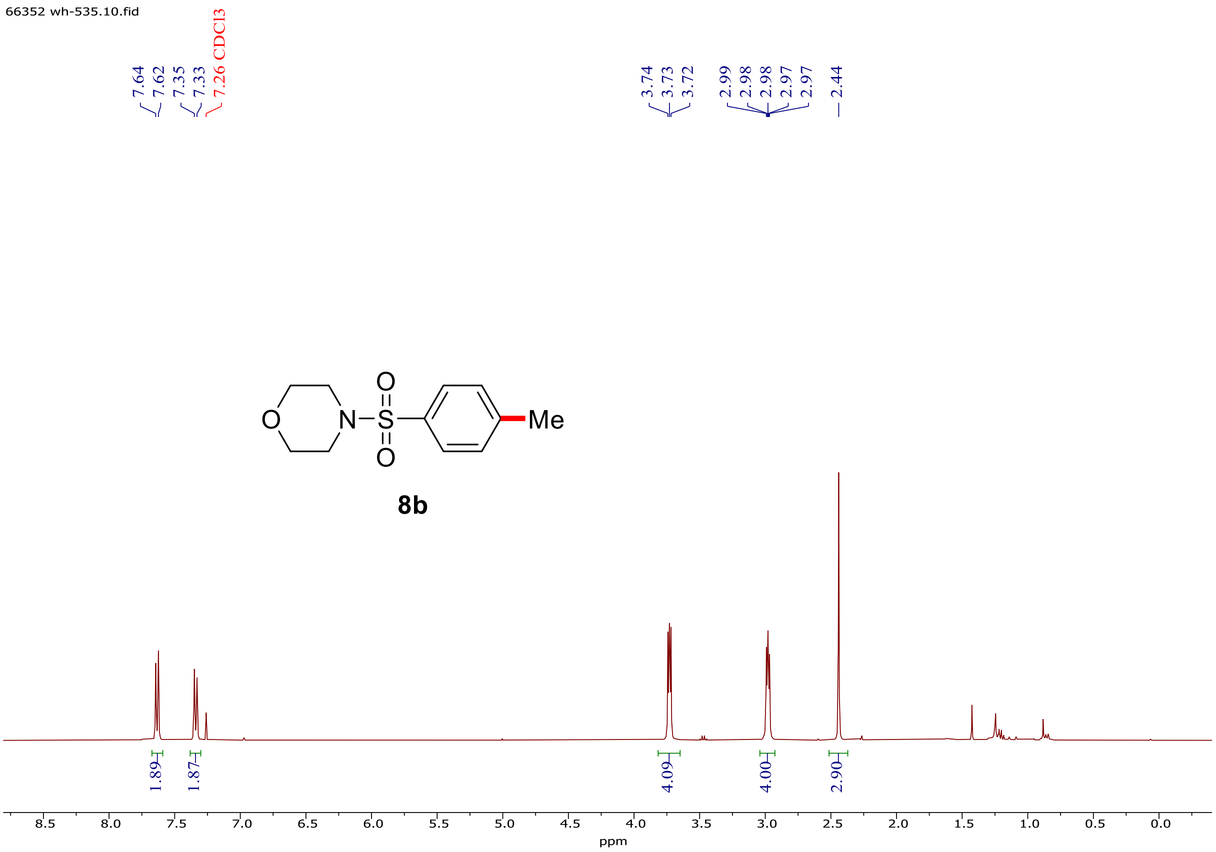


$^{13}$C NMR (101 MHz, $CDCl_3$) of **8b**

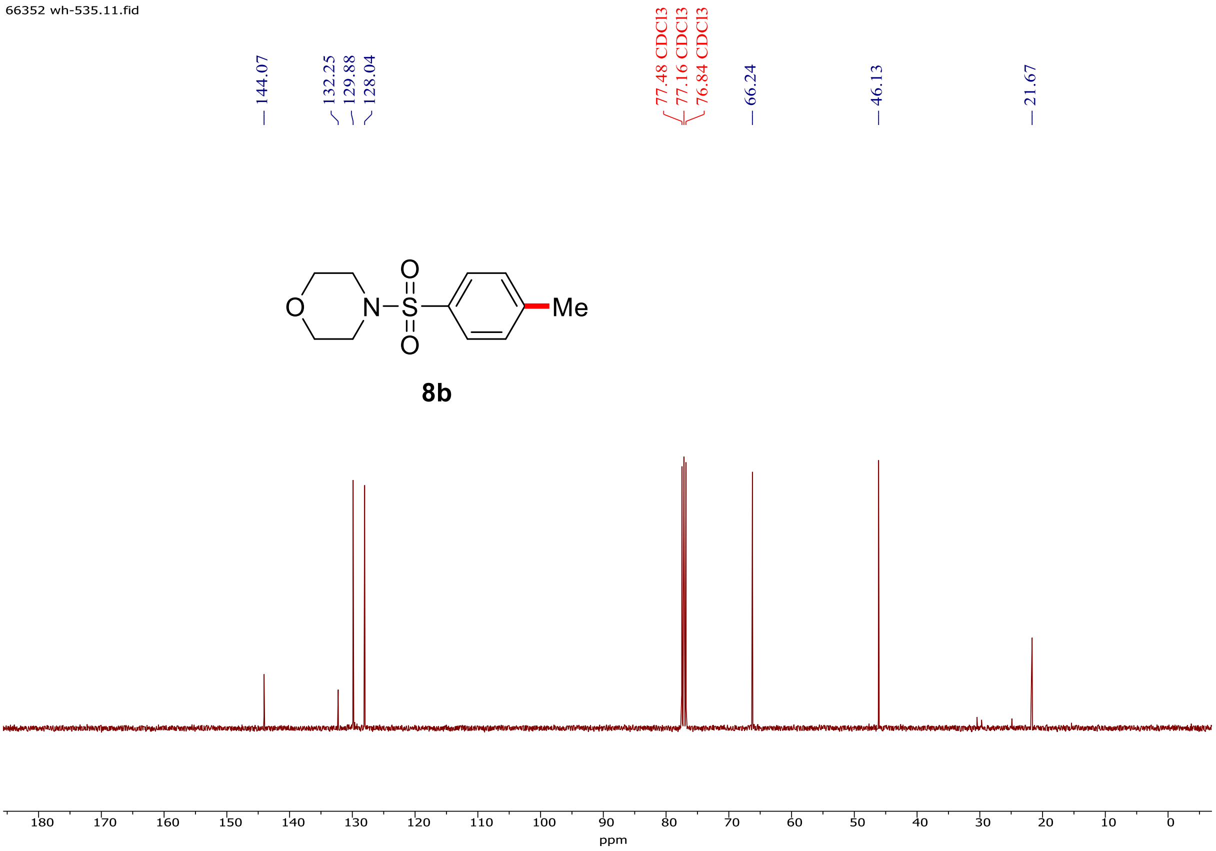

$^{1}$H NMR (400 MHz, $CDCl_3$) of **8c** (see procedure)

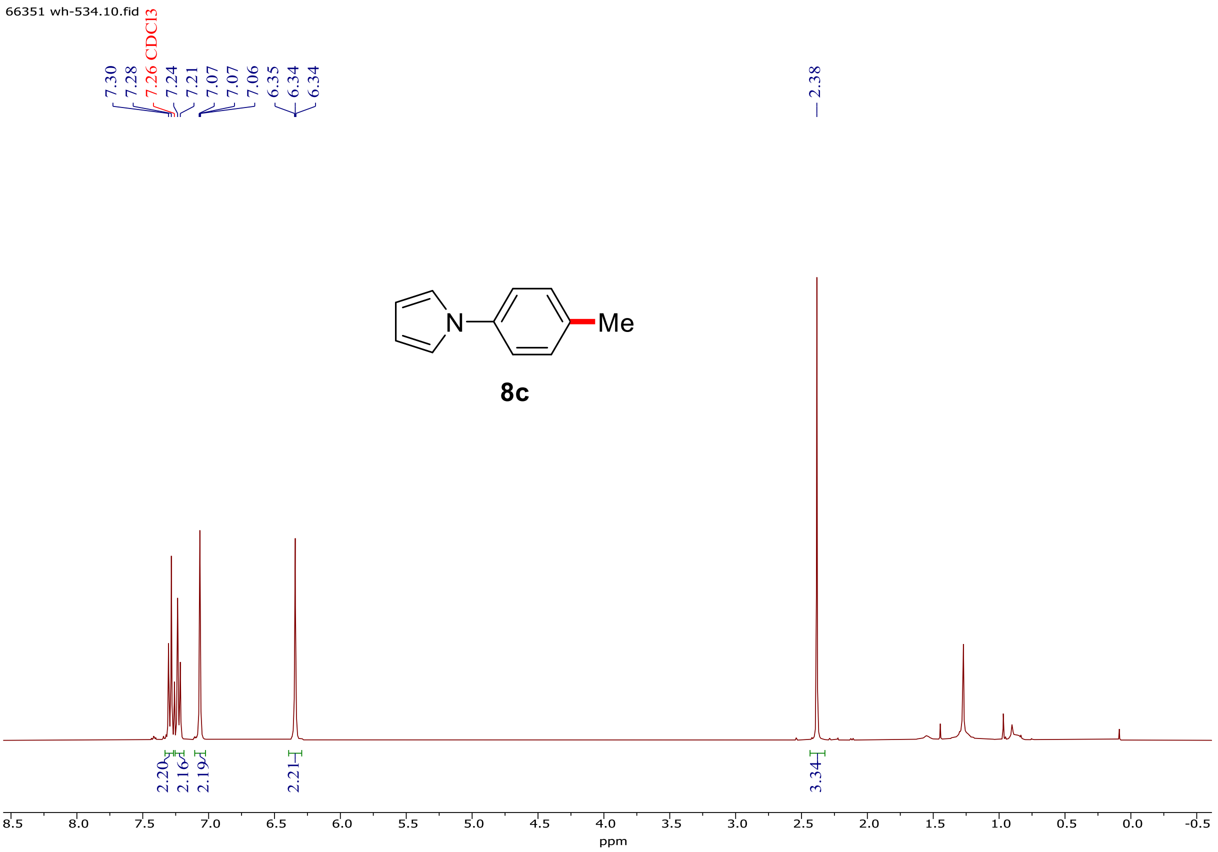


$^{13}$C NMR (101 MHz, $CDCl_3$) of **8c**

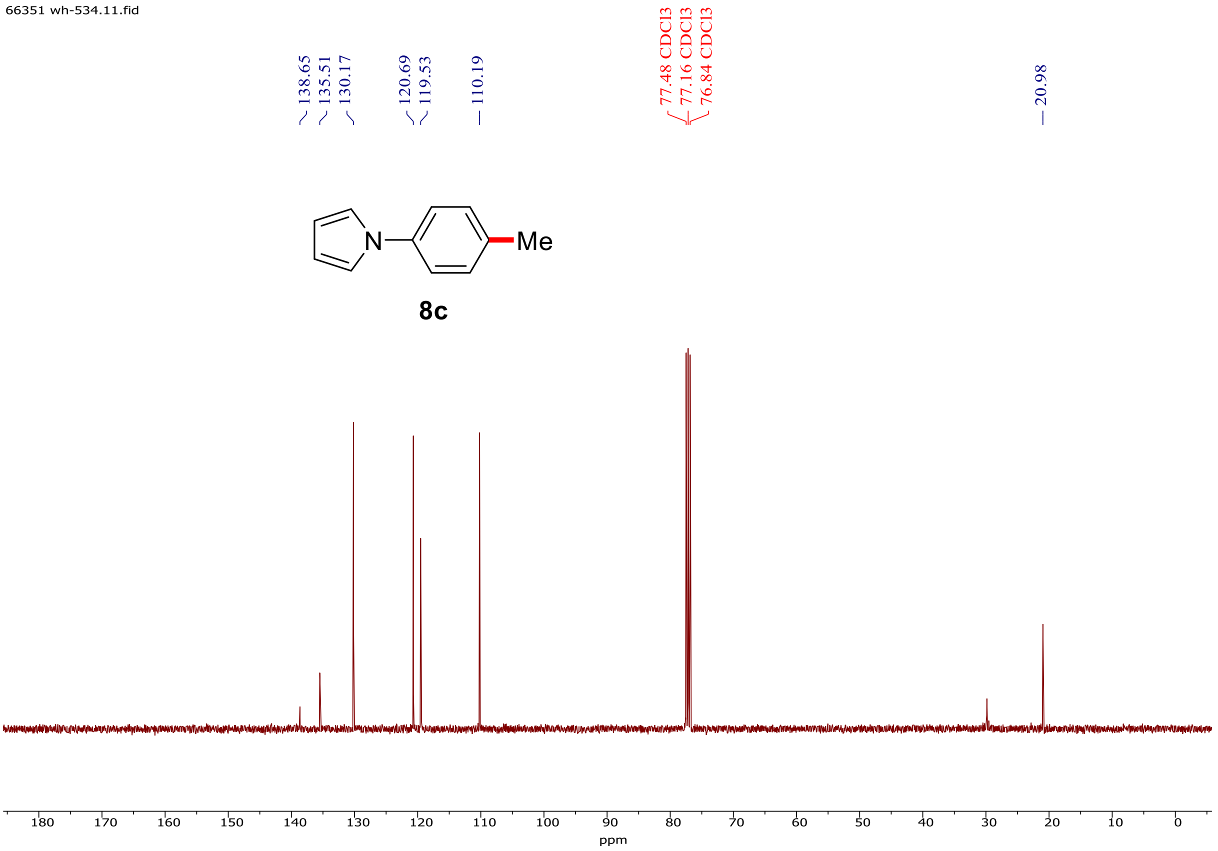

$^{1}H$ NMR (400 MHz, $CDCl_3$) of **9** (*see procedure*)

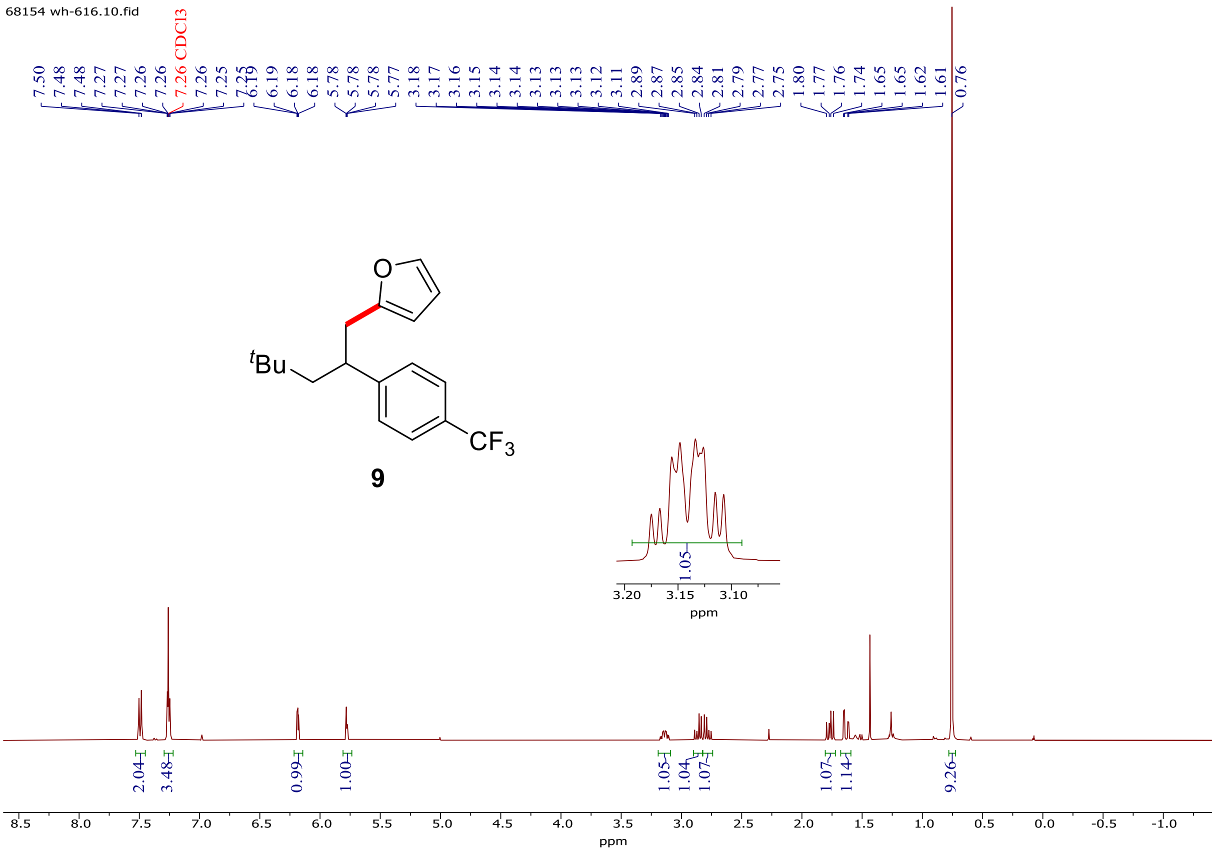


$^{13}C$ NMR (101 MHz, $CDCl_3$) of **9**

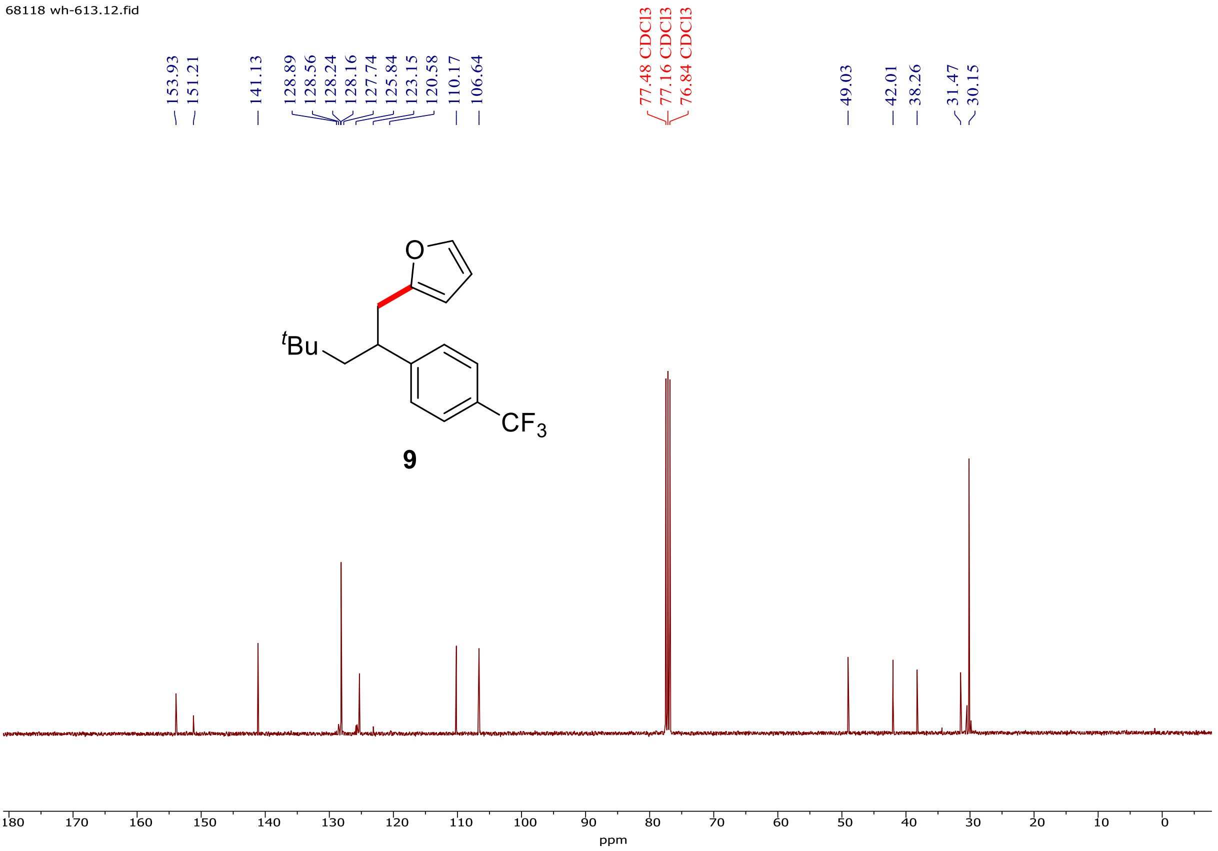

$^{19}$F NMR (376 MHz, $CDCl_3$) of **9**

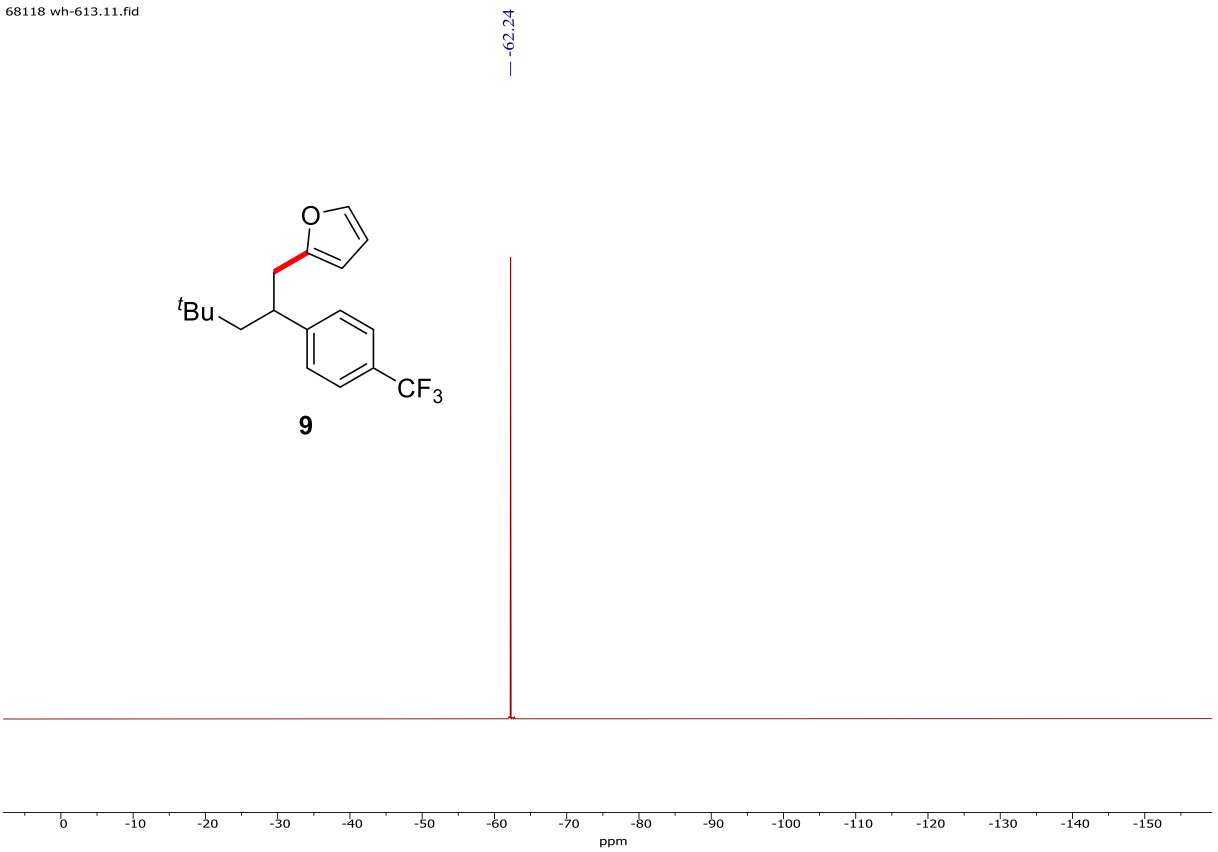


$^{1}$H NMR (400 MHz, $CDCl_3$) of **10** (*see procedure*)

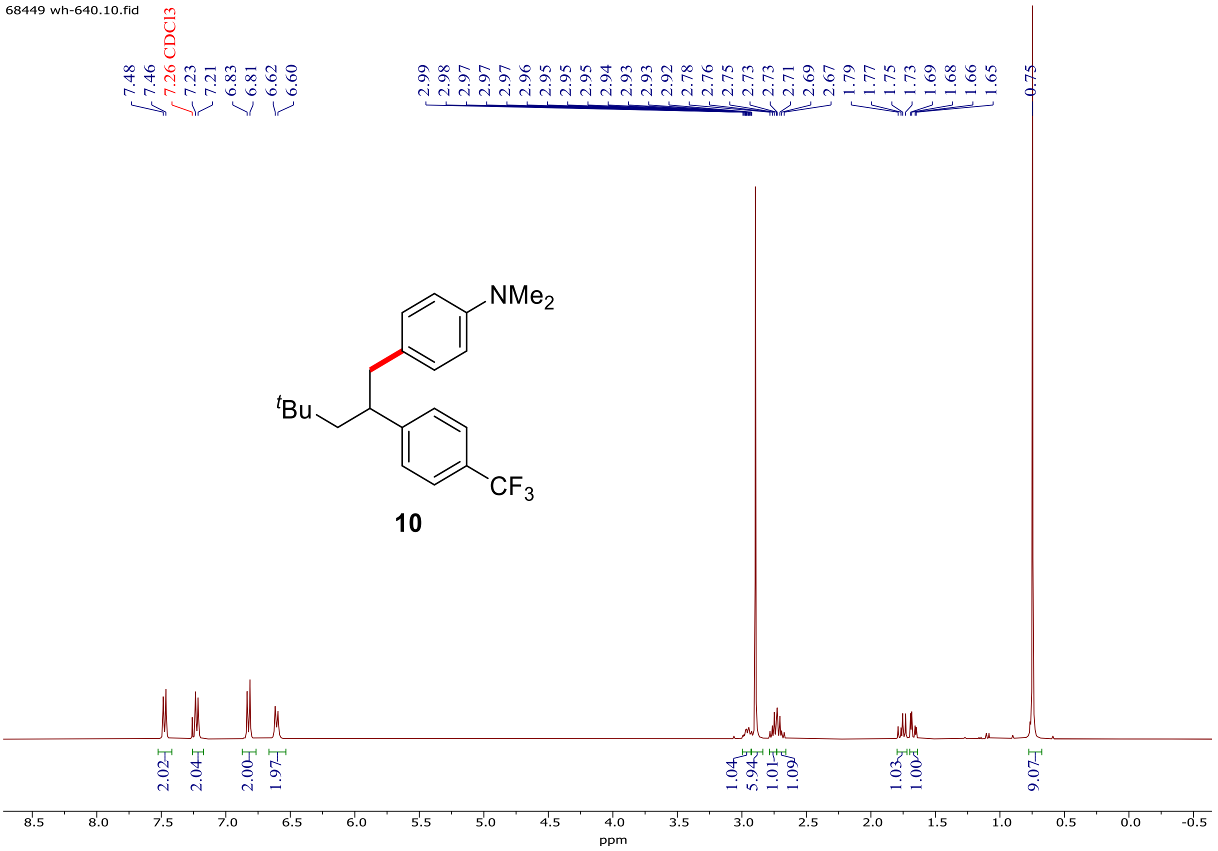

$^{13}C$ NMR (101 MHz, $CDCl_3$) of **10**

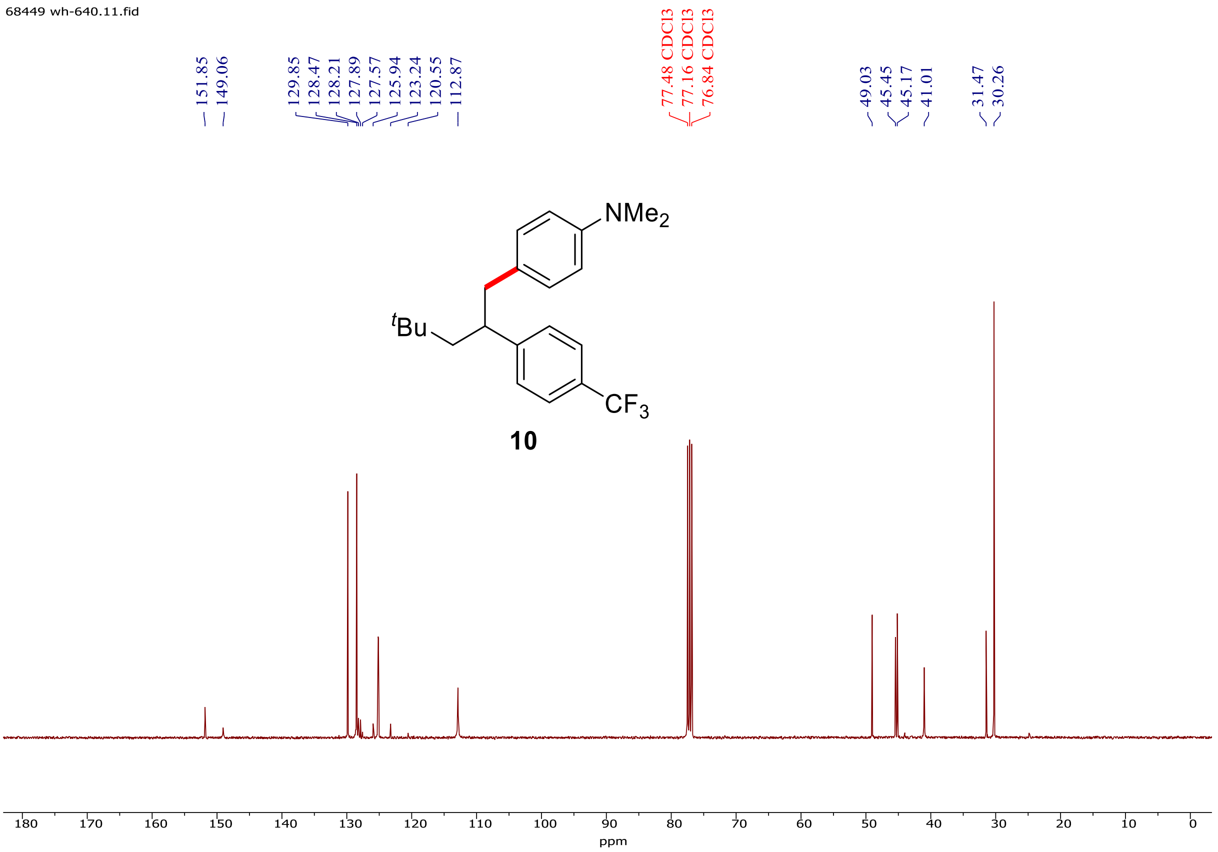


$^{19}F$ NMR (376 MHz, $CDCl_3$) of **10**

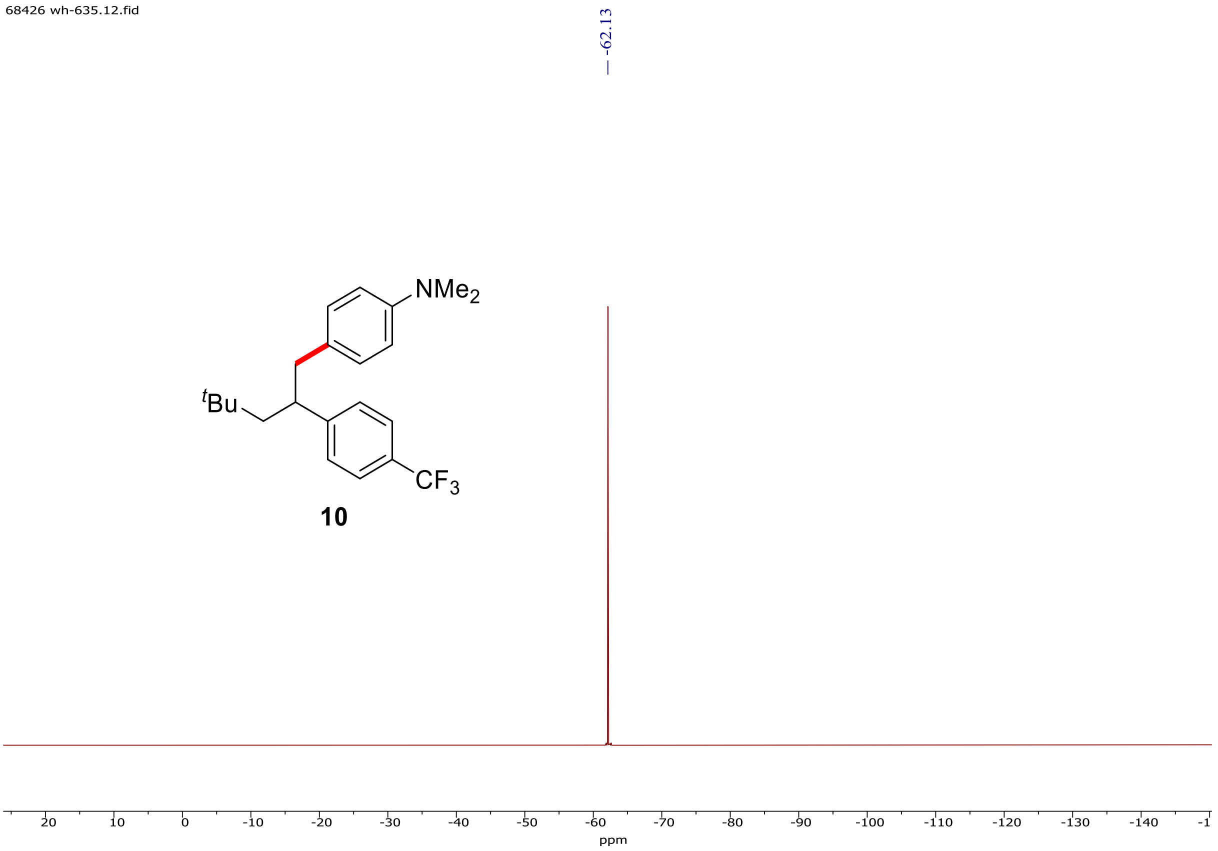

$^{1}H$ NMR (400 MHz, $CDCl_3$) of **11** (see procedure)

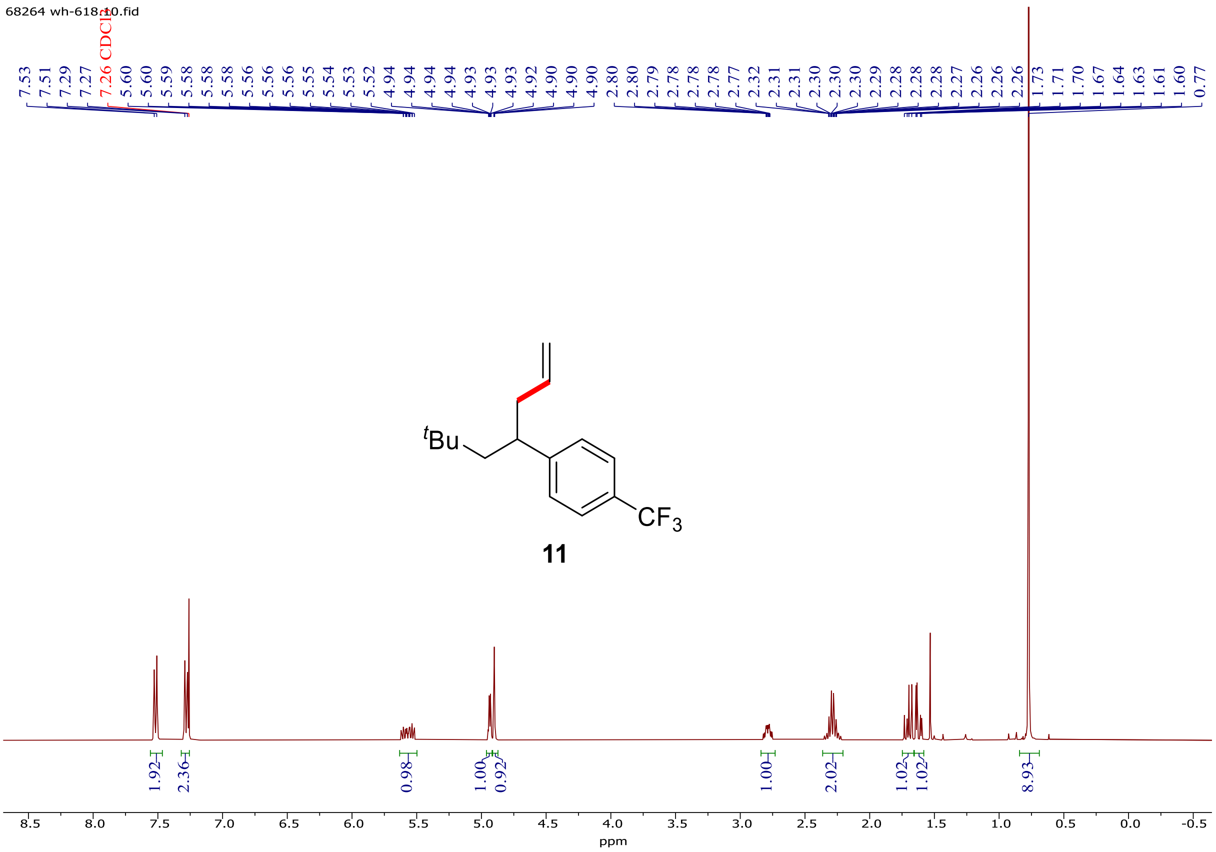


$^{13}C$ NMR (101 MHz, $CDCl_3$) of **11**

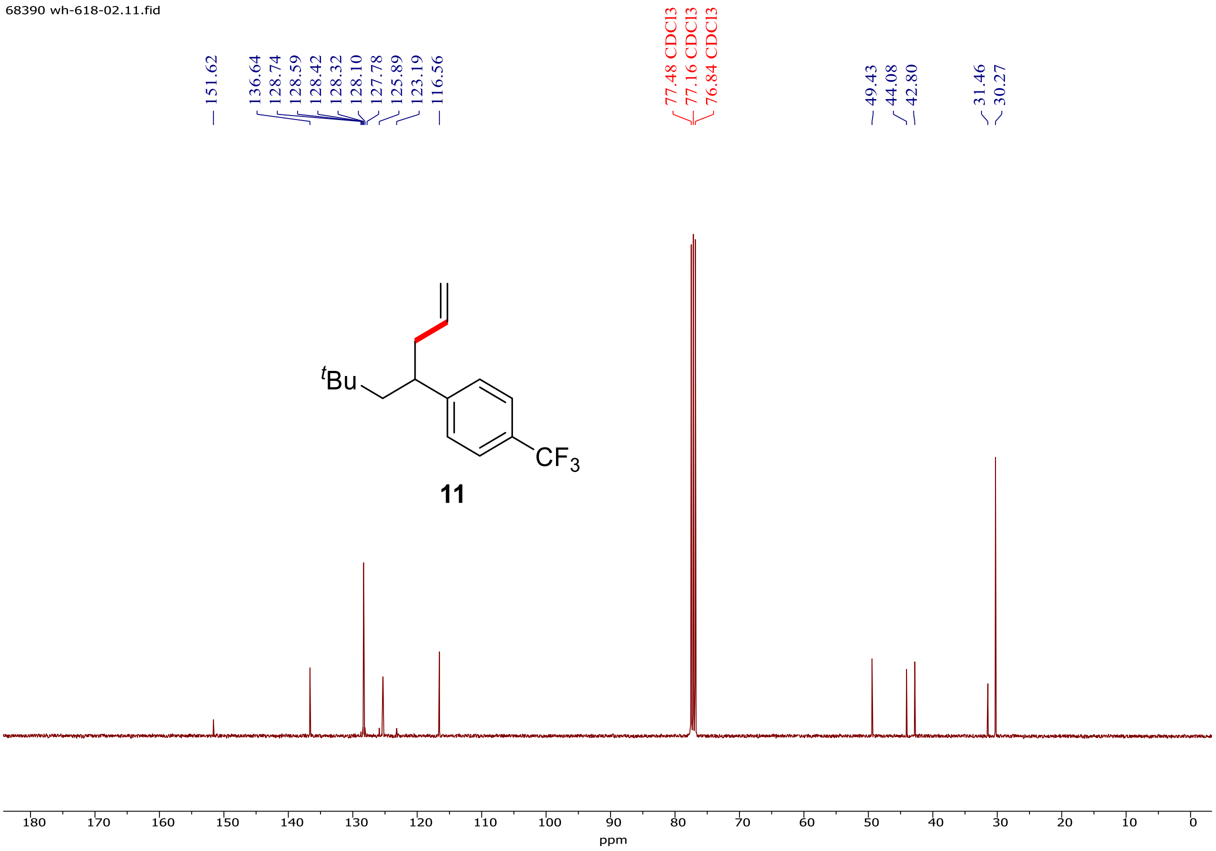

$^{19}F$ NMR (376 MHz, $CDCl_3$) of **11**

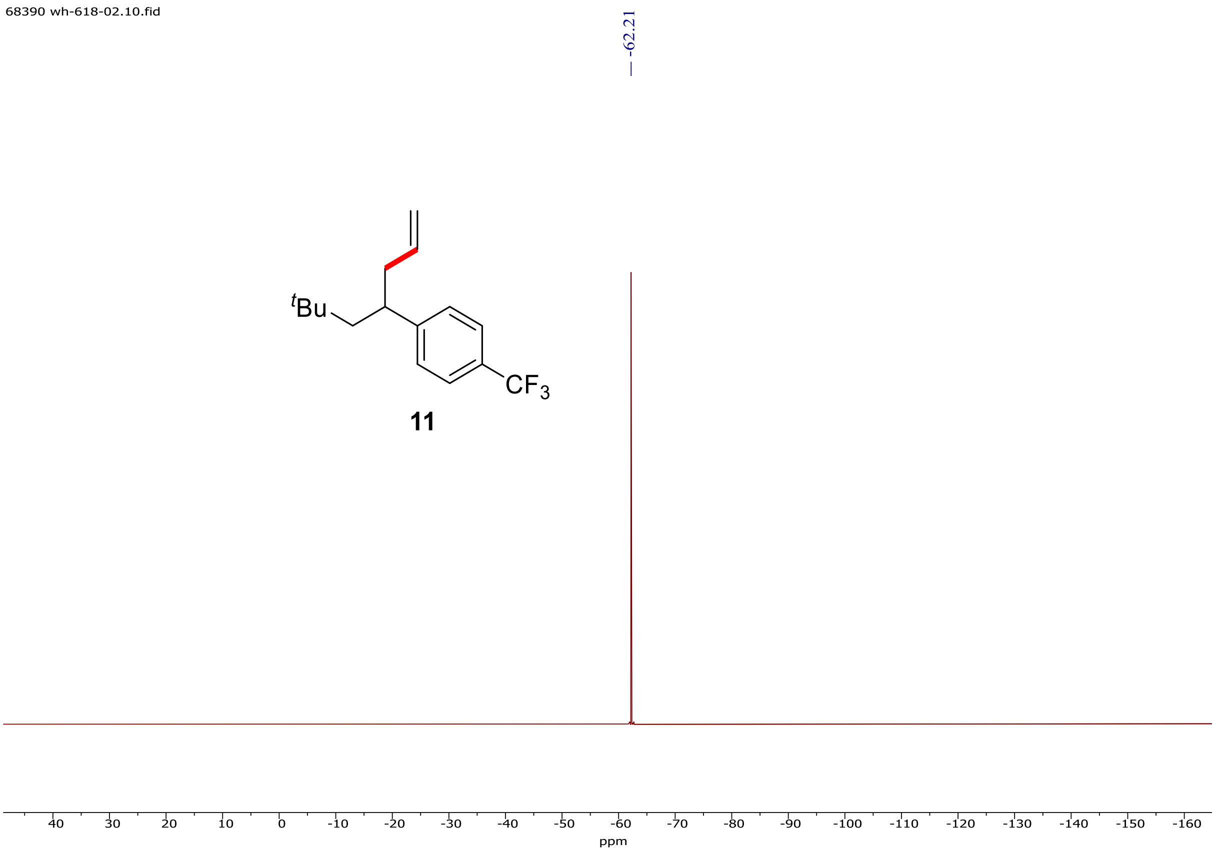


$^{1}H$ NMR (400 MHz, $CDCl_3$) of **12** (*see procedure*)

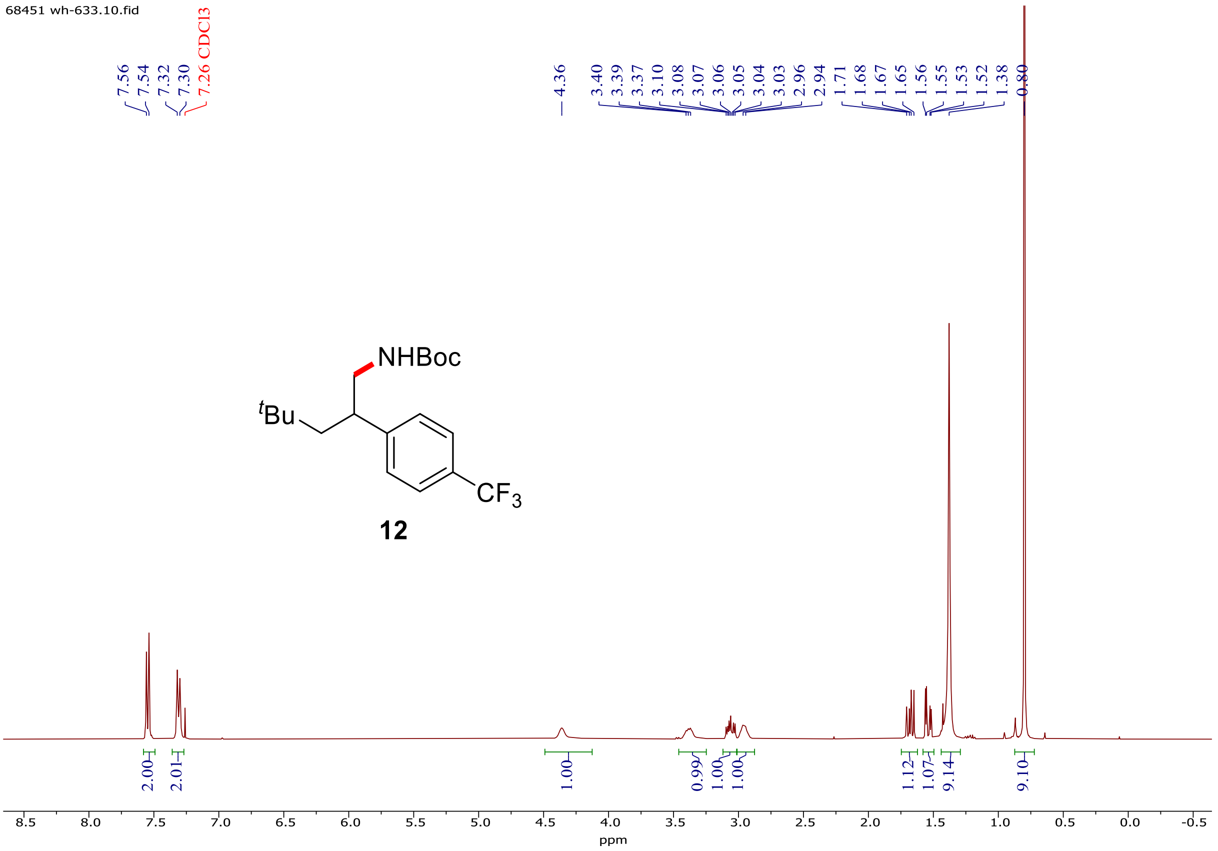

$^{13}$C NMR (101 MHz, $CDCl_3$) of **12**

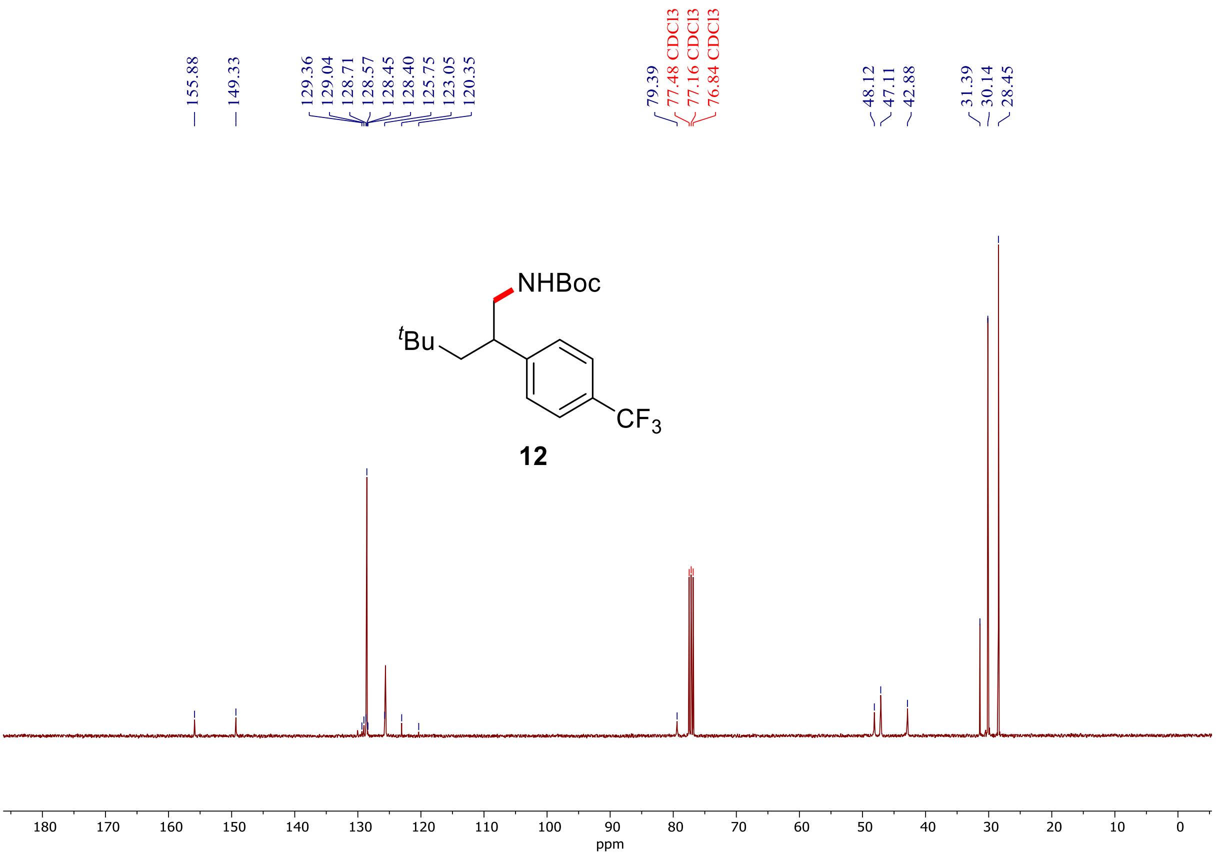


$^{19}$F NMR (376 MHz, $CDCl_3$) of **12**

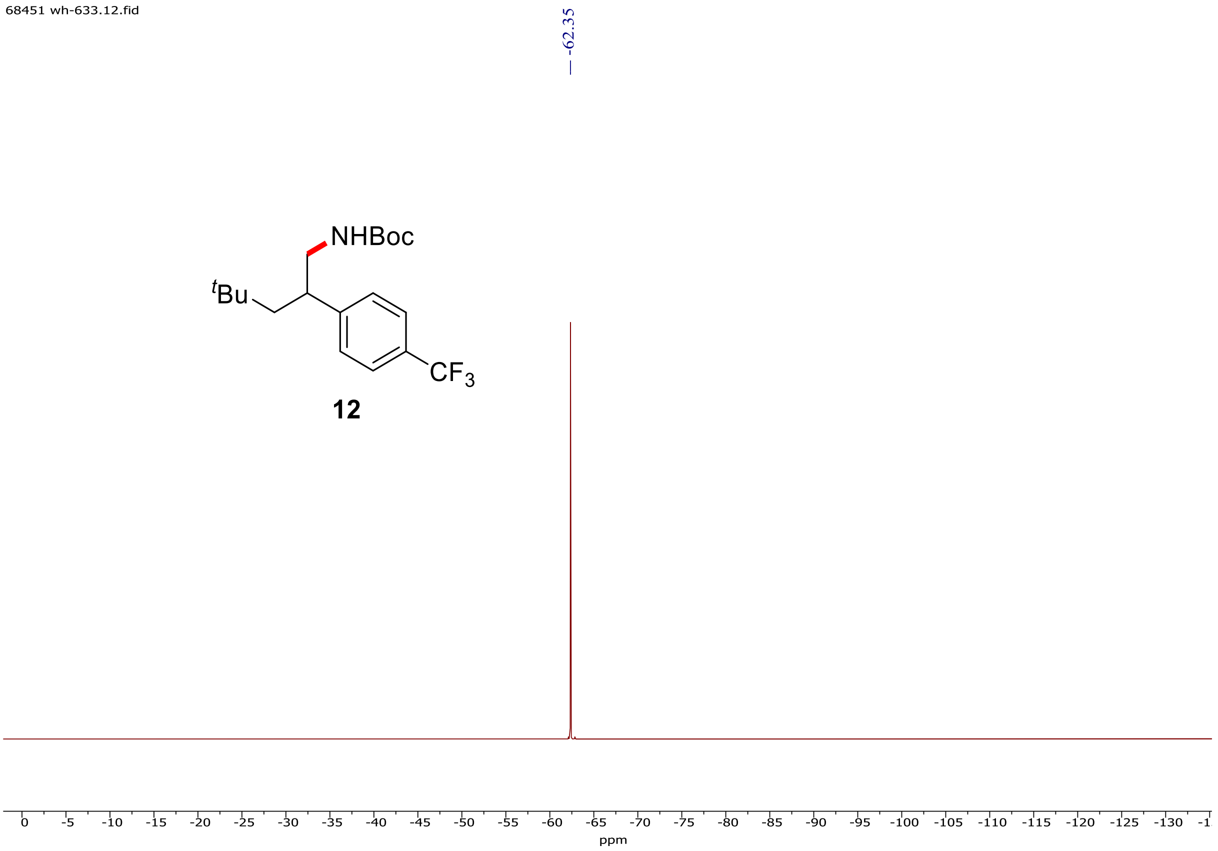

$^1$H NMR (400 MHz, $CDCl_3$) of **13** (*see procedure*)

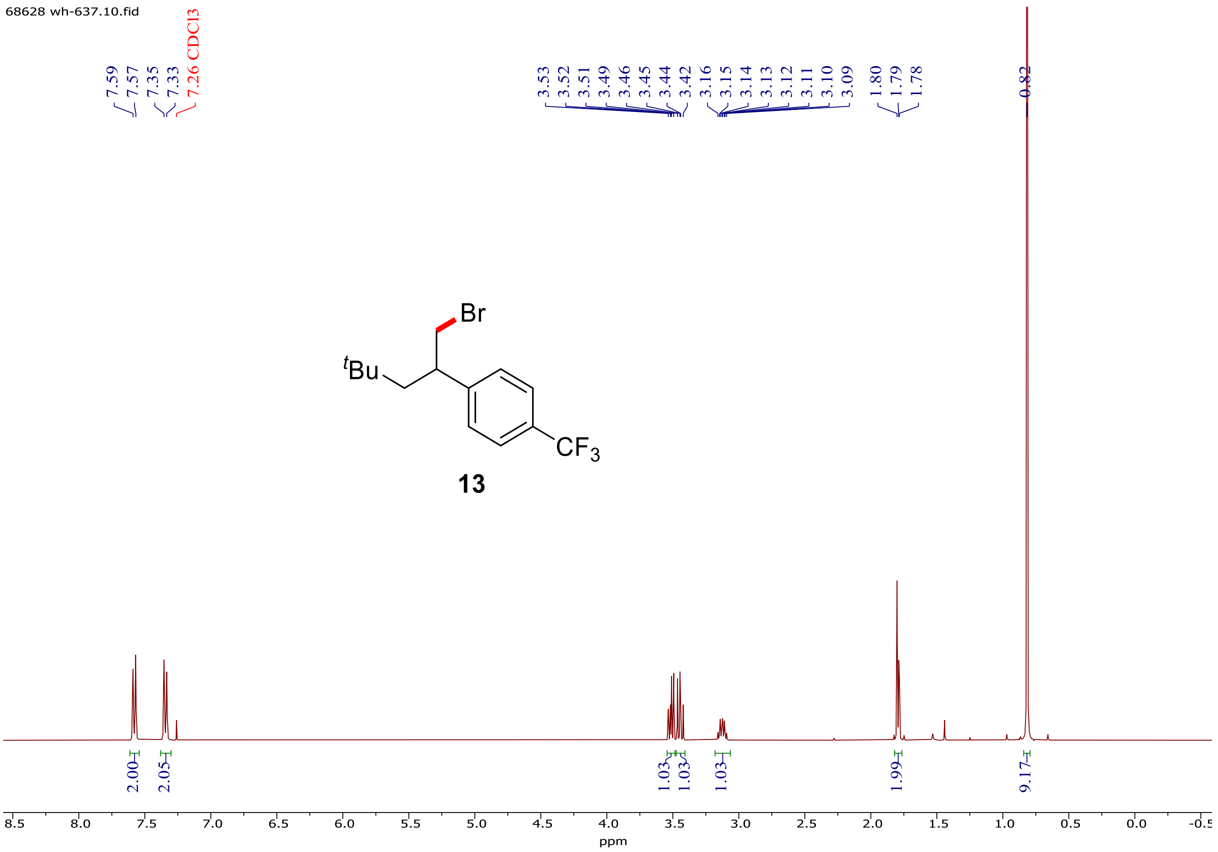


$^{13}$C NMR (101 MHz, $CDCl_3$) of **13**

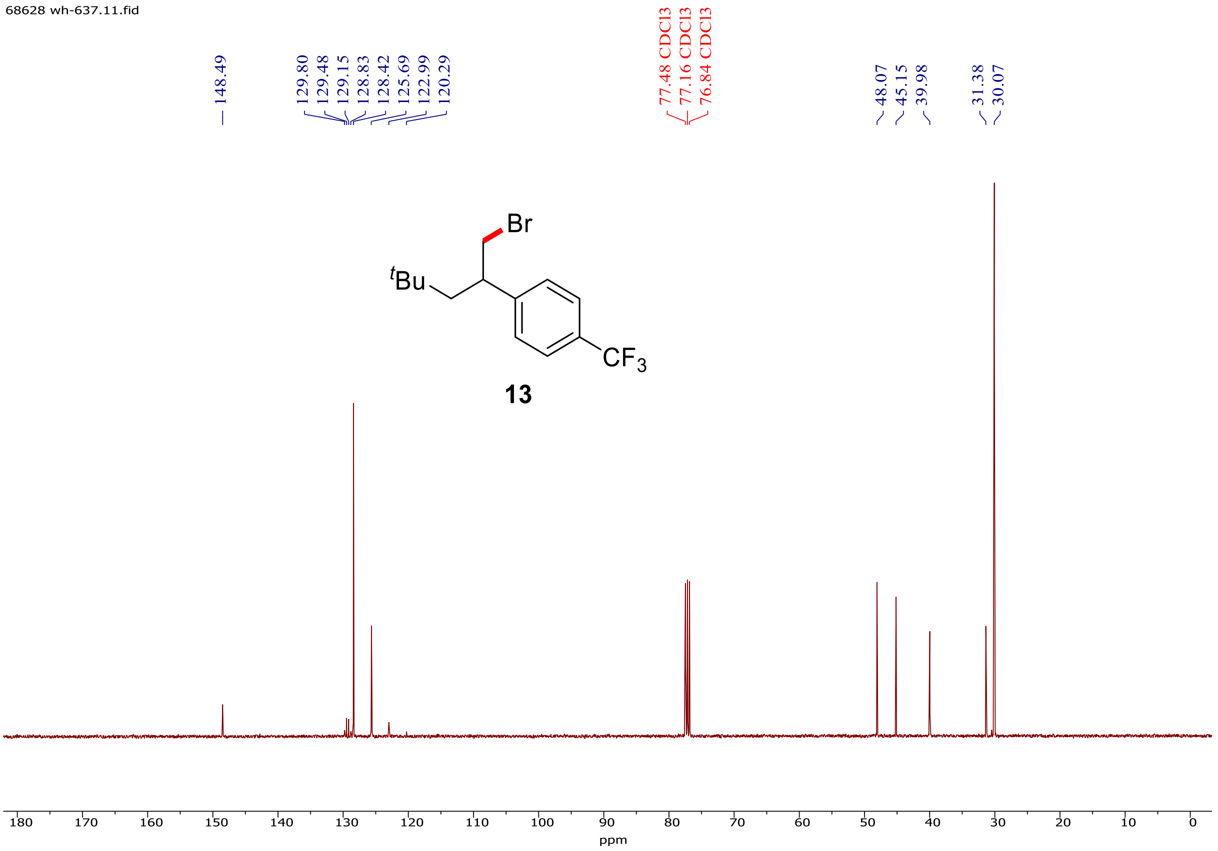

$^{19}$F NMR (376 MHz, $CDCl_3$) of **13**

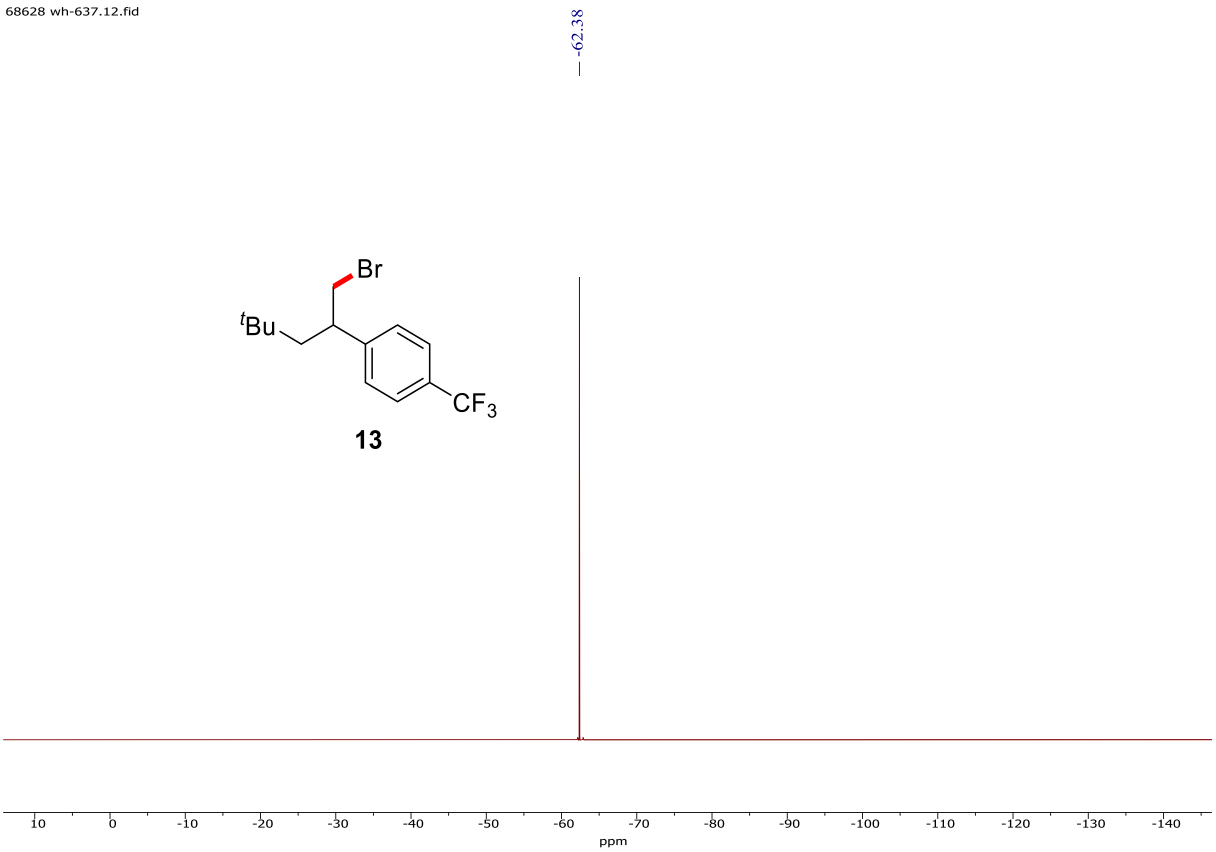


$^{1}$H NMR (400 MHz, $CDCl_3$) of **14** (see procedure)

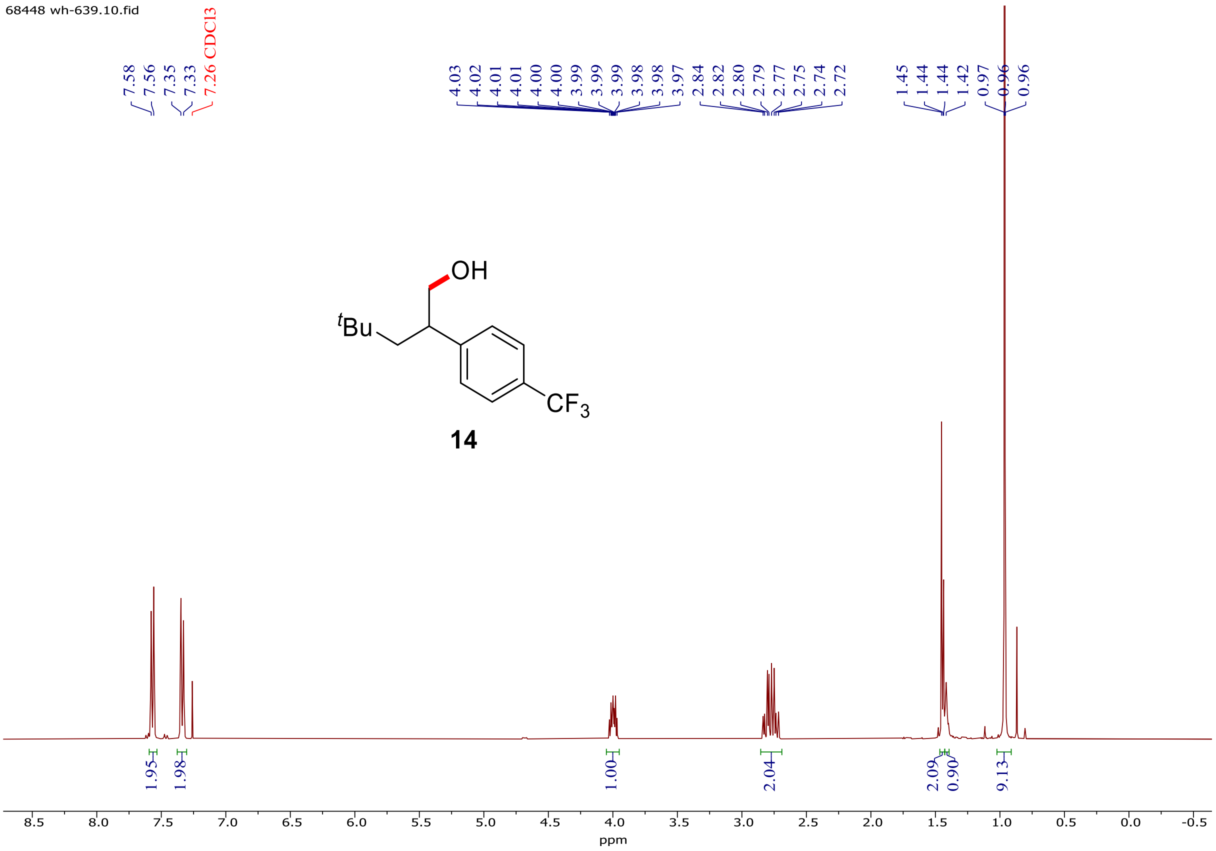

$^{13}C$ NMR (101 MHz, $CDCl_3$) of **14**

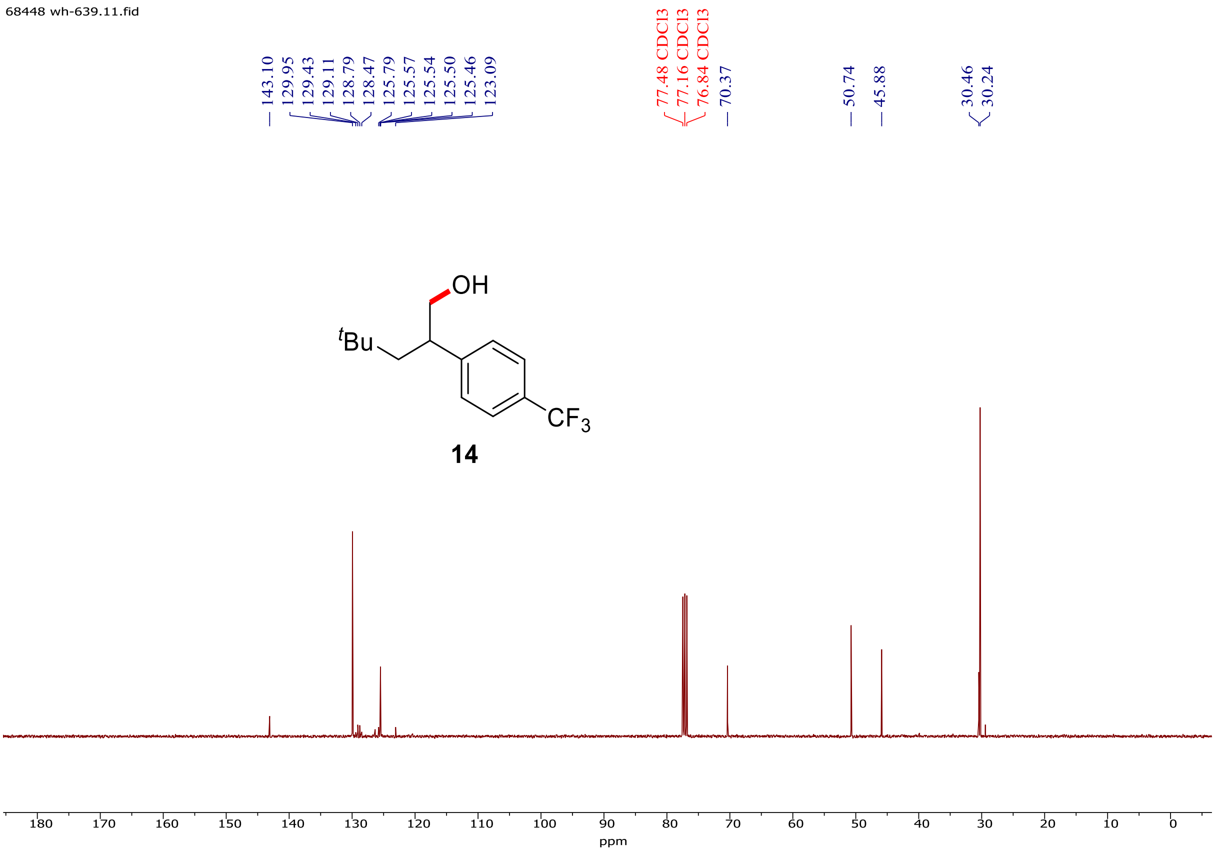


$^{19}F$ NMR (376 MHz, $CDCl_3$) of **14**

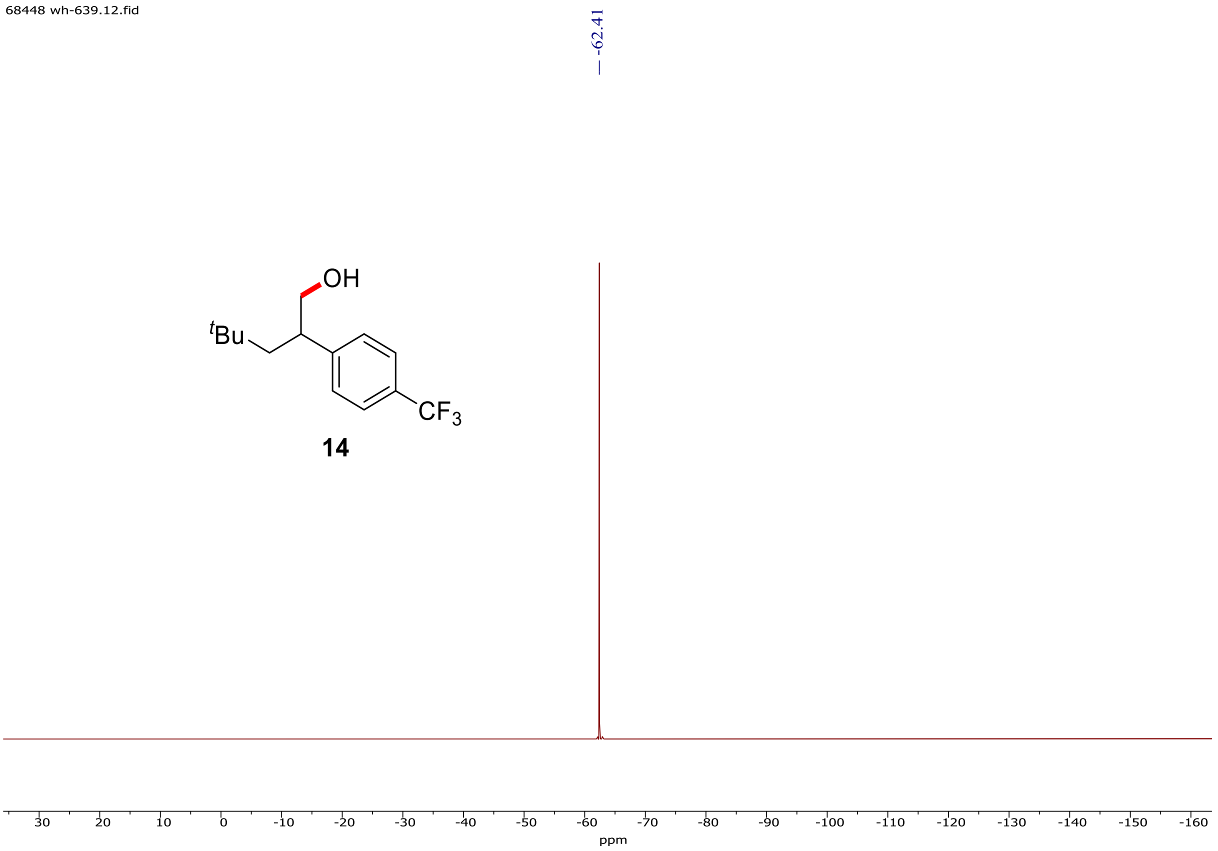

# 5. REFERENCES


[1] A. F. Burchat, J. M. Chong, N. Nielsen, *J. Organomet. Chem.* **1997**, *542*, 281-283.

[2] S. Hitosugi, D. Tanimoto, W. Nakanishi, H. Isobe, *Chem. Lett.* **2012**, *41*, 972-973.

[3] A. Bonet, C. Pubill-Ulldemolins, C. Bo, H. Gulyás, E. Fernández, *Angew. Chem. Int. Ed.* **2011**, *50*, 7158-7161.

[4] D. Kaiser, A. Noble, V. Fasano, V. K. Aggarwal, *J. Am. Chem. Soc.* **2019**, *141*, 14104-14109.

[5] S. Willems, G. Toupalas, J. C. Reisenbauer, B. Morandi, *Chem. Commun.* **2021**, *57*, 3909-3912.

[6] C. Pubill-Ulldemolins, M. Poyatos, C. Bo, E. Fernández, *Dalton Trans.* **2013**, *42*, 746-752.

[7] X.-X. Wang, L. Li, T.-J. Gong, B. Xiao, X. Lu, Y. Fu, *Org. Lett.* **2019**, *21*, 4298-4302.

[8] G. Gao, J. Yan, K. Yang, F. Chen, Q. Song, *Green Chem.* **2017**, *19*, 3997-4001.

[9] P. Zhang, J. Dong, B. Zhong, D. Zhang, H. Yuan, C. Jin, X. Xu, H. Li, Y. Zhou, Z. Liang, M. Ji, T. Xu, G. Song, L. Zhang, G. Chen, X. Meng, D. Sun, J. Shih, R. Zhang, G. Hou, C. Wang, Y. Jin, Q. Yang, *Bioorg. Med. Chem. Lett.* **2016**, *26*, 1910-1918.

[10] F. Schoenebeck, J. A. Murphy, S.-z. Zhou, Y. Uenoyama, Y. Miclo, T. Tuttle, *J. Am. Chem. Soc.* **2007**, *129*, 13368-13369.

[11] H. Woolven, C. González-Rodríguez, I. Marco, A. L. Thompson, M. C. Willis, *Org. Lett.* **2011**, *13*, 4876-4878.

[12] S. D. Dreher, S.-E. Lim, D. L. Sandrock, G. A. Molander, *J. Org. Chem.* **2009**, *74*, 3626-3631.

[13] A. Bonet, M. Odachowski, D. Leonori, S. Essafi, V. K. Aggarwal, *Nat. Chem.* **2014**, *6*, 584-589.

[14] R. J. Armstrong, W. Niwetmarin, V. K. Aggarwal, *Org. Lett.* **2017**, *19*, 2762-2765.

[15] E. K. Edelstein, A. C. Grote, M. D. Palkowitz, J. P. Morken, *Synlett* **2018**, *29*, 1749-1752.

[16] C.-T. Yang, Z.-Q. Zhang, H. Tajuddin, C.-C. Wu, J. Liang, J.-H. Liu, Y. Fu, M. Czyzewska, P. G. Steel, T. B. Marder, L. Liu, *Angew. Chem. Int. Ed.* **2012**, *51*, 528-532.

[17] R. Larouche-Gauthier, T. G. Elford, V. K. Aggarwal, *J. Am. Chem. Soc.* **2011**, *133*, 16794-16797.
